# BrumiR: A toolkit for *de novo* discovery of microRNAs from sRNA-seq data

**DOI:** 10.1093/gigascience/giac093

**Published:** 2022-10-25

**Authors:** Carol Moraga, Evelyn Sanchez, Mariana Galvão Ferrarini, Rodrigo A Gutierrez, Elena A Vidal, Marie-France Sagot

**Affiliations:** Université de Lyon, Université Lyon 1, CNRS, Laboratoire de Biométrie et Biologie Evolutive UMR 5558, F-69622 Villeurbanne, France; Inria Lyon Centre, ERABLE team, 56 Bd Niels Bohr, 69100 Villeurbanne, France; Universidad de O'Higgins, Instituto de Ciencias de la Ingeniería, 2820000 Rancagua, Chile; Centro de Genómica y Bioinformática, Facultad de Ciencias, Ingenieria y Tecnologia, Universidad Mayor, 8580745 Santiago, Chile; Agencia Nacional de Investigación y Desarrollo–Millennium Science Initiative Program, Millennium Institute for Integrative Biology iBio, 7500565 Santiago, Chile; Université de Lyon, Université Lyon 1, CNRS, Laboratoire de Biométrie et Biologie Evolutive UMR 5558, F-69622 Villeurbanne, France; Inria Lyon Centre, ERABLE team, 56 Bd Niels Bohr, 69100 Villeurbanne, France; Université de Lyon, INSA-Lyon, INRA, BF2i, UMR0203, Villeurbanne F-69621, France; Agencia Nacional de Investigación y Desarrollo–Millennium Science Initiative Program, Millennium Institute for Integrative Biology iBio, 7500565 Santiago, Chile; Departamento de Genética Molecular y Microbiología, Facultad de Ciencias Biológicas, Pontificia Universidad Católica de Chile , 8331010 Santiago, Chile; Fondo de Desarrollo de Areas Prioritarias, Center for Genome Regulation, Instituto de Ecología y Biodiversidad, 8370415 Santiago, Chile; Centro de Genómica y Bioinformática, Facultad de Ciencias, Ingenieria y Tecnologia, Universidad Mayor, 8580745 Santiago, Chile; Agencia Nacional de Investigación y Desarrollo–Millennium Science Initiative Program, Millennium Institute for Integrative Biology iBio, 7500565 Santiago, Chile; Escuela de Biotecnología, Facultad de Ciencias, Ingenieria y Tecnologia, Universidad Mayor, 8580745 Santiago, Chile; Université de Lyon, Université Lyon 1, CNRS, Laboratoire de Biométrie et Biologie Evolutive UMR 5558, F-69622 Villeurbanne, France; Inria Lyon Centre, ERABLE team, 56 Bd Niels Bohr, 69100 Villeurbanne, France

**Keywords:** miRNA, Algorithms, de novo

## Abstract

MicroRNAs (miRNAs) are small noncoding RNAs that are key players in the regulation of gene expression. In the past decade, with the increasing accessibility of high-throughput sequencing technologies, different methods have been developed to identify miRNAs, most of which rely on preexisting reference genomes. However, when a reference genome is absent or is not of high quality, such identification becomes more difficult. In this context, we developed BrumiR, an algorithm that is able to discover miRNAs directly and exclusively from small RNA (sRNA) sequencing (sRNA-seq) data. We benchmarked BrumiR with datasets encompassing animal and plant species using real and simulated sRNA-seq experiments. The results demonstrate that BrumiR reaches the highest recall for miRNA discovery, while at the same time being much faster and more efficient than the state-of-the-art tools evaluated. The latter allows BrumiR to analyze a large number of sRNA-seq experiments, from plants or animal species. Moreover, BrumiR detects additional information regarding other expressed sequences (sRNAs, isomiRs, etc.), thus maximizing the biological insight gained from sRNA-seq experiments. Additionally, when a reference genome is available, BrumiR provides a new mapping tool (BrumiR2reference) that performs an *a posteriori* exhaustive search to identify the precursor sequences. Finally, we also provide a machine learning classifier based on a random forest model that evaluates the sequence-derived features to further refine the prediction obtained from the BrumiR-core. The code of BrumiR and all the algorithms that compose the BrumiR toolkit are freely available at https://github.com/camoragaq/BrumiR.

## Introduction

MicroRNAs (miRNAs) are small RNA molecules usually shorter than 25 nucleotides (nt), which have been identified as crucial regulators of gene expression mostly at the posttranscriptional level [1]. miRNAs are involved in a wide range of biological processes, including cell cycle, differentiation, apoptosis, and disease [2]. They have been the target molecules for a large number of important applications, more particularly in cancer, in which miRNAs have been shown to play important roles in driving or suppressing tumor spread [3, 4]. In plant species, unraveling host–pathogen interactions mediated by miRNAs may shed light on plant development and its relation with the environment, both essential knowledge that can lead to the discovery of new biotechnological products for the agricultural industry [5, 6].

Since the first classification and annotation of miRNAs in *Caenorhabditis elegans* [7, 8], thousands of miRNAs have been discovered in plants, animals, and other eukaryotes. Most eukaryotic miRNAs are transcribed by RNA polymerase II [9–11], while some of them are transcribed by RNA polymerase III in animals [12]. Long precursor RNAs are folded into hairpin-like structures consisting of a terminal loop, an upper stem, the miRNA duplex region, and a lower stem and 2 arms, and they are processed in the cytoplasm, generating the miRNA/miRNA* duplex that is subsequently divided into the star and the functional mature miRNA sequence [13]. Mature miRNA processing pathways differ between animals and plants. One major difference is the length of the precursor sequences, with plant precursors longer than those of animals [14]. The mature miRNA sequences act as guides, leading the RISC complex to target RNAs to regulate their expression by transcript cleavage or translation inhibition [15, 16]. Therefore, accurate prediction of known and novel miRNAs along with their targets is essential for increasing our understanding of the miRNA biology [4, 17]. However, it has proven difficult to accurately characterize and predict the miRNAs as well as their regulatory networks [18, 19].

Nowadays, a common experimental practice is to identify miRNAs and their expression patterns using next-generation sequencing (NGS) technologies [20]. Commonly, NGS experiments are able to generate more than 20 million small RNA (sRNA) sequencing (sRNA-seq) reads, thus promoting the development of algorithms to transform and process such data into biological information [21].

Currently, there are 2 computational strategies for the discovery of miRNAs: (i) genome-based approaches that rely on the mapping of the sRNA-seq reads to a reference genome and subsequent evaluation of the sequences generating the characteristic hairpin structure of miRNA precursors [18] and (ii) machine learning approaches that rely on the biogenesis features extracted from the knowledge on miRNA sequences available in databases such as miRBase [22] and on the analysis of the duplex structure of miRNAs [23]. Genome-based methods, which have been updated at the pace of the evolving NGS technologies, are the most widely used tools in this field, and their results have populated the public miRNA repositories [21]. Such methods are the natural choice for the study of model species with high-quality reference genomes available. However, it has been shown that most of the genome-based tools struggle with a high rate of false-positive predictions when they rely only on the reference genome and do not leverage on sRNA-seq data [18]. Additionally, a critical step of such tools is the use of genome aligners [24, 25] to map the sRNA-seq reads to the reference genome. Mapping short (<30 nt) and very similar sequences to a large, complex, and repetitive reference genome is, however, a difficult and error-prone task [26]. Genome-based methods are thus highly sensitive to the aligner selected as well as to the parameters employed and the thresholds chosen (e.g., number of mismatches allowed) in order to discard mapping artifacts generated from sequencing errors [27]. Furthermore, despite all the advancements in the sequencing technologies and *de novo* assembly methods, few complete genomes are available today, which is a recurring problem that researchers working on nonmodel species face [28]. The lack of a high-quality reference genome thus reduces the possibilities for discovering novel miRNAs [23]. Genome-based methods such as miRDeep [29], miRDeep2 [30], and miR-PREFeR [31] are included in this group.

On the other hand, new methods such as miReader [32], MirPlex [33], and mirnovo [23], particularly using machine learning approaches, were specifically developed as an alternative to discover miRNAs in species without a reference genome. In the case of mirnovo, the initial step involves the clustering of the sRNA-seq reads performing an all-versus-all read comparison that is followed by a subsequent classification of the clusters into putative miRNAs using pretrained models. The performance obtained by such methods on well-annotated species is comparable to those achieved by genome-based methods [18]. However, relying exclusively on annotated miRNAs for training machine learning models may introduce a bias toward the identification of well-characterized miRNAs over species-specific ones [21]. Nonetheless, machine learning methods have demonstrated that it is possible to discover miRNAs using only the sequence information present in the sRNA-seq experiment [23].

There remains, however, a need to go further in the development of algorithms for finding novel miRNAs in nonmodel species using only the sequence information. With this purpose in mind, the adoption of a special type of graphs, called *de Bruijn* graphs, may be considered. This is a widely used approach for the *de novo* reconstruction of genome or transcriptome sequences [34]. It therefore appears to be a plausible option for organizing, clustering, and assembling the sequence information present in sRNA-seq experiments. However, accommodating the de Bruijn graph approach for the discovery of miRNAs involves the development of new methods to address the specific characteristics of sRNA-seq data. Indeed, mature miRNA sequences are short (18–24 nt), thus limiting the overlap length for building a de Bruijn graph, which in turn impacts the global topology by inducing tangled graph structures. Moreover, miRNAs captured in a sRNA-seq experiment have variable expression, from low (few reads) to highly expressed (thousands of reads), which may induce spurious graph connections that should be removed in order to isolate and detect both types of miRNAs. Finally, the sequencing errors present in sRNA-seq data further induce spurious connections and are harder to detect as compared to genomic data due to the variable expression and the shorter lengths of the miRNAs. Overall, using a de Bruijn graph to analyze sRNA-seq data and extract information from such data seems thus counterintuitive as mature miRNAs are captured full length by the current NGS technologies. However, a de Bruijn graph has several interesting properties for the discovery of miRNAs, mainly due to the fact that it encodes all the sRNA-seq sequence information at once in a compact and connected representation (graph), without the need to perform an all-versus-all read comparison or mapping to a reference.

In this article, we present BrumiR, a *de novo* algorithm based on a de Bruijn graph approach that is able to identify miRNAs directly and exclusively from sRNA-seq data. Unlike other state-of-the-art algorithms, BrumiR does not rely on a reference genome, on the availability of close phylogenetic species, or on conserved sequence information. Instead, BrumiR starts from a de Bruijn graph encoding all the reads and is able to directly identify putative miRNAs on the generated graph. BrumiR also removes sequencing errors and navigates inside the graph, detecting putative miRNAs by considering several miRNA biogenesis properties (such as expression, length, topology in the graph). Along with miRNA discovery, BrumiR can also assemble and identify other types of small and long noncoding RNAs expressed within the sequencing data. Finally, when a reference genome is available, BrumiR provides a new mapping tool (BrumiR2ref) that performs an exhaustive search to identify and validate the precursor sequences.

We extensively benchmarked BrumiR on animal and plant species using simulated and real datasets. The benchmark results demonstrate that BrumiR is very sensitive, besides being the fastest tool, and its predictions were supported by the characteristic hairpin structure of miRNAs. Finally, we also applied BrumiR to the discovery of miRNAs of *Arabidopsis thaliana* and identified 3 novel high-confidence miRNAs involved in root development. These putative miRNAs were not discovered before by any other software, thereby showing the potential of using different approaches even in the case where high-quality genomes are available. The code of BrumiR is freely available at https://github.com/camoragaq/BrumiR.

## Results

### BrumiR discovers mature miRNAs directly from the sRNA-seq reads

**Figure 1:**
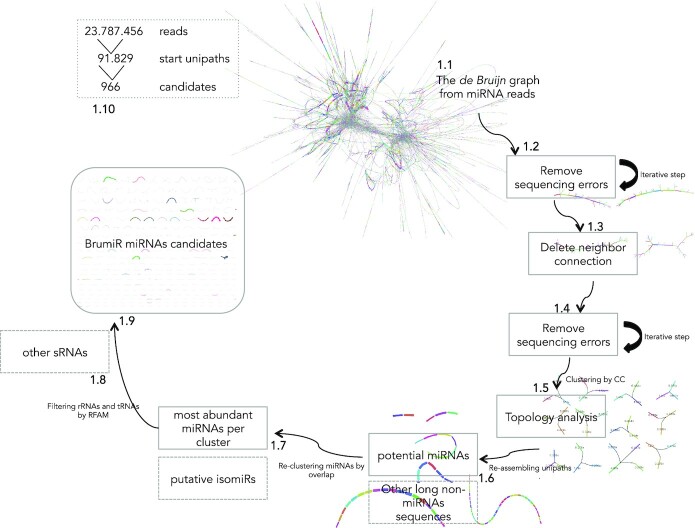
BrumiR algorithm. Different steps of BrumiR to discover miRNAs from sRNA-seq data. **1.1** De Bruijn graph step. **1.2** Tips removal iterative step. **1.3** Delete neighbor connection step. **1.4** Tips removal step repetition. **1.5** Topology analysis step. **1.6** Reassembling unipaths by CC step. **1.7** Reclustering by overlap step. **1.8** Filtering other sRNAs by RFAM step. **1.9** BrumiR candidates catalog.

The main idea behind BrumiR is that mature miRNAs can be discovered directly from the information contained in the sequenced sRNA-seq reads. To achieve this, BrumiR starts by building a de Bruijn graph directly from the sRNA-seq reads, using *k*-mers of size 14 and a depth of coverage of 50, then compacting all the simple nodes, thus leading to the unipath graph [35] (Fig. 1, Materials and Methods section). The unipath graph encodes all the sequence information of the sRNA-seq experiment, including sequencing errors, adapters, and other types of sequences (Fig. 1). The construction of the unipath graph allows avoiding entirely the alignment of the sRNA-seq reads to a reference genome. Following the unipath graph construction, BrumiR cleans the graph by removing tips (dead-end nodes) with low expression/abundance (KM <5), which are usually generated from sequencing errors (Fig. 1). One feature of the miRNA biogenesis is that after Dicer cleavage, the mature miRNA is the most abundant of the 3 by-products, and when it is sequenced, it has a uniform expression along its sequence [29]. Therefore, BrumiR expects that the neighbor elements within a particular putative miRNA will have similar expression. BrumiR checks all neighbor connections (arcs) and deletes any connection with a relative expression difference larger than 3-fold (Fig. 1, Materials and Methods section), and the new graph is cleaned again by removing tips (Fig. 1). Clusters of unipaths (connected components) with topologies related to sequencing errors are also removed (Fig. 1, Materials and Methods section).

BrumiR attempts to reassemble all unipaths within a connected component (CC) of the graph, and those with between 18 and 24 nt are classified as putative miRNAs, while longer reassembled unipaths (>24 nt) are classified as other longer sequences (Fig. 1). BrumiR then restores missing connections by reclustering the putative miRNAs performing an all-versus-all comparison. The most expressed miRNA is selected as the representative of the cluster (Fig. 1), and the remaining members are classified as potential isomiRs (Fig. 1). The final BrumiR step uses the RFAM database [36] to discard predicted miRNAs matching to other classes of RNA (e.g., ribosomal genes, Fig. 1). We build a 16-mer database using RFAM database excluding any reference to known miRNA sequences, in a similar way as mirnovo does [23]. As an example, BrumiR reduces the input sRNA-seq data by 5 orders of magnitude, generating fewer than 1,000 putative mature miRNAs (24 million input reads to 966 miRNA candidates; see Fig. 1). Finally, BrumiR outputs several FASTA files with all predicted mature miRNAs, all longer RNAs, putative isomiRs, other sRNAs (RFAM comparison), and a table with expression values for each predicted miRNA. Additionally, BrumiR outputs the final graph in a GFA format, which can be explored using Bandage [37] (Supplementary Fig. S11).

### BrumiR achieves the highest accuracy on simulated data

**Figure 2:**
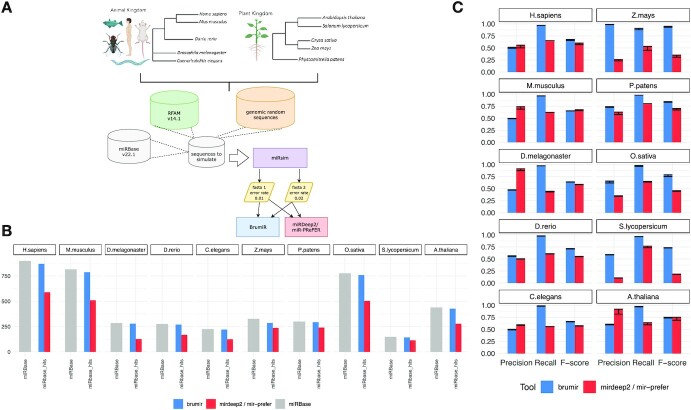
Synthetic benchmarking between BrumiR and miRDeep2. (A) Workflow and species selected. (B) miRBase input versus miRNA true-positive predictions for each tool (2 samples). (C) Benchmarking metrics for all datasets tested. The error bar indicates the distance between the 2 replicates.

To evaluate the performance of BrumiR, we applied it to discover mature miRNAs on simulated sRNA-seq reads from 10 animal and 10 plant species (Fig. 2A). We compared BrumiR to the state-of-the-art genome-based miRNA discovery tools miRDeep2 [30] and miR-PREFeR [31], which were developed specifically for animal (miRDeep2) and plant (miR-PREFeR) species. For each tested species, we generated 2 synthetic datasets with different error rates (0.01 and 0.02) using the miRsim tool implemented and provided by the BrumiR toolkit (https://github.com/camoragaq/miRsim). To simulate the reads, we used (i) the high-confidence miRNAs annotated in the miRBase database [22], (ii) sequences from the RFAM database (v14.1) [38] to simulate possible fragments from other known types of RNAs present in the sRNA-seq data, and (iii) random genomic sequences for each of the species included in the benchmark (see the Materials and Methods section). A total of 20 datasets with an average of 13.6 million reads were simulated. The list of simulated miRNAs was considered as the ground truth, and benchmark metrics (Fig. 2C) were computed to assess the performance of BrumiR and of the other software (see Materials and Methods section) (Supplementary Table S2).

BrumiR recovered more mature miRNAs than the others, on average 97% (opposed to 58% and 66% for miRDeep2 and miR-PREFeR, respectively), and presented the highest average recall across all the simulated datasets (Fig. 2B). BrumiR recovered more than 90% of the simulated mature miRNAs in 19 of the 20 simulated datasets (Fig. 2B). In particular in the *Homo sapiens* and *Drosophila melanogaster* datasets, BrumiR recovered 1.5× and 2.5× more candidates than MiRDeep2 (Fig. 2B). As concerns precision, BrumiR tended to generate more putative candidates than MiRDeep2 (median 659 vs. 332) and less than MiR-PREFeR (median 474 vs. 649). The slightly higher number of BrumiR candidates resulted in lower average precision than miRDeep2 for animal species (0.51 vs. 0.65) but was significantly higher as compared to MiR-PREFeR for plants (0.71 vs. 0.43). The lower precision achieved in animal species might be due to the fact that BrumiR does not use the hairpin structure filter employed by miRDeep2. If we consider both precision and recall (F-score), BrumiR was the top performer in 17 of the 20 datasets evaluated (Fig. 2C). With animal species, BrumiR always reached a higher F-score than miRDeep2 except for *Mus musculus*. With plant species, BrumiR was better than miR-PREFeR on most datasets, with BrumiR reaching a higher F-score in 9 of the 10 datasets (Fig. 2C).

In terms of computational time, BrumiR was the fastest method. In particular, BrumiR-core was on average 21× faster than miRDeep2 and 6× times faster than MiR-PREFeR (see Supplementary Table S3). The speed of BrumiR relies on efficient alignment-free and graph-based approaches.

Overall, we demonstrated with simulated data that BrumiR discovers putative mature miRNAs without a reference genome across different eukaryotic species, achieving the highest accuracy and computational efficiency.

### The hairpin structure of mature miRNAs is found in most of the BrumiR candidates

**Figure 3:**
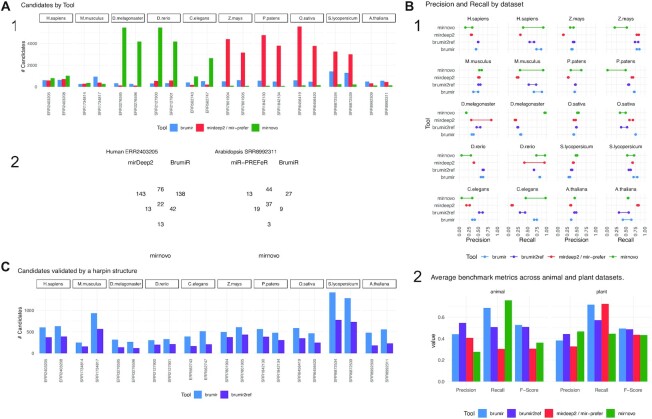
Real dataset benchmark of BrumiR and state-of-the-art tools. (A) Number of predictions by tool for all the datasets and the overlap between them for 2 datasets (1 for animal and 1 for plant). (B) Benchmarking metrics computed using miRBase annotated miRNAs, precision and recall for each dataset, and average metrics, including F-score. (C) BrumiR candidates validated by hairpin structure (BrumiR2reference).

In order to assess the performance of BrumiR on real data, we collected public datasets for the same plant and animal species evaluated in the synthetic benchmark (Fig. 2A). On average, 15.4 and 18.2 raw million reads were used for the animal and plant datasets (Supplementary Table S4), respectively. The predictions of BrumiR were compared against those of the state-of-the-art tools encompassing reference- and *de novo*–based methods [23, 30, 31], and after testing some of the most used miRNA discovery tools, we selected the best performer (Supplementary Table S5, Supplementary Fig. S9). In particular, we included mirnovo that, similar to BrumiR, can discover mature miRNAs directly from the reads. Before running the tools, low-quality reads were removed using fastp [39] (∼10%; see Materials and Methods section). All the predicted miRNAs for each tool were annotated using the miRBase database to identify known and novel predictions. On average, BrumiR predicted ∼450 putative mature miRNAs for the animal species, which was ∼0.8× higher than the miRDeep2 candidates and 5.6× lower than the candidates predicted by mirnovo (Fig. 3A1). For plant species, BrumiR predicted on average ∼700 putative mature miRNAs, which was 4.7× lower than the candidates predicted by mirR-PREFeR (3,248 on average) and 5.3× higher than the predictions of mirnovo (131 on average) (Fig. 3A1). A comparison using the miRBase [22] annotated miRNAs revealed that BrumiR shared more candidates with miRDeep2 and miR-PREFeR than with mirnovo (Fig. 3A2). However, an important fraction (on average more than 70%) of the miRBase-annotated candidates was exclusive to each tool (Fig. 3A2), which summarizes the complexity of miRNA discovery.

Considering mirGeneDB for animals and miRBase-annotated candidates for plant species as the ground truth, we computed precision, recall, and F-score for all the evaluated tools (Fig. 3B, Materials and Methods section). BrumiR achieved an accuracy (F-score) better for animals and plants than the one obtained by the other software (Fig. 3B3). Moreover, BrumiR consistently reached the highest recall for most of the datasets evaluated (Fig. 3B2). The precision values of BrumiR were slightly lower for some datasets (Fig. 3B1) in comparison with methods based on a reference genome such as miRDeep2, which has better precision due to the fact that the predictions are more conservative than the *de novo* methods (Fig. 3B3). However, on average, BrumiR reached the highest precision (∼0.44) on animal species and also on plant species (∼0.43). For the animal benchmark, we used the mirGeneDB database [40], and for plants, we used miRBase [22]. MirGeneDB is a manually curated database that has fewer entries compared to miRBase (17,599 vs. 48,885) but has more reliable miRNA sequences. Unfortunately, the number of plant miRNA annotations hosted in mirGeneDB is not enough to use it as the ground truth. We therefore kept miRBase for plant species.

We also compared BrumiR-core to de Bruijn graph transcriptome *de novo* assemblers (Trinity and Velvet; see Materials and Methods) [41, 42] in order to assess the performance of a pure de Bruijn graph approach for miRNA discovery. We can observe that the *de novo* transcriptome assemblers generated on average 40× and 4× more candidates than BrumiR for Trinity and Velvet, respectively (Supplementary Table S7). In general, the huge number of contigs generated by the transcriptome assemblers, even after filtering them by length, was poorly matched to the miRBase entries (1.2% and 36%, respectively). On the other hand, BrumiR matched the miRBase entries at a rate of 1 of every 2 candidates (52% precision average). As expected, we can conclude that most of the contigs generated by a pure de Bruijn graph transcriptome assembler are poorly related to miRNA sequences. This was expected because they are developed for mRNAseq analysis and do not consider the complexities of the sRNA-seq data like BrumiR.

In summary, this experiment showed that BrumiR and all the downstream steps it performs after the de Bruijn graph construction are essential for miRNA discovery.

The BrumiR toolkit also provides a tool to determine the hairpin loop of miRNA precursor sequences, which is the main structural feature of miRNAs [43]. BrumiR2reference maps the BrumiR predicted mature miRNA to the reference genome using an exhaustive alignment (see Materials and Methods section), generates precursor sequences, computes its secondary structure, and checks the hairpin structure using a variety of criteria inferred from analyzing more than 30,000 miRBase precursor sequences from animal and plant species (see Materials and Methods section). We used BrumiR2reference as a double validation for all the predicted mature miRNAs generated by BrumiR for the animal and plant datasets (Fig. 3C). On average, BrumiR2reference identified a valid precursor sequence having the characteristic hairpin structure for over 60% of the BrumiR candidates (Fig. 3C).

In terms of speed, BrumiR-core was the fastest tool. BrumiR was on average 19× and 38× times faster than miRDeep2 and miR-PREFeR, respectively (see Supplementary Table S6).

Overall, we demonstrated that BrumiR is a competitive tool for discovering mature miRNAs without a reference genome. We showed that it was the most sensitive on most of the datasets tested. The performance of our method was not only faster but also better than or comparable to the state-of-the-art tools. Moreover, we also provide a new mapper approach to be used when a reference genome is available, to further verify if a precursor sequence of the predicted mature miRNA is present in the genome. BrumiR therefore represents a reliable alternative for the discovery of mature miRNAs in model and nonmodel species with or without a reference genome.

### Using a supervised machine learning approach to refine the BrumiR-core prediction

**Figure 4:**
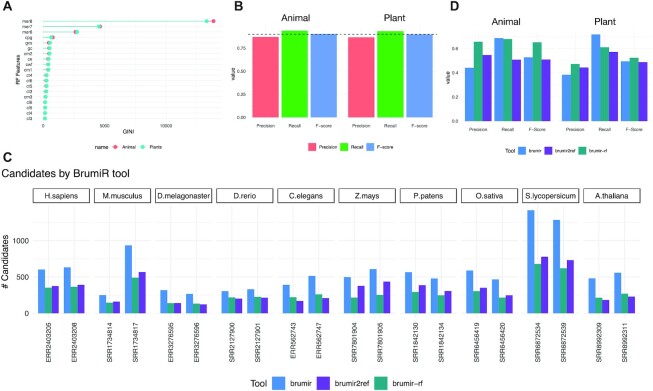
Improving the precision of BrumiR by using a random forest classifier. (A) Most informative features of the random forest classifier for animal and plant species. (B) Benchmark metrics of the random forest model to discriminate 15-mer miRNA mature sequences from random 15-mer sequences. (C) Number of candidates by BrumiR tools (BrumiR-core, BrumiR2reference, BrumiR-RF). (D) Average metrics across animal and plant real datasets for all BrumiR tools.

To further refine the prediction of BrumiR, especially in the plant datasets, we developed and implemented a supervised machine learning method based on a random forest model [44]. The random forest model classifies the BrumiR candidates into putative miRNA or random sequences. The random forest model is composed of 19 features, of which 16 are inferred directly from the 15-mer sequences of each BrumiR candidate and 3 are derived from the nucleotide composition observed on reference mature miRNA sequences. In order to use a confident input and reduce as much as possible the number of false predictions, we employed the manually curated database mirGeneDB [40] for training with animal species, while for plant species, we kept miRBase due to the low number of miRNA plant entries present on mirGeneDB. The 16 derived features are GC content (gc), GC skew content (gcs), CpG content (cpg), sequence complexity by Wootton and Federhen values (cwf), sequence Shannon entropy (ce), sequence complexity of Markov model values (cm1, cm2, cm3), sequence complexity by Trifonov values (ct3, ct4, ct5, ct6), and sequence complexity linguistic values (cl3, cl4, cl5, cl6) [45]. The nucleotide compositions are the 6-mer, 7-mer, and 8-mer observed frequency of mature miRNA sequences from the reference miRNA databases (MirGeneDB or miRBase). The features were computed on a 15-mer basis to classify any length of miRNA candidates (18–22 base pairs). A total of 35,570 15-mers were derived from the MirGeneDB, and all the 19 features were computed for each. The top 5 most informative features for discriminating miRNA from random sequences were 8-mers, 7-mers, 6-mers, CpG content, and GC content, as well as the complexity of Markov models (Fig. 4A). The benchmark results show that the random forest classifier achieves an accuracy of 90%, a precision of 87%, and a recall of 94% for discriminating animal miRNA 15-mers from random ones (Fig. 4B). The miRBase model achieves an accuracy of 90%, a precision of 87%, and a recall of 93% for discriminating plant miRNA 15-mers from random ones (Fig. 4B). We used the random forest classifier to further refine the BrumiR prediction on animal and plant real datasets. Similar to BrumiR2reference, BrumiR-RF reduced the number of BrumiR-core candidates (Fig. 4C) but without the need of a reference genome. We observe that most of the discarded candidates were likely false positives (considering the reference miRNA database is the ground truth), which results in an improved precision without affecting the recall (Fig. 4D). In summary, the BrumiR-RF classifier allowed us to increase the precision of BrumiR without affecting its overall recall and without the need of a high-quality reference genome. The BrumiR toolkit now provides tools for handling all kinds of miRNA-related information for an enhanced miRNA prediction discovery.

### Discovering novel miRNAs from sRNA-seq data of *A. thaliana* roots using BrumiR

*A. thaliana* is one of the best characterized model organisms and the first plant species in which miRNAs were cloned and sequenced [46]. To date, 436 mature miRNA sequences are included in the miRBase database. Most of these miRNAs have been identified by studies addressing the sRNAome of different plant organs [47], cell types [48], or responses to biotic or abiotic stress using sRNA-seq [49, 50].

**Figure 5:**
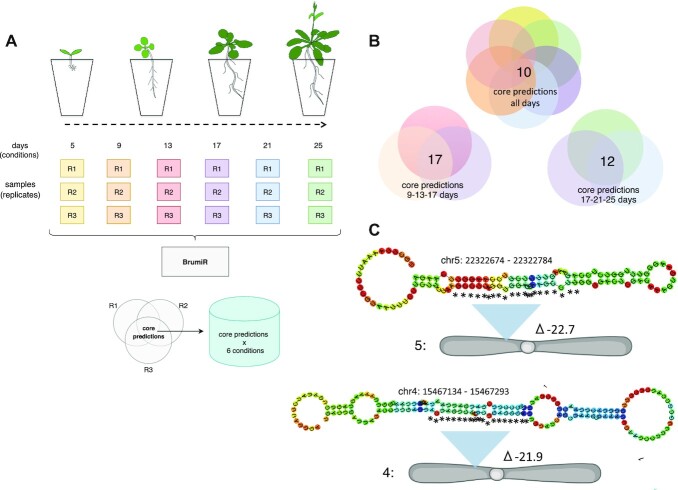
Applying BrumiR on sRNA-seq from *Arabidopsis* root libraries. (A) Experimental design implemented; roots from *Arabidopsis* on a time scale per day as conditions were sequenced in 3 technical replicates. BrumiR was used to analyze all sRNA-seq libraries, and conserved predictions by the 3 replicates were considered as a core by condition. (B) Different combinations of root growth per day were analyzed together to identify novel putative miRNAs conserved in all conditions. (C) We discovered 2 candidates as novel miRNAs that fulfill the current criteria to annotate miRNAs in plants. Moreover, they were supported directly from the sRNA-seq libraries and are conserved in all replicates in all conditions.

We sequenced sRNA-seq libraries from the roots of *A. thaliana* after different time points during vegetative development (see Materials and Methods section) (Supplementary Fig. S12) to demonstrate the potential of BrumiR to discover novel mature miRNAs in a known biological context. BrumiR was run independently for each condition and replicate. The day 5 samples were excluded because of the low number of reads when compared to the other samples (Supplementary Table S8). BrumiR predicted, on average, 1,160 mature miRNAs per sample, which were further refined to 719 using the BrumiR2ref tool. To take advantage of our experimental design, we considered as a putative miRNA the ones present in the 3 replicates (core predictions) [51] (Fig. 5A). Novel miRNAs were identified using the following steps: first, predictions were classified as known miRNAs by comparing with miRBase (141 known miRNAs out of a total of 159 miRNAs already described for *A. thaliana* in miRBase). These known miRNAs were put aside to explore the sensitivity of BrumiR in detecting novel putative miRNAs. We then clustered the remaining putative miRNAs into 3 stages: early, late, and constitutive (Fig. 5B). The days 9, 13, and 17 represent an early stage of the plant development; days 17, 21, and 25 represent a late stage of the plant development [52]; and the putative miRNAs expressed in all conditions represent the constitutive category (Supplementary Table S8). A total of 21 putative novel miRNAs were identified, and a manual curation was carried out revising all the criteria to validate and annotate miRNAs in plants [51]. We discovered 2 novel miRNAs candidates that fulfill all the recommended criteria to annotate miRNAs in plants (Supplementary Fig. S10, Supplementary Table S8). According to the revised criteria, confirmation by blot of the expression of the miRNA or miRNA* is disallowed, and it is suggested that validation of miRNA expression should be based on sRNA-seq reads only. In this way, these 2 curated novel miRNA candidates are supported directly from the sRNA-seq libraries and are expressed in all replicates in all conditions [51].

One of the curated novel miRNAs candidates (miR-8) is located in chromosome 5 (Fig. 5C); this miRNA locus has not been previously discovered because its mature sequence maps to multiple chromosomes and is therefore discarded by genome-based tools [26].

In an exploratory analysis to shed light on the potential targets of these novel miRNAs, we conducted an *in silico* target transcript prediction using the 1 algorithm [53] (Supplementary Table S10). EXO84b (AT5G49830) was found to be one of the top genes regulated by this novel miRNA miR-8 (Supplementary Table S9). In *A. thaliana*, the importance of EXO84b has been demonstrated in the development of tracheary elements or the vessel xylem system, which is essential for water and nutrient transport of vascular plants [54]. EXO84b is expressed over all days but significantly abundantly expressed in the last days, and its differential accumulation between root zones is related to emerging patterns of lateral roots and hair formation from trichomes [55].

We have also explored the known miRNAs identified by BrumiR in which we have found in almost all the samples, with a high expression, the plant miRNAs that would be playing a key role in root specification and development [56].

It is plausible to say that these novel and known miRNAs may be involved in the fine-tuning of lateral root growth in the early stages of development.

These results highlight the value of the BrumiR toolkit for discovering novel and known miRNA candidates with functional impact on the organisms studied, even in the case where high-quality genomes are available.

## Discussion

In this article, we introduced and benchmarked the BrumiR toolkit, which was designed for enabling the identification of mature miRNAs in model and nonmodel species with or without a reference genome, encompassing the plant and animal kingdoms. The BrumiR toolkit implements the following algorithms: (i) a new discovery miRNA tool (BrumiR-core), (ii) a specific genome mapper (BrumiR2ref), (iii) an sRNA-seq read simulator (miRsim), and (iv) a mature miRNA sequence classifier (BrumiR-RF). We demonstrated that BrumiR is capable of identifying mature miRNAs based only on the sequence information and generates results that are better than or comparable to the state-of-the-art tools on simulated and real datasets. We further tested the usefulness of the BrumiR toolkit for discovering novel miRNAs potentially involved in the regulation of the root development of the extensively annotated *A. thaliana* genome.

Unlike the state-of-the-art tools, BrumiR starts by encoding the sRNA-seq reads using a de Bruijn graph. This avoids the read mapping stage and the dependency on previous miRNA annotations. It also enables the identification of sequencing artifacts. A critical step of genome-based miRNA discovery tools is to identify the precursor sequence when a reference genome is available. BrumiR introduces a new mapping approach, BrumiR2reference, which scans every possible hairpin precursor in the genome, when such is available, for all the BrumiR predictions. As the hairpin structure is determined using the predicted mature miRNA instead of the reads, this alignment can support mismatches and indels and handles the case of multimapped candidates (due to repetitive regions of the genome). Such features distinguish BrumiR from the current genome-based methods.

Discovering miRNAs in nonmodel species is one of the limitations of the current methods. One exception is mirnovo, which, similar to BrumiR, can predict miRNAs using only the sRNA-seq data, and a specific training set for animal and plant species. We thus compared its performance to the one of BrumiR. Our results show that mirnovo is very conservative, generating few predictions in comparison to BrumiR. This could be due to the low number of entries of plant miRNAs in miRBase because the mirnovo approach is based on miRNA families present in this database. However, miR-PREFeR generates a larger number of candidates in plant species. The higher number of predictions of miR-PREFeR results in lower precision in most of the evaluated datasets, on which BrumiR obtained the highest F-score in 6 out of 10 cases. In animal species, BrumiR has a lower precision compared to miRDeep2 in some of the datasets, but considering the F-score, BrumiR obtains the highest rate in all the datasets. We examined possible Piwi-interacting RNA (piRNA) sequences present in the sample of *M. musculus* SRR1734817 to see if this high number of candidates was due to wrong predictions, but no relationship was found (Supplementary Table S13).

When we use the complementary tools of BrumiR, BrumiR2reference, and BrumiR-RF, BrumiR exceeds its performance, reaching the highest metric in 16 of the 20 datasets, improving the precision and reducing the number of candidates without sacrificing the recall rates.

In an attempt to increase the accuracy of BrumiR, we developed the BrumiR2reference and BrumiR-RF tools, thus reducing the number of false-positive miRNAs without sacrificing recall. We implemented a supervised random forest classifier trained on the high-confidence mature sequences available in the manually curated database mirGeneDB. The latter led to an important improvement in the accuracy of BrumiR, even in the case when a reference genome is not available. It is important to observe that the miRNA annotations remain incomplete, and although miRBase is the main repository for miRNAs, it cannot be considered the gold standard for most species (many of the entries have not been correctly validated, for example) [18]. For this reason, we used mirGeneDB, but the predictions of BrumiR are not based on miRBase or on mirGeneDB in any step of the algorithm. These tools can be used in a posterior analysis to verify the miRNAs inferred in case of not having any reference genome as a postprediction step in a complementary way.

In terms of computational resources and usability, BrumiR is the fastest method and provides a stand-alone package for running locally all the analyses. It further generates an output that is compatible with the bandage software [37], which can be employed to visualize and explore the results of BrumiR in a user-friendly way.

Moreover, BrumiR reports other sequences expressed in the sRNA-seq data, among which are putative isomiRs and longer noncoding RNAs, thereby providing additional biological insight.

Finally, we tested the effectiveness of BrumiR on sequenced sRNA-seq libraries from the roots of *A. thaliana*, and were able to discover 2 novel putative miRNAs based on the very conservative criteria proposed in [51], showing the potential of it being used alone or in combination with other methods.

In summary, we present a new and versatile method that implements novel algorithmic ideas for the study of miRNAs that complements and extends the currently existing approaches.

## Materials and Methods

### Building a de Bruijn graph for sRNA-seq data

BrumiR starts by building a compact de Bruijn graph from the sRNA-seq reads given as input. de Bruijn graphs are a widely used approach in the genome assembly problem [34]. BrumiR uses this graph to organize, detect, and exploit the sequence information of sRNA-seq experiments. BrumiR takes as input sequencing files in FASTA or FASTQ formats. The sequencing data can be cleaned, using a fastq preprocessor [39] (i.e., fastp), to remove adapter sequences and trim low-quality bases. BrumiR employs the BCALM [35] tool to build a de Bruijn graph from the sRNA-seq reads. BCALM uses a node-centric bidirected de Bruijn graph where the nodes are *k*-mers, that is, words of length *k*, and an arc between 2 nodes if the *k*−1 suffix of one node is equal to the *k*−1 prefix of the subsequent node, representing an exact overlap of *k*−1 bases [35]. A critical parameter of any de Bruijn graph approach is the *k*-mer size [57]. We observed that the length of all mature miRNA sequences stored in the miRBase database (v21) [22] fluctuates between 18 and 24 nt (Supplementary Fig. S1). To determine the optimal *k*-mer size for BrumiR, we compared the performance of BrumiR using different *k*-mer sizes (14, 16, 18, 20, 22*k-*mers). The benchmark shows that the optimal *k*-mer size for BrumiR is 14 (Supplementary Table S1, Supplementary Fig. S2), because it allows for a better handling of the sequencing errors and enables a more sensitive clustering of identical miRNA candidates, even when comparing with 18-mers, which is the *k*-mer size expected for a mature miRNA sequence (Supplementary Fig. S3). We thus empirically set the *k*-mer size equal to 14. BCALM compacts the nodes of the de Bruijn graph into maximal unipaths by gluing all the nodes of the graph with an in-degree and an out-degree equal to 1, thus generating the so-called *unipath graph* [35]. The unipath graph is the starting point of BrumiR (Figure 1A). Notice that the unipath graph generated by BCALM does not represent what is expected for a set of mature miRNAs (one connected component for each miRNA), and therefore further graph operations are needed. BrumiR uses a minimum *k*-mer frequency (KM value) of 5, and all *k*-mers with lower frequency are ignored, without losing most of the information contained in the sequencing reads (Supplementary Fig. S4). Additionally, we ran BrumiR-core using different depth coverages in order to define the optimal coverage resolution for the resulting de Bruijn graph (Supplementary Table S8). The comparison shows a convergence in the number of candidates at a depth coverage of 50, and because of this, we set this parameter at this value (Supplementary Fig. S5).

### Removing sequencing errors from the unipath sRNA-seq graph

BrumiR deletes from the unipath graph all the nodes that have only 1 connection (degree equal to 1), known as dead-end paths or tips [58]. Usually, these nodes have a low abundance value associated to them (KM less than or equal to 5, the default parameter). Moreover, BrumiR deletes isolated nodes (degree equal to 0) having a low abundance; isolated nodes highly expressed are, however, conserved for further analysis. All these nodes are likely artifacts generated from sequencing errors because they are not deeply expressed in the sRNAs-seq reads [59]. BrumiR iterates this step 3 times in order to prune and clean the unipath graph (polishing). This operation, called “tip removal,” edits the original unipath graph, and therefore a new unipath graph with a new structure is generated (Fig. 1B).

### An expressed mature miRNA has uniform coverage

The unipath graph of a set of miRNAs from an sRNA-seq experiment has nonuniform coverage as different miRNAs and other elements may be connected in a single big component (Fig. 1). BrumiR evaluates each connection of the unipath graph to identify those that link 2 nodes with a large expression difference. According to the miRNA biogenesis, after a stable miRNA precursor is cleaved by Dicer, among its 3 products, the miRNA mature sequence is the most abundant, and when it is sequenced, it has a uniform expression along its sequence [29]. Thus, due to miRNA biogenesis, it is possible to capture the complete miRNA mature sequence having a homogeneous expression [30] directly from the sRNA-seq experiments. BrumiR expects a similar KM value for *k*-mers originating from the same mature miRNA gene. Accordingly, if we observe 2 connected nodes that show a big difference in their abundance values, this connection is deleted and we keep the nodes unconnected. In particular, 2 unipaths *U = {a,b}* connected in the graph have a KM value associated to them that represents their coverage from the reads information. BrumiR scans all the neighbor connections, and if the difference between their KMs is larger than 3-fold, the connection is deleted (Ui_km_/Uj_km_ > 3). In this way, BrumiR defines a relative threshold that will depend on each unipath neighborhood in the graph. Finally, BrumiR repeats the tips removal step to eliminate new low-frequency isolated nodes (Fig. 1C).

### miRNAs and other sequences are captured in single connected components

After the previous steps of BrumiR, a new unipath graph emerges, with a new structure. It is thus necessary to identify and classify the new connected elements within the graph (Fig. 1). A connected component (CC) of a graph is a maximal strongly connected subgraph [60]. BrumiR computes the CCs of the unipath graph, and then each CC is processed independently to identify miRNA candidates as well as to discard other sequences present in the unipath graph.

### BrumiR classifies low-abundance nonlinear topologies as sequencing artifacts

BrumiR detects topologies that are potentially related to sequencing errors and thus unlikely to be miRNA candidates. The shapes of these topologies were identified by visual inspection of several unipath graphs and are described in detail in Supplementary Fig. S6. Usually, they have low KM and are composed of lowly expressed branching nodes with 3, 4, or 5 connections to the principal structures in the graph (Supplementary Fig. S6). Moreover, we observed that the sequences contained in these topologies were usually redundant and contained in other linear and more expressed CCs. In this way, we are not discarding relevant sequence information. BrumiR removes about 10% of the CCs in this step.

### Reassembling unipaths within each CC

BrumiR reassembles all unipaths present in the linear CCs by bundling the nodes with an in and out degree equal to 1 into a new unipath. BrumiR classifies them into different types based on their length. The latter is the length of the sequence represented by the new unipath. All CCs having a length between 18 and 24 are stored as potential miRNA sequences. The CCs corresponding to an isolated node that have high KM (KM >50) are included in the latter group. CCs with lengths over 24 are classified as longer sequences or other types of genomic sequences captured along with the miRNAs. The longer sequences are put aside for later analysis. Moreover, BrumiR identifies circular CCs and branching CCs. The former are circular unipaths and the latter CCs with a high number of branching nodes. Branching CCs are not considered in the subsequent steps because they are likely sequencing errors (low abundance) or contamination present in the sRNA-seq data (Supplementary Fig. S7).

### Reclustering potential miRNAs

After grouping unipaths by CCs, BrumiR builds an overlap graph to rescue the missing connections between potential miRNA candidates sharing an overlap with another candidate. First, BrumiR adds all the candidates as nodes of the overlap graph, and then an all-versus-all *k*-mer comparison is performed using exact overlaps of length *k* = 15. Candidates sharing an exact overlap are connected in the overlap graph. Then, the connected components are computed to identify clusters of miRNA candidates, and the most expressed candidate within each component is selected as the representative candidate of the cluster. The representative candidates are compared all-versus-all in a second overlap step that allows a maximum edit distance of 2, which is implemented using the edlib library [61]. BrumiR then builds a second overlap graph, computes again the connected components, and selects the most expressed candidate as the representative of each cluster. The other members of each connected component are classified as putative isomiRs and saved in a file for later analysis.

### Identifying other expressed RNA sequences

In sRNA-seq experiments, different types of RNAs are expressed, some of which, such as small noncoding RNA elements, may have a similar length to miRNAs [62]. The RFAM database [38] is a collection of curated RNA families including 3 functional classes of RNAs (noncoding, *cis*-regulatory elements, and self-splicing RNAs), which are classified into families according to their secondary structure and sequence information (covariance models) [36]. We downloaded 3017 RNA families present in RFAM (v14.1) and excluded 529 miRNA families. The sequences of 2,488 RFAM families were concatenated (a total of 2,736,549 sequences) and used to build a 16-mer database with the KMC3 *k*-mer counter tool [63] (“-fm -n100 -k16 -ci5”). All distinct 16-mers with a frequency lower than 5 were excluded, leading to a total of 6,204,556 distinct 16-mers related to RNA elements. Additionally, we downloaded all the mature miRNA sequences from miRBase (v22.1) [22] and built a 16-mer database with KMC3 (“-fm -n100 -k16 -ci1 mature.fa.gz”). RFAM 16-mers matching 16-mers from the 16-mer mature miRBase database were excluded from RFAM, leading to a 16-mer RFAM database with a total of 6,204,487 distinct 16-mers. Finally, the BrumiR candidates (18–24 length) were matched to the 16-mer RFAM database, and matching candidates were excluded and reported as sequences potentially associated with other RNA elements. The BrumiR candidates passing the aforementioned filter are reported as the final list of miRNA candidates.

### Identifying precursor sequences for BrumiR candidates (BrumiR2reference)

Unlike current state-of-the-art tools that perform miRNA discovery by mapping the sRNA-seq reads to a reference genome, BrumiR generates candidates by operating directly on the sRNA-seq reads. The reduced list of potential BrumiR miRNA candidates permits the computation of a more exhaustive alignment than when mapping directly the sRNA-seq reads to the reference genome. BrumiR aligns each candidate to the reference genome using an exact alignment method that computes the edit distance [14] between 2 strings and thus supports mismatches, insertions, and deletions. The BrumiR2reference tool divides the reference genome in nonoverlapping windows of 200 bp (adjustable parameter), and then the window is indexed using 12-mers and each miRNA candidate is matched in both strands (split at 12-mers). When a 12-mer match is found, an exhaustive alignment is computed between the window and the matching miRNA candidate. The alignment is performed using a fast implementation of Myers’s bit-vector algorithm [61].

An miRNA candidate is stored as a hit if the alignment in the current genomic window has an edit distance less than or equal to 2. After scanning all the genomic windows, the vector of hits is sorted by miRNA candidate, edit distance (0–2), and alignment sequence coverage. For a single miRNA candidate, a maximum of 100 genomic locations (best hits) are selected. BrumiR2reference then builds a potential precursor sequence for each selected hit using a strategy similar to the ones employed by miRDeep2 [30] and Mirinho [64]. BrumiR excises the potential precursor hairpin sequence from the flanking genomic coordinates of the reported miRNA candidate hits (mature sequence) in both strands. Potential precursor hairpin sequences of length 110 bp are built for animal species from both strands, while for plant species, hairpin sequences of lengths 110, 150, 200, 250, and 300 bp are built from both strands [14]. Secondary structure prediction for all the potential precursor sequences is performed using RNAfold (v2.4.9) [65]. Secondary structures with a minimum free energy in the range of 15 to 80 kcal/mol are checked for a hairpin loop characteristic of miRNAs [43] (Supplementary Fig. S8). Structures with a hairpin loop composed of a single segment without pseudo-knot, multiloops, external loops, and with less than 5 bulges, 3 dangling ends, and 10 internal loops are classified as characteristic secondary structures of miRNA precursor sequences. The aforementioned filters were derived from analyzing the secondary structure of 38,589 precursor sequences stored in miRBase (v22.1) [22] using a modified version of the bpRNA program [66] (Supplementary Fig. S9).

### Benchmarking BrumiR against transcriptome de Bruijn graph assemblers

In order to determine the value of BrumiR for extracting miRNA candidates directly from a de Bruijn graph, we compared the BrumiR approach against 2 de Bruijn graph transcriptome *de novo* assemblers, namely, Trinity [41] and Velvet [42]. The benchmark was performed using 4 real datasets, including human and *Arabidopsis*. The seed length and minimum contig length for the transcriptome assemblers were fixed at 14-mers for all tools. Then, the contigs longer than 24 nt were filtered out for Trinity and Velvet. For BrumiR, we eliminated the last step using the RFAM database [36] information to filter out other kinds of sRNA sequences, and we used all the predictions to compare to the transcriptome *de novo* assemblers. Finally, the BrumiR candidates and contigs generated by Trinity and Velvet were mapped against the miRBase database (Blast search).

### Benchmarking BrumiR using simulated sRNA-seq reads

We simulated synthetic reads from animal and plant species and compared the results of BrumiR to those obtained with the miRDeep2 [30] and miR-PREFeR [31] tools. The sRNA-seq reads were simulated using miRsim (https://github.com/camoragaq/miRsim), a tool that we developed specifically for simulating sRNA-seq reads from a list of known miRNA mature sequences. miRsim is based on *wgsim* (https://github.com/lh3/wgsim), which is a widely used tool for simulating short Illumina genomic reads. miRsim includes functionalities specific of sRNA-seq reads such as variable depth/coverage and shorter read lengths. miRNA mature sequences were obtained from miRBase [22] for animal (high confidence) and plant species. Additionally, to simulate the typical fragments contained in real sRNA-seq data, we included sequences from the RFAM database (v14.1) [36] and random genomic sequences from the genomes for each of the species included in the benchmark (10% of the sequences for RFAM and genomic sequences, respectively). The animal species that we considered were *H. sapiens,M. musculus, D. melanogaster,Danio rerio*, and *C. elegans*, while the following plant species were included: *A. thaliana, Oryza sativa,Physcomitrella patens,Zea mays*, and *Solanum lycopersicum*. Supplementary Table S2 provides further details (i.e., number of reads, number of mature miRNAs, etc.) for each simulated dataset. MiRDeep2 was run on the animal datasets with the default parameters and using the score suggested by the developers, providing the respective reference genome. Similarly, miR-PREFeR was run with the default parameters on the plant datasets. BrumiR was run with the default parameters on both the animal and plant datasets. The miRNA annotations were not included for the genome-based tools in order to make a fairer comparison with BrumiR, which does not use this information. The list of simulated miRNAs was considered as the ground truth, and precision, recall, and F-score quality metrics were computed to assess the performance of each discovery tool. The benchmark metrics were defined as follows:

\begin{equation*} Recall = \frac{{TP}}{{TP + FN}}
\end{equation*}

 

\begin{equation*} Precision = \frac{{TP}}{{TP + FP}}
\end{equation*}

 

\begin{equation*} F - score = 2*\frac{{(Recall*Precision)}}{{(Recall + Precision)}}
\end{equation*}

where

TP = true-positive elements predicted as miRNAs present in the miRBase input list.
FP = false-positive elements predicted as miRNAs but not present in the miRBase input list.
FN = false-negative elements not predicted as miRNAs but that were present in the miRBase input list.

### Benchmarking BrumiR using real sRNA-seq reads

We downloaded publicly available sRNA-seq data for the plant and animal species listed in the synthetic benchmark, and 2 datasets for each species were included (Supplementary Table S4). We wanted to benchmark BrumiR in a simple but exhaustive way by selecting the top performer for genome-based and genome-free methods. We benchmarked some of the most used prediction tools in a reduced version of the real dataset, and the results were conclusive to select the best methods (Supplementary Table S5, Supplementary Fig. S10). We included mirnovo [23], a tool that can discover miRNAs without a reference genome. The predictions of BrumiR were benchmarked along with MiRDeep2 (v2.0.1.2) [30] and mirnovo for the animal datasets. Similarly, miR-PREFeR [31] replaced MiRDeep2 for the plant datasets (Supplementary Table S4).

The stand-alone packages of BrumiR, miRDeep2, and miR-PREFeR were used to discover miRNAs in all datasets. The software mirnovo was run using its web version because the stand-alone package was not available and the developer recommends the use of the web version instead. The miRNA discovery was performed for each sample independently using default parameters for MiRDeep2, miR-PREFeR, and mirnovo. In particular, we used the scripts provided by miRDeep2 and miR-PREFeR to map the reads to the reference genome, and the predictions for these tools were performed on the resulting alignment files. The mirnovo predictions were done using the animal and plant universal panel, respectively, as recommended when the reference genome is not available. BrumiR was run using the command line and parameters provided in the Supplementary Section 1. Moreover, the predictions of BrumiR were refined using the BrumiR2reference tool on the available reference genome of the selected species (Supplementary Table S4). Benchmark metrics (precision, recall, and F-score) were computed as before but considering all the annotated mature sequences present in mirGeneDB for animal and miRBase (v22.1) for plant species as the ground truth.

### A random forest model to refine the predictions of BrumiR-core

The random forest model is composed of 19 features, of which 16 are inferred directly from 15-mer sequences of each BrumiR candidate and 3 derived from the nucleotide composition observed on reference mature miRNA sequences (miRGeneDB and miRbase) [22, 40]. The nucleotide composition was analyzed using the 6-mer, 7-mer, and 8-mer observed frequency of the mature miRNA sequences of reference miRNA databases (MirGeneDB and miRbase). The features are computed on a 15-mer basis to classify any length of miRNA candidates (18–22 base pairs). A total of 35,570 15-mers were derived from the MiRGeneDB, and all 19 features were computed for each. A matching amount of 15-mer random sequences was generated, and all 19 features were computed for each. The whole training and evaluation dataset comprised 71.140 15-mers of the 2 classes (random and mature miRNA sequences). The training and evaluation of the random forest were performed using 75% and 25%, respectively. The performance of this classifier on all the real datasets is reported in Supplementary Table S9. Finally, a Rnotebook including all the steps required to build the random forest model is available at the BrumiR GitHub repository here: https://github.com/camoragaq/BrumiR/tree/master/brumir-rf.

### miRNA discovery from *Arabidopsis* root samples

*A. thaliana* Col-0 seedlings were grown hydroponically on Phytatrays on 0.5× Murashige and Skoog medium (cat. M519; Phytotechnology Laboratories, 14610 W 106th St, Lenexa, KS 66215, Estados Unidos) under long-day conditions (16 hours of light and 8 hours of dark) at 22°C. Total RNA was isolated from plant roots after 5, 9, 13, 17, 21, and 25 days postgermination using the mirVana miRNA Isolation Kit (cat. AM1560; Thermo Fisher Scientific, Waltham, Massachusetts, Estados Unidos). RNA concentration was determined using the Qubit RNA BR Assay Kit (cat. Q10210; Thermo Fisher Scientific), and integrity was verified by capillary electrophoresis on a Fragment Analyzer^TM^ (Advanced Analytical Technologies, Inc., 37 Ramland Rd, Orangeburg, NY 10962, Estados Unidos). The indexed sRNA libraries were built employing the TruSeq small RNA Sample Preparation Kit (Illumina, Inc., San Diego, CA 92122, USA) following the manufacturer's instructions. Briefly, 3′ and 5′ adaptors were sequentially ligated to 1 μg total RNA prior to reverse transcription and library amplification by PCR. Size selection of the sRNA libraries was performed on 6% Novex TBE PAGE Gels (cat. EC6265BOX; Thermo Fisher Scientific) and purified by ethanol precipitation. Both the library size assessment and library quantification were carried out in a Fragment Analyzer^TM^. Finally, the libraries were pooled and sequenced on an Illumina NextSeq 500 platform (Supplementary Fig. S12).

All samples were analyzed with BrumiR separately with default parameters to identify the candidate miRNAs. We further validated the candidates having a putative precursor with a hairpin structure analysis using the BrumiR2ref tool with the reference genome for *A. thaliana* (GCF_000001735.4_TAIR10.1_genomic.fna). All validated candidate miRNAs were compared to known miRNAs described for *A. thaliana* (437) present in miRBase (v21) (Supplementary Table S9). We used the current criteria to validate and annotate miRNAs in plants that are based on experimental evidence coming directly from the sequencing libraries, as shown in Axtell 2018 [51]. We conserved the candidates predicted in all the replicates (as described in Fig. 4), and the putative novel miRNAs were manually curated. Specifically (Supplementary Table S11), we checked the criteria related to precursor length, hairpin structure, and miRNA length in at least 2 sRNA-seq libraries (biological replicates) (Supplementary Fig. S11) [18]. Then a target analysis was performed using the Araport 11 complementary DNA library with the plant‐specific psRNATarget algorithm (based on a best expectation score) (Supplementary Table S12) [53].

## Availability of Supporting Source Code and Requirements

Project name: BrumiR

Project home page: https://github.com/camoragaq/BrumiR

Operating system(s): Unix, Linux, and Mac OSX

Programming language: C++, PERL, and R

Other requirements: Compilation was tested with g++ using -std = c++11 for Linux and Mac OSX system

RRID:SCR_022727

License: MIT

Any restrictions to use by nonacademics: none

## Availability of Supporting Data

Snapshots of our code and other data further supporting this work are openly available in the *GigaScience* repository, GigaDB [67].

## Additional Files

**Supplementary Fig. S1**. Length distribution of mature sequences in miRBase. We used miRBase v21 with a total of 35,828 entries and we observed that the length is between 19 and 24 nt.

**Supplementary Fig. S2**. Determination of the optimal BrumiR *k*-mer size. (A) Number of candidates predicted and matching mriBase entries as a function of the *k*-mer size for an animal and plant dataset. (B) Benchmark metric computed for each *k*-mer size evaluated. The best performance is obtained at *k*-mer = 14.

**Supplementary Fig. S3**. Benchmark metric comparison for animals and plants using different miRNA discovery tools and 2 BrumiR *k*-mer sizes (14-mers and 18-mers). The top and bottom plots shown the average of precision, recall, and F-score across the animal and plant datasets for BrumiR (*k* = 18 and *k* = 14), miRdeeep2/Mir-PREFER, and mirnovo. The best performance for BrumiR is achieved when using a *k*-mer size of 14.

**Supplementary Fig. S4**. The *k*-mer spectrum of sRNA-seq data. (A) The histogram shows the number of distinct *k*-mers (y-axis) as a function of the read coverage (KC x-axis). In the lower coverage of the spectrum (black rectangle), we observe a high number of distinct *k*-mers, which are likely sequencing errors. The *k*-mers that correspond to noise represent approximately less than 15% of the total number of *k*-mers.

**Supplementary Fig. S5**. Comparing the performance of BrumiR using different depth coverage values.

**Supplementary Fig. S6**. BrumiR classifies low-abundance nonlinear topologies as sequencing errors. (A) BrumiR identifies these topologies connected to the principal structures in the graph, which appear after the first tip removal steps of BrumiR. (B) These topologies have low abundance (KM value) and are composed of branching nodes with 3, 4, or 5 connections.

**Supplementary Fig. S7**. Reassembling unipaths within each CC. BrumiR reassembles all unipaths present in a linear CC by bundling the nodes with an in and out degree equal to 1 into a new unipath. BrumiR rebuilds each unipath within a CC and classifies them into different types. (A) Circular CCs: when all unipaths are have an in and out connection, we classify the CC as a circular sequence that is not a putative miRNA. (B) Branching CCs: when we detect a CC with a high number of branching nodes, we do not consider it anymore for the moment, because we consider it related to sequencing errors (usually they have a low KM value). (C) Long CCs: when we detect more than 10 unipaths, we can classify them as longer noncoding sequences, but we still keep them for later analysis. (D) Potential miRNAs: all assembled unipaths (CCs) having a length between 18 and 24 are stored as potential miRNA sequences.

**Supplementary Fig. S8**. Workflow of the BrumiR2Reference tool. The main steps involved the mapping of the miRNA candidates to the genome using nonoverlapping windows (1); each hit is further refined using an exhaustive alignment (2). For each hit, a precursor sequence is built (3), and its secondary structure is determined using RNAfold (4). Finally, structures fulfilling a set of criteria (5) are classified as precursor sequences.

**Supplementary Fig. S9**. Structure properties of miRBase precursor sequences. (A) Free-energy distribution of 38,589 precursor sequences folded with RNAfold. (B) Different types of RNA secondary structure elements composing precursor miRNA sequences. (C) Analysis of secondary structure elements performed on 38,589 precursor sequences in miRBase using the bpRNA package. (D) Examples of precursor sequences for animal and plant species.

**Supplementary Fig. S10**. F-score distribution of reduced benchmark including 5 miRNA discovery tools.

**Supplementary Fig. S11**. Novel miRNAs candidates discovered by BrumiR using *Arabidopsis thaliana* root experiments that fulfill all the criteria to validate and annotate miRNAs in plants (Axtell Meyers, 2018).

**Supplementary Fig. S12**. Visualization with Bandage. BrumiR provides an output compatible with the Bandage software, which can be employed to visualize and explore the results in a user-friendly way.

**Supplementary Fig. S13**. Experimental procedure of the *Arabidopsis thaliana* roots sampling and the sRNA-seq library construction. The seedlings for each sampling point are excised from their aerial shoots, and the roots are pooled and stored in triplicates for RNA isolation. The RNAs are evaluated for concentration and integrity, above 200 ng/µL, and an RNA quality number (RQN) score over 8.0, respectively, to begin sRNA-seq library construction. The 145- to 160-bp library was purified from polyacrylamide gels and validated as a unique fragment between 145 and 160 bp. Finally, the successful libraries were processed for next-generation sequencing (NGS) procedures. Representative AATI Fragment Analyzer electropherograms are shown for RNA integrity and sequencing library validation. LM, a lower marker at 20 nt and 35 bp; UM, an upper marker at 4,000 nt and 6,000 bp. Polyacrylamide gel electrophoresis (PAGE) of reverse-transcribed complementary DNAs from small RNAs are shown. Red lines indicate fragments of interest (including sRNAs and miRNAs). Extraction procedures for fragments from PAGE are described in the library construction procedures by the manufacturer. *Figure partially created with BioRender.com.

**Supplementary Table S1**. Different *k*-mer seed length to evaluate the performance of BrumiR.

**Supplementary Table S2**. Simulated sRNA-seq used to evaluate the performance of BrumiR.

**Supplementary Table S3**. Total elapsed time per tool (seconds) on synthetic datasets. The total elapsed time reported includes only the core step of each algorithm.

**Supplementary Table S4**. Real sRNA-seq data used to evaluate the performance of BrumiR.

**Supplementary Table S5**. Reduced benchmark 5 miRNA discovery tools.

**Supplementary Table S6**. Total elapsed time per tool (seconds) on real datasets. The total elapsed time reported includes only the core step of each algorithm.

**Supplementary Table S7**. RNA transcriptome assemblers comparison.

**Supplementary Table S8**. Number of candidates per depth coverage.

**Supplementary Table S9**. Random forest classifier benchmark to evaluate the performance BrumiR.

**Supplementary Table S10**. miRNA discovery from the root samples of *Arabidopsis thaliana* using BrumiR.

**Supplementary Table S11**. Novel miRNAs in the root samples of *Arabidopsis thaliana* predicted by BrumiR.

**Supplementary Table S12**. Novel microRNAs and their putative interactions obtained using psRNATarget. miRNA Acc., microRNA identification; Target Acc., mRNA target identification, linked to the *Arabidopsis thaliana* messenger RNA (mRNA) library with the Araport V11 genome annotation. Expectation: mismatches penalty between mature small RNA and the target sequence; the lower the value, the better the prediction (with 5.0 as a maximum threshold). Inhibition: refers to the possible mechanisms used by the sRNA to regulate its mRNA target, described in plants. Target Desc: refers to the gene description for the mRNA target, found in the Araport V11 annotation. Multiplicity: indicates how many times a sRNA has a target sequence in a unique mRNA.

**Supplementary Table S13**. piRNAs-db total entries:54,865 match entries:345.

giac093_GIGA-D-20-00262_Original_Submission

giac093_GIGA-D-20-00262_Revision_1

giac093_GIGA-D-20-00262_Revision_2

giac093_GIGA-D-20-00262_Revision_3

giac093_Response_to_Reviewer_Comments_Original_Submission

giac093_Response_to_Reviewer_Comments_Revision_1

giac093_Response_to_Reviewer_Comments_Revision_2

giac093_Reviewer_1_Report_Original_Submission Dadi Gao -- 9/20/2020 Reviewed

giac093_Reviewer_1_Report_Revision_1 Dadi Gao -- 12/3/2021 Reviewed

giac093_Reviewer_2_Report_Original_Submission Marc Friedlander -- 9/25/2020 Reviewed

giac093_Reviewer_3_Report_Original_Submission Ernesto Picardi -- 10/1/2020 Reviewed

giac093_Supplemental_File

## Abbreviations

CC: connected component; CE: sequence Shannon entropy; CPG: cytosine and guanine separated by only 1 phosphate group; CPU: central processing unit; CWF: sequence complexity by Wootton and Federhen values; ENA: European Nucleotide Archive; GC: guanine cytosine; GFA: graphical fragment assembly; KM: *k*-mer mean abundance; NGS: next-generation sequencing; SRA: Sequence Read Archive.

## Competing Interests

The authors declare that they have no competing interests.

## Funding

This work was supported by CONICYT BECAS CHILE DOCTORADO 2016/FOLIO 72170320 granted to C.M., by a postdoctorate fellowship from the Agence National de Recherche (ANR-GREEN 17_CE20_0031_01) granted to M.G.F., and by Fondo Nacional de Desarrollo Científico y Tecnológico (FONDECYT)-ANID grants 1170926 and 1211130, ANID PCI-Redes Internacionales entre Centros de Investigación grant REDES180097, ANID—Millennium Science Initiative Program—ICN17_022, and ANID/ACT210007 to E.A.V.

## Authors' Contributions

C.M. designed, developed, implemented, and benchmarked BrumiR. M.F.S. guided the development of BrumiR. E.S. conducted the *A. thaliana* experiments. E.A.V. designed and supervised the *A. thaliana* experiments. C.M. wrote the initial version of the manuscript with inputs from all other authors. M.F.S. and M.G.F. helped to improve the manuscript. E.A.V., M.F.S., M.G.F., and R.A.G. provided crucial biological feedback. All authors provided helpful discussions for the work and reviewed the manuscript.
