# Supplementary material for: BrumiR: A toolkit for de novo discovery of microRNAs from sRNA-seq data
Source: Gigascience. 2022 Oct 25;11:giac093. doi: 10.1093/gigascience/giac093 (PMC9596168; doi:10.1093/gigascience/giac093)
Supplement: giac093_GIGA-D-20-00262_Revision_1 [file giac093_giga-d-20-00262_revision_1.pdf]

GigaScience

BrumiR: A toolkit for de novo discovery of microRNAs from sRNA-seq data.

--Manuscript Draft--

**Manuscript Number:**	GIGA-D-20-00262R1
**Full Title:**	BrumiR: A toolkit for de novo discovery of microRNAs from sRNA-seq data.
**Article Type:**	Technical Note
**Funding Information:**	becas chile DOCTORADO - ANID (72170320) — Dr. Carol Moraga<br>Agence Nationale de la Recherche (ANRGREEN 17_CE20_0031_01) — Dr Mariana Galvao Ferrarini<br>fondecyt Chile (1170926) — Dr Elena A Vidal<br>ANID redes internacionales (REDES180097) — Dr Elena A Vidal
**Abstract:**	Background: MicroRNAs (miRNAs) are small non-coding RNAs that are key players in the regulation of gene expression. In the last decade, with the increasing accessibility of high-throughput sequencing technologies, different methods have been developed to identify miRNAs, most of which rely on pre-existing reference genomes. However, when a reference genome is absent or is not of high quality, such identification becomes more difficult. Results: In this context, we developed BrumiR, an algorithm that is able to discover miRNAs directly and exclusively from sRNA-seq data. We benchmarked BrumiR with datasets encompassing animal and plant species using real and simulated sRNA-seq experiments. The results demonstrate that BrumiR reaches the highest recall for miRNA discovery, while at the same time being much faster and more efficient than the state-of-the-art tools evaluated. The latter allows BrumiR to analyze a large number of sRNA-seq experiments, from plants or animals species. Moreover, BrumiR detects additional information regarding other expressed sequences (sRNAs, isomiRs, etc.), thus maximizing the biological insight gained from sRNA-seq experiments. Finally, when a reference genome is available, BrumiR provides a new mapping tool (BrumiR2reference) that performs an a posteriori exhaustive search to identify the precursor sequences. Conclusions: In summary, we present a new and versatile method that implements novel algorithmic ideas for the study of miRNAs that complements and extends the currently existing approaches. The code of BrumiR is freely available at https://github.com/camoragaq/BrumiR .
**Corresponding Author:**	Carol Moraga<br>Université Claude Bernard Lyon 1: Universite Claude Bernard Lyon 1<br>Villeurbanne, FRANCE
**Corresponding Author Secondary Information:**	
**Corresponding Author's Institution:**	Université Claude Bernard Lyon 1: Universite Claude Bernard Lyon 1
**Corresponding Author's Secondary Institution:**	
**First Author:**	Carol Moraga
**First Author Secondary Information:**	
**Order of Authors:**	Carol Moraga<br>Evelyn Sanchez<br>Mariana Galvao Ferrarini<br>Rodrigo A Gutierrez<br>Elena A Vidal<br>Marie-France Sagot
**Order of Authors Secondary Information:**	

Powered by Editorial Manager® and ProduXion Manager® from Aries Systems Corporation

Response to Reviewers:	5th November 2021<br>Dear Dr Nicole Nogoy,<br>Thank you very much for considering our manuscript for publication in GigaScience. We would like to thank the referees and you for the careful assessment of our manuscript. We have attempted to address all points raised by the referees and hope that the responses are satisfactory. With these revisions, we believe that our manuscript has been substantially improved and hope that it is now suitable for publication in GigaScience.<br>Please find below our point-by-point replies to the reviewers' comments. All changes in the main manuscript and the supplement have been marked in blue font, including all the Supplementary material. We refer to the changes we have made in the manuscript using consecutive line numbering.<br>Yours sincerely,<br>Carol Moraga<br><br>Reviewer #1: Summary:<br>The authors developed a de novo assembly method, BrumiR, for small RNA sequencing data based on de Bruijin graph algorithm. This tool displayed a relatively high sensitivity in finding miRNAs and helped the authors discover a novel miRNA in A. thaliana roots.<br>Major comments:<br>1. Have the authors compared the performance with different seed lengths? Even if the minimal miR length is 18nt in MiRBase 21, seed=18 might not necessarily lead to the best AUC or F score (This might also be related to Comment 4).<br><br>We followed the reviewer recommendations and we now compare the performance of BrumiR using different seed lengths of 14, 16, 18, 20, and 22; in both animal and plant datasets. The benchmark results are reported in Table S1. The benchmark shows that the optimal seed size for BrumiR is indeed 14 and not 18 as was previously set by default. The main reason for this improved performance is that shorter seed lengths permit a better handling of sequencing errors (shorter k-mers are less likely to be sequencing errors), and enable a more sensitive clustering of identical miRNAs. Overall, we observe that the benchmark metrics (Supplementary Table S1, Figure S2) improves in comparison to the observed for the previous seed length (k=18) by 1.67X , 0.97X, 1.46X for precision, recall, and F-score in animal species, and 4.09X, 1.13X, 3X for precision, recall, and F-score in plant species, respectively. In light of the results, we have now set the default seed length to k=14, and we thank the reviewer again for this key suggestion that allows us to further improve the performance of BrumiR. Finally, the code has been updated accordingly and a new release has been published in the Github repository.<br>The updated text is in page 8, lines 13; page 25, lines 18-22.<br><br>2. The authors need to benchmark BrumiR with more existing tools (e.g. those ML-based methods), and to include more genome-free methods (e.g. MiRNAgFree).<br><br><br>In our current benchmark, we included miRDeep2, miR-PREFeR and mirnovo, and a total of 10 animal and plant datasets. miRDeep2 is the most used tool in the field with more than 1530 citations and is considered the reference miRNA prediction tool for animal species. In addition, we included miR-PREFeR that, like miRDeep2, is one of the few tools designed specifically for plant species when a reference genome is available. Complementing these popular reference-based methods, we included mirnovo, which is a genome-free discovery tool based on machine learning. The mirnovo manuscript presents a benchmark comparison with miReader and miRplex which are de novo tools and mirnovo showed better performance than the aforementioned tools. This is one of our main reasons for considering mirnovo as the best tool for performing miRNA discovery without a reference genome. Therefore, we selected the tools that perform best for miRNA discovery, including reference and de novo based.<br><br>Nevertheless, we tried to follow the reviewer's suggestion and attempted to add more tools to our benchmark. However, we had difficulties in running the existing tools because most of them are not under active development, do not provide update binaries (standalone versions), or their web-server does not work. For instance,

miRplex does not provide recent binaries (the current one, v.01, is from 2012), leading to problems with some old libraries and we could not run it. In another example, the miReader software mirPlex is not in active development (updated for the last time in 2014). We were able to run the standalone version, but after letting it run for more than one month on a single sample, we decided to stop it.

Besides these issues, we succeeded in running the miRanalyzer (genome-reference based), and miRNAgFree (de novo based) tools. The benchmark results are provided in Supplementary Table S5 using a reduced version of the real dataset. The benchmark results show that BrumiR obtained the highest F-score rates, as do miRDeep2 and mirnovo (Supplementary FigureS13). We also observed the poor performance on plant species of most of the tools, which shows the potential of BrumiR, as well as being a tool that can be used for both animal and plant species.

Currently, there is no standard for benchmarking miRNA prediction methods, which means that each new method tries to make the comparison in its own way. For this reason, we tried to make an effort to create a benchmark standard and we developed miRsim to generate benchmarks based on a ground truth known a priori and using the same conditions for all the tools.

Overall, we wanted to benchmark BrumiR in a simple but exhaustive way selecting the top performers for genome-based and genome-free methods. Finally, several miRNA tools do not provide stand-alone versions and therefore prevent extensive benchmarking when only the server is available. Being able to generate a good benchmark remains a challenge in the field of miRNAs where there are no standards.

The updated text is in page 13, lines 17; page 14, lines 1-2; page 36, lines 11-16.

3. It is also interesting to know whether de novo method for mRNA assembly would be useful on the miRNA side. It would be great if the authors were able to compare the performance of BrumiR2reference (without filtering for RFAM) with Trinity in genome-guided mode, by tweaking its seed length to be the same as BrumiR.

We followed the reviewer's recommendation and included two de Bruijn graph de novo transcriptome assemblers, namely Trinity (inchworm), and Velvet (Supplementary Table S7). The genome guide mode of Trinity first aligns the reads to the genome and then a de novo assembly is performed using the inchworm tool for each partition. The genome guide pipeline does not provide access to the seed length of inchworm (default k=25) whose default value exceeds the length of the miRNA sequences. Therefore, we ran it independently and without mapping the reads to the genome, because we thought that in this way the comparison between BrumiR and de novo transcriptome assemblers is more appropriate and allows us to focus on how the candidates are extracted from the de Bruijn graph. To perform the comparison, we used 4 real datasets; including human and Arabidopsis. The seed length and minimum contig length for the transcriptome assemblers were set to k=15 (edge-centric de Bruijn graph, overlap equals 14 nt), and l=18, for both inchworm and Velvet. Then, the contigs longer than 24 nt were filtered out for BrumiR (without filtering for RFAM) and the transcriptome de novo assemblers. Finally, the candidates were matched against miRBase (blast search) and the results of the comparison are presented in Supplementary Table S7. We can observe that the de novo transcriptome assemblers generated on average 40X and 4X more candidates than BrumiR, for Trinity and Velvet, respectively.

In general, the huge number of contigs generated by the transcriptome assemblers, even after filtering them by length, were poorly matched to miRBase entries (1,2% and 36%, indeed). On the other hand, BrumiR matched miRBase entries at a rate of 1 every 2 candidates (52% precision average). As expected, we can conclude that most of the contigs generated by a pure de Bruijn graph transcriptome assembler are poorly related to miRNA sequences. This was expected because they are developed for mRNAseq analysis and do not consider the complexities of the sRNA seq data like BrumiR.
In summary, this experiment showed that BrumiR and all the downstream steps it performs after the De Bruijn graph construction are essential for miRNA discovery.

	The updated text is in page 15, lines 9-22; page 16, lines 1-3; page 33, lines 14-22; page 34, lines 1-5.<br><br>4. The tool's sensitivity is promising across animal and plant datasets. However, the average precision is quite low, an average precision of 0.3 means a false discovery rate of 0.7. This is not an accepted value for a tool designed to discover novel miRNA. Is there any parameter the author could tweak towards a better performance? For example, is the seed length of 18nt too short to start with? Are there any other sequence features the authors should take into account to boost the performance? Or maybe some post-assembly filtering approaches might be sufficient and helpful.<br><br>We followed the reviewer's recommendations and we were able to improve the precision of BrumiR while maintaining a similar sensitivity. The major gain in the precision of BrumiR comes from a reduced seed length, which allows it to better deal with sequencing errors, and from an improved clustering of the reads coming from the same miRNA candidates, plus some code improvements. Overall, our precision for animals and plants increased by 1.67X and 4.09X on average respectively, which led to a combined F-score increased by 1.5X and 3X for animals and plants respectively, significantly reducing the rate of false-positives of our previous version. This can be further reduced when a reference genome is available and BrumiR2Reference is used. Additionally, we also provided a new script to annotate the candidates using the miRBase entries, providing a subset of high confidence BrumiR predictions. The novel candidates can be further refined using additional criteria (such as coverage, presence on several miRNA-seq experiments, etc.) that we describe in Figure 4 and Supplementary FigureS10, in the discovery of novel miRNAs in the Arabidopsis genome using BrumiR.<br>Moreover, we intend to improve our method by implementing a random forest classifier that will enable a better characterization of the novel candidates not present in miRbase. The random forest model learns the sequence features of the miRNA annotated in miRbase and can be used to assign a probability of being a miRNA or not for novel BrumiR candidates for species with or without a reference genome. This feature should be included in a future version of our method.<br>However, at this point we believe that in the absence of a reference genome, BrumiR is able to provide a list of potential miRNA candidates which is not usually possible with the other genome-based prediction tools that have a similar prediction power. We compared BrumiR against the best genome based miRNA discovery tool and we had comparable or better results, even without using a reference genome.<br><br>The updated text is in page 8, lines 13; page 12, lines 3-15 ; page 13, lines 1-3; page 14, lines 21-22 ; page 17, lines 5-6; page 23, lines 1-13; page 25, lines 18-22; page 26, lines 1-4.<br><br><br><br>5. Wet-lab validation (e.g. Luciferase assay) for the identified novel miRs will leverage the real-life usefulness of BrumiR. This is extremely important, as the tool showed a high false discovery rate.<br>We agree that functional validation of miRNAs predicted by BrumiR is an important point in order to highlight the usefulness of our tool; and we intend to continue our studies with Arabidopsis' novel miRNA candidates in the near future to provide additional evidence towards the in vivo functions of such candidates in a more specialized manuscript. Unfortunately, this is not within the scope of our study and more specifically the current global pandemic has not helped us finish such functional studies, because this part of the work has been done in Chile where our partner wet-lab was closed most of the part of the pandemic and currently is resuming is normal operation.<br><br>Even so, we would like to point out that current criteria to validate and annotate miRNAs in plants are based on experimental evidence coming directly from the sequencing libraries and that confirmation by blot of the expression of the miRNA or miRNA* is disallowed, as proposed by Axtell 2018*. What we think could be done in order to improve the sRNA-seq dataset instead would be to enrich the sequencing libraries with functional sRNAs coupled to proteins (as proposed in Grentzinger et al., 2014**). At this point, we believe that we currently fulfill several state-of-the-art criteria

Powered by Editorial Manager® and ProduXion Manager® from Aries Systems Corporation

for miRNA annotation in plants. We have revised all the criteria exposed in Axtell, 2018* for novel miRNA candidates predicted by BrumiR on the Arabidopsis miRNA-seq data. We concentrated our effort on 22 novel miRNA candidates that were expressed in at least 3 replicates, and we found that 4 of them fulfilled at least 7 of 8 criteria, with 2 of them fulfilling all the criteria exposed in Axtell 2018. We have updated this part of the manuscript (Figure 4, Figure S10, and Table S8, S9, S10) accordingly to reflect these changes and we thank the reviewer for pointing out the need for more comprehensive criteria to reflect the accuracy of BrumiR when predicting new candidates.

(*) Axtell, M. J., & Meyers, B. C. (2018). Revisiting criteria for plant microRNA annotation in the era of big data. The Plant Cell, 30(2), 272-284.
(**) Grentzinger T, Armenise C, Pelisson A, Brun C, Mugat B, Chambeyron S. A user-friendly chromatographic method to purify small regulatory RNAs. Methods. 2014 May 1;67(1):91-101. doi: 10.1016/j.ymeth.2013.05.011. Epub 2013 May 28. PMID: 23727218.

The updated text is in page 19, lines 6-14; page 19, lines 21-23; page 20, lines 1-11; page 21, lines 1-10; page 38, lines 17-24.

Minor comments:
1. MiRNA maturation involves RNA editing. Can the authors comment on how this would be handled and captured by BrumiR. It seems that the authors allow mismatches when clustering the potential miRNAs via edlib library. It is interesting to know whether or not, or to what extent, edlib would help in including RNA edited candidates in the final result.

We use the edit distance library to allow a maximum of 2 nt differences in the cluster step of BrumiR. Then, for each cluster, we select the most expressed candidates but a reference is kept for all the cluster members. It is possible to use these clusters to identify miRNA candidates that may be RNA edited but the final output of BrumiR contains only one representative member of each cluster. We have included a flag in the output of BrumiR that reports the size of the cluster for each miRNA candidate. This allows us to explore if a candidate might be RNA edited or not. This is provided in a list as one of the BrumiR output files (*.edit_clusters.ED2.txt). Overall, BrumiR permits a mismatch maximum of two nucleotides when clustering candidates and therefore allows for the identification of candidates with potential RNA edition.

Reviewer #2:
The authors here present BrumiR, a de Bruijn-based method to discover miRNAs independently of a reference genome. Today most miRNA discovery and annotation is done by mapping sequenced RNAs to readily available reference genomes and analyzing the mapping profiles. However, there are some uses cases where the genome-free approach is needed (particularly for species that have no reference genome or where the genomes have missing parts); therefore BrumiR could potentially be useful for the community. However, the comparison to existing tools needs to be done in a more careful way.

Major comments:
1- RFAM filtering is not really part of the prediction step, this is rather a filtering step. Therefore, to make a fair comparison with mirnovo (the other genome-free tool), BrumiR should additionally be run without RFAM filtering, and mirnovo should additionally be run using the exact same RFAM filtering.

We used the RFAM database to filter out other kinds of RNAs present in the miRNA-seq experiment. Mirnovo calculates a consensus sequence for each cluster (candidate) and then an alignment is performed against the RFAM database to identify candidates matching rRNAs and tRNAs, and these matching candidates are filtered out by mirnovo. The latter step is performed by mirnovo by default and cannot be turned off (there is not a parameter allowing this). In BrumiR, we used the RFAM

database to filter RNAs not associated with miRNAs. We built a k-mer database excluding all the RFAM families annotated as miRNAs. All the BrumiR candidates matching this k-mer database are excluded. This step is similar for both tools with the same goal (excluding other RNAs) but using different algorithms (alignment vs k-mer matches). We therefore considered that the comparison that we did is appropriate and fair for both tools. On average, 1% of the BrumiR candidates are filtered-out on this step.

The updated text is in page 9, lines 19-21.

2- it appears that 16-mers from miRBase miRNAs were specifically excluded from the RFAM catalog used for the filtering, which is reasonable. However, the miRNAs from the exact benchmarked species should not be included in the used miRBase 16-mer catalog, to avoid circular reasoning.

Yes, we agree with the reviewer that including miRNAs in this step may generate circular reasoning. This represents a final step of BrumiR where the aim is to remove sequences related to rRNAs and tRNAs, but we do not annotate or guide BrumiR towards previously annotated miRNA sequences present in the RFAM database. To achieve this, the 16-mer database was built from the RFAM database excluding all the families in all the taxa related to miRNA genes (529 families excluded), conserving the most abundant k-mers (more abundant than 5). Moreover, all 16-mers present in the miRBase database were also excluded. In summary, we excluded any reference to known miRNA sequences present in RFAM from the 16-mer database.

The updated text is in page 9, lines 19-21.

3- miRDeep2 software should ideally not be run with default options - this is particularly important since the miRDeep2 performance in this manuscript appears lower than what is reported in other studies (e.g. Friedlander et al. 2012). First, reference mature miRNAs from a related and well-annotated species should be included to support the prediction. Second, a score cut-off should be used that gives a decent signal-to-noise ratio according to the miRDeep2 output overview table (for instance 5:1). Third, all read pre-processing and genome mapping should be performed with the mapper.pl script which is part of the miRDeep2 package.

We agree with the reviewer that the ideal case is to use miRDeep2 according to the recommendations suggested by the developers (Friedländer et al, 2012). For this reason, we first used the script proposed by the developers to map the reads to the reference genome (mapper.pl). Then, we used the miRDeep2 pipeline in a “de novo” mode because we wanted to make a prediction without using previous knowledge, as we did with mirnovo and BrumiR. Finally, we selected all the predictions with a minimum miRDeep2 score of 5.1 (signal-to-noise ratio).
Also, the benchmark presented in the miRDeep2 manuscript was performed in a different way, because they used a pool of several samples, and the metrics were computed using all predictions, and for this reason, they are not comparable to our benchmark. As we mentioned above, there is no standard for benchmarking the miRNA prediction methods, which means that each new method tries to make the comparison in its own way and the metrics used could be quite different depending on the design of the benchmark.
miRDeep2 presented a good performance in most of the datasets, having the highest precision rate. However, we observed that it is a very conservative method because it does not predict many new candidates, even using the "de novo" mode.

The updated text is in page 35, lines 8-9, lines 11-13; page 13, lines 17; page 14, lines 1-2; page 15, lines 1-3;.

4- it appears that only miRNA-derived sequences were included in the simulated data. In fact, real small RNA-seq data typically contains fragments from other known types of RNA and also sequences from unannotated parts from the genome. Therefore, the authors should use simulated data that also includes samples from RFAM and randomly sampled sequences from the reference genome (for instance 10% of each). Overall, the use of simulated sequence data could be put a bit in the background in this study, since real small RNA-seq data is in fact readily available these days and

typically has a structure that is not easy to simulate. Further, there is little reason not to use real data, since the miRNAs in miRBase tend to be reasonably well curated for most species and therefore can function well as a gold standard for benchmarking.

We followed the reviewer's suggestion and we added to our simulated data sequences from the RFAM and the genomes for each of the species included in the benchmark. Now the simulated data include 10% of sequences from RFAM and 10% of random genomic sequences. We have repeated the synthetic benchmark and the new results are reported in the corresponding section. We agree that it is hard to mimic the structure of real miRNA-seq experiments, but the simulated data provide a good overview of the performance of the tools as well as a more controlled way to compare such performance since all the expressed miRNA sequences are known, which may not be the case for real data. Finally, we also included an extensive benchmark for the same species using real miRNA-seq data (Supplementary TableS4) and used the miRbase entries as the gold standard for computing the benchmark metrics as proposed by the reviewer.

The updated text is in page 11, lines 11-17; page 34, lines 19-22; page 35, lines 1-2.

5- precision of BrumiR is in some cases lower than 0.2, for instance for one mouse dataset. From this dataset ~3000 mouse miRNAs are reported - the majority of which are not in miRBase and can reasonably be presumed to be false positives. The authors should comment on why this particular dataset appears to produce so many false positives for BrumiR - could this have to do with the prevalence of piRNAs that the software cannot easily discern from miRNAs? Also, the authors should reflect on what kind of use cases could tolerate these thousands of false positives. Would this be for generating candidates for downstream high-throughput validation?

We took into account the recommendations of Reviewer #1 which led to a considerable improvement in our predictions and improved the benchmarking metrics in general across all the datasets. After we changed the k-mer size to 14, we improved over all the datasets the precision rates of BrumiR (Supplementary TableS1, TableS4). Moreover, to address the question of Reviewer #2 related to the possibility of having piRNAs predicted as putative miRNAs in this dataset, we compared the predictions of BrumiR against the repository of piRNAs annotated for Mus musculus (https://www.pirnadb.org/). We observed that less than 4% of BrumiR candidates have a match with a piRNAs annotated in this database (Supplementary TableS9), a 3% and 10% of predictions of miRDeep2 and mirnovo, respectively, have a match with piRNAs. We can observe that none of the miRNA discovery tools would be capturing piRNAs instead of miRNAs, according
to the data presented here.

The updated text is in page 12, lines 3-15; page 14, lines 20-21; page 15, line 1-3; page 23, lines 10-13.

6- the authors should either benchmark BrumiR against the genome-free methods miReader and MirPlex, or explain why this comparison is not relevant.

We used mirnovo as a de novo predicted tool. As in the mirnovo paper, the authors compare mirnovo against these tools, miReader and MirPlex, and mirnovo has the best performance, we chose to compare BrumiR only against the best performance tools only in a genome-based and de novo approach, meaning against respectively miRDeep2 and mirnovo, as we explained above we compared BrumiR with 4 different miRNA discovery tools using a reduced version of the real dataset. The results provided in Supplementary TableS5 showed that the highest F-score rates are for BrumiR, miRDeep2, miR-PREFeR and mirnovo (Supplementary FigureS9). We decided to benchmark BrumiR in a simple but exhaustive way selecting the top performers for genome- based and genome-free methods.

The updated text is in page 13, lines 17; page 14, lines 1-2; page 36, lines 11-16.

Minor comments:
- the brief introduction to miRNA biology should be carefully edited by an expert in the field. Currently, very old reviews are being cited (e.g. Bartel 2004), and some of the

other references appear to be a bit spurious (e.g. why focus on plant host-pathogen interactions out of the hundreds of established functions of miRNAs?). The excellent review of Dave Bartel from 2018 contains references to numerous milestone studies that the introduction could build on.
We included miRNA biology background and we verified our references. Thanks to the reviewers for having pointed this out.

The updated text is in page 3, lines 6-20.

- the authors write on page 2 that genome-based methods struggle with a high rate of false positive prediction, citing [9]. However, this is a mis-reference, since the reference [9] states that methods that rely on _only_ the genome and do not leverage on small RNA-seq data have high false positive rates.
We changed this sentence.

The updated text is in page 4, lines 20-21.

Reviewer #3:
The manuscript by Moraga et al. describes BrumiR, a software devoted to the de novo identification of miRNAs from deep sequencing experiments of the RNA fraction at low molecular weight.
In contrast with existing tools, BrumiR is based on de Bruijn graphs, generated directly from raw fastq reads. The performances on simulated and real sequencing data, in terms of precision, recall and F-Score, are very good. In addition, the tool is ultra-fast, enabling the analysis of huge amount of data.
I have tried to use BrumiR but I always got a GLIB error. I have tested the script on different Linux and Mac computers but I was not able to fix the GLIB error. It seems that a very recent version of the GLIB library is required. So, unfortunately, I didn't have the possibility to test the program and look at the outputs.
Major concerns:

1- I was not able to run the program and, thus, provide a correct revision. In my opinion, the github page should take into account this by providing the minimal software and hardware architecture to run BrumiR. Authors could also include a copy of the output files (by the way, there is a typo in the description of the second output file).

We do thank the reviewer for raising this point and we created a docker container that provides all the needed software to facilitate the execution of BrumiR. The docker image is available on dockerHub at (https://hub.docker.com/repository/docker/camoragaq/BrumiR) and instructions for downloading the image are provided on the Github repository of BrumiR. Additionally, we have created a demo dataset hosted at https://github.com/camoragaq/BrumiR_demo where we have included all the expected output files for the demo dataset.

The updated text is in page 40, lines 4-6.

2- Since the tool is able to identify novel miRNAs and look also at known ones, they could provide an output file including the read count per miRNA. In addition, since the tool is expected to be ultra-fast (not checked … see above), the differential gene expression analysis could also be implemented.

BrumiR outputs an abundance estimation based on the k-mer counts for each predicted miRNA candidate. Currently, BrumiR quantify each candidate using the total number of k-mers (KC), and the average number of k-mers per base (KM). Both values can be used as proxy to access the expression of each miRNA candidate. However, how has been previously shown for transcriptome mRNAseq data the accurate estimation of gene expression based on k-mers require the development of specific algorithms (Kallisto, Salmon) (Bray et al., 2016*) (Patro et al., 2017**). The adaptation of these algorithmics ideas to miRNAseq data is one of the main ideas that we are

	going to develop in a subsequent manuscript of the BrumiR toolkit.<br>(*) Bray, N., Pimentel, H., Melsted, P. et al. Near-optimal probabilistic RNA-seq quantification. Nat Biotechnol 34, 525–527 (2016). https://doi.org/10.1038/nbt.3519<br>(**) Patro, R., Duggal, G., Love, M. I., Irizarry, R. A., & Kingsford, C. (2017). Salmon provides fast and bias-aware quantification of transcript expression. Nature methods, 14(4), 417–419. https://doi.org/10.1038/nmeth.4197<br><br>3- I suggest also implementing a graphical output. A sort of summary in a decorated html page.<br><br>We followed the reviewer's suggestion and we now provide an R notebook to create an interactive and graphical summary of the output of BrumiR. We do plan to extend these capabilities in the future to further enhance the graphical report (i.e. by providing functions to quickly determine differentially expressed miRNAs). A full example of the R Notebook is provided with the demo dataset.<br><br>The updated text is in page 40, lines 5-6.<br><br>4- By using BrumiR, authors analyze miRNAs in Arabidopsis during the development, discovering three novel miRNAs. Although bioinformatics evidences indicate that they could be real miRNAs, an experimental validation is required. Indeed, these miRNAs have been detected by BrumiR only. I think that this validation could be easily done because authors directly performed sRNAseq data. In my opinion, this experiment could really improve the manuscript and assess the high performance of BrumiR.<br><br>We agree with the reviewer that experimental validation of the candidate miRNAs is an important step and a valuable suggestion for our work. Following the reviewer's suggestion, we have revised all the updated criteria on plant miRNA annotation based on Axtell, 2018* for each novel candidate miRNA predicted by BrumiR using the Arabidopsis thaliana datasets. These criteria are based directly on precursor and miRNA features that can be determined directly from the sRNA-Seq reads. Considering these criteria, we found that 2 of our miRNA candidates fulfill all of them (Figure 4, Figure S8, S9, S10). As such, these results highlight the usefulness of BrumiR for predicting new candidate miRNAs even in model species where the miRNA catalogs are highly complete. Although, this last results section of the BrumiR manuscript highlight the power of BrumiR to discover new miRNAs even in highly curated genomes, we believe that the biological insight and subsequent validations may be the focus of a more specialized manuscript rather than the current one where the focus is the new algorithmic ideas that we are presenting.<br><br>(*) Axtell, M. J., & Meyers, B. C. (2018). Revisiting criteria for plant microRNA annotation in the era of big data. The Plant Cell, 30(2), 272-284.<br><br>The updated text is in page 19, lines 6-14; page 19, lines 21-23; page 20, lines 1-11; page 21, lines 1-10; page 38, lines 17-24.<br><br>Again, we would like to thank all referees for their crucial suggestions that inspired us to further improve BrumiR, led to remarkable new benchmark results, and made the flow of the Results part more natural.<br>Sincerely,<br>Carol Moraga on behalf of all authors,
**Additional Information:**	
**Question**	**Response**
Are you submitting this manuscript to a special series or article collection?	No

Powered by Editorial Manager® and ProduXion Manager® from Aries Systems Corporation

**Experimental design and statistics**<br><br>Full details of the experimental design and statistical methods used should be given in the Methods section, as detailed in our Minimum Standards Reporting Checklist. Information essential to interpreting the data presented should be made available in the figure legends.<br><br>Have you included all the information requested in your manuscript?	Yes
**Resources**<br><br>A description of all resources used, including antibodies, cell lines, animals and software tools, with enough information to allow them to be uniquely identified, should be included in the Methods section. Authors are strongly encouraged to cite Research Resource Identifiers (RRIDs) for antibodies, model organisms and tools, where possible.<br><br>Have you included the information requested as detailed in our Minimum Standards Reporting Checklist?	Yes
**Availability of data and materials**<br><br>All datasets and code on which the conclusions of the paper rely must be either included in your submission or deposited in publicly available repositories (where available and ethically appropriate), referencing such data using a unique identifier in the references and in the "Availability of Data and Materials" section of your manuscript.<br><br>Have you have met the above requirement as detailed in our Minimum Standards Reporting Checklist?	Yes

Manuscript Click here to access/download;Manuscript;BrumiR_main.pdf

OXFORD (GIGA)^n SCIENCE

GigaScience, 2020, 1–19

doi: xx.xxxx/xxxx
Manuscript in Preparation
Technical note

TECHNICAL NOTE

BrumiR: A toolkit for *de novo* discovery of microRNAs from sRNA-seq data.

Carol Moraga[1,2,†], Evelyn Sanchez[3,4], Mariana Galvão Ferrarini[5], Rodrigo A. Gutierrez[4,6,7], Elena A. Vidal[3,4,8] and Marie-France Sagot[1,2,†]

[1]Inria Grenoble Rhoñe-Alpes, 655, Avenue de l'Europe, 38334 Montbonnot, France. and [2]Université de Lyon, Université Lyon 1, CNRS, Laboratoire de Biométrie et Biologie Evolutive UMR 5558, F-69622 Villeurbanne, France. and [3]Centro de Genómica y Bioinformática, Facultad de Ciencias, Universidad Mayor, Chile. and [4]Millennium Institute for Integrative Biology iBio, Chile. and [5]Université de Lyon, INSA-Lyon, INRA, BF2i, UMR0203, F-69621 Villeurbanne, France. and [6]Departamento de Genética Molecular y Microbiología, Facultad de Ciencias Biológicas, Pontificia Universidad Católica de Chile. and [7]FONDAP Center for Genome Regulation. and [8]Escuela de Biotecnología, Facultad de Ciencias, Universidad Mayor.

[†] To whom correspondence should be addressed: Carol Moraga – camoragaq@gmail.com and Marie-France Sagot – marie-france.sagot@inria.fr

Abstract

Background: MicroRNAs (miRNAs) are small non-coding RNAs that are key players in the regulation of gene expression. In the last decade, with the increasing accessibility of high-throughput sequencing technologies, different methods have been developed to identify miRNAs, most of which rely on pre-existing reference genomes. However, when a reference genome is absent or is not of high quality, such identification becomes more difficult. **Results:** In this context, we developed BrumiR, an algorithm that is able to discover miRNAs directly and exclusively from sRNA-seq data. We benchmarked BrumiR with datasets encompassing animal and plant species using real and simulated sRNA-seq experiments. The results demonstrate that BrumiR reaches the highest recall for miRNA discovery, while at the same time being much faster and more efficient than the state-of-the-art tools evaluated. The latter allows BrumiR to analyze a large number of sRNA-seq experiments, from plants or animals species. Moreover, BrumiR detects additional information regarding other expressed sequences (sRNAs, isomiRs, etc.), thus maximizing the biological insight gained from sRNA-seq experiments. Finally, when a reference genome is available, BrumiR provides a new mapping tool (BrumiR2reference) that performs an *a posteriori* exhaustive search to identify the precursor sequences. **Conclusions:** In summary, we present a new and versatile method that implements novel algorithmic ideas for the study of miRNAs that complements and extends the currently existing approaches. The code of BrumiR is freely available at https://github.com/camoragaq/BrumiR.

Key words: sRNA; miRNAs; *de novo* miRNA discovery; NGS; sRNA-seq; de Bruijn graph; algorithms.

Compiled on: August 27, 2020.
Draft manuscript prepared by the author.

Findings

Background

MicroRNAs (henceforth denoted by miRNAs) are small RNA molecules usually shorter than 25 nucleotides (nt), which have been identified as crucial regulators of gene expression mostly at the post-transcriptional level [1]. miRNAs are involved in a wide range of biological processes including cell cycle, differentiation, apoptosis and disease [2]. They have been the target molecules for a large number of important applications, more particularly in cancer where miRNAs have been shown to play important roles in driving or suppressing tumor spread [3, 4]. In plant species, unravelling host-pathogen interactions mediated by miRNAs may shed light on plant development and its relation with the environment, both essential knowledge that can lead to the discovery of new biotechnological products for the agricultural industry [5, 6].

Since the first classification and annotation of miRNAs [7, 8], accurately identifying them as well as the regulatory networks in which they are involved has proven difficult [9, 10]. Accurate prediction of known and novel miRNAs along with their targets is however essential for increasing our understanding of the miRNA biology [3]. Nowadays, a common experimental practice is to identify miRNAs and their expression patterns using next generation sequencing technologies (NGS) [11]. Commonly, NGS experiments are able to generate more than 20 million sRNA-seq reads, thus promoting the development of algorithms to transform and process such data into biological information [12].

Currently, there are two computational strategies for the discovery of miRNAs: 1) genome-based approaches that rely on the mapping of the sRNA-seq reads to a reference genome and subsequent evaluation of the sequences generating the characteristic hairpin structure of miRNA precursors [9]; 2) machine-learning approaches which rely on the biogenesis features extracted from the knowledge on miRNA sequences available in databases such as miRBase [13] and on the analysis of the duplex structure of miRNAs [14]. Genome-based methods, that have been updated at the pace of the evolving NGS technologies, are the most widely used tools in this field, and their results have populated the public miRNA repositories [12]. Such methods are the natural choice for the study of model species with high quality reference genomes available. However, it has been shown that most of the genome-based tools struggle with a high rate of false positive predictions [9]. Additionally, a critical step of such tools is the use of genome aligners [15, 16] to map the sRNA-seq reads to the reference genome. Mapping short (<30 nt) and very similar sequences to a large, complex, and repetitive reference genome is however a difficult and error-prone task [17]. Genome-based methods are thus highly sensitive to the aligner selected as well as to the parameters employed and the thresholds chosen (*e.g.* number of mismatches allowed) in order to discard mapping artefacts generated from sequencing errors [18]. Furthermore, despite all the advancements in the sequencing technologies and *de novo* assembly methods, few complete genomes are available today, which is a recurring problem that researchers working on non-model species face [19]. The lack of a high quality reference genome thus reduces the possibilities for discovering novel miRNAs [14]. Genome-based methods such as miRDeep [20], miRDeep2 [21], and miR-PREFeR [22] are included in this group.

On the other hand, new methods such as miReader [23], MirPlex [24], and mirnovo [14], in particular using machine-learning approaches, were specifically developed as an alternative to discover miRNAs in species without a reference genome. In the case of mirnovo, the initial step involves the clustering of the sRNA-seq reads performing an all-vs-all read comparison that is followed by a subsequent classification of the clusters into putative miRNAs using pre-trained models. The performance obtained by such methods on well-annotated species is comparable to those achieved by genome-based methods [9]. However, relying exclusively on annotated miRNAs for training machine learning models may introduce a bias towards the identification of well-characterized miRNAs over species-specific ones [12]. Nonetheless, machine learning methods have demonstrated that it is possible to discover miRNAs using only the sequence information present in the sRNA-seq experiment [14].

There remains however a need to go further in the development of algorithms for finding novel miRNAs in non-model species using only the sequence information. With this purpose in mind, the adoption of a special type of graphs called *de Bruijn* graphs may be considered. This is a widely used approach for the *de novo* reconstruction of genome or transcriptome sequences [25]. It therefore appears to be a plausible option for organizing, clustering and assembling the sequence information present in sRNA-seq experiments. However, accommodating the de Bruijn graph approach for the discovery of miRNAs involves the development of new methods to address the specific characteristics of sRNA-seq data. Indeed, mature miRNA sequences are short (18-24 nt), thus limiting the overlap length for building a de Bruijn graph which in turn impacts the global topology by inducing tangled graph structures. Moreover, miRNAs captured in a sRNA-seq experiment have variable expression, from low (few reads) to highly expressed (thousands of reads), which may induce spurious graph connections that should be removed in order to isolate and detect both types of miRNAs. Finally, the sequencing errors present in sRNA-seq data further induce spurious connections and are harder to detect as compared to genomic data due to the variable expression and the shorter lengths of the miRNAs. Overall, using a de Bruijn graph to analyze sRNA-seq data and extract information from such data seems thus counterintuitive as mature miRNAs are captured full-length by the current NGS technologies. However, a de Bruijn graph has several interesting properties for the discovery of miRNAs, mainly due to the fact that it encodes all the sRNA-seq sequence information at once in a compact and connected representation (graph), without the need to perform an all-vs-all read comparison or mapping to a reference.

In this paper, we present BrumiR, a *de novo* algorithm based on a de Bruijn graph approach that is able to identify miRNAs directly and exclusively from sRNA-seq data. Unlike other state-of-the-art algorithms, BrumiR does not rely on a reference genome, on the availability of close phylogenetic species, or on conserved sequence information. Instead, BrumiR starts from a de Bruijn graph encoding all the reads and is able to directly identify putative miRNAs on the generated graph. BrumiR also removes sequencing errors and navigates inside the graph detecting putative miRNAs by considering several miRNA biogenesis properties (such as expression, length, topology in the graph). Along with miRNA discovery, BrumiR can also assemble and identify other types of small and long non-coding RNAs expressed within the sequencing data. Finally, when a reference genome is available, BrumiR provides a new mapping tool (BrumiR2reference) that performs an exhaustive search to identify and validate the precursor sequences. We extensively benchmarked BrumiR on animal and plant species using simulated and real datasets. The benchmark results demonstrate that BrumiR is very sensitive, besides being the fastest tool, and its predictions were supported by the characteristic hairpin structure of miRNAs. Finally, we also applied BrumiR to the discovery of miRNAs of *Arabidopsis thaliana* and identified three novel high-confidence miRNAs involved in root development. These putative miRNAs were not discovered before by any other software, thereby showing the potential of using different approaches even in the case where high quality genomes are available. The code of BrumiR is freely available at https://github.com/camoragaq/BrumiR.

Algorithm

Building a de Bruijn graph for sRNA-seq data.

BrumiR starts by building a compact de Bruijn graph from the sRNA-seq reads given as input. De Bruijn graphs are a widely used approach in the genome assembly problem [25]. BrumiR uses this graph to organize, detect, and exploit the sequence information of sRNA-seq experiments. BrumiR takes as input sequencing files in FASTA or FASTQ formats. The sequencing data can be cleaned, using a fastq pre-processor [26] (*i.e.* fastp), to remove adapter sequences and trim low quality bases. BrumiR employs the Bcalm [27] tool to build a de Bruijn graph from the sRNA-seq reads. Bcalm uses a node-centric bi-directed de Bruijn graph where the nodes are k-mers, that is words of length k, and an arc between two nodes if the $k-1$ suffix of one node is equal to the $k-1$ prefix of the subsequent node, representing an exact overlap of $k-1$ bases [27]. A critical parameter of any de Bruijn graph approach is the k-mer size [28]. We observed that the length of all mature miRNA sequences stored in the miRBase database (v21) [13] have a minimum value of 18 nt (Supplementary Figure S1). We thus empirically set the k-mer size equal to 18. Bcalm compacts the nodes of the de Bruijn graph into maximal unipaths by gluing all the nodes of the graph with an in-degree and an out-degree equal to one, thus generating the so-called unipath graph [27]. The unipath graph is the starting point of BrumiR (Figure 1.1). Notice that the unipath graph generated by Bcalm does not represent what is expected for a set of mature miRNAs (one connected component for each miRNA) and therefore further graph operations are needed. BrumiR uses a minimum k-mer frequency (KM value) of 5 and all k-mers with lower frequency are ignored, without losing most of the information contained in the sequencing reads (Supplementary Figure S2).

Removing sequencing errors from the unipath sRNA-seq graph.

BrumiR deletes from the unipath graph all the nodes that have only one connection (degree equal to 1), known as dead-end paths or tips [29]. Usually, these nodes have a low abundance value associated to them (KM less than or equal to 5, the default parameter). Moreover, BrumiR deletes isolated nodes (degree equal to 0) having a low abundance; isolated nodes highly expressed are however conserved for further analysis. All these nodes are likely artifacts generated from sequencing errors because they are not deeply expressed in the sRNAs-seq reads [30]. BrumiR iterates this step 3 times in order to prune and clean the unipath graph (polishing). This operation, called 'tip removal', edits the original unipath graph, and therefore a new unipath graph with a new structure is generated (Figure 1.2).

An expressed mature miRNA has uniform coverage.

The unipath graph of a set of miRNAs from a sRNA-seq experiment has non-uniform coverage as different miRNAs and other elements may be connected in a single big component (Figure 1.1). BrumiR evaluates each connection of the unipath graph to identify those that link two nodes with a large expression difference. According to the miRNA biogenesis, after a stable miRNA precursor is cleaved by Dicer, among its three products, the miRNA mature sequence is the most abundant and when it is sequenced, it has a uniform expression along its sequence [20]. Thus due to miRNA biogenesis, it is possible to capture the complete miRNA mature sequence having a homogeneous expression [9] directly from the sRNA-seq experiments. BrumiR expects a similar KM value for k-mers originating from the same mature miRNA gene. Accordingly, if we observe two connected nodes that show a big difference in their abundance values, this connection is deleted and we keep the nodes unconnected. In particular, two unipaths $U = [a, b]$ connected in the graph have a KM value associated to them that represents their coverage from the reads information. BrumiR scans all the neighbor connections and if the difference between their KMs is larger than three-fold, the connection is deleted ($Ui_{km}/Uj_{km} > 3$). In this way, BrumiR defines a relative threshold that will depend on each unipath neighborhood in the graph. Finally, BrumiR repeats the tips removal step to eliminate new low frequency isolated nodes (Figure 1.4).

miRNAs and other sequences are captured in single connected components.

After the previous steps of BrumiR, a new unipath graph emerges, with a new structure. It is thus necessary to identify and classify the new connected elements within the graph (Figure 1). A connected component (CC) of a graph is a maximal strongly connected subgraph [31]. BrumiR computes the CCs of the unipath graph, and then each CC is processed independently to identify miRNA candidates as well as to discard other sequences present in the unipath graph.

BrumiR *classifies low abundance non-linear topologies as sequencing artefacts.*

BrumiR detects topologies that are potentially related to sequencing errors and thus unlikely to be miRNA candidates. The shapes of these topologies were identified by visual inspection of several unipath graphs and are described in detail in Supplementary Figure S3. Usually they have low KM and are composed of lowly expressed branching nodes with 3, 4 or 5 connections to the principal structures in the graph (Supplementary Figure S3). Moreover, we observed that the sequences contained in these topologies were usually redundant and contained in other linear and more expressed CCs. In this way, we are not discarding relevant sequence information. BrumiR removes about 10% of the CCs in this step.

Re-assembling unipaths within each CC.

BrumiR re-assembles all unipaths present in the linear CCs by bundling the nodes with in and out degree equal to 1 into a new unipath. BrumiR classifies them into different types based on their length. The latter is the length of the sequence represented by the new unipath. All CCs having a length between 18 and 24 are stored as potential miRNA sequences. The CCs corresponding to an isolated node that have high KM (KM>50) are included in the latter group. CCs with lengths over 24 are classified as longer sequences or other types of genomic sequences captured along with the miRNAs. The longer sequences are put aside for later analysis. Moreover, BrumiR identifies circular CCs and branching CCs. The former are circular unipaths and the latter CCs with a high number of branching nodes. Branching CCs are not considered in the subsequent steps because they are likely sequencing errors (low abundance) or contamination present in the sRNA-seq data (Supplementary Figure S4).

Re-clustering potential miRNAs.

After grouping unipaths by CCs, BrumiR builds an overlap graph to rescue the missing connections between potential miRNA candidates sharing an overlap with another candidate. First, BrumiR adds all the candidates as nodes of the overlap graph, then an all-vs-all k-mer comparison is performed using exact overlaps of length k=15. Candidates sharing an exact overlap are connected in the overlap graph. Then, the connected components are computed to identify clusters of miRNA candidates, and the most expressed candidate within each component is selected as the representative candidate of the cluster. The representative candidates are compared all-vs-all in a second overlap step that allows a maximum edit distance of 2, which is implemented using the edlib library [32]. BrumiR then builds a second overlap graph, computes again the connected components, and selects the most expressed candidate as the representative of each cluster. The other members of each connected component are classified as putative isomiRs and saved in a file for later analysis.

Identifying other expressed RNA sequences.

In sRNA-seq experiments, different types of RNAs are expressed, some of which, such as small non-coding RNA elements, may have similar length with miRNAs [33]. The RFAM database [34] is a collection of curated RNA families including three functional classes of RNAs (non-coding, cis-regulatory elements, and self-splicing RNAs), which are classified into families according to their secondary structure and sequence information (Covariance Models) [34]. We downloaded 3,017 RNA families present in RFAM (v14.1) and excluded 529 miRNA families [35]. The sequences of 2,488 RFAM families were concatenated (a total of 2,736,549

sequences) and used to build a 16-mer database with the KMC3 k-mer counter tool [36] ("-fm -n100 -k16 -ci5"). All distinct 16-mers with a frequency lower than 5 were excluded, leading to a total of 6,204,556 distinct 16-mers related to RNA elements. Additionally, we downloaded all the mature miRNA sequences from miRBase (v22.1) [13] and built a 16-mer database with KMC3 ("-fm -n100 -k16 -ci1 mature.fa.gz"). RFAM 16-mers matching 16-mers from the 16-mer mature miRBase database were excluded from RFAM, leading to a 16-mer RFAM database with a total of 6,204,487 distinct 16-mers. Finally, the BrumiR candidates (18-24 length) were matched to the 16-mer RFAM database, and matching candidates were excluded and reported as sequences potentially associated to other RNA elements. The BrumiR candidates passing the aforementioned filter are reported as the final list of miRNA candidates.

***Identifying precursor sequences for* BrumiR *candidates* (BrumiR2reference).**

Unlike current state-of-the-art tools that perform miRNA discovery by mapping the sRNA-seq reads to a reference genome, BrumiR generates candidates by operating directly on the sRNA-seq reads. The reduced list of potential BrumiR miRNA candidates permits the computation of a more exhaustive alignment than when mapping directly the sRNA-seq reads to the reference genome. BrumiR aligns each candidate to the reference genome using an exact alignment method that computes the edit distance [37] between two strings and thus support mismatches, insertions and deletions. The BrumiR2reference tool divides the reference genome in non-overlapping windows of 200bp (adjustable parameter), then the window is indexed using 12-mers and each miRNA candidate is matched in both strands (split at 12-mers). When a 12-mer match is found, an exhaustive alignment is computed between the window and the matching miRNA candidate. The alignment is performed using a fast implementation of Myers' bit-vector algorithm [32]. A miRNA candidate is stored as hit if the alignment in the current genomic window has an edit distance less than or equal to 2. After scanning all the genomic windows, the vector of hits is sorted by miRNA-candidate; edit distance (0-2), and alignment sequence coverage. For a single miRNA-candidate, a maximum of 100 genomic locations (best hits) are selected. BrumiR2reference then builds a potential precursor sequence for each selected hit using a strategy similar to the ones employed by miRDeep2 [21] and Mirinho [38]. BrumiR excises the potential precursor hairpin sequence from the flanking genomic coordinates of the reported miRNA candidate hits (mature sequence) in both strands. Potential precursor hairpin sequences of length 110 bp are built for animal species from both strands, while for plant species hairpin sequences of lengths 110, 150, 200, 250 and 300 bp are built from both strands [37]. Secondary structure prediction for all the potential precursor sequences is performed using RNAfold (v2.4.9) [39]. Secondary structures with a minimum free energy in the range of 15-80 kcal/mol are checked for a hairpin loop characteristic of miRNAs [40] (Supplementary Figure S5). Structures with a hairpin loop composed of a single segment without pseudo-knot, multi-loops, external loops and with less than 5 bulges, 3 dangling ends, and 10 internal loops are classified as characteristic secondary structures of miRNA precursor sequences. The aforementioned filters were derived from analyzing the secondary structure of 38,589 precursor sequences stored in miRBase (v22.1) [13] using a modified version of the bpRNA program [41] (Supplementary Figure S6).

Data description

Benchmarking* BrumiR *using simulated sRNA-seq reads.

We simulated synthetic reads from animal and plant species, and compared the results of BrumiR to those obtained with the miRDeep2 [21] and miR-PREFeR [22] tools. The sRNA-seq reads were simulated using miRsim (`https://github.com/camoragaq/miRsim`), a tool that we developed specifically for simulating sRNA-seq reads from a list of known miRNA mature sequences. miRsim is based on wgsim (`https://github.com/lh3/wgsim`), which is a widely used tool for simulating short Illumina genomic reads. miRsim includes functionalities specific of sRNA-seq reads such as variable depth/coverage and shorter read lengths. miRNA mature se-

quences were obtained from miRBase [13] for animal (High Confidence) and plant species. The animal species that we considered were: *Homo sapiens*, *Mus musculus*, *Drosophila melanogaster*, *Danio rerio*, and *Caenorhabditis elegans*, while the following plant species were included: *A. thaliana*, *Oryza sativa*, *Physcomitrella patens*, *Zea mays*, and *Solanum lycopersicum*. Supplementary Table S1 provides further details (*i.e.* number of reads, number of mature miRNAs etc.) for each simulated dataset. miRDeep2 was run on the animal datasets with the default parameters providing the respective reference genome. Similarly, miR-PREFeR was run with the default parameters on the plant datasets. BrumiR was run with the default parameters on both the animal and plant datasets. The list of simulated miRNAs was considered as the ground truth, and precision, recall and F-Score quality metrics were computed to assess the performance of each discovery tool. The benchmark metrics were defined as follows:

$$recall = \frac{TP}{TP+FN}$$

$$Precision = \frac{TP}{TP+FP}$$

$$F - Score = 2 * \frac{(Recall*Precision)}{(Recall+Precision)}$$

where:

TP = true positive elements predicted as miRNAs present in the miRBase input list;

FP = false positive elements predicted as miRNAs but not present in the miRBase input list;

FN = false negative elements not predicted as miRNAs, but that were present in the miRBase input list.

Benchmarking* BrumiR *using real sRNA-seq reads.

We downloaded publicly available sRNA-seq data for the plant and animal species listed in the synthetic benchmark, and two datasets for each species were included (Supplementary Table S2). Additionally, we included mirnovo [14], a tool that can discover miRNAs without a reference genome. The predictions of BrumiR were benchmarked along with miRDeep2 (v2.0.1.2) [21] and mirnovo for the animal datasets. Similarly, miR-PREFeR [22] replaced miRDeep2 for the plant datasets (Supplementary Table S2).

The stand-alone packages of BrumiR, miRDeep2 and miR-PREFeR were used to discover miRNAs in all datasets. The software mirnovo was run using its web version because the stand-alone package was not available and the developer recommends the use of the web version instead. The miRNA discovery was performed for each sample independently using default parameters for miRDeep2, miR-PREFeR and mirnovo. In particular, we used the scripts provided by miRDeep2 and miR-PREFeR to map the reads to the reference genome, and the predictions for these tools were performed on the resulting alignment files. The mirnovo predictions were done using the animal and plant universal panel respectively, as recommended when the reference genome is not available. BrumiR was run using the command line and parameters provided in the Supplementary Material. Moreover, the predictions of BrumiR were refined using the BrumiR2reference tool on the available reference genome of the selected species (Supplementary Table S2). Benchmark metrics (precision, recall, and F-Score) were computed as before but considering all the annotated mature sequences present in miRBase (v22.1) [13] as the ground-truth.

miRNA discovery from* Arabidopsis *root samples.

A. thaliana Col-0 seedlings were grown hydroponically on Phytatrays on 0.5X Murashige and Skoog medium (Phytotechnology Laboratories, cat. M519) under long-day conditions (16h light and 8h dark) at 22°C. Total RNA was isolated from plant roots after 5, 9, 13, 17, 21, and 25 days post-germination using the mirVana miRNA Isolation Kit (Thermo Fisher Scientific, cat. AM1560). RNA concentration was determined using the Qubit RNA BR Assay Kit (Thermo Fisher Scientific, cat. Q10210), and integrity was verified by capillary electrophoresis on a Fragment Analyzer^{TM} (Advanced Analytical Technologies, Inc.). The indexed sRNA libraries were

built employing the TruSeq small RNA Sample Preparation Kit (Illumina, Inc.) following the manufacturer's instructions. Briefly, 3' and 5' adaptors were sequentially ligated to 1 μg of total RNA prior to reverse transcription and library amplification by PCR. Size selection of the sRNA libraries was performed on 6% Novex TBE PAGE Gels (Thermo Fisher Scientific, cat. EC6265BOX) and purified by ethanol precipitation. Both the library size assessment and library quantification were carried out in a Fragment Analyzer^{TM}. Finally, the libraries were pooled and sequenced on an Illumina NextSeq 500 platform.

All samples were analyzed with BrumiR separately with default parameters to identify the candidate miRNAs. We further validated the candidates having a putative precursor with a hairpin structure analysis using the BrumiR2reference tool with the reference genome for *A. thaliana* (GCF_000001735.4_TAIR10.1_genomic.fna). All validated candidate miRNAs were compared with known miRNAs described for *A. thaliana* (437) present in miRBase (v21). The putative novel miRNAs were curated manually, specifically, we checked the hairpin features, mature sequence alignment position, star sequence in the precursor sequence, mismatches in the seed region, and exact overlap in the antisense miRNA sequence [9]. Then a target analysis was performed using the Araport 11 cDNA library with the plant-specific psRNATarget algorithm (based on a best expectation score) [42].

Results

BrumiR *discovers mature miRNAs directly from the sRNA-seq reads.*

The main idea behind BrumiR is that mature miRNAs can be discovered directly from the information contained in the sequenced sRNA-seq reads. To achieve this, BrumiR starts by building a de Bruijn graph (using k-mers of size 18) from the sRNA-seq reads, then compacting all the simple nodes thus leading to the unipath graph [27] (Figure 1.1, Methods section). The unipath graph encodes all the sequence information of the sRNA-seq experiment, including sequencing errors, adapters, and other types of sequences (Figure 1.1). The construction of the unipath graph allows to avoid entirely the alignment of the sRNA-seq reads to a reference genome. Following the unipath graph construction, BrumiR cleans the graph by removing tips (dead-end nodes) with low expression/abundance (KM < 5), which are usually generated from sequencing errors (Figure 1.2).

One feature of the miRNA biogenesis is that after Dicer cleavage, the mature miRNA is the most abundant of the three by-products and when it is sequenced, it has a uniform expression along its sequence [20]. Therefore, BrumiR expects that the neighbor elements within a particular putative miRNA will have similar expression. BrumiR checks all neighbor connections (arcs), and deletes any connection with a relative expression difference larger than 3 fold (Figure 1.3, Methods section), and the new graph is cleaned again by removing tips (Figure 1.4). Clusters of unipaths (connected components) with topologies related to sequencing errors are also removed (Figure 1.5, Methods section). BrumiR attempts to re-assemble all unipaths within a connected component (CC) of the graph, and those with between 18 and 24 nt are classified as putative miRNAs, while longer re-assembled unipaths (>24 nt) are classified as other longer sequences (Figure 1.6). BrumiR then restores missing connections by re-clustering the putative miRNAs performing an all-vs-all comparison. The most expressed miRNA is selected as the representative of the cluster (Figure 1.7) and the remaining members are classified as potential isomiRs (Figure 1.7).

The final BrumiR step uses the RFAM database to discard predicted miRNAs matching to other classes of RNA (*e.g.* Ribosomal genes, Figure 1.8). As an example, BrumiR reduces the input sRNA-seq data by five orders of magnitude generating less than 1,000 putative mature miRNAs (24 million input reads to 966 miRNA candidates, see Figure 1.10). Finally, BrumiR outputs several FASTA files with all predicted mature miRNAs, all longer RNAs, putative isomiRs, other sRNAs (RFAM comparison), and a table with expression values for each predicted miRNA (Figure 1.9). Additionally, BrumiR outputs the final graph in GFA format, which can be explored using Bandage [43] (Supplementary Figure S7).

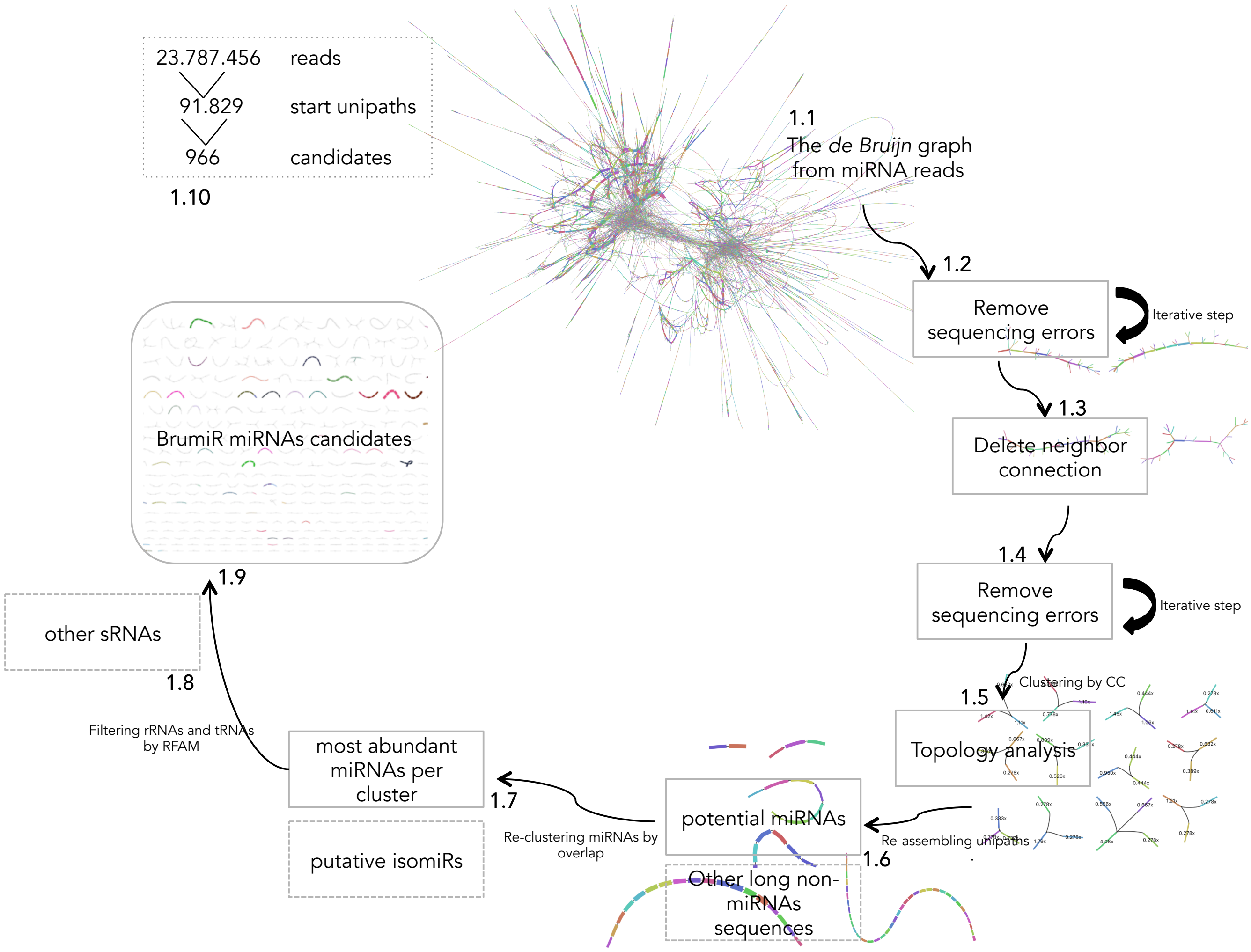

Figure 1. BrumiR algorithm. Different steps of BrumiR to discover miRNAs from sRNA-seq data. 1.1 De Bruijn graph step, 1.2 Tips removal iterative step, 1.3 Delete neighbor connection step, 1.4 Tips removal step repetition, 1.5 Topology analysis step, 1.6 Re-assembling unipaths by CC step, 1.7 Re-clustering by overlap step, 1.8 Filtering other sRNAs by RFAM step, 1.9 BrumiR candidates catalog, 1.10 Example of the reduction after all the BrumiR steps.

BrumiR ***achieves the highest accuracy on simulated data.***

To evaluate the performance of BrumiR, we applied it to discover mature miRNAs on simulated sRNA-seq reads from 10 animal and 10 plant species (Figure 2A). We compared BrumiR to the state-of-the-art genome-based miRNA discovery tools miRDeep2 [21] and miR-PREFeR [22], which were developed specifically for animal (miRDeep2) and plant (miR-PREFeR) species. For each tested species, we generated two synthetic datasets with different error-rates (0.01 and 0.02) using the miRsim tool implemented and provided by the BrumiR toolkit (https://github.com/camoragaq/miRsim), and the high-confidence miRNAs annotated in the miRBase database (see the Methods section). A total of 20 datasets with an average of 11.5 million reads were simulated. The list of simulated miRNAs was considered as the ground truth, and benchmark metrics (Figure 2C) were computed to assess the performance of BrumiR and of the other software (see the Methods section and Supplementary Table S1).

BrumiR recovered more mature miRNAs than the others, on average 92% (opposed to 41% and 64% for miRDeep2 and miR-PREFeR, respectively), and presented the highest average recall across all the simulated datasets (Figure 2B). BrumiR recovered more than 90% of the simulated mature miRNAs in 17 of the 20 simulated datasets (Figure 2B). In particular in the *H. sapiens*, *M. musculus* and *D. melanogaster* datasets, BrumiR recovered three times more candidates than miRDeep2 (Figure 2B). As concerns precision, BrumiR tended to generate more putative candidates than MiR-PREFeR (median 316 vs 264) and miRDeep2 (median 445 vs 308). The slightly higher number of BrumiR candidates resulted in lower average precision than miRDeep2 (0.59 vs 0.69) and MiR-PREFeR (0.63 vs 0.87). This is due to the fact that BrumiR does not use the hairpin structure filter employed by the other software. If we consider both precision and recall (F-Score), BrumiR was the top performer in 16 of the 20 datasets evaluated (Figure 2C). With animal species, BrumiR always reached a higher F-score than miRDeep2. With plant species, BrumiR was better or comparable to miR-PREFeR on most datasets, except for Z. mays and P. pattens where miR-PREFeR reached a higher F-Score (Figure 2C). In terms of computational time, BrumiR was the fastest method. In particular, BrumiR core was on average 30X faster than miRDeep2 and 10X times faster than MiR-PREFeR (Supplementary Table S3). The speed of BrumiR relies on efficient alignment-free and graph-based approaches.

Overall, we demonstrated with simulated data that BrumiR discovers putative mature miRNAs without a reference genome across different eukaryotic species achieving the highest accuracy and computational efficiency.

The hairpin structure of mature miRNAs is found in most of the **BrumiR** ***candidates.***

In order to assess the performance of BrumiR on real data, we collected public datasets for the same plant and animal species evaluated in the synthetic benchmark (Figure 2A). On average, 15.4 and 18.2 raw million reads were used for the animal and plant datasets (Supplementary Table S2), respectively. The predictions of BrumiR were compared against those of the state-of-the-art tools encompassing reference and *de novo* based methods [14, 21, 22]. In particular, we included mirnovo that similarly to BrumiR can discover mature miRNAs directly from the reads. Before running the tools, low-quality reads were removed using fastp [26] (~10%, see the Methods section). All the predicted miRNAs for each tool were annotated using the miRBase database to identify known and novel predictions. On average, BrumiR predicted ~1,000 putative mature miRNAs for the animal species, which was ~2.7X higher than the miRDeep2 candidates and 1.7X lower than the candidates predicted by mirnovo (Figure 3A1). For plant species, BrumiR predicted on average ~1,900 putative mature miRNAs, which was lower than the candidates predicted by mirR-PREFeR [3], and higher than the predictions of mirnovo (301 on average) (Figure 3A1). A comparison using the miRBase [13] annotated miRNAs revealed that BrumiR shared more candidates with miRDeep2 and miR-PREFeR than with mirnovo (Figure 3A2). However, an important fraction (on average more than 70%) of the miRBase-annotated candidates were exclusive to each tool (Figure 3A2), which summarizes the complexity of miRNA discovery.

Considering miRBase-annotated candidates as the ground-truth, we computed precision, recall and F-Score for all the evaluated tools (Figure 3B, Methods section). BrumiR achieved an accuracy (F-Score) better (animals) or comparable (plants) to the one

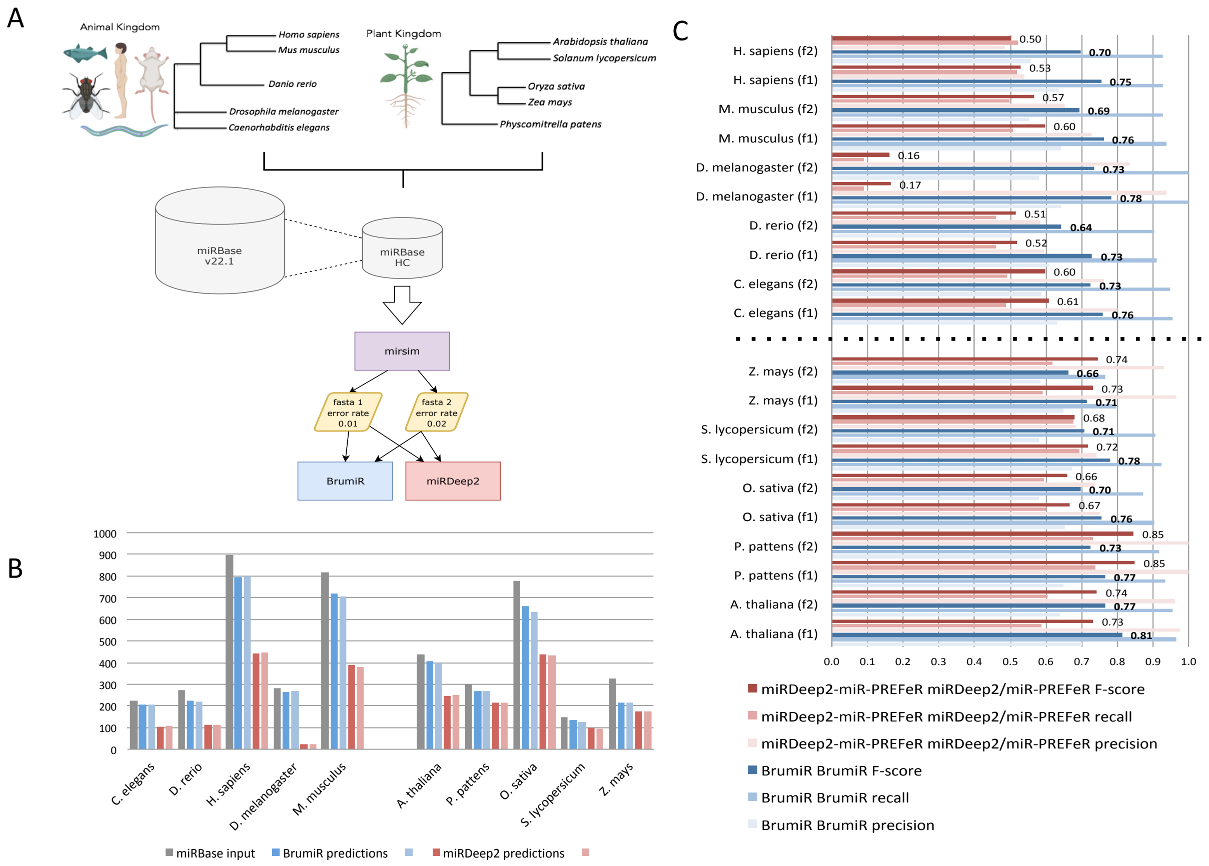

Figure 2. Synthetic benchmarking between BrumiR and miRDeep2. A) Workflow and species selected, B) miRBase input vs miRNA true positive predictions for each tool (2 samples), C) Benchmarking metrics for all datasets tested.

obtained by the other software (Figure 3B3). Moreover, BrumiR consistently reached the highest recall for most of the datasets evaluated (Figure 3B2). The precision values of BrumiR were slightly lower for some datasets (Figure 3B1), however, none of the methods performed well on this metric (Figure 3B3). In particular, miRDeep2 reached the highest precision (~0.7) on animal species and all methods performed poorly on the evaluated plant species (average precision <0.3). The low precision with plant species may be the product of a low number of entries annotated in miRBase for plants (10.414 vs 38.471 animals) as well as of a higher complexity of plant miRNAs [37]. The BrumiR toolkit also provides a tool to determine the hairpin loop of miRNA precursor sequences, which is the main structural feature of miRNAs [40]. BrumiR2reference maps the mature miRNAs predicted by BrumiR to the reference genome using an exhaustive alignment (see the Methods section), generates precursor sequences, computes their secondary structure, and checks the hairpin structure using a variety of criteria inferred from analyzing more than 30,000 miRBase precursor sequences from animal and plant species (see the Methods section). We used BrumiR2reference as a double validation for all the predicted mature miRNAs generated by BrumiR for the animal and plant datasets (Figure 3C). On average, BrumiR2reference identified a valid precursor sequence having the characteristic hairpin structure for over 70% of the BrumiR candidates (Figure 3C). In terms of speed, BrumiR core was the fastest tool. BrumiR was on average 120X and 220X times faster than miRDeep2 and miR-PREFeR, respectively (Supplementary Table S4).

Overall, we demonstrated that BrumiR is a competitive tool for discovering mature miRNAs without a reference genome. We showed that it was the most sensitive on most of the datasets tested. The performance of our method was not only faster, but also better or comparable to the state-of-the-art tools. Moreover, we also provide a new mapper approach to be used when a reference genome is available, to further verify if a precursor sequence of the predicted mature miRNA is present in the genome. BrumiR therefore represents a reliable alternative for the discovery of mature miRNAs in model and non-model species with or without a reference genome.

***Discovering novel miRNAs from sRNA-seq data of* A. thaliana *roots using* BrumiR.**

A. thaliana is one of the best characterized model organisms, and the first plant species in which miRNAs were cloned and sequenced [44]. To date, 436 mature miRNA sequences are included in the miRBase database. Most of these miRNAs have been identified by studies addressing the sRNAome of different plant organs [45], cell types [46], or responses to biotic or abiotic stress using sRNA-seq [47, 48].

We sequenced sRNA-seq libraries from the roots of *A. thaliana* after different time points during vegetative development (see the Methods section) (Supplementary Figure S8) to demonstrate the potential of BrumiR to discover novel mature miRNAs in a known biological context. BrumiR was run independently for each condition and replicate. The day 5 samples were excluded because of the low number of reads when compared to the other samples (Supplementary Table S5). BrumiR predicted, on average, 1,120 mature miRNAs per sample, which were further refined to 678 using the BrumiR2reference tool. To take advantage of our experimental design, we considered as a putative miRNA the ones present in the three replicates (core predictions) [49] (Figure 4A). Novel miRNAs were identified using the following steps: First, predictions were classified as known miRNAs by comparing with miRBase (160 known miRNAs out of a total of 436 miRNAs already described for *A. thaliana* in miRBase). These known miRNAs were put aside to explore the sensitivity of BrumiR in detecting novel putative miRNAs. We then clustered the remaining putative miRNAs into three stages: early, late, and constitutive (Figure 4B). The days 9, 13 and 17 represent an early stage of the plant development [50]; days 17, 21 and 25 represent a late stage of the plant development [50], and the putative miRNAs expressed in all conditions represent the constitutive category. A total of 25 putative novel miRNAs were identified, and a manual curation was carried out using all the information provided by BrumiR. Three curated novel miRNAs fulfilling all the recommended criteria to annotate miRNAs in plants [49] were discovered by BrumiR (Supplementary Figure S9, Supplementary Table S6). One of the curated miRNAs is located in chr1:29,612,248-29,612,361 (from now on denoted as miR-8) with a free energy of -40.5 and the

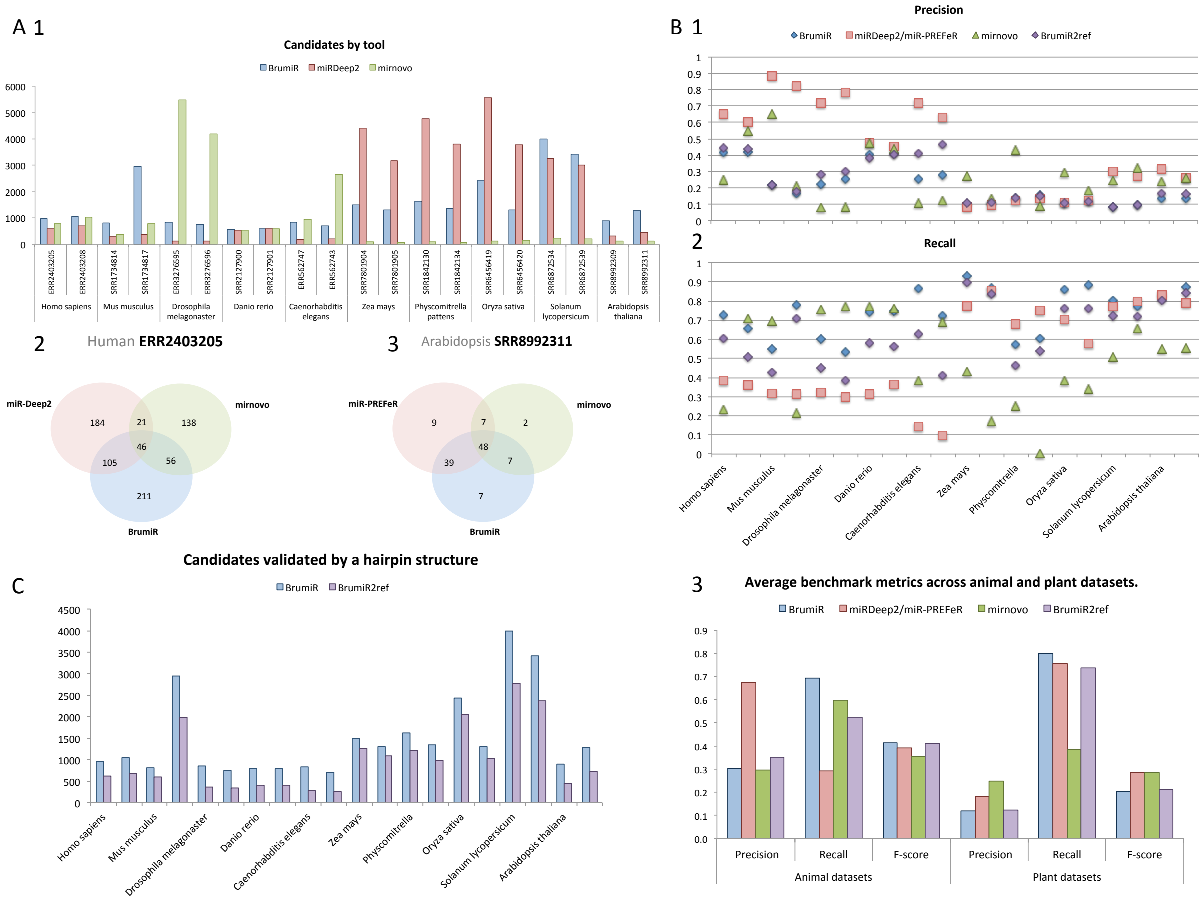

Figure 3. Real dataset benchmark of BrumiR and state-of-the-art tools. A) Number of predictions by tool for all the datasets and the overlap between them for 2 datasets (1 for animal and 1 for plant); B) Benchmarking metrics computed using miRBase annotated miRNAs, precision and recall for each dataset; and average metrics, including F-score. C) BrumiR candidates validated by Hairpin structure (BrumiR2Reference).

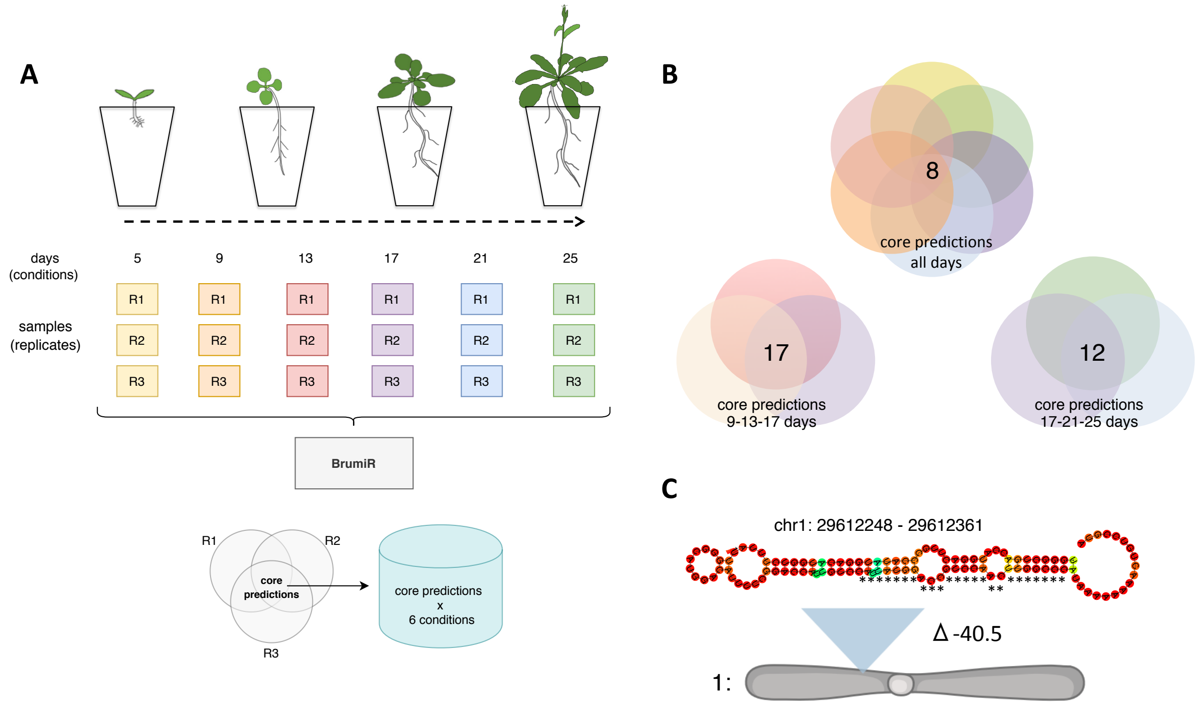

Figure 4. Applying BrumiR on sRNA-seq from *Arabidopsis* root libraries. A) Experimental design implemented; roots from *Arabidopsis* in a time-scale per day as conditions were sequenced in three technical replicates. BrumiR was used to analyze all sRNA-seq librairies, and conserved predictions by the three replicates was considered as a core by condition. B) Different combinations of root growth per day were analyzed together to identify novel putative miRNAs conserved in all conditions. C) miR-8 is discovered as a novel miRNA, supported by the hairpin analysis, and is conserved in all replicates in all conditions.

characteristic hairpin structure of plant miRNAs (Figure 4C). Interestingly, this miRNA locus has not been previously discovered because its mature sequence maps to multiple chromosomes, and is therefore discarded by genome-based tools [17].

In an exploratory analysis to shed light on the potential targets of these novel miRNAs, we conducted an in silico target transcript prediction using the psRNATarget algorithm [42] (Supplementary Table S7). FSD-1 (AT4G25100) was found to be one of the top genes regulated by this novel miRNA miR-8 (Supplementary Table S6). In *A. thaliana*, FSD-1 encodes a Fe superoxide dismutase enzyme which regulates reactive oxygen species (ROS) levels of chloroplast and cytosol and participates in salt stress tolerance [51]. Moreover, knockout mutants of FSD-1 exibit a lower number of lateral roots, thereby suggesting an important role in root development [52]. FSD-1 is developmentally regulated, abundantly expressed from the 3rd day to the 13th day but significantly decreased in the following days, and its differential accumulation between root zones is related to emerging patterns of lateral roots and hair formation from trichomes [53]. Another predicted mRNA target of miR-8 is PER24 (AT2G39040), which is a peroxidase gene involved in the detoxification of ROS in the extracellular and Na+ homeostasis and which plays an important role in the resistance to salinity stress as does the FSD-1 gene [51]. It is plausible to say that these novel miRNAs may be involved in the fine-tuning of lateral root growth in the early stages of development. These results highlight the value of the BrumiR toolkit for discovering novel miRNA candidates with functional impact on the organisms studied, even in the case where high quality genomes are available.

Discussion

In this paper, we introduced and benchmarked the BrumiR toolkit, which was designed for enabling the identification of mature miRNAs in model and non-model species with or without a reference genome, encompassing the plant and animal kingdoms. The BrumiR toolkit implements the following algorithms: 1) a new discovery miRNA tool (BrumiR-core), 2) a specific genome mapper (BrumiR2reference), and 3) a sRNA-seq read simulator (miRsim). We demonstrated that BrumiR is capable of identifying mature miRNAs based only on the sequence information, and generates results that are better or comparable to the state-of-the-art tools on simulated and real datasets. We further tested the usefulness of the BrumiR toolkit for discovering novel miRNAs potentially involved in the regulation of the root development of the extensively annotated *A. thaliana* genome.

Unlike the state-of-the-art tools, BrumiR starts by encoding the sRNA-seq reads using a de Bruijn graph. This avoids the read mapping stage and the dependency on previous miRNA annotations. It also enables the identification of sequencing artifacts. A critical step of genome-based miRNA discovery tools is to identify the precursor sequence when a reference genome is available. BrumiR introduces a new mapping approach, BrumiR2reference, which scans every possible hairpin precursor in the genome, when such is available, for all the BrumiR predictions. As the hairpin structure is determined using the predicted mature miRNA instead of the reads, this alignment can support mismatches and indels and handles the case of multi-mapped candidates (due to repetitive regions of the genome). Such features distinguish BrumiR from the current genome-based methods.

Discovering miRNAs in non-model species is one of the limitations of the current methods. One exception is mirnovo, which similarly to BrumiR can predict miRNAs using only the sRNA-seq data, and a specific training set for animal and plant species. We thus compared its performance to the one of BrumiR. Our results show that mirnovo is very conservative, generating few predictions but with a high accuracy. BrumiR generates a larger number of candidates than mirnovo, most of which are annotated in miRBase but also others that correspond to new candidates. However, the higher number of predictions of BrumiR results in a lower accuracy in some of the datasets evaluated, representing a potential weakness of our method. To address this, we developed the BrumiR2reference tool to refine the BrumiR predictions, thus reducing the number of false-positive putative miRNAs. We plan to further reduce the false-positive rate of BrumiR by using a random-forest classifier trained on the high confidence mature sequences available in miRBase. The latter will improve the accuracy of BrumiR even in the case when a reference genome is not available. It is important to observe that the miRNA annotations remain incomplete and although miRBase is the main repository for miRNAs, it cannot be considered as the gold-standard for most species [9]. For this reason, the predictions of BrumiR are not based on miRBase in any step of the algorithm. However, miRBase can be used with all due precaution in a posterior analysis to verify the miRNAs inferred in case of not having any reference genome.

In terms of computational resources and usability, BrumiR is the fastest method and provides a stand-alone package for running locally all the analyses. It further generates an output that is compatible with the Bandage software [43], which can be employed to visualize and explore the results of BrumiR in a user-friendly way. Moreover, BrumiR reports other sequences expressed in the sRNA-seq data among which are putative isomiRs and longer non-coding RNAs, thereby providing additional biological insight. Finally, we tested the effectiveness of BrumiR on sequenced sRNA-seq libraries from the roots of *A. thaliana*, and were able to annotate 3 novel putative miRNAs based on the very conservative criteria proposed in [49], showing the potential of it being used alone or in combination with other methods.

In summary, we present a new and versatile method that implements novel algorithmic ideas for the study of miRNAs that complements and extends the currently existing approaches.

Code Availability

The BrumiR code (v1.0) used in this manuscript is freely available at https://github.com/camoragaq/BrumiR, and is open software under the MIT license.

Funding

This work was supported by CONICYT BECAS CHILE DOCTORADO 2016/FOLIO 72170320 granted to CM, by a post-doctorate fellowship from the Agence National de Recherche (ANRGREEN 17_CE20_0031_01) granted to MGF, as well as by Fondo Nacional de Desarrollo Científico y Tecnológico (FONDECYT)-ANID grant 1170926, ANID PCI-Redes Internacionales entre Centros de Investigación grant REDES180097 and Instituto Milenio iBio - Iniciativa Científica Milenio MINECON granted to E.A.V. This research was performed using the computing facilities of the LBBE/PRABI and the France Génomique e-infrastructure (ANR-10-INBS-09-08).

Author Contributions

CM designed, developed, implemented and benchmarked BrumiR. MFS guided the development of BrumiR. ES conducted the *A. thaliana* experiments. EAV designed and supervised the *A. thaliana* experiments. CM wrote the initial version of the manuscript with inputs from all other authors. MFS and MGF helped to improve the manuscript. EAV, MFS, MGF and RAG provided crucial biological feedback. All authors provided helpful discussions for the work and reviewed the manuscript.

Conflict of interest statement.

None declared.

References

1. Bartel DP. MicroRNAs: Genomics, Biogenesis, Mechanism, and Function. Cell 2004 Jan;116(2):281–297.
2. Bartel DP. MicroRNAs: target recognition and regulatory functions. cell 2009;136(2):215–233.
3. Peng Y, Croce CM. The role of MicroRNAs in human cancer. Signal Transduction and Targeted Therapy 2016 Jan;1:15004.
4. Greene J, Baird AM, Brady L, Lim M, Gray SG, McDermott R, et al. Circular RNAs: Biogenesis, Function and Role in Human Diseases. Frontiers in Molecular Biosciences 2017;4:38.
5. Lin R, He L, He J, Qin P, Wang Y, Deng Q, et al. Comprehensive analysis of microRNA-Seq and target mRNAs of rice sheath blight pathogen provides new insights into pathogenic regulatory mechanisms. DNA Research 2016 Oct;23(5):415–425.
6. Wang M, Weiberg A, Lin FM, Thomma BPHJ, Huang HD, Jin H. Bidirectional cross-kingdom RNAi and fungal uptake of external RNAs confer plant protection. Nature Plants 2016 Oct;2(10):16151.
7. Lau NC, Lim LP, Weinstein EG, Bartel DP. An Abundant Class of Tiny RNAs with Probable Regulatory Roles in *Caenorhabditis elegans*. Science 2001 Oct;294(5543):858–862.
8. Lagos-Quintana M, Rauhut R, Lendeckel W, Tuschl T. Identification of novel genes coding for small expressed RNAs. Science (New York, NY) 2001 Oct;294(5543):853–858.
9. Bortolomeazzi M, Gaffo E, Bortoluzzi S. A survey of software tools for microRNA discovery and characterization using RNA-seq. Briefings in Bioinformatics 2019;20(3):918–930.

10. Pinzón N, Li B, Martinez L, Sergeeva A, Presumey J, Apparailly F, et al. microRNA target prediction programs predict many false positives. Genome research 2017;27(2):234–245.
11. Morin RD, O'Connor MD, Griffith M, Kuchenbauer F, Delaney A, Prabhu AL, et al. Application of massively parallel sequencing to microRNA profiling and discovery in human embryonic stem cells. Genome research 2008;18(4):610–621.
12. Chen L, Heikkinen L, Wang C, Yang Y, Sun H, Wong G. Trends in the development of miRNA bioinformatics tools. Briefings in Bioinformatics 2019;20(5):1836–1852.
13. Kozomara A, Griffiths-Jones S. miRBase: annotating high confidence microRNAs using deep sequencing data. Nucleic Acids Research 2014 Jan;42(Database issue):D68–D73.
14. Vitsios DM, Kentepozidou E, Quintais L, Benito-Gutiérrez E, van Dongen S, Davis MP, et al. Mirnovo: genome-free prediction of microRNAs from small RNA sequencing data and single-cells using decision forests. Nucleic Acids Research 2017 Dec;45(21):e177–e177.
15. Langmead B, Trapnell C, Pop M, Salzberg SL. Ultrafast and memory-efficient alignment of short DNA sequences to the human genome. Genome Biology 2009 Mar;10(3):R25.
16. Li H, Durbin R. Fast and accurate short read alignment with Burrows-Wheeler transform. Bioinformatics 2009 Jul;25(14):1754–1760.
17. Ziemann M, Kaspi A, El-Osta A. Evaluation of microRNA alignment techniques. RNA 2016 Aug;22(8):1120–1138.
18. Li Y, Zhang Z, Liu F, Vongsangnak W, Jing Q, Shen B. Performance comparison and evaluation of software tools for microRNA deep-sequencing data analysis. Nucleic acids research 2012;40(10):4298–4305.
19. A reference standard for genome biology. Nature Biotechnology 2018 Dec;36(12):1121.
20. Friedländer MR, Chen W, Adamidi C, Maaskola J, Einspanier R, Knespel S, et al. Discovering microRNAs from deep sequencing data using miRDeep. Nature biotechnology 2008;26(4):407–415.
21. Friedländer MR, Mackowiak SD, Li N, Chen W, Rajewsky N. miRDeep2 accurately identifies known and hundreds of novel microRNA genes in seven animal clades. Nucleic Acids Research 2012 Jan;40(1):37–52.
22. Lei J, Sun Y. miR-PREFeR: an accurate, fast and easy-to-use plant miRNA prediction tool using small RNA-Seq data. Bioinformatics (Oxford, England) 2014 Oct;30(19):2837–2839.
23. Jha A, Shankar R. miReader: Discovering Novel miRNAs in Species without Sequenced Genome. PLOS ONE 2013 Jun;8(6):e66857.
24. Mapleson D, Moxon S, Dalmay T, Moulton V. MirPlex: a tool for identifying miRNAs in high-throughput sRNA datasets without a genome. Journal of Experimental Zoology Part B, Molecular and Developmental Evolution 2013 Jan;320(1):47–56.
25. Compeau PE, Pevzner PA, Tesler G. Why are de Bruijn graphs useful for genome assembly? Nature biotechnology 2011;29(11):987.
26. Chen S, Zhou Y, Chen Y, Gu J. fastp: an ultra-fast all-in-one FASTQ preprocessor. Bioinformatics 2018 Sep;34(17):i884–i890. Publisher: Oxford Academic.
27. Chikhi R, Limasset A, Medvedev P. Compacting de Bruijn graphs from sequencing data quickly and in low memory. Bioinformatics 2016 Jun;32(12):i201–i208.
28. Durai DA, Schulz MH. Informed kmer selection for *de novo* transcriptome assembly. Bioinformatics 2016 Jun;32(11):1670–1677.
29. Chikhi R, Rizk G. Space-efficient and exact de Bruijn graph representation based on a Bloom filter. Algorithms for Molecular Biology 2013;8(1):22.
30. Deorowicz S, Debudaj-Grabysz A, Grabowski S. Disk-based k-mer counting on a PC. BMC Bioinformatics 2013 May;14(1):160.
31. Lewis HR, Papadimitriou CH. Symmetric space-bounded computation. Theoretical Computer Science 1982 Aug;19(2):161–187.
32. Šošić M, Šikić M. Edlib: a C/C ++ library for fast, exact sequence alignment using edit distance. Bioinformatics 2017

May;33(9):1394–1395.

33. Lambert M, Benmoussa A, Provost P. Small Non-Coding RNAs Derived from Eukaryotic Ribosomal RNA. Non-Coding RNA 2019 Mar;5(1):16.

34. Kalvari I, Argasinska J, Quinones-Olvera N, Nawrocki EP, Rivas E, Eddy SR, et al. Rfam 13.0: shifting to a genome-centric resource for non-coding RNA families. Nucleic Acids Research 2018 Jan;46(D1):D335–D342.

35. Kalvari I, Nawrocki EP, Argasinska J, Quinones-Olvera N, Finn RD, Bateman A, et al. Non-Coding RNA Analysis Using the Rfam Database. Current Protocols in Bioinformatics 2018;62(1):e51.

36. Kokot M, Dlugosz M, Deorowicz S. KMC 3: counting and manipulating k-mer statistics. Bioinformatics (Oxford, England) 2017 Sep;33(17):2759–2761.

37. Meyers BC, Axtell MJ, Bartel B, Bartel DP, Baulcombe D, Bowman JL, et al. Criteria for Annotation of Plant MicroRNAs. The Plant Cell 2008 Dec;20(12):3186–3190.

38. Higashi S, Fournier C, Gautier C, Gaspin C, Sagot MF. Mirinho: An efficient and general plant and animal pre-miRNA predictor for genomic and deep sequencing data. BMC Bioinformatics 2015 May;16(1):179.

39. Lorenz R, Bernhart SH, Höner zu Siederdissen C, Tafer H, Flamm C, Stadler PF, et al. ViennaRNA Package 2.0. Algorithms for Molecular Biology 2011 Nov;6(1):26.

40. Roden C, Gaillard J, Kanoria S, Rennie W, Barish S, Cheng J, et al. Novel determinants of mammalian primary microRNA processing revealed by systematic evaluation of hairpin-containing transcripts and human genetic variation. Genome Research 2017 Mar;27(3):374–384.

41. Danaee P, Rouches M, Wiley M, Deng D, Huang L, Hendrix D. bpRNA: large-scale automated annotation and analysis of RNA secondary structure. Nucleic Acids Research 2018 Jun;46(11):5381–5394.

42. Dai X, Zhuang Z, Zhao PX. psRNATarget: a plant small RNA target analysis server (2017 release). Nucleic Acids Research 2018 Jul;46(W1):W49–W54.

43. Wick RR, Schultz MB, Zobel J, Holt KE. Bandage: interactive visualization of *de novo* genome assemblies. Bioinformatics 2015 Oct;31(20):3350–3352.

44. Reinhart BJ, Weinstein EG, Rhoades MW, Bartel B, Bartel DP. MicroRNAs in plants. Genes & Development 2002 Jul;16(13):1616–1626.

45. Fahlgren N, Howell MD, Kasschau KD, Chapman EJ, Sullivan CM, Cumbie JS, et al. High-Throughput Sequencing of *Arabidopsis* microRNAs: Evidence for Frequent Birth and Death of MIRNA Genes. PLOS ONE 2007 Feb;2(2):e219.

46. Breakfield NW, Corcoran DL, Petricka JJ, Shen J, Sae-Seaw J, Rubio-Somoza I, et al. High-resolution experimental and computational profiling of tissue-specific known and novel miRNAs in *Arabidopsis*. Genome Research 2011 Sep;.

47. Hsieh LC, Lin SI, Shih ACC, Chen JW, Lin WY, Tseng CY, et al. Uncovering small RNA-mediated responses to phosphate deficiency in *Arabidopsis* by deep sequencing. Plant Physiology 2009 Dec;151(4):2120–2132.

48. Moldovan D, Spriggs A, Yang J, Pogson BJ, Dennis ES, Wilson IW. Hypoxia-responsive microRNAs and trans-acting small interfering RNAs in *Arabidopsis*. Journal of Experimental Botany 2010 Jan;61(1):165–177.

49. Axtell MJ, Meyers BC. Revisiting Criteria for Plant MicroRNA Annotation in the Era of Big Data. The Plant Cell 2018 Feb;30(2):272–284.

50. Satbhai SB, Ristova D, Busch W. Underground tuning: quantitative regulation of root growth. Journal of Experimental Botany 2015 Feb;66(4):1099–1112.

51. Guan Q, Wu J, Yue X, Zhang Y, Zhu J. A nuclear calcium-sensing pathway is critical for gene regulation and salt stress tolerance in *Arabidopsis*. PLoS Genet 2013;9(8):e1003755.

52. Kuo WY, Huang CH, Liu AC, Cheng CP, Li SH, Chang WC, et al. Chaperonin 20 mediates iron superoxide dismutase (FeSOD)

activity independent of its co-chaperonin role in *Arabidopsis* chloroplasts. The New Phytologist 2013 Jan;197(1):99–110.

53. Dvořák P, Krasylenko Y, Ovečka M, Basheer J, Zapletalová V, Šamaj J, et al. FSD1: developmentally-regulated plastidial, nuclear and cytoplasmic enzyme with anti-oxidative and osmoprotective role. Plant, Cell & Environment 2020 Apr;.

Manuscript revised version

Click here to access/download;Manuscript;BrumiR-revised-version.pdf

BrumiR: A toolkit for *de novo* discovery of microRNAs from sRNA-seq data.

Carol Moraga[1,2,*], Evelyn Sanchez[3,4], Mariana Galvão Ferrarini[1,5], Rodrigo A. Gutierrez[4,6,7], Elena A. Vidal[3,4,8], Marie-France Sagot[1,2,*]

[1]Université de Lyon, Université Lyon 1, CNRS, Laboratoire de Biométrie et Biologie Evolutive UMR 5558, F-69622 Villeurbanne, France. Erable Team, Inria Grenoble Rhône-Alpes, 38334 Montbonnot, France. [3]Centro de Genómica y Bioinformática, Facultad de Ciencias, Universidad Mayor, Chile. [4]Millennium Institute for Integrative Biology iBio, Chile. [5]University of Lyon, INSA-Lyon, INRA, BF2i, UMR0203, F-69621 Villeurbanne, France. [6]Departamento de Genetica Molecular y Microbiologia, Facultad de Ciencias Biologicas, Pontificia Universidad Católica de Chile. [7]FONDAP Center for Genome Regulation. [8]Escuela de Biotecnología, Facultad de Ciencias, Universidad Mayor.

To whom correspondence should be addressed: Carol Moraga - camoragaq@gmail.com, Marie-France Sagot – marie-france.sagot@inria.fr

Abstract

MicroRNAs (miRNAs) are small non-coding RNAs that are key players in the regulation of gene expression. In the last decade, with the increasing accessibility of high-throughput sequencing technologies, different methods have been developed to identify miRNAs, most of which rely on pre-existing reference genomes. However, when a reference genome is absent or is not of high quality, such identification becomes more difficult. In this context, we

developed BrumiR, an algorithm that is able to discover miRNAs directly and exclusively from sRNA-seq data. We benchmarked BrumiR with datasets encompassing animal and plant species using real and simulated sRNA-seq experiments. The results demonstrate that BrumiR reaches the highest recall for miRNA discovery, while at the same time being much faster and more efficient than the state-of-the-art tools evaluated. The latter allows BrumiR to analyze a large number of sRNA-seq experiments, from plants or animal species. Moreover, BrumiR detects additional information regarding other expressed sequences (sRNAs, isomiRs, etc.), thus maximizing the biological insight gained from sRNA-seq experiments. Finally, when a reference genome is available, BrumiR provides a new mapping tool (BrumiR2ref) that performs an *a posteriori* exhaustive search to identify the precursor sequences. The code of BrumiR is freely available at https://github.com/camoragaq/BrumiR.

Introduction

MicroRNAs (henceforth denoted by miRNAs) are small RNA molecules usually shorter than 25 nucleotides (nt), which have been identified as crucial regulators of gene expression mostly at the post-transcriptional level (Bartel, 2004). miRNAs are involved in a wide range of biological processes including cell cycle, differentiation, apoptosis and disease (Bartel, 2009). They have been the target molecules for a large number of important applications, more particularly in cancer where miRNAs have been shown to play important roles in driving or suppressing tumor spread (Greene et al., 2017; Peng & Croce, 2016). In plant

species, unravelling host-pathogen interactions mediated by miRNAs may shed light on plant development and its relation with the environment, both essential knowledge that can lead to the discovery of new biotechnological products for the agricultural industry (Lin et al., 2016; Wang et al., 2016).

Since the first classification and annotation of miRNAs in C.elegans (Lagos-Quintana et al., 2001; Lau et al., 2001), thousands of miRNAs have been discovered in plants, animals and other eukaryotes. Most eukaryotic miRNAs are transcribed by RNA polymerase II (Cai et al., 2004; Lee et al., 2002, 2004), while some of them are transcribed by RNA polymerase III in animals (Borchert et al., 2006). Long precursor RNAs are folded into hairpin-like structures consisting of a terminal loop, an upper stem, the miRNA duplex region, a lower stem and two arms, and are processed in the cytoplasm generating the miRNA/miRNA* duplex which is subsequently divided into the star and the functional mature miRNA sequence (Lee et al., 2003). Mature miRNA processing pathways differ between animals and plants. One major difference is the length of the precursor sequences, with plant precursors longer than those of animals (Meyers et al., 2008). The mature miRNA sequences act as guides leading the RISC complex to target RNAs to regulate their expression by transcript cleavage or translation inhibition (Khvorova et al., 2003; Schwarz et al., 2003). Therefore, accurate prediction of known and novel miRNAs along with their targets is essential for increasing our understanding of the miRNA biology (Bartel, 2018; Peng & Croce, 2016). However, it has proven difficult to accurately characterize and predict the miRNAs as well as their regulatory networks (Bortolomeazzi et al., 2019; Pinzón et al., 2017).

Nowadays, a common experimental practice is to identify miRNAs and their expression patterns using next generation sequencing technologies (NGS) (Morin et al., 2008). Commonly, NGS experiments are able to generate more than 20 million sRNA-seq reads, thus promoting the development of algorithms to transform and process such data into biological information (L. Chen et al., 2019).

Currently, there are two computational strategies for the discovery of miRNAs: 1) genome-based approaches that rely on the mapping of the sRNA-seq reads to a reference genome and subsequent evaluation of the sequences generating the characteristic hairpin structure of miRNA precursors (Bortolomeazzi et al., 2019); 2) machine-learning approaches which rely on the biogenesis features extracted from the knowledge on miRNA sequences available in databases such as miRBase (Kozomara & Griffiths-Jones, 2014) and on the analysis of the duplex structure of miRNAs (Vitsios et al., 2017). Genome-based methods, that have been updated at the pace of the evolving NGS technologies, are the most widely used tools in this field, and their results have populated the public miRNA repositories (L. Chen et al., 2019). Such methods are the natural choice for the study of model species with high quality reference genomes available. However, it has been shown that most of the genome-based tools struggle with a high rate of false positive predictions when they rely only on the reference genome and do not leverage on sRNAseq data (Bortolomeazzi et al., 2019). Additionally, a critical step of such tools is the use of genome aligners (Langmead et al., 2009; H. Li & Durbin, 2009) to map the sRNA-seq reads to the reference genome. Mapping short (<30 nt) and very similar sequences to a

large, complex, and repetitive reference genome is however a difficult and error-prone task (Ziemann et al., 2016). Genome-based methods are thus highly sensitive to the aligner selected as well as to the parameters employed and the thresholds chosen (*e.g.* number of mismatches allowed) in order to discard mapping artefacts generated from sequencing errors (Y. Li et al., 2012). Furthermore, despite all the advancements in the sequencing technologies and *de novo* assembly methods, few complete genomes are available today, which is a recurring problem that researchers working on non-model species face ("A Reference Standard for Genome Biology," 2018). The lack of a high quality reference genome thus reduces the possibilities for discovering novel miRNAs (Vitsios et al., 2017). Genome-based methods such as miRDeep (Friedländer et al., 2008), miRDeep2 (Friedländer et al., 2012), and miR-PREFeR (Lei & Sun, 2014) are included in this group.

On the other hand, new methods such as miReader (Jha & Shankar, 2013), MirPlex (Mapleson et al., 2013), and mirnovo (Vitsios et al., 2017), in particular using machine-learning approaches, were specifically developed as an alternative to discover miRNAs in species without a reference genome. In the case of mirnovo, the initial step involves the clustering of the sRNA-seq reads performing an all-vs-all read comparison that is followed by a subsequent classification of the clusters into putative miRNAs using pre-trained models. The performance obtained by such methods on well-annotated species is comparable to those achieved by genome-based methods (Bortolomeazzi et al., 2019). However, relying exclusively on annotated miRNAs for training machine learning models may introduce a bias towards the identification of

well-characterized miRNAs over species-specific ones (L. Chen et al., 2019). Nonetheless, machine learning methods have demonstrated that it is possible to discover miRNAs using only the sequence information present in the sRNA-seq experiment (Vitsios et al., 2017).

There remains however a need to go further in the development of algorithms for finding novel miRNAs in non-model species using only the sequence information. With this purpose in mind, the adoption of a special type of graphs called *de Bruijn* graphs may be considered. This is a widely used approach for the *de novo* reconstruction of genome or transcriptome sequences (Compeau et al., 2011). It therefore appears to be a plausible option for organizing, clustering and assembling the sequence information present in sRNA-seq experiments. However, accommodating the de Bruijn graph approach for the discovery of miRNAs involves the development of new methods to address the specific characteristics of sRNA-seq data. Indeed, mature miRNA sequences are short (18-24 nt), thus limiting the overlap length for building a de Bruijn graph which in turn impacts the global topology by inducing tangled graph structures. Moreover, miRNAs captured in a sRNA-seq experiment have variable expression, from low (few reads) to highly expressed (thousands of reads), which may induce spurious graph connections that should be removed in order to isolate and detect both types of miRNAs. Finally, the sequencing errors present in sRNA-seq data further induce spurious connections and are harder to detect as compared to genomic data due to the variable expression and the shorter lengths of the miRNAs. Overall, using a de Bruijn graph to analyze sRNA-seq data and extract information from such data seems thus

counterintuitive as mature miRNAs are captured full-length by the current NGS technologies. However, a de Bruijn graph has several interesting properties for the discovery of miRNAs, mainly due to the fact that it encodes all the sRNA-seq sequence information at once in a compact and connected representation (graph), without the need to perform an all-vs-all read comparison or mapping to a reference.

In this paper, we present BrumiR, a *de novo* algorithm based on a de Bruijn graph approach that is able to identify miRNAs directly and exclusively from sRNA-seq data. Unlike other state-of-the-art algorithms, BrumiR does not rely on a reference genome, on the availability of close phylogenetic species, or on conserved sequence information. Instead, BrumiR starts from a de Bruijn graph encoding all the reads and is able to directly identify putative miRNAs on the generated graph. BrumiR also removes sequencing errors and navigates inside the graph detecting putative miRNAs by considering several miRNA biogenesis properties (such as expression, length, topology in the graph). Along with miRNA discovery, BrumiR can also assemble and identify other types of small and long non-coding RNAs expressed within the sequencing data. Finally, when a reference genome is available, BrumiR provides a new mapping tool (BrumiR2ref) that performs an exhaustive search to identify and validate the precursor sequences.

We extensively benchmarked BrumiR on animal and plant species using simulated and real datasets. The benchmark results demonstrate that BrumiR is very sensitive, besides being the fastest tool, and its predictions were supported by the characteristic hairpin structure of miRNAs. Finally, we also

applied BrumiR to the discovery of miRNAs of *Arabidopsis thaliana* and identified three novel high-confidence miRNAs involved in root development. These putative miRNAs were not discovered before by any other software, thereby showing the potential of using different approaches even in the case where high quality genomes are available. The code of BrumiR is freely available at https://github.com/camoragaq/BrumiR.

RESULTS

BrumiR discovers mature miRNAs directly from the sRNA-seq reads.

The main idea behind BrumiR is that mature miRNAs can be discovered directly from the information contained in the sequenced sRNA-seq reads. To achieve this, BrumiR starts by building a de Bruijn graph (using *k*-mers of size 14) from the sRNA-seq reads, then compacting all the simple nodes thus leading to the unipath graph (Chikhi et al., 2016) (Figure 1.1, Methods section). The unipath graph encodes all the sequence information of the sRNA-seq experiment, including sequencing errors, adapters, and other types of sequences (Figure 1.1). The construction of the unipath graph allows to avoid entirely the alignment of the sRNA-seq reads to a reference genome. Following the unipath graph construction, BrumiR cleans the graph by removing tips (dead-end nodes) with low expression/abundance (KM < 5), which are usually generated from sequencing errors (Figure 1.2). One feature of the miRNA biogenesis is

that after Dicer cleavage, the mature miRNA is the most abundant of the three by-products and when it is sequenced, it has a uniform expression along its sequence (Friedländer et al., 2008). Therefore, BrumiR expects that the neighbor elements within a particular putative miRNA will have similar expression. BrumiR checks all neighbor connections (arcs), and deletes any connection with a relative expression difference larger than 3 fold (Figure 1.3, Methods section), and the new graph is cleaned again by removing tips (Figure 1.4). Clusters of unipaths (connected components) with topologies related to sequencing errors are also removed (Figure 1.5, Methods section).

BrumiR attempts to re-assemble all unipaths within a connected component (CC) of the graph, and those with between 18 and 24 nt are classified as putative miRNAs, while longer re-assembled unipaths (>24 nt) are classified as other longer sequences (Figure 1.6). BrumiR then restores missing connections by re-clustering the putative miRNAs performing an all-vs-all comparison. The most expressed miRNA is selected as the representative of the cluster (Figure 1.7) and the remaining members are classified as potential isomiRs (Figure 1.7). The final BrumiR step uses the RFAM database (Kalvari, Argasinska, et al., 2018) to discard predicted miRNAs matching to other classes of RNA (*e.g.* Ribosomal genes, Figure 1.8). We build a 16-mer database using RFAM database excluding any reference to known miRNA sequences, in a similar way as mirnovo does (Vitsios et al., 2017). As an example, BrumiR reduces the input sRNA-seq data by five orders of magnitude generating less than 1,000 putative mature miRNAs (24 million input reads to 966 miRNA candidates, see Figure 1.10). Finally, BrumiR outputs several FASTA files with all predicted mature

miRNAs, all longer RNAs, putative isomiRs, other sRNAs (RFAM comparison), and a table with expression values for each predicted miRNA. Additionally, BrumiR outputs the final graph in GFA format, which can be explored using Bandage (Wick et al., 2015) (Figure S11).

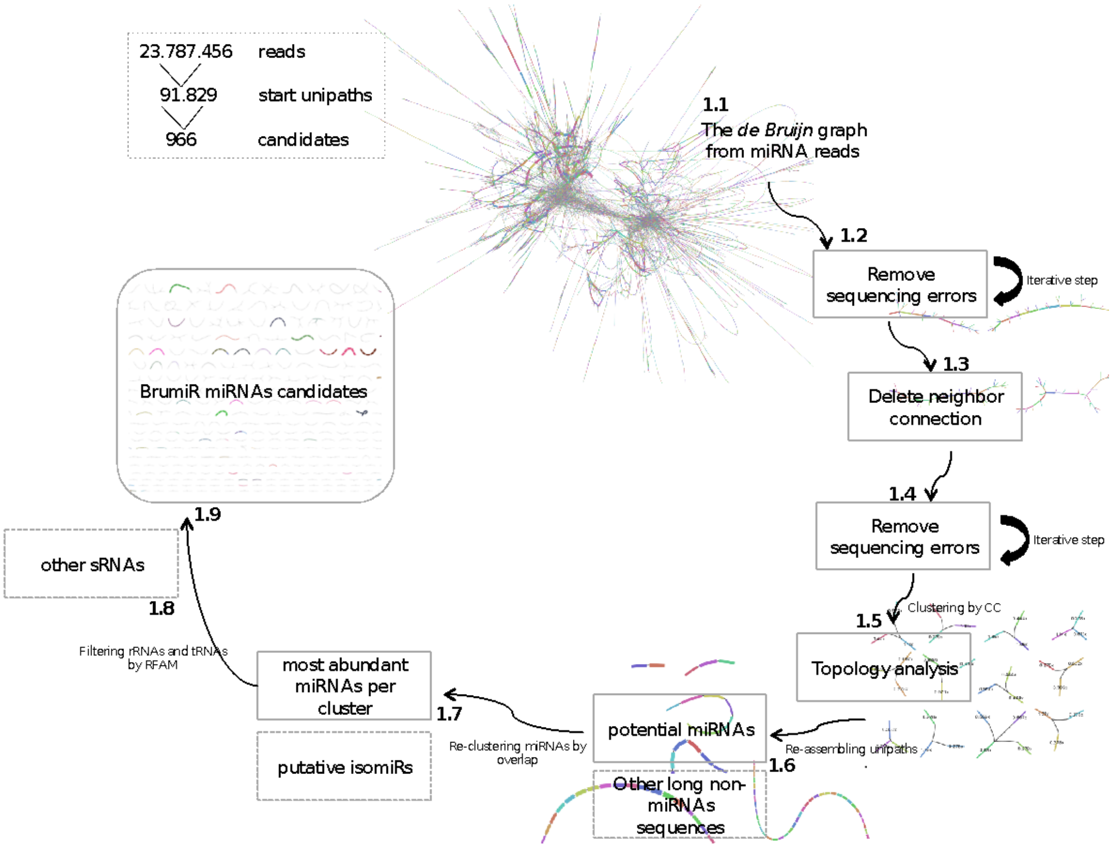

Figure 1. **BrumiR algorithm**. Different steps of BrumiR to discover miRNAs from sRNA-seq data. **1.1** De Bruijn graph step, **1.2** Tips removal iterative step, **1.3** Delete neigbor connection step, **1.4** Tips removal step repetition, **1.5** Topology analysis step, **1.6** Re-assembling unipaths by CC step, **1.7** Re-clustering by overlap step, **1.8** Filtering other sRNAs by RFAM step, **1.9** BrumiR candidates catalog.

BrumiR achieves the highest accuracy on simulated data.

To evaluate the performance of BrumiR, we applied it to discover mature miRNAs on simulated sRNA-seq reads from 10 animal and 10 plant species (Figure 2A). We compared BrumiR to the state-of-the-art genome-based miRNA discovery tools miRDeep2 (Friedländer et al., 2012) and miR-PREFeR (Lei & Sun, 2014), which were developed specifically for animal (miRDeep2) and plant (miR-PREFeR) species. For each tested species, we generated two synthetic datasets with different error-rates (0.01 and 0.02) using the miRsim tool implemented and provided by the BrumiR toolkit (https://github.com/camoragaq/miRsim). To simulate the reads, we used (i) the high-confidence miRNAs annotated in the miRBase database (Kozomara & Griffiths-Jones, 2014), (ii) sequences from the RFAM database (v14.1) (Kalvari, Nawrocki, et al., 2018) to simulate possible fragments from other known types of RNAs present in the sRNA-seq data, and (iii) random genomic sequences for each of the species included in the benchmark (see the Methods section). A total of 20 datasets with an average of 13.6 million reads were simulated. The list of simulated miRNAs was considered as the ground truth, and benchmark metrics (Figure 2C) were computed to assess the performance of BrumiR and of the other software (See Methods section) (Supplementary Table S2).

BrumiR recovered more mature miRNAs than the others, on average 97% (opposed to 58% and 66% for miRDeep2 and miR-PREFeR, respectively), and presented the highest average recall across all the simulated datasets (Figure

2B). BrumiR recovered more than 90% of the simulated mature miRNAs in 19 of the 20 simulated datasets (Figure 2B). In particular in the *H. sapiens* and *D. melanogaster* datasets, BrumiR recovered 1,5X and 2,5X more candidates than MiRDeep2 (Figure 2B). As concerns precision, BrumiR tended to generate more putative candidates than MiRDeep2 (median 659 vs 332) and less than MiR-PREFeR (median 474 vs 649). The slightly higher number of BrumiR candidates resulted in lower average precision than miRDeep2 for animal species (0.51 vs 0.65), but was significally higher as compared to MiR-PREFeR for plants (0.71 vs 0.43). The lower precision achieved in animal species might be due to the fact that BrumiR does not use the hairpin structure filter employed by miRDeep2. If we consider both precision and recall (F-Score), BrumiR was the top performer in 17 of the 20 datasets evaluated (Figure 2C). With animal species, BrumiR always reached a higher F-score than miRDeep2 except for *M. musculus*. With plant species, BrumiR was better to miR-PREFeR on most datasets, BrumiR reached a higher F-Score in 9 of the 10 datasets (Figure 2C).

In terms of computational time, BrumiR was the fastest method. In particular, BrumiR core was on average 21X faster than miRDeep2 and 6X times faster than MiR-PREFeR (see Table S3). The speed of BrumiR relies on efficient alignment-free and graph-based approaches.

Overall, we demonstrated with simulated data that BrumiR discovers putative mature miRNAs without a reference genome across different eukaryotic species achieving the highest accuracy and computational efficiency.

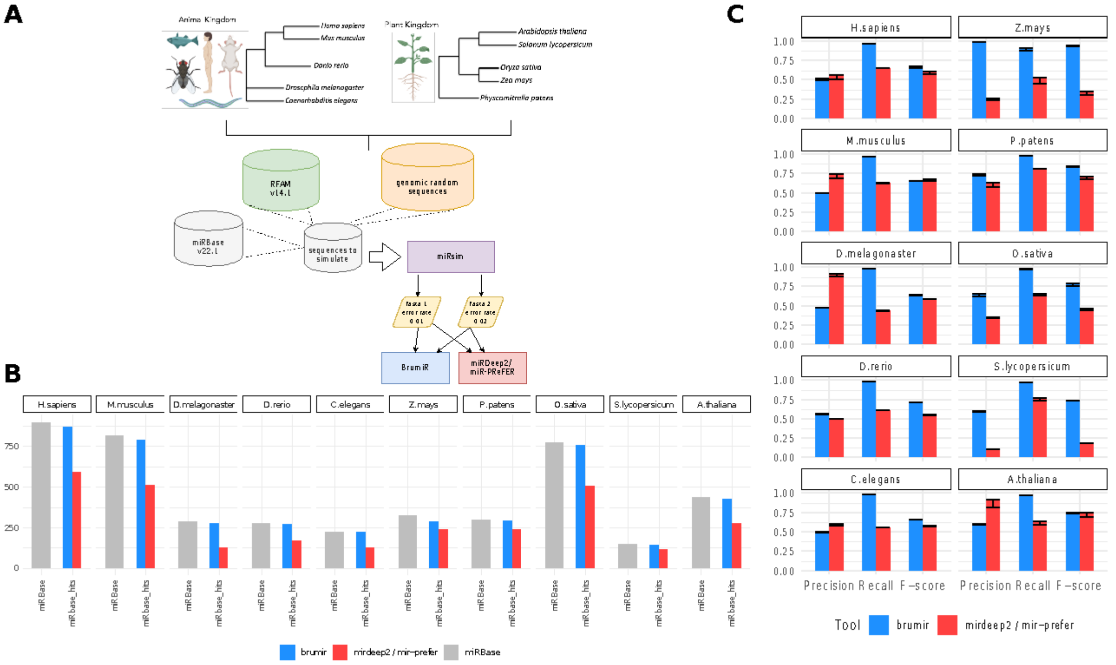

Figure 2. Synthetic benchmarking between BrumiR and miRDeep2. A) Workflow and species selected, **B)** miRBase input vs miRNA true positive predictions for each tool (2 samples), **C)** Benchmarking metrics for all datasets tested, the error bar indicates the distance between the 2 replicates.

The hairpin structure of mature miRNAs is found in most of the BrumiR candidates.

In order to assess the performance of BrumiR on real data, we collected public datasets for the same plant and animal species evaluated in the synthetic benchmark (Figure 2A). On average, 15.4 and 18.2 raw million reads were used for the animal and plant datasets (Supplementary Table S4), respectively. The predictions of BrumiR were compared against those of the state-of-the-art tools encompassing reference and *de novo* based methods (Friedländer et al., 2012; Lei & Sun, 2014; Vitsios et al., 2017), after testing some of the most used

miRNA discovery tools, we selected the best performer (Supplementary Table S5, Supplementary Figure S9). In particular, we included mirnovo that similarly to BrumiR can discover mature miRNAs directly from the reads. Before running the tools, low-quality reads were removed using fastp (S. Chen et al., 2018) (~10%, see Methods section). All the predicted miRNAs for each tool were annotated using the miRBase database to identify known and novel predictions. On average, BrumiR predicted ~1,000 putative mature miRNAs for the animal species, which was ~2.8X higher than the miRDeep2 candidates and 1.7X lower than the candidates predicted by mirnovo (Figure 3A1). For plant species, BrumiR predicted on average ~1,800 putative mature miRNAs, which was lower than the candidates predicted by mirR-PREFeR (3,248 on average), and higher than the predictions of mirnovo (301 on average) (Figure 3A1). A comparison using the miRBase (Kozomara & Griffiths-Jones, 2014) annotated miRNAs revealed that BrumiR shared more candidates with miRDeep2 and miR-PREFeR than with mirnovo (Figure 3A2). However, an important fraction (on average more than 70%) of the miRBase-annotated candidates were exclusive to each tool (Figure 3A2), which summarizes the complexity of miRNA discovery.

Considering miRBase-annotated candidates as the ground-truth, we computed precision, recall and F-Score for all the evaluated tools (Figure 3B, Method section). BrumiR achieved an accuracy (F-Score) better for animals and plants to the one obtained by the other software (Figure 3B3). Moreover, BrumiR consistently reached the highest recall for most of the datasets evaluated (Figure 3B2). The precision values of BrumiR were slightly lower for

some datasets (Figure 3B1). However, methods based on the reference genome as miRDeep2 has better precision in some datasets because the predictions are more conservative than the *de novo* methods (Figure 3B3). In particular, miRdeep2 reached the highest precision (~0.7) on animal species and all methods struggled on the evaluated plant species (average precision <0.47). The lower precision with plant species may be the product of a low number of entries annotated in miRBase for plants (10.414 vs 38.471 animals) as well as of a higher complexity of plant miRNAs (Meyers et al., 2008).

We also compared BrumiR core to de Bruijn graph transcriptome de novo assemblers (Trinity and Velvet, see Methods) (Grabherr et al., 2011; Zerbino & Birney, 2008) in order to assess the performance of a pure de Bruijn graph approach for miRNA discovery. We can observe that the de novo transcriptome assemblers generated on average 40X and 4X more candidates than BrumiR, for Trinity and Velvet respectively (Supplementary Table S7). In general, the huge number of contigs generated by the transcriptome assemblers, even after filtering them by length, were poorly matched to the miRBase entries (1,2% and 36%,indeed). On the other hand, BrumiR matched the miRBase entries at a rate of 1 every 2 candidates (52% precision average). As expected, we can conclude that most of the contigs generated by a pure de Bruijn graph transcriptome assembler are poorly related to miRNA sequences. This was expected because they are developed for mRNAseq analysis and do not consider the complexities of the sRNA seq data like BrumiR.

In summary, this experiment showed that BrumiR and all the downstream steps it performs after the de Bruijn graph construction are essential for miRNA discovery.

The BrumiR toolkit also provides a tool to determine the hairpin loop of miRNA precursor sequences, which is the main structural feature of miRNAs (Roden et al., 2017). BrumiR2reference maps the BrumiR predicted mature miRNA to the reference genome using an exhaustive alignment (See Methods section), generates precursor sequences, computes its secondary structure, and checks the hairpin structure using a variety of criteria inferred from analyzing more than 30,000 miRBase precursor sequences from animal and plant species (see Methods section). We used BrumiR2reference as a double validation for all the predicted mature miRNAs generated by BrumiR for the animal and plant datasets (Figure 3C). On average, BrumiR2reference identified a valid precursor sequence having the characteristic hairpin structure for over 60% of the BrumiR candidates (Figure 3C).

In terms of speed, BrumiR core was the fastest tool. BrumiR was on average 19X and 38X times faster than miRDeep2 and miR-PREFeR, respectively (See Table S6).

Overall, we demonstrated that BrumiR is a competitive tool for discovering mature miRNAs without a reference genome. We showed that it was the most sensitive on most of the datasets tested. The performance of our method was not only faster, but also better or comparable to the state-of-the-art tools. Moreover, we also provide a new mapper approach to be used when a

reference genome is available, to further verify if a precursor sequence of the predicted mature miRNA is present in the genome. BrumiR therefore represents a reliable alternative for the discovery of mature miRNAs in model and non-model species with or without a reference genome.

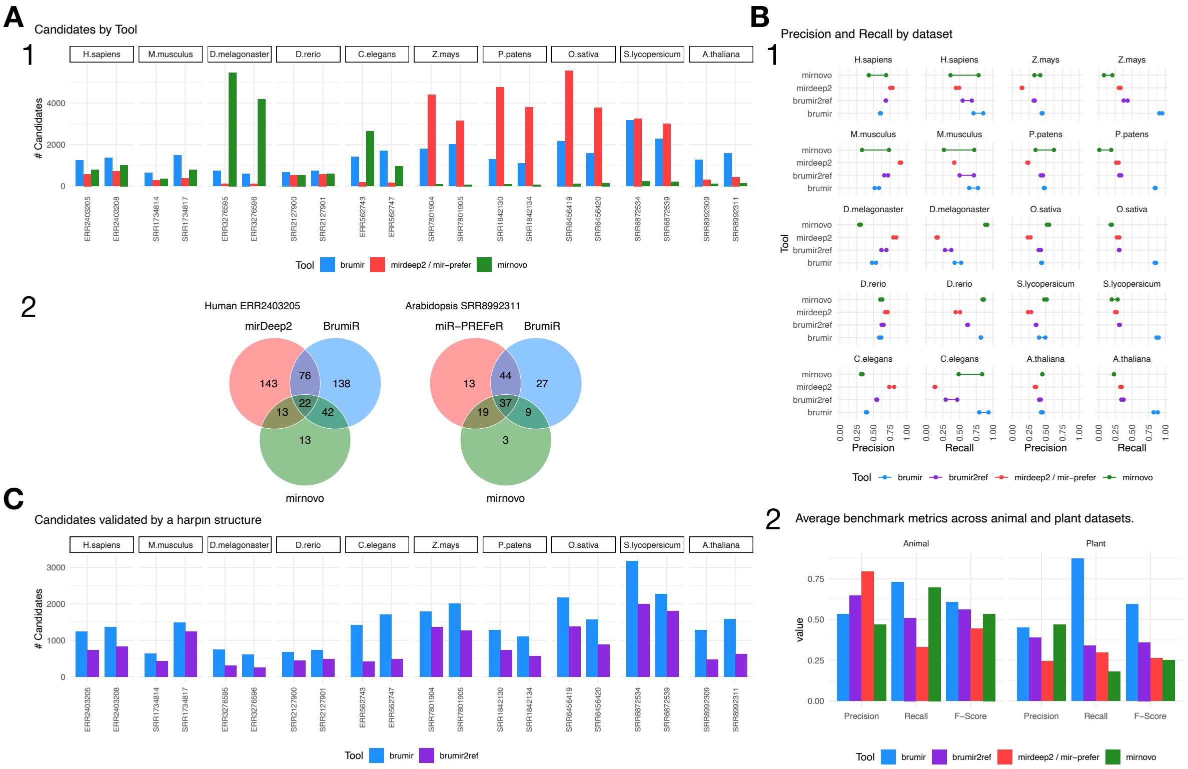

Figure 3. Real dataset benchmark of BrumiR and state-of-the-art tools. A) Number of predictions by tool for all the datasets and the overlap between them for 2 datasets (1 for animal and 1 for plant); **B)** Benchmarking metrics computed using miRBase annotated miRNAs, precision and recall for each dataset; and average metrics, including F-score. **C)** BrumiR candidates validated by Hairpin structure (BrumiR2Reference).

Discovering novel miRNAs from sRNA-seq data of *A. thaliana* roots using BrumiR.

A. thaliana is one of the best characterized model organisms, and the first plant species in which miRNAs were cloned and sequenced (Reinhart et al., 2002). To date, 436 mature miRNA sequences are included in the miRBase database. Most of these miRNAs have been identified by studies addressing the sRNAome of different plant organs (Fahlgren et al., 2007), cell types (Breakfield et al., 2011), or responses to biotic or abiotic stress using sRNA-seq (Hsieh et al., 2009; Moldovan et al., 2010) (Hsieh et al., 2009).

We sequenced sRNA-seq libraries from the roots of *A. thaliana* after different time points during vegetative development (see Methods section) (Figure S12) to demonstrate the potential of BrumiR to discover novel mature miRNAs in a known biological context. BrumiR was run independently for each condition and replicate. The day 5 samples were excluded because of the low number of reads when compared to the other samples (Supplementary Table S8). BrumiR predicted, on average, 1,160 mature miRNAs per sample, which were further refined to 719 using the BrumiR2ref tool. To take advantage of our experimental design, we considered as a putative miRNA the ones present in the three replicates (core predictions) (Axtell & Meyers, 2018) (Figure 4A). Novel miRNAs were identified using the following steps: First, predictions were classified as known miRNAs by comparing with miRBase (141 known miRNAs out of a total of 159 miRNAs already described for *A. thaliana* in miRBase). These known miRNAs were put aside to explore the sensitivity of BrumiR in detecting novel

putative miRNAs. We then clustered the remaining putative miRNAs into three stages: early, late, and constitutive (Figure 4B). The days 9, 13 and 17 represent an early stage of the plant development; days 17, 21 and 25 represent a late stage of the plant development (Satbhai et al., 2015), and the putative miRNAs expressed in all conditions represent the constitutive category (Supplementary Table S8). A total of 21 putative novel miRNAs were identified, and a manual curation was carried out revising all the criteria to validate and annotate miRNAs in plants (Axtell & Meyers, 2018). We discovered two novel miRNAs candidates that fulfill all the recommended criteria to annotate miRNAs in plants (Figure S10, Table S8). According to the revised criteria, confirmation by blot of the expression of the miRNA or miRNA* is disallowed, and it is suggested that validation of miRNA expression should be based on sRNA-seq reads only. In this way, these two curated novel miRNA candidates are supported directly from the sRNA-seq libraries and are expressed in all replicates in all conditions (Axtell & Meyers, 2018).

One of the curated novel miRNAs candidates (miR-8) is located in Chromosome 5 (Figure 4C), this miRNA locus has not been previously discovered because its mature sequence maps to multiple chromosomes, and is therefore discarded by genome-based tools (Ziemann et al., 2016).

In an exploratory analysis to shed light on the potential targets of these novel miRNAs, we conducted an *in silico* target transcript prediction using the 1 algorithm (Dai et al., 2018) (Supplementary Table S10). EXO84b (AT5G49830) was found to be one of the top genes regulated by this novel miRNA miR-8

(Supplementary Table S9). In A. thaliana, it has been demonstrated the importance of EXO84b in the development of trackeary elements or vassel xylem system which is essencial for water and nutrient transport of vascular plants (Vukašinović et al., 2017). EXO84b is expressed over all days but significantly abundantly expressed in the last days, and its differential accumulation between root zones is related to emerging patterns of lateral roots and hair formation from trichomes (Dvořák et al., 2020).

We have also explored the known miRNAs identified by BrumiR in where we have found in almost all the samples, with a higly expression, the plant miRNAs that would be playing a key role in root specification and development (Couzigou & Combier, 2016).

It is plausible to say that these novel and known miRNAs may be involved in the fine-tuning of lateral root growth in the early stages of development.

These results highlight the value of the BrumiR toolkit for discovering novel and known miRNA candidates with functional impact on the organisms studied, even in the case where high quality genomes are available.

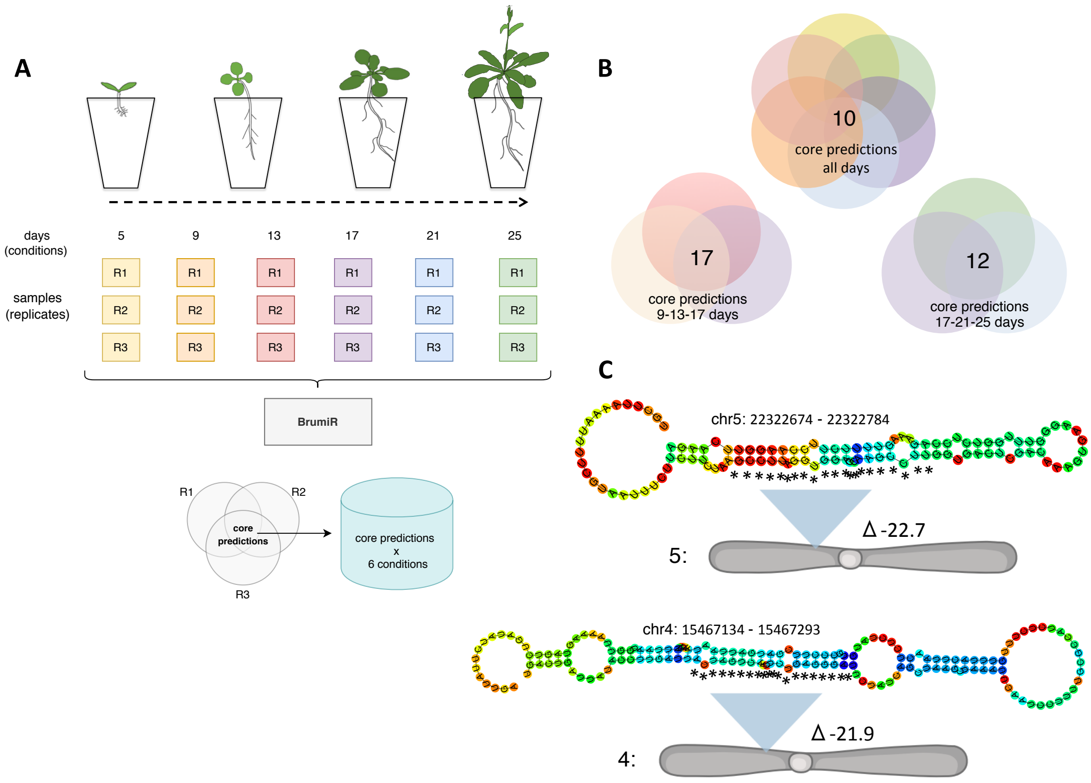

Figure 4. Applying BrumiR on sRNA-seq from *Arabidopsis* root libraries. A) Experimental design implemented; roots from *Arabidopsis* in a time-scale per day as conditions were sequenced in three technical replicates. BrumiR was used to analyze all sRNA-seq librairies, and conserved predictions by the three replicates was considered as a core by condition. **B)** Different combinations of root growth per day were analyzed together to identify novel putative miRNAs conserved in all conditions. **C)** We discovered 2 candidates as novel miRNAs that fulfill the current criteria to annotate miRNAs in plants. Moreover, they were supported directly from the sRNA-seq libraries and are conserved in all replicates in all conditions.

DISCUSSION

In this paper, we introduced and benchmarked the BrumiR toolkit, which was designed for enabling the identification of mature miRNAs in model and non-model species with or without a reference genome, encompassing the plant and animal kingdoms. The BrumiR toolkit implements the following algorithms:

1) a new discovery miRNA tool (BrumiR-core), 2) a specific genome mapper (BrumiR2ref), and 3) an sRNA-seq read simulator (miRsim). We demonstrated that BrumiR is capable of identifying mature miRNAs based only on the sequence information and generates results that are better or comparable to the state-of-the-art tools on simulated and real datasets. We further tested the usefulness of the BrumiR toolkit for discovering novel miRNAs potentially involved in the regulation of the root development of the extensively annotated *A. thaliana* genome.

Unlike the state-of-the-art tools, BrumiR starts by encoding the sRNA-seq reads using a de Bruijn graph. This avoids the read mapping stage and the dependency on previous miRNA annotations. It also enables the identification of sequencing artifacts. A critical step of genome-based miRNA discovery tools is to identify the precursor sequence when a reference genome is available. BrumiR introduces a new mapping approach, BrumiR2reference, which scans every possible hairpin precursor in the genome, when such is available, for all the BrumiR predictions. As the hairpin structure is determined using the predicted mature miRNA instead of the reads, this alignment can support mismatches and indels and handles the case of multi-mapped candidates (due to repetitive regions of the genome). Such features distinguish BrumiR from the current genome-based methods.

Discovering miRNAs in non-model species is one of the limitations of the current methods. One exception is mirnovo, which similarly to BrumiR can predict miRNAs using only the sRNA-seq data, and a specific training set for animal and plant species. We thus compared its performance to the one of

BrumiR. Our results show that mirnovo is very conservative, generating few predictions in comparison to BrumiR, this could be due to the low number of entries of plant miRNAs in miRBase because mirnovo approach is based on miRNAs families present in this database. However, miR-PREFeR generates a larger number of candidates in plant species. The higher number of predictions of miR-PREFeR results in lower precision in most of the evaluated datasets, in which BrumiR obtained the highest performance in plant species. In animal species, BrumiR has a lower precision compared to miRDeep2, and in some cases, it generates a large number of candidates, which represents a potential weakness of our method. We examined possible piRNA sequences present in some of the samples (*Mus musculus* SRR1734817) to see if this high number of candidates was due to wrong predictions, but no relationship was found (Supplementary Table S11). To address the number of candidates generated, we developed the BrumiR2reference tool to refine the BrumiR predictions, thus reducing the number of false-positive putative miRNAs. We plan to further reduce the false-positive rate of BrumiR by using a random-forest classifier trained on the high confidence mature sequences available in miRBase. The latter will improve the accuracy of BrumiR even in the case when a reference genome is not available. It is important to observe that the miRNA annotations remain incomplete and although miRBase is the main repository for miRNAs, it cannot be considered as the gold-standard for most species (many of the entries have not been correctly validated, for example) (Bortolomeazzi et al., 2019). For this reason, the predictions of BrumiR are not based on miRBase in any step of the algorithm. However, miRBase can be used with all due

precaution in a posterior analysis to verify the miRNAs inferred in case of not having any reference genome.

In terms of computational resources and usability, BrumiR is the fastest method and provides a stand-alone package for running locally all the analyses. It further generates an output that is compatible with the bandage software (Wick et al., 2015), which can be employed to visualize and explore the results of BrumiR in a user-friendly way.

Moreover, BrumiR reports other sequences expressed in the sRNA-seq data among which are putative isomiRs and longer non-coding RNAs, thereby providing additional biological insight.

Finally, we tested the effectiveness of BrumiR on sequenced sRNA-seq libraries from the roots of *A. thaliana*, and were able to discover 2 novel putative miRNAs based on the very conservative criteria proposed in (Axtell & Meyers, 2018), showing the potential of it being used alone or in combination with other methods.

In summary, we present a new and versatile method that implements novel algorithmic ideas for the study of miRNAs that complements and extends the currently existing approaches.

MATERIALS AND METHODS

Building a de Bruijn graph for sRNA-seq data.

BrumiR starts by building a compact de Bruijn graph from the sRNA-seq reads given as input. De Bruijn graphs are a widely used approach in the genome assembly problem (Compeau et al., 2011). BrumiR uses this graph to organize, detect, and exploit the sequence information of sRNA-seq experiments. BrumiR takes as input sequencing files in FASTA or FASTQ formats. The sequencing data can be cleaned, using a fastq pre-processor (S. Chen et al., 2018) (*i.e.* fastp), to remove adapter sequences and trim low quality bases. BrumiR employs the BCALM (Chikhi et al., 2016) tool to build a de Bruijn graph from the sRNA-seq reads. BCALM uses a node-centric bi-directed de Bruijn graph where the nodes are *k*-mers, that is words of length *k*, and an arc between two nodes if the *k-1* suffix of one node is equal to the *k-1* prefix of the subsequent node, representing an exact overlap of *k-1* bases (Chikhi et al., 2016). A critical parameter of any de Bruijn graph approach is the *k*-mer size (Durai & Schulz, 2016). We observed that the length of all mature miRNA sequences stored in the miRBase database (v21) (Kozomara & Griffiths-Jones, 2014) fluctuates between 18 to 24nt (Supplementary Figure S1). To determine the optimal *k*-mer size for BrumiR, we compared the performance of BrumiR using different *k*-mer sizes (14-16-18-20-22). The benchmark shows that the optimal *k*-mer size for BrumiR is 14 (Supplementary Table S1, Supplementary Figure S2), because it allows for a better handling of the sequencing errors and

enables a more sensitive clustering of identical miRNA candidates, even when comparing with 18-mers which is the *k*-mer size expected for a mature miRNA sequence (Supplementary Figure S3). We thus empirically set the *k*-mer size equal to 14. BCALM compacts the nodes of the de Bruijn graph into maximal unipaths by gluing all the nodes of the graph with an in-degree and an out-degree equal to one, thus generating the so-called *unipath graph* (Chikhi et al., 2016). The unipath graph is the starting point of BrumiR (Figure1A). Notice that the unipath graph generated by BCALM does not represent what is expected for a set of mature miRNAs (one connected component for each miRNA) and therefore further graph operations are needed. BrumiR uses a minimum *k*-mer frequency (KM value) of 5 and all *k*-mers with lower frequency are ignored, without losing most of the information contained in the sequencing reads (Supplementary Figure S4).

Removing sequencing errors from the unipath sRNA-seq graph.

BrumiR deletes from the unipath graph all the nodes that have only one connection (degree equal to 1), known as dead-end paths or tips (Chikhi & Rizk, 2013). Usually, these nodes have a low abundance value associated to them (KM less than or equal to 5, the default parameter). Moreover, BrumiR deletes isolated nodes (degree equal to 0) having a low abundance; isolated nodes highly expressed are however conserved for further analysis. All these nodes

are likely artifacts generated from sequencing errors because they are not deeply expressed in the sRNAs-seq reads (Deorowicz et al., 2013). BrumiR iterates this step 3 times in order to prune and clean the unipath graph (polishing). This operation, called ‘tip removal’, edits the original unipath graph, and therefore a new unipath graph with a new structure is generated (Figure 1B).

An expressed mature miRNA has uniform coverage.

The unipath graph of a set of miRNAs from an sRNA-seq experiment has non-uniform coverage as different miRNAs and other elements may be connected in a single big component (Figure 1.1). BrumiR evaluates each connection of the unipath graph to identify those that link two nodes with a large expression difference. According to the miRNA biogenesis, after a stable miRNA precursor is cleaved by Dicer, among its three products, the miRNA mature sequence is the most abundant and when it is sequenced, it has a uniform expression along its sequence (Friedländer et al., 2008). Thus due to miRNA biogenesis, it is possible to capture the complete miRNA mature sequence having a homogeneous expression (Friedländer et al., 2012) directly from the sRNA-seq experiments. BrumiR expects a similar KM value for *k*-mers originating from the same mature miRNA gene. Accordingly, if we observe two connected nodes that show a big difference in their abundance values, this connection is deleted and we keep the nodes unconnected. In particular, two unipaths $U=\{a,b\}$ connected in the graph have a KM value associated to them that represents

their coverage from the reads information. BrumiR scans all the neighbor connections and if the difference between their KMs is larger than three-fold, the connection is deleted ($Ui_{km}/Uj_{km} > 3$). In this way, BrumiR defines a relative threshold that will depend on each unipath neighborhood in the graph. Finally, BrumiR repeats the tips removal step to eliminate new low frequency isolated nodes (Figure 1C).

miRNAs and other sequences are captured in single connected components.

After the previous steps of BrumiR, a new unipath graph emerges, with a new structure. It is thus necessary to identify and classify the new connected elements within the graph (Figure 1). A connected component (CC) of a graph is a maximal strongly connected subgraph (Lewis & Papadimitriou, 1982). BrumiR computes the CCs of the unipath graph, and then each CC is processed independently to identify miRNA candidates as well as to discard other sequences present in the unipath graph.

BrumiR classifies low abundance non-linear topologies as sequencing artefacts.

BrumiR detects topologies that are potentially related to sequencing errors and thus unlikely to be miRNA candidates. The shapes of these topologies were

identified by visual inspection of several unipath graphs and are described in detail in Figure S5. Usually they have low KM and are composed of lowly expressed branching nodes with 3, 4 or 5 connections to the principal structures in the graph (Figure S5). Moreover, we observed that the sequences contained in these topologies were usually redundant and contained in other linear and more expressed CCs. In this way, we are not discarding relevant sequence information. BrumiR removes about 10% of the CCs in this step.

Re-assembling unipaths within each CC.

BrumiR re-assembles all unipaths present in the linear CCs by bundling the nodes with in and out degree equal to 1 into a new unipath. BrumiR classifies them into different types based on their length. The latter is the length of the sequence represented by the new unipath. All CCs having a length between 18 and 24 are stored as potential miRNA sequences. The CCs corresponding to an isolated node that have high KM (KM>50) are included in the latter group. CCs with lengths over 24 are classified as longer sequences or other types of genomic sequences captured along with the miRNAs. The longer sequences are put aside for later analysis. Moreover, BrumiR identifies circular CCs and branching CCs. The former are circular unipaths and the latter CCs with a high number of branching nodes. Branching CCs are not considered in the subsequent steps because they are likely sequencing errors (low abundance) or contamination present in the sRNA-seq data (Figure S6).

Re-clustering potential miRNAs.

After grouping unipaths by CCs, BrumiR builds an overlap graph to rescue the missing connections between potential miRNA candidates sharing an overlap with another candidate. First, BrumiR adds all the candidates as nodes of the overlap graph, then an all-vs-all *k*-mer comparison is performed using exact overlaps of length *k*=15. Candidates sharing an exact overlap are connected in the overlap graph. Then, the connected components are computed to identify clusters of miRNA candidates, and the most expressed candidate within each component is selected as the representative candidate of the cluster. The representative candidates are compared all-vs-all in a second overlap step that allows a maximum edit distance of 2, which is implemented using the edlib library (Šošić & Šikić, 2017). BrumiR then builds a second overlap graph, computes again the connected components, and selects the most expressed candidate as the representative of each cluster. The other members of each connected component are classified as putative isomiRs and saved in a file for later analysis.

Identifying other expressed RNA sequences.

In sRNA-seq experiments, different types of RNAs are expressed, some of which, such as small non-coding RNA elements, may have similar length with miRNAs (Lambert et al., 2019). The RFAM database (Kalvari, Nawrocki, et al., 2018) is a collection of curated RNA families including three functional classes

of RNAs (non-coding, cis-regulatory elements, and self-splicing RNAs), which are classified into families according to their secondary structure and sequence information (Covariance Models) (Kalvari, Argasinska, et al., 2018). We downloaded 3,017 RNA families present in RFAM (v14.1) and excluded 529 miRNA families. The sequences of 2,488 RFAM families were concatenated (a total of 2,736,549 sequences) and used to build a 16-mer database with the KMC3 *k*-mer counter tool (Kokot et al., 2017) ("-fm -n100 -k16 -ci5"). All distinct 16-mers with a frequency lower than 5 were excluded, leading to a total of 6,204,556 distinct 16-mers related to RNA elements. Additionally, we downloaded all the mature miRNA sequences from miRBase (v22.1) (Kozomara & Griffiths-Jones, 2014) and built a 16-mer database with KMC3 ("-fm -n100 -k16 -ci1 mature.fa.gz"). RFAM 16-mers matching 16-mers from the 16-mer mature miRBase database were excluded from RFAM, leading to a 16-mer RFAM database with a total of 6,204,487 distinct 16-mers. Finally, the BrumiR candidates (18-24 length) were matched to the 16-mer RFAM database, and matching candidates were excluded and reported as sequences potentially associated to other RNA elements. The BrumiR candidates passing the aforementioned filter are reported as the final list of miRNA candidates.

Identifying precursor sequences for BrumiR candidates (BrumiR2Reference).

Unlike current state-of-the-art tools that perform miRNA discovery by mapping the sRNA-seq reads to a reference genome, BrumiR generates candidates by

operating directly on the sRNA-seq reads. The reduced list of potential BrumiR miRNA candidates permits the computation of a more exhaustive alignment than when mapping directly the sRNA-seq reads to the reference genome. BrumiR aligns each candidate to the reference genome using an exact alignment method that computes the edit distance (Meyers et al., 2008) between two strings and thus support mismatches, insertions and deletions. The BrumiR2reference tool divides the reference genome in non-overlapping windows of 200bp (adjustable parameter), then the window is indexed using 12-mers and each miRNA candidate is matched in both strands (split at 12-mers). When a 12-mer match is found, an exhaustive alignment is computed between the window and the matching miRNA candidate. The alignment is performed using a fast implementation of Myers' bit-vector algorithm (Šošić & Šikić, 2017).

A miRNA candidate is stored as a hit if the alignment in the current genomic window has an edit distance less than or equal to 2. After scanning all the genomic windows, the vector of hits is sorted by miRNA-candidate; edit distance (0-2), and alignment sequence coverage. For a single miRNA-candidate, a maximum of 100 genomic locations (best hits) are selected. BrumiR2reference then builds a potential precursor sequence for each selected hit using a strategy similar to the ones employed by miRDeep2 (Friedländer et al., 2012) and Mirinho (Higashi et al., 2015). BrumiR excises the potential precursor hairpin sequence from the flanking genomic coordinates of the reported miRNA candidate hits (mature sequence) in both strands. Potential precursor hairpin sequences of length 110 bp are built for animal species from

both strands, while for plant species hairpin sequences of lengths 110, 150, 200, 250 and 300 bp are built from both strands (Meyers et al., 2008). Secondary structure prediction for all the potential precursor sequences is performed using RNAfold (v2.4.9) (Lorenz et al., 2011). Secondary structures with a minimum free energy in the range of 15-80 kcal/mol are checked for a hairpin loop characteristic of miRNAs (Roden et al., 2017) (Figure S7). Structures with a hairpin loop composed of a single segment without pseudo-knot, multi-loops, external loops and with less than 5 bulges, 3 dangling ends, and 10 internal loops are classified as characteristic secondary structures of miRNA precursor sequences. The aforementioned filters were derived from analyzing the secondary structure of 38,589 precursor sequences stored in miRBase (v22.1) (Kozomara & Griffiths-Jones, 2014) using a modified version of the bpRNA program (Danaee et al., 2018) (Figure S8).

Benchmarking BrumiR against transcriptome de Bruijn graph assemblers.

In order to determine the value of BrumiR for extracting miRNA candidates directly from a de Bruijn graph, we compared the BrumiR approach against two de Bruijn graph transcriptome de novo assemblers, namely Trinity (Grabherr et al., 2011) and Velvet (Zerbino & Birney, 2008). The benchmark was performed using 4 real datasets; including human and *Arabidopsis*. The seed length and minimum contig length for the transcriptome assemblers were fixed at 14-mer for all tools. Then, the contigs longer than 24 nt were filtered out for Trinity and

Velvet. For BrumiR, we eliminated the last step using the RFAM database (Kalvari, Argasinska, et al., 2018) information to filter out other kinds of sRNA sequences, and we used all the predictions to compare to the transcriptome de novo assemblers. Finally, the BrumiR candidates and contigs generated by Trinity and Velvet were mapped against the miRBase database (Blast search).

Benchmarking BrumiR using simulated sRNA-seq reads.

We simulated synthetic reads from animal and plant species, and compared the results of BrumiR to those obtained with the miRDeep2 (Friedländer et al., 2012) and miR-PREFeR (Lei & Sun, 2014) tools. The sRNA-seq reads were simulated using miRsim (https://github.com/camoragaq/miRsim), a tool that we developed specifically for simulating sRNA-seq reads from a list of known miRNA mature sequences. miRsim is based on *wgsim* (https://github.com/lh3/wgsim), which is a widely used tool for simulating short Illumina genomic reads. miRsim includes functionalities specific of sRNA-seq reads such as variable depth/coverage and shorter read lengths. miRNA mature sequences were obtained from miRBase (Kozomara & Griffiths-Jones, 2014) for animal (High Confidence) and plant species. Additionally, to simulate the typical fragments contained in real sRNA-seq data, we included sequences from the RFAM database (v14.1) (Kalvari, Argasinska, et al., 2018) and random genomic sequences from the genomes for each of the species included in the

benchmark (10% of the sequences for RFAM and genomic sequences, respectively). The animal species that we considered were: *Homo sapiens, Mus musculus*, *Drosophila melanogaster, Danio rerio*, and *Caenorhabditis elegans*, while the following plant species were included: *A. thaliana*, *Oryza sativa, Physcomitrella patens, Zea mays,* and *Solanum lycopersicum*. Supplementary Table S2 provides further details (*i.e.* number of reads, number of mature miRNAs *etc*.) for each simulated dataset. MiRDeep2 was run on the animal datasets with the default parameters and using the score suggested by the developers, providing the respective reference genome. Similarly, miR-PREFeR was run with the default parameters on the plant datasets. BrumiR was run with the default parameters on both the animal and plant datasets. The miRNA annotations were not included for the genome-based tools in order to make a fairer comparison with BrumiR which does not use this information. The list of simulated miRNAs was considered as the ground truth, and precision, recall and F-Score quality metrics were computed to assess the performance of each discovery tool. The benchmark metrics were defined as follows:

$$Recall = \frac{TP}{TP + FN}$$

$$Precision = \frac{TP}{TP + FP}$$

$$F - score = 2 * \frac{(Recall * Precision)}{(Recall + Precision)}$$

where:

TP= true positive elements predicted as miRNAs present in the miRBase input list.

FP= false positive elements predicted as miRNAs but not present in the miRBase input list.

FN= false negative elements not predicted as miRNAs, but that were present in the miRBase input list.

Benchmarking BrumiR using real sRNA-seq reads.

We downloaded publicly available sRNA-seq data for the plant and animal species listed in the synthetic benchmark, and two datasets for each species were included (Supplementary Table S4). We wanted to benchmark BrumiR in a simple but exhaustive way by selecting the top performer for genome-based and genome-free methods. We benchmarked some of the most used prediction tools in a reduced version of the real dataset, and the results were conclusive to select the best methods (Supplementary Table S5, Supplementary Figure S9). We included mirnovo (Vitsios et al., 2017), a tool that can discover miRNAs without a reference genome. The predictions of BrumiR were benchmarked along with MiRDeep2 (v2.0.1.2) (Friedländer et al., 2012) and mirnovo for the animal datasets. Similarly, miR-PREFeR (Lei & Sun, 2014) replaced MiRDeep2 for the plant datasets (Supplementary Table S4).

The stand-alone packages of BrumiR, miRDeep2 and miR-PREFeR were used to discover miRNAs in all datasets. The software mirnovo was run using its web version because the stand-alone package was not available and the developer

recommends the use of the web version instead. The miRNA discovery was performed for each sample independently using default parameters for MiRDeep2, miR-PREFeR and mirnovo. In particular, we used the scripts provided by miRDeep2 and miR-PREFeR to map the reads to the reference genome, and the predictions for these tools were performed on the resulting alignment files. The mirnovo predictions were done using the animal and plant universal panel respectively, as recommended when the reference genome is not available. BrumiR was run using the command line and parameters provided in the Supplementary Section 1. Moreover, the predictions of BrumiR were refined using the BrumiR2reference tool on the available reference genome of the selected species (Supplementary Table S4). Benchmark metrics (precision, recall, and F-Score) were computed as before but considering all the annotated mature sequences present in miRBase (v22.1) as the ground-truth.

miRNA discovery from *Arabidopsis* root samples.

A. thaliana Col-0 seedlings were grown hydroponically on Phytatrays on 0.5X Murashige and Skoog medium (Phytotechnology Laboratories, cat. M519) under long-day conditions (16h light and 8h dark) at 22°C. Total RNA was isolated from plant roots after 5, 9, 13, 17, 21, and 25 days post-germination using the mirVana miRNA Isolation Kit (Thermo Fisher Scientific, cat. AM1560). RNA concentration was determined using the Qubit RNA BR Assay Kit (Thermo Fisher Scientific, cat. Q10210), and integrity was verified by capillary

electrophoresis on a Fragment Analyzer™ (Advanced Analytical Technologies, Inc.). The indexed sRNA libraries were built employing the TruSeq small RNA Sample Preparation Kit (Illumina, Inc.) following the manufacturer's instructions. Briefly, 3’ and 5’ adaptors were sequentially ligated to 1 μg of total RNA prior to reverse transcription and library amplification by PCR. Size selection of the sRNA libraries was performed on 6% Novex TBE PAGE Gels (Thermo Fisher Scientific, cat. EC6265BOX) and purified by ethanol precipitation. Both the library size assessment and library quantification were carried out in a Fragment Analyzer™. Finally, the libraries were pooled and sequenced on an Illumina NextSeq 500 platform (Supplemetary FigureS12).

All samples were analyzed with BrumiR separately with default parameters to identify the candidate miRNAs. We further validated the candidates having a putative precursor with a hairpin structure analysis using the BrumiR2ref tool with the reference genome for *A. thaliana* (GCF_000001735.4_TAIR10.1_genomic.fna). All validated candidate miRNAs were compared to known miRNAs described for *A. thaliana* (437) present in miRBase (v21) (Supplemetary TableS8). We used the current criteria to validate and annotate miRNAs in plants which are based on experimental evidence coming directly from the sequencing libraries, as shown in Axtell 2018 (Axtell & Meyers, 2018); we conserved the candidates predicted in all the replicates (as is described in Figure 4); and the putative novel miRNAs were manually curated, specifically (Supplemetary TableS9), we checked the criteria related to precursor length, hairpin structure and miRNA length in at least two sRNA-seq libraries (biological replicates) (Figure S10) (Bortolomeazzi et al., 2019). Then a

target analysis was performed using the Araport 11 cDNA library with the plant-specific psRNATarget algorithm (based on a best expectation score) (Supplemetary TableS10) (Dai et al., 2018).

CODE AVAILABILITY

The BrumiR code (v1.0) used in this manuscript is freely available at https://github.com/camoragaq/BrumiR, and is open software under the MIT license. Also, a docker image is available on dockerHub at (https://hub.docker.com/repository/docker/camoragaq/BrumiR), and a demo dataset hosted at https://github.com/camoragaq/BrumiR_demo.

ACKNOWLEDGEMENTS

This work was supported by CONICYT BECAS CHILE DOCTORADO 2016/FOLIO 72170320 granted to CM, by a post-doctorate fellowship from the Agence National de Recherche (ANR-GREEN 17_CE20_0031_01) granted to MGF, as well as by Fondo Nacional de Desarrollo Científico y Tecnológico (FONDECYT)-ANID grant 1170926, ANID PCI-Redes Internacionales entre Centros de Investigación grant REDES180097 and Instituto Milenio iBio - Iniciativa Científica Milenio MINECON granted to E.A.V. This research was performed using the computing facilities of the LBBE/PRABI and the France Génomique e-infrastructure (ANR-10-INBS-09-08).

AUTHOR CONTRIBUTIONS

CM designed, developed, implemented and benchmarked BrumiR. MFS guided the development of BrumiR. ES conducted the *A. thaliana* experiments. EAV designed and supervised the *A. thaliana* experiments. CM wrote the initial version of the manuscript with inputs from all other authors. MFS and MGF

helped to improve the manuscript. EAV, MFS, MGF and RAG provided crucial biological feedback. All authors provided helpful discussions for the work and reviewed the manuscript.

9.0.1 Conflict of interest statement.

None declared.

References

A reference standard for genome biology. (2018). *Nature Biotechnology*, *36*(12), 1121. https://doi.org/10.1038/nbt.4318

Axtell, M. J., & Meyers, B. C. (2018). Revisiting Criteria for Plant MicroRNA Annotation in the Era of Big Data. *The Plant Cell*, *30*(2), 272–284. https://doi.org/10.1105/tpc.17.00851

Bartel, D. P. (2004). MicroRNAs: Genomics, Biogenesis, Mechanism, and Function. *Cell*, *116*(2), 281–297. https://doi.org/10.1016/S0092-8674(04)00045-5

Bartel, D. P. (2009). MicroRNAs: Target recognition and regulatory functions. *Cell*, *136*(2), 215–233. https://doi.org/10.1016/j.cell.2009.01.002

Bartel, D. P. (2018). Metazoan MicroRNAs. *Cell*, *173*(1), 20–51. https://doi.org/10.1016/j.cell.2018.03.006

Borchert, G. M., Lanier, W., & Davidson, B. L. (2006). RNA polymerase III transcribes human microRNAs. *Nature Structural & Molecular Biology*, *13*(12), 1097–1101. https://doi.org/10.1038/nsmb1167

Bortolomeazzi, M., Gaffo, E., & Bortoluzzi, S. (2019). A survey of software tools for microRNA discovery and characterization using RNA-seq. *Briefings in Bioinformatics*, *20*(3), 918–930. https://doi.org/10.1093/bib/bbx148

Breakfield, N. W., Corcoran, D. L., Petricka, J. J., Shen, J., Sae-Seaw, J., Rubio-Somoza, I., Weigel, D., Ohler, U., & Benfey, P. N. (2011). High-resolution experimental and computational profiling of tissue-specific known and novel miRNAs in Arabidopsis. *Genome Research*. https://doi.org/10.1101/gr.123547.111

Cai, X., Hagedorn, C. H., & Cullen, B. R. (2004). Human microRNAs are processed from capped, polyadenylated transcripts that can also function as mRNAs. *RNA (New York, N.Y.)*, *10*(12), 1957–1966. https://doi.org/10.1261/rna.7135204

Chen, L., Heikkinen, L., Wang, C., Yang, Y., Sun, H., & Wong, G. (2019). Trends in the development of miRNA bioinformatics tools. *Briefings in Bioinformatics*, *20*(5), 1836–1852. https://doi.org/10.1093/bib/bby054

Chen, S., Zhou, Y., Chen, Y., & Gu, J. (2018). fastp: An ultra-fast all-in-one FASTQ preprocessor. *Bioinformatics*, *34*(17), i884–i890. https://doi.org/10.1093/bioinformatics/bty560

Chikhi, R., Limasset, A., & Medvedev, P. (2016). Compacting de Bruijn graphs from sequencing data quickly and in low memory. *Bioinformatics*, *32*(12), i201–i208. https://doi.org/10.1093/bioinformatics/btw279

Chikhi, R., & Rizk, G. (2013). Space-efficient and exact de Bruijn graph representation based on a Bloom filter. *Algorithms for Molecular Biology*, *8*(1), 22. https://doi.org/10.1186/1748-7188-8-22

Compeau, P. E. C., Pevzner, P. A., & Tesler, G. (2011). Why are de Bruijn graphs useful for genome assembly? *Nature Biotechnology*, *29*(11), 987–991. https://doi.org/10.1038/nbt.2023

Couzigou, J.-M., & Combier, J.-P. (2016). Plant microRNAs: Key regulators of root architecture and biotic interactions. *New Phytologist*, *212*(1), 22–35. https://doi.org/10.1111/nph.14058

Dai, X., Zhuang, Z., & Zhao, P. X. (2018). psRNATarget: A plant small RNA target analysis server (2017 release). *Nucleic Acids Research*, *46*(W1), W49–W54. https://doi.org/10.1093/nar/gky316

Danaee, P., Rouches, M., Wiley, M., Deng, D., Huang, L., & Hendrix, D. (2018). bpRNA: Large-scale automated annotation and analysis of RNA secondary structure. *Nucleic Acids Research*, *46*(11), 5381–5394. https://doi.org/10.1093/nar/gky285

Deorowicz, S., Debudaj-Grabysz, A., & Grabowski, S. (2013). Disk-based k-mer counting on a PC. *BMC Bioinformatics*, *14*(1), 160. https://doi.org/10.1186/1471-2105-14-160

Durai, D. A., & Schulz, M. H. (2016). Informed kmer selection for de novo transcriptome assembly. *Bioinformatics*, *32*(11), 1670–1677. https://doi.org/10.1093/bioinformatics/btw217

Dvořák, P., Krasylenko, Y., Ovečka, M., Basheer, J., Zapletalová, V., Šamaj, J., & Takáč, T. (2020). FSD1: Developmentally-regulated plastidial, nuclear and cytoplasmic enzyme with anti-oxidative and osmoprotective role. *Plant, Cell & Environment*. https://doi.org/10.1111/pce.13773

Fahlgren, N., Howell, M. D., Kasschau, K. D., Chapman, E. J., Sullivan, C. M., Cumbie, J. S., Givan, S. A., Law, T. F., Grant, S. R., Dangl, J. L., & Carrington, J. C. (2007). High-Throughput Sequencing of Arabidopsis microRNAs: Evidence for Frequent Birth and Death of MIRNA Genes. *PLOS ONE*, *2*(2), e219. https://doi.org/10.1371/journal.pone.0000219

Friedländer, M. R., Chen, W., Adamidi, C., Maaskola, J., Einspanier, R., Knespel, S., & Rajewsky, N. (2008). Discovering microRNAs from deep sequencing data using miRDeep. *Nature Biotechnology*, *26*(4), 407–415. https://doi.org/10.1038/nbt1394

Friedländer, M. R., Mackowiak, S. D., Li, N., Chen, W., & Rajewsky, N. (2012). MiRDeep2 accurately identifies known and hundreds of novel microRNA genes in seven animal clades. *Nucleic Acids Research*, *40*(1), 37–52. https://doi.org/10.1093/nar/gkr688

Grabherr, M. G., Haas, B. J., Yassour, M., Levin, J. Z., Thompson, D. A., Amit, I., Adiconis, X., Fan, L., Raychowdhury, R., Zeng, Q., Chen, Z., Mauceli, E., Hacohen, N., Gnirke, A., Rhind, N., di Palma, F., Birren, B. W., Nusbaum, C., Lindblad-Toh, K., … Regev, A. (2011). Trinity: Reconstructing a full-length transcriptome without a genome from RNA-Seq data. *Nature Biotechnology*, *29*(7), 644–652. https://doi.org/10.1038/nbt.1883

Greene, J., Baird, A.-M., Brady, L., Lim, M., Gray, S. G., McDermott, R., & Finn, S. P. (2017). Circular RNAs: Biogenesis, Function and Role in Human Diseases. *Frontiers in Molecular Biosciences*, *4*, 38. https://doi.org/10.3389/fmolb.2017.00038

Higashi, S., Fournier, C., Gautier, C., Gaspin, C., & Sagot, M.-F. (2015). Mirinho: An efficient and general plant and animal pre-miRNA predictor for genomic and deep sequencing data. *BMC Bioinformatics*, *16*(1), 179. https://doi.org/10.1186/s12859-015-0594-0

Hsieh, L.-C., Lin, S.-I., Shih, A. C.-C., Chen, J.-W., Lin, W.-Y., Tseng, C.-Y., Li, W.-H., & Chiou, T.-J. (2009). Uncovering small RNA-mediated responses to phosphate deficiency in Arabidopsis by deep sequencing. *Plant Physiology*, *151*(4), 2120–2132. https://doi.org/10.1104/pp.109.147280

Jha, A., & Shankar, R. (2013). miReader: Discovering Novel miRNAs in Species without Sequenced Genome. *PLOS ONE*, *8*(6), e66857. https://doi.org/10.1371/journal.pone.0066857

Kalvari, I., Argasinska, J., Quinones-Olvera, N., Nawrocki, E. P., Rivas, E., Eddy, S. R., Bateman, A., Finn, R. D., & Petrov, A. I. (2018). Rfam 13.0: Shifting to a genome-centric resource for non-coding RNA families. *Nucleic Acids Research*, *46*(D1), D335–D342. https://doi.org/10.1093/nar/gkx1038

Kalvari, I., Nawrocki, E. P., Argasinska, J., Quinones-Olvera, N., Finn, R. D., Bateman, A., & Petrov, A. I. (2018). Non-Coding RNA Analysis Using the Rfam Database. *Current Protocols in Bioinformatics*, *62*(1), e51. https://doi.org/10.1002/cpbi.51

Khvorova, A., Reynolds, A., & Jayasena, S. D. (2003). Functional siRNAs and miRNAs exhibit strand bias. *Cell*, *115*(2), 209–216. https://doi.org/10.1016/s0092-8674(03)00801-8

Kokot, M., Dlugosz, M., & Deorowicz, S. (2017). KMC 3: Counting and manipulating k-mer statistics. *Bioinformatics (Oxford, England)*, *33*(17), 2759–2761. https://doi.org/10.1093/bioinformatics/btx304

Kozomara, A., & Griffiths-Jones, S. (2014). miRBase: Annotating high confidence microRNAs using deep sequencing data. *Nucleic Acids Research*, *42*(Database issue), D68–D73. https://doi.org/10.1093/nar/gkt1181

Lagos-Quintana, M., Rauhut, R., Lendeckel, W., & Tuschl, T. (2001). Identification of novel genes coding for small expressed RNAs. *Science (New York, N.Y.)*, *294*(5543), 853–858. https://doi.org/10.1126/science.1064921

Lambert, M., Benmoussa, A., & Provost, P. (2019). Small Non-Coding RNAs Derived from Eukaryotic Ribosomal RNA. *Non-Coding RNA*, *5*(1), 16. https://doi.org/10.3390/ncrna5010016

Langmead, B., Trapnell, C., Pop, M., & Salzberg, S. L. (2009). Ultrafast and memory-efficient alignment of short DNA sequences to the human genome. *Genome Biology*, *10*(3), R25. https://doi.org/10.1186/gb-2009-10-3-r25

Lau, N. C., Lim, L. P., Weinstein, E. G., & Bartel, D. P. (2001). An Abundant Class of Tiny RNAs with Probable Regulatory Roles in Caenorhabditis elegans. *Science*, *294*(5543), 858–862. https://doi.org/10.1126/science.1065062

Lee, Y., Ahn, C., Han, J., Choi, H., Kim, J., Yim, J., Lee, J., Provost, P., Rådmark, O., Kim, S., & Kim, V. N. (2003). The nuclear RNase III Drosha initiates microRNA processing. *Nature*, *425*(6956), 415–419. https://doi.org/10.1038/nature01957

Lee, Y., Jeon, K., Lee, J.-T., Kim, S., & Kim, V. N. (2002). MicroRNA maturation: Stepwise processing and subcellular localization. *The EMBO Journal*, *21*(17), 4663–4670. https://doi.org/10.1093/emboj/cdf476

Lee, Y., Kim, M., Han, J., Yeom, K.-H., Lee, S., Baek, S. H., & Kim, V. N. (2004). MicroRNA genes are transcribed by RNA polymerase II. *The EMBO Journal*, *23*(20), 4051–4060. https://doi.org/10.1038/sj.emboj.7600385

Lei, J., & Sun, Y. (2014). miR-PREFeR: An accurate, fast and easy-to-use plant miRNA prediction tool using small RNA-Seq data. *Bioinformatics (Oxford, England)*, *30*(19), 2837–2839. https://doi.org/10.1093/bioinformatics/btu380

Lewis, H. R., & Papadimitriou, C. H. (1982). Symmetric space-bounded computation. *Theoretical Computer Science*, *19*(2), 161–187. https://doi.org/10.1016/0304-3975(82)90058-5

Li, H., & Durbin, R. (2009). Fast and accurate short read alignment with Burrows–Wheeler transform. *Bioinformatics*, *25*(14), 1754–1760. https://doi.org/10.1093/bioinformatics/btp324

Li, Y., Zhang, Z., Liu, F., Vongsangnak, W., Jing, Q., & Shen, B. (2012). Performance comparison and evaluation of software tools for microRNA deep-sequencing data analysis. *Nucleic Acids Research*, *40*(10), 4298–4305. https://doi.org/10.1093/nar/gks043

Lin, R., He, L., He, J., Qin, P., Wang, Y., Deng, Q., Yang, X., Li, S., Wang, S., Wang, W., Liu, H., Li, P., & Zheng, A. (2016). Comprehensive analysis of microRNA-Seq and target mRNAs of rice sheath blight pathogen provides new insights into pathogenic regulatory mechanisms. *DNA Research*, *23*(5), 415–425. https://doi.org/10.1093/dnares/dsw024

Lorenz, R., Bernhart, S. H., Höner zu Siederdissen, C., Tafer, H., Flamm, C., Stadler, P. F., & Hofacker, I. L. (2011). ViennaRNA Package 2.0. *Algorithms for Molecular Biology*, *6*(1), 26. https://doi.org/10.1186/1748-7188-6-26

Mapleson, D., Moxon, S., Dalmay, T., & Moulton, V. (2013). MirPlex: A tool for identifying miRNAs in high-throughput sRNA datasets without a genome. *Journal of Experimental Zoology. Part B, Molecular and Developmental Evolution*, *320*(1), 47–56. https://doi.org/10.1002/jez.b.22483

Meyers, B. C., Axtell, M. J., Bartel, B., Bartel, D. P., Baulcombe, D., Bowman, J. L., Cao, X., Carrington, J. C., Chen, X., Green, P. J., Griffiths-Jones, S., Jacobsen, S. E., Mallory, A. C., Martienssen, R. A., Poethig, R. S., Qi, Y., Vaucheret, H., Voinnet, O., Watanabe, Y., … Zhu, J.-K. (2008). Criteria for Annotation of Plant

MicroRNAs. *The Plant Cell*, *20*(12), 3186–3190. https://doi.org/10.1105/tpc.108.064311

Moldovan, D., Spriggs, A., Yang, J., Pogson, B. J., Dennis, E. S., & Wilson, I. W. (2010). Hypoxia-responsive microRNAs and trans-acting small interfering RNAs in Arabidopsis. *Journal of Experimental Botany*, *61*(1), 165–177. https://doi.org/10.1093/jxb/erp296

Morin, R. D., O'Connor, M. D., Griffith, M., Kuchenbauer, F., Delaney, A., Prabhu, A.-L., Zhao, Y., McDonald, H., Zeng, T., Hirst, M., Eaves, C. J., & Marra, M. A. (2008). Application of massively parallel sequencing to microRNA profiling and discovery in human embryonic stem cells. *Genome Research*, *18*(4), 610–621. https://doi.org/10.1101/gr.7179508

Peng, Y., & Croce, C. M. (2016). The role of MicroRNAs in human cancer. *Signal Transduction and Targeted Therapy*, *1*, 15004. https://doi.org/10.1038/sigtrans.2015.4

Pinzón, N., Li, B., Martinez, L., Sergeeva, A., Presumey, J., Apparailly, F., & Seitz, H. (2017). MicroRNA target prediction programs predict many false positives. *Genome Research*, *27*(2), 234–245. https://doi.org/10.1101/gr.205146.116

Reinhart, B. J., Weinstein, E. G., Rhoades, M. W., Bartel, B., & Bartel, D. P. (2002). MicroRNAs in plants. *Genes & Development*, *16*(13), 1616–1626. https://doi.org/10.1101/gad.1004402

Roden, C., Gaillard, J., Kanoria, S., Rennie, W., Barish, S., Cheng, J., Pan, W., Liu, J., Cotsapas, C., Ding, Y., & Lu, J. (2017). Novel determinants of mammalian primary microRNA processing revealed by systematic evaluation of hairpin-

containing transcripts and human genetic variation. *Genome Research*, *27*(3), 374–384. https://doi.org/10.1101/gr.208900.116

Satbhai, S. B., Ristova, D., & Busch, W. (2015). Underground tuning: Quantitative regulation of root growth. *Journal of Experimental Botany*, *66*(4), 1099–1112. https://doi.org/10.1093/jxb/eru529

Schwarz, D. S., Hutvágner, G., Du, T., Xu, Z., Aronin, N., & Zamore, P. D. (2003). Asymmetry in the assembly of the RNAi enzyme complex. *Cell*, *115*(2), 199–208. https://doi.org/10.1016/s0092-8674(03)00759-1

Šošić, M., & Šikić, M. (2017). Edlib: A C/C ++ library for fast, exact sequence alignment using edit distance. *Bioinformatics*, *33*(9), 1394–1395. https://doi.org/10.1093/bioinformatics/btw753

Vitsios, D. M., Kentepozidou, E., Quintais, L., Benito-Gutiérrez, E., van Dongen, S., Davis, M. P., & Enright, A. J. (2017). Mirnovo: Genome-free prediction of microRNAs from small RNA sequencing data and single-cells using decision forests. *Nucleic Acids Research*, *45*(21), e177–e177. https://doi.org/10.1093/nar/gkx836

Vukašinović, N., Oda, Y., Pejchar, P., Synek, L., Pečenková, T., Rawat, A., Sekereš, J., Potocký, M., & Žárský, V. (2017). Microtubule-dependent targeting of the exocyst complex is necessary for xylem development in Arabidopsis. *The New Phytologist*, *213*(3), 1052–1067. https://doi.org/10.1111/nph.14267

Wang, J., Chen, J., & Sen, S. (2016). MicroRNA as Biomarkers and Diagnostics. *Journal of Cellular Physiology*, *231*(1), 25–30. https://doi.org/10.1002/jcp.25056

Wick, R. R., Schultz, M. B., Zobel, J., & Holt, K. E. (2015). Bandage: Interactive visualization of de novo genome assemblies. *Bioinformatics*, *31*(20), 3350–3352. https://doi.org/10.1093/bioinformatics/btv383

Zerbino, D. R., & Birney, E. (2008). Velvet: Algorithms for de novo short read assembly using de Bruijn graphs. *Genome Research*, *18*(5), 821–829. https://doi.org/10.1101/gr.074492.107

Ziemann, M., Kaspi, A., & El-Osta, A. (2016). Evaluation of microRNA alignment techniques. *RNA*, *22*(8), 1120–1138. https://doi.org/10.1261/rna.055509.115

Supplementary material revised version

Click here to access/download
Supplementary Material
BrumiR-rev-Supplementary.pdf

Click here to access/download
Supplementary Material
BrumiR-supp.pdf

Click here to access/download;Personal Cover;BrumiR-rev-letter.pdf

5th November 2021

Dear Dr Nicole Nogoy,

Thank you very much for considering our manuscript for publication in GigaScience. We would like to thank the referees and you for the careful assessment of our manuscript. We have attempted to address all points raised by the referees and hope that the responses are satisfactory. With these revisions, we believe that our manuscript has been substantially improved and hope that it is now suitable for publication in GigaScience.

Please find below our point-by-point replies to the reviewers' comments. All changes in the main manuscript and the supplement have been marked in blue font, including all the Supplementary material. We refer to the changes we have made in the manuscript using consecutive line numbering.

Yours sincerely,

Carol Moraga

Reviewer #1: Summary:
The authors developed a de novo assembly method, BrumiR, for small RNA sequencing data based on de Bruijin graph algorithm. This tool displayed a relatively high sensitivity in finding miRNAs and helped the authors discover a novel miRNA in A. thaliana roots.
Major comments:
1. Have the authors compared the performance with different seed lengths? Even if the minimal miR length is 18nt in MiRBase 21, seed=18 might not necessarily lead to the best AUC or F score (This might also be related to Comment 4).

We followed the reviewer recommendations and we now compare the performance of BrumiR using different seed lengths of 14, 16, 18, 20, and 22; in both animal and plant datasets. The benchmark results are reported in Table S1. The benchmark shows that the optimal seed size for BrumiR is indeed 14 and not 18 as was previously set by default. The main reason for this improved performance is that shorter seed lengths permit a better handling of sequencing errors (shorter *k*-mers are less likely to be sequencing errors), and enable a more sensitive clustering of identical miRNAs. Overall, we observe that the benchmark metrics (Supplementary Table S1, Figure S2) improves in comparison to the observed for the previous seed length (k=18) by 1.67X , 0.97X, 1.46X for precision, recall, and F-score in animal species, and 4.09X, 1.13X, 3X for precision, recall, and F-score in plant species, respectively. In light of the results, we have now set the default seed length to *k*=14, and we thank the reviewer again for this key suggestion that allows us to further improve the performance of BrumiR. Finally, the code has been updated accordingly and a new release has been published in the Github repository.

The updated text is in page 8, lines 13; page 25, lines 18-22.

2. The authors need to benchmark BrumiR with more existing tools (e.g. those ML-based methods), and to include more genome-free methods (e.g. MiRNAgFree).

In our current benchmark, we included miRDeep2, miR-PREFeR and mirnovo, and a total of 10 animal and plant datasets. miRDeep2 is the most used tool in the field with more than 1530 citations and is considered the reference miRNA prediction tool for animal species. In addition, we included miR-PREFeR that, like miRDeep2, is one of the few tools designed specifically for plant species when a reference genome is available. Complementing these popular reference-based methods,

we included mirnovo, which is a genome-free discovery tool based on machine learning. The mirnovo manuscript presents a benchmark comparison with miReader and miRplex which are de novo tools and mirnovo showed better performance than the aforementioned tools. This is one of our main reasons for considering mirnovo as the best tool for performing miRNA discovery without a reference genome. Therefore, we selected the tools that perform best for miRNA discovery, including reference and de novo based.

Nevertheless, we tried to follow the reviewer's suggestion and attempted to add more tools to our benchmark. However, we had difficulties in running the existing tools because most of them are not under active development, do not provide update binaries (standalone versions), or their web-server does not work. For instance, miRplex does not provide recent binaries (the current one, v.01, is from 2012), leading to problems with some old libraries and we could not run it. In another example, the miReader software mirPlex is not in active development (updated for the last time in 2014). We were able to run the standalone version, but after letting it run for more than one month on a single sample, we decided to stop it.

Besides these issues, we succeeded in running the miRanalyzer (genome-reference based), and miRNAgFree (de novo based) tools. The benchmark results are provided in Supplementary Table S5 using a reduced version of the real dataset. The benchmark results show that BrumiR obtained the highest F-score rates, as do miRDeep2 and mirnovo (Supplementary FigureS13). We also observed the poor performance on plant species of most of the tools, which shows the potential of BrumiR, as well as being a tool that can be used for both animal and plant species.

Currently, there is no standard for benchmarking miRNA prediction methods, which means that each new method tries to make the comparison in its own way. For this reason, we tried to make an effort to create a benchmark standard and we developed miRsim to generate benchmarks based on a ground truth known a priori and using the same conditions for all the tools.

Overall, we wanted to benchmark BrumiR in a simple but exhaustive way selecting the top performers for genome-based and genome-free methods. Finally, several miRNA tools do not provide stand-alone versions and therefore prevent extensive benchmarking when only the server is available. Being able to generate a good benchmark remains a challenge in the field of miRNAs where there are no standards.

The updated text is in page 13, lines 17; page 14, lines 1-2; page 36, lines 11-16.

3. It is also interesting to know whether de novo method for mRNA assembly would be useful on the miRNA side. It would be great if the authors were able to compare the performance of BrumiR2reference (without filtering for RFAM) with Trinity in genome-guided mode, by tweaking its seed length to be the same as BrumiR.

We followed the reviewer's recommendation and included two de Bruijn graph de novo transcriptome assemblers, namely Trinity (inchworm), and Velvet (Supplementary Table S7). The genome guide mode of Trinity first aligns the reads to the genome and then a de novo assembly is performed using the inchworm tool for each partition. The genome guide pipeline does not provide access to the seed length of inchworm (default k=25) whose default value exceeds the length of the miRNA sequences. Therefore, we ran it independently and without mapping the reads to the genome, because we thought that in this way the comparison between BrumiR and de novo transcriptome assemblers is more appropriate and allows us to focus on how the candidates are extracted from the de Bruijn graph. To perform the comparison, we used 4 real datasets; including human and *Arabidopsis*. The seed length and minimum contig length for the transcriptome assemblers were set to k=15 (edge-centric de Bruijn graph, overlap equals 14 nt), and l=18, for both inchworm and Velvet. Then, the contigs longer than 24 nt were filtered out for BrumiR (without filtering for RFAM) and the transcriptome de novo assemblers. Finally, the candidates were matched against miRBase (blast search) and the results of the comparison are presented in

Supplementary Table S7. We can observe that the de novo transcriptome assemblers generated on average 40X and 4X more candidates than BrumiR, for Trinity and Velvet, respectively.

In general, the huge number of contigs generated by the transcriptome assemblers, even after filtering them by length, were poorly matched to miRBase entries (1,2% and 36%, indeed). On the other hand, BrumiR matched miRBase entries at a rate of 1 every 2 candidates (52% precision average). As expected, we can conclude that most of the contigs generated by a pure de Bruijn graph transcriptome assembler are poorly related to miRNA sequences. This was expected because they are developed for mRNAseq analysis and do not consider the complexities of the sRNA seq data like BrumiR.
In summary, this experiment showed that BrumiR and all the downstream steps it performs after the De Bruijn graph construction are essential for miRNA discovery.

The updated text is in page 15, lines 9-22; page 16, lines 1-3; page 33, lines 14-22; page 34, lines 1-5.

4. The tool's sensitivity is promising across animal and plant datasets. However, the average precision is quite low, an average precision of 0.3 means a false discovery rate of 0.7. This is not an accepted value for a tool designed to discover novel miRNA. Is there any parameter the author could tweak towards a better performance? For example, is the seed length of 18nt too short to start with? Are there any other sequence features the authors should take into account to boost the performance? Or maybe some post-assembly filtering approaches might be sufficient and helpful.

We followed the reviewer’s recommendations and we were able to improve the precision of BrumiR while maintaining a similar sensitivity. The major gain in the precision of BrumiR comes from a reduced seed length, which allows it to better deal with sequencing errors, and from an improved clustering of the reads coming from the same miRNA candidates, plus some code improvements. Overall, our precision for animals and plants increased by 1.67X and 4.09X on average respectively, which led to a combined F-score increased by 1.5X and 3X for animals and plants respectively, significantly reducing the rate of false-positives of our previous version. This can be further reduced when a reference genome is available and BrumiR2Reference is used. Additionally, we also provided a new script to annotate the candidates using the miRBase entries, providing a subset of high confidence BrumiR predictions. The novel candidates can be further refined using additional criteria (such as coverage, presence on several miRNA-seq experiments, etc.) that we describe in Figure 4 and Supplementary FigureS10, in the discovery of novel miRNAs in the *Arabidopsis* genome using BrumiR.
Moreover, we intend to improve our method by implementing a random forest classifier that will enable a better characterization of the novel candidates not present in miRbase. The random forest model learns the sequence features of the miRNA annotated in miRbase and can be used to assign a probability of being a miRNA or not for novel BrumiR candidates for species with or without a reference genome. This feature should be included in a future version of our method.
However, at this point we believe that in the absence of a reference genome, BrumiR is able to provide a list of potential miRNA candidates which is not usually possible with the other genome-based prediction tools that have a similar prediction power. We compared BrumiR against the best genome based miRNA discovery tool and we had comparable or better results, even without using a reference genome.

The updated text is in page 8, lines 13; page 12, lines 3-15 ; page 13, lines 1-3; page 14, lines 21-22 ; page 17, lines 5-6; page 23, lines 1-13; page 25, lines 18-22; page 26, lines 1-4.

5. Wet-lab validation (e.g. Luciferase assay) for the identified novel miRs will leverage the real-life usefulness of BrumiR. This is extremely important, as the tool showed a high false discovery rate.

We agree that functional validation of miRNAs predicted by BrumiR is an important point in order to highlight the usefulness of our tool; and we intend to continue our studies with Arabidopsis' novel miRNA candidates in the near future to provide additional evidence towards the in vivo functions of such candidates in a more specialized manuscript. Unfortunately, this is not within the scope of our study and more specifically the current global pandemic has not helped us finish such functional studies, because this part of the work has been done in Chile where our partner wet-lab was closed most of the part of the pandemic and currently is resuming is normal operation.

Even so, we would like to point out that current criteria to validate and annotate miRNAs in plants are based on experimental evidence coming directly from the sequencing libraries and that confirmation by blot of the expression of the miRNA or miRNA* is disallowed, as proposed by Axtell 2018*. What we think could be done in order to improve the sRNA-seq dataset instead would be to enrich the sequencing libraries with functional sRNAs coupled to proteins (as proposed in Grentzinger et al., 2014**). At this point, we believe that we currently fulfill several state-of-the-art criteria for miRNA annotation in plants. We have revised all the criteria exposed in Axtell, 2018* for novel miRNA candidates predicted by BrumiR on the *Arabidopsis* miRNA-seq data. We concentrated our effort on 22 novel miRNA candidates that were expressed in at least 3 replicates, and we found that 4 of them fulfilled at least 7 of 8 criteria, with 2 of them fulfilling all the criteria exposed in Axtell 2018. We have updated this part of the manuscript (Figure 4, Figure S10, and Table S8, S9, S10) accordingly to reflect these changes and we thank the reviewer for pointing out the need for more comprehensive criteria to reflect the accuracy of BrumiR when predicting new candidates.

(*) Axtell, M. J., & Meyers, B. C. (2018). Revisiting criteria for plant microRNA annotation in the era of big data. *The Plant Cell*, *30*(2), 272-284.
(**) Grentzinger T, Armenise C, Pelisson A, Brun C, Mugat B, Chambeyron S. A user-friendly chromatographic method to purify small regulatory RNAs. Methods. 2014 May 1;67(1):91-101. doi: 10.1016/j.ymeth.2013.05.011. Epub 2013 May 28. PMID: 23727218.

The updated text is in page 19, lines 6-14; page 19, lines 21-23; page 20, lines 1-11; page 21, lines 1-10; page 38, lines 17-24.

Minor comments:

1. MiRNA maturation involves RNA editing. Can the authors comment on how this would be handled and captured by BrumiR. It seems that the authors allow mismatches when clustering the potential miRNAs via edlib library. It is interesting to know whether or not, or to what extent, edlib would help in including RNA edited candidates in the final result.

We use the edit distance library to allow a maximum of 2 nt differences in the cluster step of BrumiR. Then, for each cluster, we select the most expressed candidates but a reference is kept for all the cluster members. It is possible to use these clusters to identify miRNA candidates that may be RNA edited but the final output of BrumiR contains only one representative member of each cluster. We have included a flag in the output of BrumiR that reports the size of the cluster for each miRNA candidate. This allows us to explore if a candidate might be RNA edited or not. This is provided in a list as one of the BrumiR output files (*.edit_clusters.ED2.txt). Overall, BrumiR permits a mismatch maximum of two nucleotides when clustering candidates and therefore allows for the identification of candidates with potential RNA edition.

Reviewer #2:
The authors here present BrumiR, a de Bruijn-based method to discover miRNAs independently of a reference genome. Today most miRNA discovery and annotation is done by mapping sequenced RNAs to readily available reference genomes and analyzing the mapping profiles. However, there are some uses cases where the genome-free approach is needed (particularly for species that have no reference genome or where the genomes have missing parts); therefore BrumiR could potentially be useful for the community. However, the comparison to existing tools needs to be done in a more careful way.

Major comments:
1- RFAM filtering is not really part of the prediction step, this is rather a filtering step. Therefore, to make a fair comparison with mirnovo (the other genome-free tool), BrumiR should additionally be run without RFAM filtering, and mirnovo should additionally be run using the exact same RFAM filtering.

We used the RFAM database to filter out other kinds of RNAs present in the miRNA-seq experiment. Mirnovo calculates a consensus sequence for each cluster (candidate) and then an alignment is performed against the RFAM database to identify candidates matching rRNAs and tRNAs, and these matching candidates are filtered out by mirnovo. The latter step is performed by mirnovo by default and cannot be turned off (there is not a parameter allowing this). In BrumiR, we used the RFAM database to filter RNAs not associated with miRNAs. We built a k-mer database excluding all the RFAM families annotated as miRNAs. All the BrumiR candidates matching this k-mer database are excluded. This step is similar for both tools with the same goal (excluding other RNAs) but using different algorithms (alignment vs *k*-mer matches). We therefore considered that the comparison that we did is appropriate and fair for both tools. On average, 1% of the BrumiR candidates are filtered-out on this step.

The updated text is in page 9, lines 19-21.

2- it appears that 16-mers from miRBase miRNAs were specifically excluded from the RFAM catalog used for the filtering, which is reasonable. However, the miRNAs from the exact benchmarked species should not be included in the used miRBase 16-mer catalog, to avoid circular reasoning.

Yes, we agree with the reviewer that including miRNAs in this step may generate circular reasoning. This represents a final step of BrumiR where the aim is to remove sequences related to rRNAs and tRNAs, but we do not annotate or guide BrumiR towards previously annotated miRNA sequences present in the RFAM database. To achieve this, the 16-mer database was built from the RFAM database excluding all the families in all the taxa related to miRNA genes (529 families excluded), conserving the most abundant *k*-mers (more abundant than 5). Moreover, all 16-mers present in the miRBase database were also excluded. In summary, we excluded any reference to known miRNA sequences present in RFAM from the 16-mer database.

The updated text is in page 9, lines 19-21.

3- miRDeep2 software should ideally not be run with default options - this is particularly important since the miRDeep2 performance in this manuscript appears lower than what is reported in other studies (e.g. Friedlander et al. 2012). First, reference mature miRNAs from a related and well-annotated species should be included to support the prediction. Second, a score cut-off should be used that gives a decent signal-to-noise ratio according to the miRDeep2 output overview table (for instance 5:1). Third, all read pre-processing and genome mapping should be performed with the mapper.pl script which is part of the miRDeep2 package.

We agree with the reviewer that the ideal case is to use miRDeep2 according to the recommendations suggested by the developers (Friedländer et al, 2012). For this reason, we first used the script proposed by the developers to map the reads to the reference genome (mapper.pl). Then, we used the miRDeep2 pipeline in a “de novo” mode because we wanted to make a prediction without using previous knowledge, as we did with mirnovo and BrumiR. Finally, we selected all the predictions with a minimum miRDeep2 score of 5.1 (signal-to-noise ratio).
Also, the benchmark presented in the miRDeep2 manuscript was performed in a different way, because they used a pool of several samples, and the metrics were computed using all predictions, and for this reason, they are not comparable to our benchmark. As we mentioned above, there is no standard for benchmarking the miRNA prediction methods, which means that each new method tries to make the comparison in its own way and the metrics used could be quite different depending on the design of the benchmark.
miRDeep2 presented a good performance in most of the datasets, having the highest precision rate. However, we observed that it is a very conservative method because it does not predict many new candidates, even using the "de novo" mode.

The updated text is in page 35, lines 8-9, lines 11-13; page 13, lines 17; page 14, lines 1-2; page 15, lines 1-3;.

4- it appears that only miRNA-derived sequences were included in the simulated data. In fact, real small RNA-seq data typically contains fragments from other known types of RNA and also sequences from unannotated parts from the genome. Therefore, the authors should use simulated data that also includes samples from RFAM and randomly sampled sequences from the reference genome (for instance 10% of each). Overall, the use of simulated sequence data could be put a bit in the background in this study, since real small RNA-seq data is in fact readily available these days and typically has a structure that is not easy to simulate. Further, there is little reason not to use real data, since the miRNAs in miRBase tend to be reasonably well curated for most species and therefore can function well as a gold standard for benchmarking.

We followed the reviewer’s suggestion and we added to our simulated data sequences from the RFAM and the genomes for each of the species included in the benchmark. Now the simulated data include 10% of sequences from RFAM and 10% of random genomic sequences. We have repeated the synthetic benchmark and the new results are reported in the corresponding section. We agree that it is hard to mimic the structure of real miRNA-seq experiments, but the simulated data provide a good overview of the performance of the tools as well as a more controlled way to compare such performance since all the expressed miRNA sequences are known, which may not be the case for real data. Finally, we also included an extensive benchmark for the same species using real miRNA-seq data (Supplementary TableS4) and used the miRbase entries as the gold standard for computing the benchmark metrics as proposed by the reviewer.

The updated text is in page 11, lines 11-17; page 34, lines 19-22; page 35, lines 1-2.

5- precision of BrumiR is in some cases lower than 0.2, for instance for one mouse dataset. From this dataset ~3000 mouse miRNAs are reported - the majority of which are not in miRBase and can reasonably be presumed to be false positives. The authors should comment on why this particular dataset appears to produce so many false positives for BrumiR - could this have to do with the prevalence of piRNAs that the software cannot easily discern from miRNAs? Also, the authors should reflect on what kind of use cases could tolerate these thousands of false positives. Would this be for generating candidates for downstream high-throughput validation?

We took into account the recommendations of Reviewer #1 which led to a considerable improvement in our predictions and improved the benchmarking metrics in general across all the datasets. After we changed the *k*-mer size to 14, we improved over all the datasets the precision rates of BrumiR (Supplementary TableS1, TableS4). Moreover, to address the question of

Reviewer #2 related to the possibility of having piRNAs predicted as putative miRNAs in this dataset, we compared the predictions of BrumiR against the repository of piRNAs annotated for *Mus musculus* (https://www.pirnadb.org/). We observed that less than 4% of BrumiR candidates have a match with a piRNAs annotated in this database (Supplementary TableS9), a 3% and 10% of predictions of miRDeep2 and mirnovo, respectively, have a match with piRNAs. We can observe that none of the miRNA discovery tools would be capturing piRNAs instead of miRNAs, according to the data presented here.

The updated text is in page 12, lines 3-15; page 14, lines 20-21; page 15, line 1-3; page 23, lines 10-13.

6- the authors should either benchmark BrumiR against the genome-free methods miReader and MirPlex, or explain why this comparison is not relevant.

We used mirnovo as a de novo predicted tool. As in the mirnovo paper, the authors compare mirnovo against these tools, miReader and MirPlex, and mirnovo has the best performance, we chose to compare BrumiR only against the best performance tools only in a genome-based and de novo approach, meaning against respectively miRDeep2 and mirnovo, as we explained above we compared BrumiR with 4 different miRNA discovery tools using a reduced version of the real dataset. The results provided in Supplementary TableS5 showed that the highest F-score rates are for BrumiR, miRDeep2, miR-PREFeR and mirnovo (Supplementary FigureS9). We decided to benchmark BrumiR in a simple but exhaustive way selecting the top performers for genome--based and genome-free methods.

The updated text is in page 13, lines 17; page 14, lines 1-2; page 36, lines 11-16.

Minor comments:
- the brief introduction to miRNA biology should be carefully edited by an expert in the field. Currently, very old reviews are being cited (e.g. Bartel 2004), and some of the other references appear to be a bit spurious (e.g. why focus on plant host-pathogen interactions out of the hundreds of established functions of miRNAs?). The excellent review of Dave Bartel from 2018 contains references to numerous milestone studies that the introduction could build on.
We included miRNA biology background and we verified our references. Thanks to the reviewers for having pointed this out.

The updated text is in page 3, lines 6-20.

- the authors write on page 2 that genome-based methods struggle with a high rate of false positive prediction, citing [9]. However, this is a mis-reference, since the reference [9] states that methods that rely on _only_ the genome and do not leverage on small RNA-seq data have high false positive rates.
We changed this sentence.

The updated text is in page 4, lines 20-21.

Reviewer #3:

The manuscript by Moraga et al. describes BrumiR, a software devoted to the de novo identification of miRNAs from deep sequencing experiments of the RNA fraction at low molecular weight.

In contrast with existing tools, BrumiR is based on de Bruijn graphs, generated directly from raw fastq reads. The performances on simulated and real sequencing data, in terms of precision, recall and F-Score, are very good. In addition, the tool is ultra-fast, enabling the analysis of huge amount of data.

I have tried to use BrumiR but I always got a GLIB error. I have tested the script on different Linux and Mac computers but I was not able to fix the GLIB error. It seems that a very recent version of the GLIB library is required. So, unfortunately, I didn't have the possibility to test the program and look at the outputs.

Major concerns:

1- I was not able to run the program and, thus, provide a correct revision. In my opinion, the github page should take into account this by providing the minimal software and hardware architecture to run BrumiR. Authors could also include a copy of the output files (by the way, there is a typo in the description of the second output file).

We do thank the reviewer for raising this point and we created a docker container that provides all the needed software to facilitate the execution of BrumiR. The docker image is available on dockerHub at (https://hub.docker.com/repository/docker/camoragaq/BrumiR) and instructions for downloading the image are provided on the Github repository of BrumiR. Additionally, we have created a demo dataset hosted at https://github.com/camoragaq/BrumiR_demo where we have included all the expected output files for the demo dataset.

The updated text is in page 40, lines 4-6.

2- Since the tool is able to identify novel miRNAs and look also at known ones, they could provide an output file including the read count per miRNA. In addition, since the tool is expected to be ultra-fast (not checked … see above), the differential gene expression analysis could also be implemented.

BrumiR outputs an abundance estimation based on the *k*-mer counts for each predicted miRNA candidate. Currently, BrumiR quantify each candidate using the total number of *k*-mers (KC), and the average number of *k*-mers per base (KM). Both values can be used as proxy to access the expression of each miRNA candidate. However, how has been previously shown for transcriptome mRNAseq data the accurate estimation of gene expression based on *k*-mers require the development of specific algorithms (Kallisto, Salmon) (Bray et al., 2016*) (Patro et al., 2017**). The adaptation of these algorithmics ideas to miRNAseq data is one of the main ideas that we are going to develop in a subsequent manuscript of the BrumiR toolkit.

(*) Bray, N., Pimentel, H., Melsted, P. *et al.* Near-optimal probabilistic RNA-seq quantification. *Nat Biotechnol* **34,** 525–527 (2016). https://doi.org/10.1038/nbt.3519

(**) Patro, R., Duggal, G., Love, M. I., Irizarry, R. A., & Kingsford, C. (2017). Salmon provides fast and bias-aware quantification of transcript expression. *Nature methods*, *14*(4), 417–419. https://doi.org/10.1038/nmeth.4197

3- I suggest also implementing a graphical output. A sort of summary in a decorated html page.

We followed the reviewer's suggestion and we now provide an R notebook to create an interactive and graphical summary of the output of BrumiR. We do plan to extend these capabilities in the future to further enhance the graphical report (i.e. by providing functions to quickly determine differentially expressed miRNAs). A full example of the R Notebook is provided with the demo dataset.

The updated text is in page 40, lines 5-6.

4- By using BrumiR, authors analyze miRNAs in Arabidopsis during the development, discovering three novel miRNAs. Although bioinformatics evidences indicate that they could be real miRNAs, an experimental validation is required. Indeed, these miRNAs have been detected by BrumiR only. I think that this validation could be easily done because authors directly performed sRNAseq data. In my opinion, this experiment could really improve the manuscript and assess the high performance of BrumiR.

We agree with the reviewer that experimental validation of the candidate miRNAs is an important step and a valuable suggestion for our work. Following the reviewer's suggestion, we have revised all the updated criteria on plant miRNA annotation based on Axtell, 2018* for each novel candidate miRNA predicted by BrumiR using the *Arabidopsis thaliana* datasets. These criteria are based directly on precursor and miRNA features that can be determined directly from the sRNA-Seq reads. Considering these criteria, we found that 2 of our miRNA candidates fulfill all of them (Figure 4, Figure S8, S9, S10). As such, these results highlight the usefulness of BrumiR for predicting new candidate miRNAs even in model species where the miRNA catalogs are highly complete. Although, this last results section of the BrumiR manuscript highlight the power of BrumiR to discover new miRNAs even in highly curated genomes, we believe that the biological insight and subsequent validations may be the focus of a more specialized manuscript rather than the current one where the focus is the new algorithmic ideas that we are presenting.

(*) Axtell, M. J., & Meyers, B. C. (2018). Revisiting criteria for plant microRNA annotation in the era of big data. *The Plant Cell*, *30*(2), 272-284.

The updated text is in page 19, lines 6-14; page 19, lines 21-23; page 20, lines 1-11; page 21, lines 1-10; page 38, lines 17-24.

Again, we would like to thank all referees for their crucial suggestions that inspired us to further improve BrumiR, led to remarkable new benchmark results, and made the flow of the Results part more natural.

Sincerely,

Carol Moraga on behalf of all authors,

Personal Cover
Click here to access/download;Personal Cover;BrumiR-cover-letter.pdf

Marie-France SAGOT, Director of Research Inria
Inria & Laboratoire de Biométrie et Biologie Évolutive (LBBE) – UMR5558
University of Lyon
43 bd. du 11 Novembre 1918
69622 Villeurbanne cedex
France

Giga Science
Oxford Academic
Oxford, England, OX2 6DP, United Kingdom

Lyon, August 27th, 2020

Dear Editor,

Please find enclosed our manuscript entitled "BrumiR: A toolkit for *de novo* discovery of microRNAs from sRNA-seq data" which we would like to submit for publication as an article in *GigaScience*.

Since the first classification and annotation of miRNAs, accurately identifying them has proven difficult. Accurate prediction of known and novel miRNAs is however essential for increasing our understanding of the miRNA biology. In the last decade, with the increasing accessibility of high-throughput sequencing technologies, different methods have been developed to identify miRNAs, but most of them rely exclusively on pre-existing reference genomes. However, despite all the advancements in the sequencing technologies and *de novo* assembly algorithms, few complete genomes are available today, which is a recurrent problem that researchers working on non-model species face. Therefore, the lack of a high-quality reference genome reduces the possibilities for discovering novel miRNAs.

In this article, we introduce the BrumiR toolkit, which is a package composed of three tools; 1) a new discovery miRNA tool (BrumiR-core), 2) a specific genome mapper (Brumir2Reference), and 3) a sRNA-seq read simulator (miRsim). In particular, BrumiR-core is a *de novo* algorithm based on a de Bruijn graph approach that is able to identify miRNAs directly and exclusively from sRNA-seq data. Unlike other state-of-the-art tools, BrumiR does not rely on a reference genome, on the availability of close phylogenetic species, or on conserved sequence information. Instead, BrumiR starts from a de Bruijn graph encoding all the reads and is able to directly identify putative mature miRNAs on the generated graph. Along with miRNA discovery, BrumiR assembles and identifies other types of small and long non-coding RNAs expressed within the sequencing data. Additionally, when a reference genome is available, BrumiR provides a new mapping tool (BrumiR2reference) that performs an exhaustive search to identify and validate the precursor sequences.

We extensively benchmarked the BrumiR toolkit on animal and plant species using simulated and real datasets. The benchmark results show that BrumiR is very sensitive, is the fastest tool, and its predictions were supported by the characteristic hairpin structure of miRNAs. Finally, we demonstrate the power of BrumiR for discovering novel miRNAs in the model plant *Arabidopsis thaliana*. We sequenced a total of 18 sRNA-seq libraries from different stages of root development and used the BrumiR toolkit to analyze our data. We annotated three novel miRNAs involved in root development, showing on a real biological situation how BrumiR catches novel information even in the case of highly annotated genomes.

The preprint of BrumiR is in BioRxiv [https://doi.org/10.1101/2020.08.07.240689], and the BrumiR code is freely available in GitHub [https://github.com/camoragaq/BrumiR]. The BrumiR toolkit is a versatile method that represents an important contribution to the discovery of miRNAs in species with or without a reference genome. We therefore believe that it is appropriate to the wide audience of *GigaScience*.

We confirm that this manuscript has not been published elsewhere and is not under consideration by another journal. All authors have approved the manuscript and agree with the submission to *GigaScience*. The authors have no conflict of interest to declare.

Please address all correspondence to: Carol Moraga, camoragaq@gmail.com, INRIA, France; and Marie-France Sagot, marie-france.sagot@inria.fr, INRIA, France.

We look forward to hearing from you at your earliest convenience.

Sincerely,

Marie-France Sagot, Director of Research Inria
