# Supplementary material for: BrumiR: A toolkit for de novo discovery of microRNAs from sRNA-seq data
Source: Gigascience. 2022 Oct 25;11:giac093. doi: 10.1093/gigascience/giac093 (PMC9596168; doi:10.1093/gigascience/giac093)
Supplement: giac093_GIGA-D-20-00262_Revision_3 [file giac093_giga-d-20-00262_revision_3.pdf]

GigaScience

BrumiR: A toolkit for de novo discovery of microRNAs from sRNA-seq data.

--Manuscript Draft--

**Manuscript Number:**	GIGA-D-20-00262R3
**Full Title:**	BrumiR: A toolkit for de novo discovery of microRNAs from sRNA-seq data.
**Article Type:**	Technical Note
**Funding Information:**	becas chile DOCTORADO - ANID (72170320) — Dr. Carol Moraga<br>Agence Nationale de la Recherche (ANRGREEN 17_CE20_0031_01) — Dr Mariana Galvao Ferrarini<br>fondecyt Chile (1170926) — Dr Elena A Vidal<br>ANID redes internacionales (REDES180097) — Dr Elena A Vidal<br>anid fondecyt (1211130) — Dr Elena A Vidal
**Abstract:**	MicroRNAs (miRNAs) are small non-coding RNAs that are key players in the regulation of gene expression. In the last decade, with the increasing accessibility of high-throughput sequencing technologies, different methods have been developed to identify miRNAs, most of which rely on pre-existing reference genomes. However, when a reference genome is absent or is not of high quality, such identification becomes more difficult. In this context, we developed BrumiR, an algorithm that is able to discover miRNAs directly and exclusively from sRNA-seq data. We benchmarked BrumiR with datasets encompassing animal and plant species using real and simulated sRNA-seq experiments. The results demonstrate that BrumiR reaches the highest recall for miRNA discovery, while at the same time being much faster and more efficient than the state-of-the-art tools evaluated. The latter allows BrumiR to analyze a large number of sRNA-seq experiments, from plants or animal species. Moreover, BrumiR detects additional information regarding other expressed sequences (sRNAs, isomiRs, etc.), thus maximizing the biological insight gained from sRNA-seq experiments. Additionally, when a reference genome is available, BrumiR provides a new mapping tool (BrumiR2ref) that performs an a posteriori exhaustive search to identify the precursor sequences. Finally, we also provide a machine learning classifier based on a Random Forest model that evaluates the sequence-derived features to further refine the prediction obtained from BrumiR-core. The code of BrumiR and all the algorithms that compose the BrumiR-toolkit are freely available at https://github.com/camoragaq/BrumiR.
**Corresponding Author:**	Carol Moraga<br>Université Claude Bernard Lyon 1: Universite Claude Bernard Lyon 1<br>Villeurbanne, FRANCE
**Corresponding Author Secondary Information:**	
**Corresponding Author's Institution:**	Université Claude Bernard Lyon 1: Universite Claude Bernard Lyon 1
**Corresponding Author's Secondary Institution:**	
**First Author:**	Carol Moraga
**First Author Secondary Information:**	
**Order of Authors:**	Carol Moraga<br>Evelyn Sanchez<br>Mariana Galvao Ferrarini<br>Rodrigo A Gutierrez<br>Elena A Vidal

Powered by Editorial Manager® and ProduXion Manager® from Aries Systems Corporation

	Marie-France Sagot
**Order of Authors Secondary Information:**	
**Response to Reviewers:**	Dear Nicole,<br><br>Following the last revisions, we have completed all the editorial requirements regarding the format and availability of datasets and code.<br>We provide the manuscript in the format required by GigaScience, and in an editable format for production.<br><br>Thanks and best regards,<br><br>Carol
**Additional Information:**	
**Question**	**Response**
Are you submitting this manuscript to a special series or article collection?	No
**Experimental design and statistics**<br><br>Full details of the experimental design and statistical methods used should be given in the Methods section, as detailed in our Minimum Standards Reporting Checklist. Information essential to interpreting the data presented should be made available in the figure legends.<br><br>Have you included all the information requested in your manuscript?	Yes
**Resources**<br><br>A description of all resources used, including antibodies, cell lines, animals and software tools, with enough information to allow them to be uniquely identified, should be included in the Methods section. Authors are strongly encouraged to cite Research Resource Identifiers (RRIDs) for antibodies, model organisms and tools, where possible.<br><br>Have you included the information requested as detailed in our Minimum Standards Reporting Checklist?	Yes
**Availability of data and materials**	Yes

Powered by Editorial Manager® and ProduXion Manager® from Aries Systems Corporation

All datasets and code on which the conclusions of the paper rely must be either included in your submission or deposited in publicly available repositories (where available and ethically appropriate), referencing such data using a unique identifier in the references and in the "Availability of Data and Materials" section of your manuscript.<br><br>Have you have met the above requirement as detailed in our Minimum Standards Reporting Checklist?	

Manuscript

Click here to access/download;Manuscript;BrumiR-revised-version-update.docx

BrumiR: A toolkit for *de novo* discovery of microRNAs from sRNA-seq data.

Carol Moraga[1,2,*], Evelyn Sanchez[3,4], Mariana Galvão Ferrarini[1,5], Rodrigo A. Gutierrez[4,6,7], Elena A. Vidal[3,4,8], Marie-France Sagot[1,2,*]

[1]Université de Lyon, Université Lyon 1, CNRS, Laboratoire de Biométrie et Biologie Evolutive UMR 5558, F-69622 Villeurbanne, France. Erable Team, Inria Grenoble Rhône-Alpes, 38334 Montbonnot, France. [2]Universidad de O'Higgins, Instituto de Ciencias de la Ingeniería, Rancagua, Chile. [3]Centro de Genómica y Bioinformática, Facultad de Ciencias, Universidad Mayor, Chile. [4]Millennium Institute for Integrative Biology iBio, Chile. [5]University of Lyon, INSA-Lyon, INRA, BF2i, UMR0203, F-69621 Villeurbanne, France. [6]Departamento de Genética Molecular y Microbiología, Facultad de Ciencias Biológicas, Pontificia Universidad Católica de Chile. [7]FONDAP Center for Genome Regulation, Instituto de Ecología y Biodiversidad. [8]Escuela de Biotecnología, Facultad de Ciencias, Universidad Mayor.

To whom correspondence should be addressed: Carol Moraga - camoragaq@gmail.com, Marie-France Sagot – marie-france.sagot@inria.fr

Abstract

MicroRNAs (miRNAs) are small non-coding RNAs that are key players in the regulation of gene expression. In the last decade, with the increasing accessibility of high-throughput sequencing technologies, different methods have been developed

to identify miRNAs, most of which rely on pre-existing reference genomes. However, when a reference genome is absent or is not of high quality, such identification becomes more difficult. In this context, we developed BrumiR, an algorithm that is able to discover miRNAs directly and exclusively from sRNA-seq data. We benchmarked BrumiR with datasets encompassing animal and plant species using real and simulated sRNA-seq experiments. The results demonstrate that BrumiR reaches the highest recall for miRNA discovery, while at the same time being much faster and more efficient than the state-of-the-art tools evaluated. The latter allows BrumiR to analyze a large number of sRNA-seq experiments, from plants or animal species. Moreover, BrumiR detects additional information regarding other expressed sequences (sRNAs, isomiRs, etc.), thus maximizing the biological insight gained from sRNA-seq experiments. Additionally, when a reference genome is available, BrumiR provides a new mapping tool (BrumiR2ref) that performs an *a posteriori* exhaustive search to identify the precursor sequences. Finally, we also provide a machine learning classifier based on a Random Forest model that evaluates the sequence-derived features to further refine the prediction obtained from BrumiR-core. The code of BrumiR and all the algorithms that compose the BrumiR-toolkit are freely available at https://github.com/camoragaq/BrumiR.

Introduction

MicroRNAs (henceforth denoted by miRNAs) are small RNA molecules usually shorter than 25 nucleotides (nt), which have been identified as crucial regulators of gene expression mostly at the post-transcriptional level [1]. miRNAs are involved in a wide range of biological processes including cell cycle, differentiation, apoptosis and disease [2]. They have been the target molecules for a large number of important applications, more particularly in cancer where miRNAs have been shown to play important roles in driving or suppressing tumor spread [3], [4]. In plant species, unraveling host-pathogen interactions mediated by miRNAs may shed light on plant development and its relation with the environment, both essential knowledge that can lead to the discovery of new biotechnological products for the agricultural industry [5], [6].

Since the first classification and annotation of miRNAs in C.elegans [7], [8], thousands of miRNAs have been discovered in plants, animals and other eukaryotes. Most eukaryotic miRNAs are transcribed by RNA polymerase II [9]–[11], while some of them are transcribed by RNA polymerase III in animals [12]. Long precursor RNAs are folded into hairpin-like structures consisting of a terminal loop, an upper stem, the miRNA duplex region, a lower stem and two arms, and are processed in the cytoplasm generating the miRNA/miRNA* duplex which is subsequently divided into the star and the functional mature miRNA sequence [13]. Mature miRNA processing pathways differ between animals and plants. One major difference is the

length of the precursor sequences, with plant precursors longer than those of animals [14]. The mature miRNA sequences act as guides leading the RISC complex to target RNAs to regulate their expression by transcript cleavage or translation inhibition [15], [16]. Therefore, accurate prediction of known and novel miRNAs along with their targets is essential for increasing our understanding of the miRNA biology [4], [17]. However, it has proven difficult to accurately characterize and predict the miRNAs as well as their regulatory networks [18], [19].

Nowadays, a common experimental practice is to identify miRNAs and their expression patterns using next generation sequencing technologies (NGS) [20]. Commonly, NGS experiments are able to generate more than 20 million sRNA-seq reads, thus promoting the development of algorithms to transform and process such data into biological information [21].

Currently, there are two computational strategies for the discovery of miRNAs: 1) genome-based approaches that rely on the mapping of the sRNA-seq reads to a reference genome and subsequent evaluation of the sequences generating the characteristic hairpin structure of miRNA precursors [18]; 2) machine-learning approaches which rely on the biogenesis features extracted from the knowledge on miRNA sequences available in databases such as miRBase [22] and on the analysis of the duplex structure of miRNAs [23]. Genome-based methods, that have been updated at the pace of the evolving NGS technologies, are the most widely used tools in this field, and their results have populated the public miRNA repositories [21].

Such methods are the natural choice for the study of model species with high quality reference genomes available. However, it has been shown that most of the genome-based tools struggle with a high rate of false positive predictions when they rely only on the reference genome and do not leverage on sRNAseq data [18]. Additionally, a critical step of such tools is the use of genome aligners [24], [25] to map the sRNA-seq reads to the reference genome. Mapping short (<30 nt) and very similar sequences to a large, complex, and repetitive reference genome is however a difficult and error-prone task [26]. Genome-based methods are thus highly sensitive to the aligner selected as well as to the parameters employed and the thresholds chosen (*e.g.* number of mismatches allowed) in order to discard mapping artefacts generated from sequencing errors [27]. Furthermore, despite all the advancements in the sequencing technologies and *de novo* assembly methods, few complete genomes are available today, which is a recurring problem that researchers working on non-model species face [28]. The lack of a high quality reference genome thus reduces the possibilities for discovering novel miRNAs [23]. Genome-based methods such as miRDeep [29], miRDeep2 [30], and miR-PREFeR [31] are included in this group.

On the other hand, new methods such as miReader [32], MirPlex [33], and mirnovo [23], in particular using machine-learning approaches, were specifically developed as an alternative to discover miRNAs in species without a reference genome. In the case of mirnovo, the initial step involves the clustering of the sRNA-seq reads performing an all-vs-all read comparison that is followed by a subsequent

classification of the clusters into putative miRNAs using pre-trained models. The performance obtained by such methods on well-annotated species is comparable to those achieved by genome-based methods [18]. However, relying exclusively on annotated miRNAs for training machine learning models may introduce a bias towards the identification of well-characterized miRNAs over species-specific ones [21]. Nonetheless, machine learning methods have demonstrated that it is possible to discover miRNAs using only the sequence information present in the sRNA-seq experiment [23].

There remains however a need to go further in the development of algorithms for finding novel miRNAs in non-model species using only the sequence information. With this purpose in mind, the adoption of a special type of graphs called *de Bruijn* graphs may be considered. This is a widely used approach for the *de novo* reconstruction of genome or transcriptome sequences [34]. It therefore appears to be a plausible option for organizing, clustering and assembling the sequence information present in sRNA-seq experiments. However, accommodating the de Bruijn graph approach for the discovery of miRNAs involves the development of new methods to address the specific characteristics of sRNA-seq data. Indeed, mature miRNA sequences are short (18-24 nt), thus limiting the overlap length for building a de Bruijn graph which in turn impacts the global topology by inducing tangled graph structures. Moreover, miRNAs captured in a sRNA-seq experiment have variable expression, from low (few reads) to highly expressed (thousands of reads),

which may induce spurious graph connections that should be removed in order to isolate and detect both types of miRNAs. Finally, the sequencing errors present in sRNA-seq data further induce spurious connections and are harder to detect as compared to genomic data due to the variable expression and the shorter lengths of the miRNAs. Overall, using a de Bruijn graph to analyze sRNA-seq data and extract information from such data seems thus counterintuitive as mature miRNAs are captured full-length by the current NGS technologies. However, a de Bruijn graph has several interesting properties for the discovery of miRNAs, mainly due to the fact that it encodes all the sRNA-seq sequence information at once in a compact and connected representation (graph), without the need to perform an all-vs-all read comparison or mapping to a reference.

In this paper, we present BrumiR, a *de novo* algorithm based on a de Bruijn graph approach that is able to identify miRNAs directly and exclusively from sRNA-seq data. Unlike other state-of-the-art algorithms, BrumiR does not rely on a reference genome, on the availability of close phylogenetic species, or on conserved sequence information. Instead, BrumiR starts from a de Bruijn graph encoding all the reads and is able to directly identify putative miRNAs on the generated graph. BrumiR also removes sequencing errors and navigates inside the graph detecting putative miRNAs by considering several miRNA biogenesis properties (such as expression, length, topology in the graph). Along with miRNA discovery, BrumiR can also assemble and identify other types of small and long non-coding RNAs expressed

within the sequencing data. Finally, when a reference genome is available, BrumiR provides a new mapping tool (BrumiR2ref) that performs an exhaustive search to identify and validate the precursor sequences.

We extensively benchmarked BrumiR on animal and plant species using simulated and real datasets. The benchmark results demonstrate that BrumiR is very sensitive, besides being the fastest tool, and its predictions were supported by the characteristic hairpin structure of miRNAs. Finally, we also applied BrumiR to the discovery of miRNAs of *Arabidopsis thaliana* and identified three novel high-confidence miRNAs involved in root development. These putative miRNAs were not discovered before by any other software, thereby showing the potential of using different approaches even in the case where high quality genomes are available. The code of BrumiR is freely available at https://github.com/camoragaq/BrumiR.

RESULTS

BrumiR discovers mature miRNAs directly from the sRNA-seq reads.

The main idea behind BrumiR is that mature miRNAs can be discovered directly from the information contained in the sequenced sRNA-seq reads. To achieve this, BrumiR starts by building a de Bruijn graph directly from the sRNA-seq reads, using *k*-mers of size 14 and a depth of coverage of 50, then compacting all the simple nodes

thus leading to the unipath graph [35] (Figure 1.1, Methods section). The unipath graph encodes all the sequence information of the sRNA-seq experiment, including sequencing errors, adapters, and other types of sequences (Figure 1.1). The construction of the unipath graph allows to avoid entirely the alignment of the sRNA-seq reads to a reference genome. Following the unipath graph construction, BrumiR cleans the graph by removing tips (dead-end nodes) with low expression/abundance (KM < 5), which are usually generated from sequencing errors (Figure 1.2). One feature of the miRNA biogenesis is that after Dicer cleavage, the mature miRNA is the most abundant of the three by-products and when it is sequenced, it has a uniform expression along its sequence [29]. Therefore, BrumiR expects that the neighbor elements within a particular putative miRNA will have similar expression. BrumiR checks all neighbor connections (arcs), and deletes any connection with a relative expression difference larger than 3 fold (Figure 1.3, Methods section), and the new graph is cleaned again by removing tips (Figure 1.4). Clusters of unipaths (connected components) with topologies related to sequencing errors are also removed (Figure 1.5, Methods section).

BrumiR attempts to re-assemble all unipaths within a connected component (CC) of the graph, and those with between 18 and 24 nt are classified as putative miRNAs, while longer re-assembled unipaths (>24 nt) are classified as other longer sequences (Figure 1.6). BrumiR then restores missing connections by re-clustering the putative miRNAs performing an all-vs-all comparison. The most expressed miRNA is selected

as the representative of the cluster (Figure 1.7) and the remaining members are classified as potential isomiRs (Figure 1.7). The final BrumiR step uses the RFAM database [36] to discard predicted miRNAs matching to other classes of RNA (*e.g.* Ribosomal genes, Figure 1.8). We build a 16-mer database using RFAM database excluding any reference to known miRNA sequences, in a similar way as mirnovo does [23]. As an example, BrumiR reduces the input sRNA-seq data by five orders of magnitude generating less than 1,000 putative mature miRNAs (24 million input reads to 966 miRNA candidates, see Figure 1.10). Finally, BrumiR outputs several FASTA files with all predicted mature miRNAs, all longer RNAs, putative isomiRs, other sRNAs (RFAM comparison), and a table with expression values for each predicted miRNA. Additionally, BrumiR outputs the final graph in GFA format, which can be explored using Bandage [37] (Figure S11).

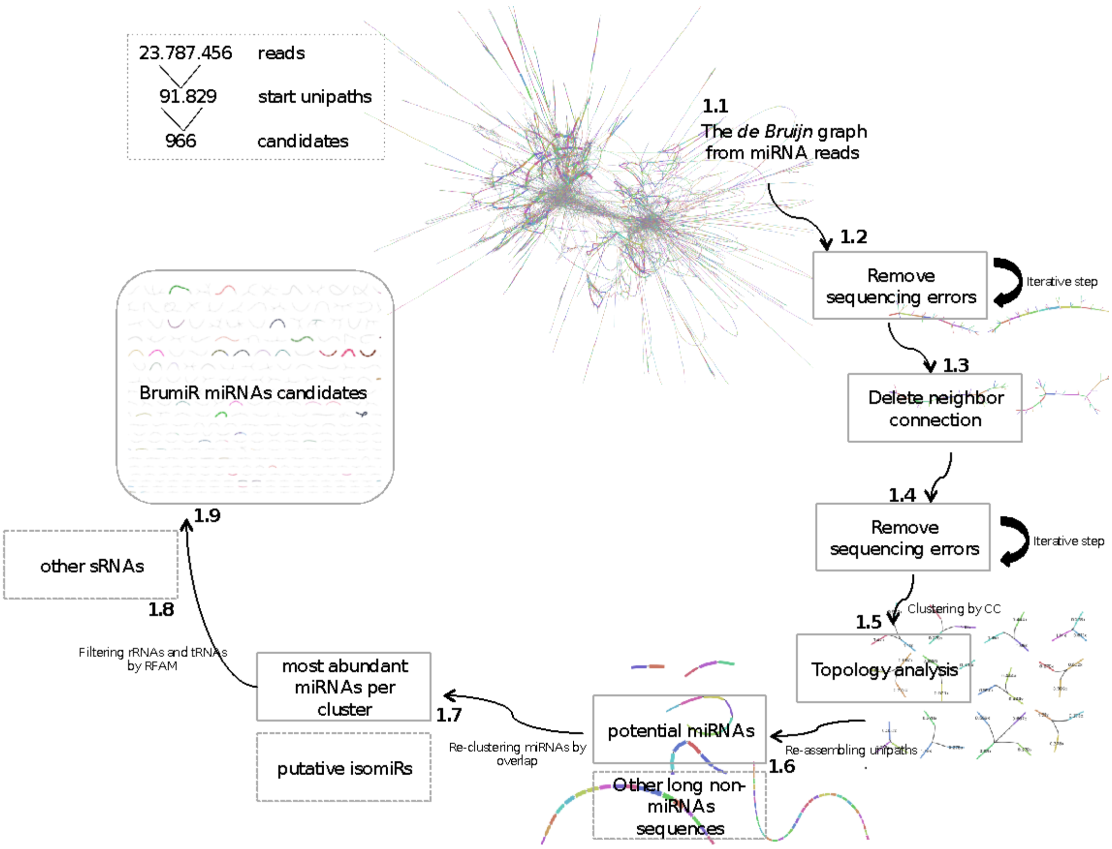

Figure 1. **BrumiR algorithm**. Different steps of BrumiR to discover miRNAs from sRNA-seq data. **1.1** De Bruijn graph step, **1.2** Tips removal iterative step, **1.3** Delete neigbor connection step, **1.4** Tips removal step repetition, **1.5** Topology analysis step, **1.6** Re-assembling unipaths by CC step, **1.7** Re-clustering by overlap step, **1.8** Filtering other sRNAs by RFAM step, **1.9** BrumiR candidates catalog.

BrumiR achieves the highest accuracy on simulated data.

To evaluate the performance of BrumiR, we applied it to discover mature miRNAs on simulated sRNA-seq reads from 10 animal and 10 plant species (Figure 2A). We compared BrumiR to the state-of-the-art genome-based miRNA discovery tools miRDeep2 [30] and miR-PREFeR [31], which were developed specifically for animal (miRDeep2) and plant (miR-PREFeR) species. For each tested species, we generated two synthetic datasets with different error-rates (0.01 and 0.02) using the miRsim tool implemented and provided by the BrumiR toolkit

(https://github.com/camoragaq/miRsim). To simulate the reads, we used (i) the high-confidence miRNAs annotated in the miRBase database [22], (ii) sequences from the RFAM database (v14.1) [38] to simulate possible fragments from other known types of RNAs present in the sRNA-seq data, and (iii) random genomic sequences for each of the species included in the benchmark (see the Methods section). A total of 20 datasets with an average of 13.6 million reads were simulated. The list of simulated miRNAs was considered as the ground truth, and benchmark metrics (Figure 2C) were computed to assess the performance of BrumiR and of the other software (See Methods section) (Supplementary Table S2).

BrumiR recovered more mature miRNAs than the others, on average 97% (opposed to 58% and 66% for miRDeep2 and miR-PREFeR, respectively), and presented the highest average recall across all the simulated datasets (Figure 2B). BrumiR recovered more than 90% of the simulated mature miRNAs in 19 of the 20 simulated datasets (Figure 2B). In particular in the *H. sapiens* and *D. melanogaster* datasets, BrumiR recovered 1,5X and 2,5X more candidates than MiRDeep2 (Figure 2B). As concerns precision, BrumiR tended to generate more putative candidates than MiRDeep2 (median 659 vs 332) and less than MiR-PREFeR (median 474 vs 649). The slightly higher number of BrumiR candidates resulted in lower average precision than miRDeep2 for animal species (0.51 vs 0.65), but was significally higher as compared to MiR-PREFeR for plants (0.71 vs 0.43). The lower precision achieved in animal species might be due to the fact that BrumiR does not use the hairpin structure filter

employed by miRDeep2. If we consider both precision and recall (F-Score), BrumiR was the top performer in 17 of the 20 datasets evaluated (Figure 2C). With animal species, BrumiR always reached a higher F-score than miRDeep2 except for *M. musculus*. With plant species, BrumiR was better to miR-PREFeR on most datasets, BrumiR reached a higher F-Score in 9 of the 10 datasets (Figure 2C).

In terms of computational time, BrumiR was the fastest method. In particular, BrumiR core was on average 21X faster than miRDeep2 and 6X times faster than MiR-PREFeR (see Table S3). The speed of BrumiR relies on efficient alignment-free and graph-based approaches.

Overall, we demonstrated with simulated data that BrumiR discovers putative mature miRNAs without a reference genome across different eukaryotic species achieving the highest accuracy and computational efficiency.

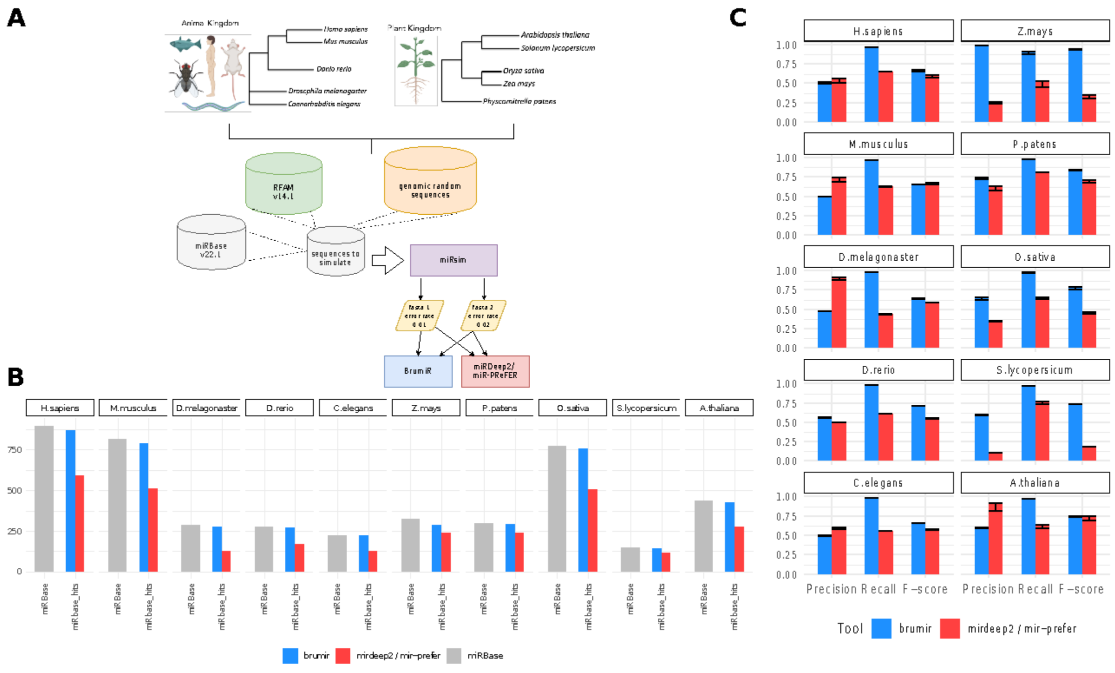

Figure 2. Synthetic benchmarking between BrumiR and miRDeep2. A) Workflow and species selected, **B)** miRBase input vs miRNA true positive predictions for each tool (2 samples), **C)** Benchmarking metrics for all datasets tested, the error bar indicates the distance between the 2 replicates.

The hairpin structure of mature miRNAs is found in most of the BrumiR candidates.

In order to assess the performance of BrumiR on real data, we collected public datasets for the same plant and animal species evaluated in the synthetic benchmark (Figure 2A). On average, 15.4 and 18.2 raw million reads were used for the animal and plant datasets (Supplementary Table S4), respectively. The predictions of BrumiR were compared against those of the state-of-the-art tools encompassing reference and *de novo* based methods [23], [30], [31], after testing some of the most used miRNA discovery tools, we selected the best performer (Supplementary Table

S5, Supplementary Figure S9). In particular, we included mirnovo that similarly to BrumiR can discover mature miRNAs directly from the reads. Before running the tools, low-quality reads were removed using fastp [39] (~10%, see Methods section). All the predicted miRNAs for each tool were annotated using the miRBase database to identify known and novel predictions. On average, BrumiR predicted ~450 putative mature miRNAs for the animal species, which was ~0.8X higher than the miRDeep2 candidates and 5.6X lower than the candidates predicted by mirnovo (Figure 3A1). For plant species, BrumiR predicted on average ~700 putative mature miRNAs, which was 4.7X lower than the candidates predicted by mirR-PREFeR (3,248 on average), and 5.3X higher than the predictions of mirnovo (131 on average) (Figure 3A1). A comparison using the miRBase [22] annotated miRNAs revealed that BrumiR shared more candidates with miRDeep2 and miR-PREFeR than with mirnovo (Figure 3A2). However, an important fraction (on average more than 70%) of the miRBase-annotated candidates were exclusive to each tool (Figure 3A2), which summarizes the complexity of miRNA discovery.

Considering mirGeneDB for animals and miRBase-annotated candidates for plant species as the ground truth, we computed precision, recall, and F-Score for all the evaluated tools (Figure 3B, Method section). BrumiR achieved an accuracy (F-Score) better for animals and plants than the one obtained by the other software (Figure 3B3). Moreover, BrumiR consistently reached the highest recall for most of the datasets evaluated (Figure 3B2). The precision values of BrumiR were slightly lower

for some datasets (Figure 3B1) in comparison with methods based on a reference genome such as miRDeep2, which has better precision due to the fact that the predictions are more conservative than the *de novo* methods (Figure 3B3). However, on average, BrumiR reached the highest precision (~0.44) on animal species, and also on plant species (~0.43). For the animal benchmark, we used the mirGeneDB database [40] and for plants, we used miRBase [22]. MirGeneDB is a manually curated database which has fewer entries compared to miRBase (17599 vs 48885) but has more reliable miRNA sequences. Unfortunately, the number of plant miRNA annotations hosted in mirGeneDB is not enough to use it as the ground truth. We therefore kept miRBase for plant species.

We also compared BrumiR-core to de Bruijn graph transcriptome de novo assemblers (Trinity and Velvet, see Methods) [41], [42] in order to assess the performance of a pure de Bruijn graph approach for miRNA discovery. We can observe that the de novo transcriptome assemblers generated on average 40X and 4X more candidates than BrumiR, for Trinity and Velvet respectively (Supplementary Table S7). In general, the huge number of contigs generated by the transcriptome assemblers, even after filtering them by length, were poorly matched to the miRBase entries (1,2% and 36%, indeed). On the other hand, BrumiR matched the miRBase entries at a rate of 1 of every 2 candidates (52% precision average). As expected, we can conclude that most of the contigs generated by a pure de Bruijn graph transcriptome assembler are poorly related to miRNA sequences. This was expected

because they are developed for mRNAseq analysis and do not consider the complexities of the sRNA seq data like BrumiR.

In summary, this experiment showed that BrumiR and all the downstream steps it performs after the de Bruijn graph construction are essential for miRNA discovery.

The BrumiR toolkit also provides a tool to determine the hairpin loop of miRNA precursor sequences, which is the main structural feature of miRNAs [43]. BrumiR2reference maps the BrumiR predicted mature miRNA to the reference genome using an exhaustive alignment (See Methods section), generates precursor sequences, computes its secondary structure, and checks the hairpin structure using a variety of criteria inferred from analyzing more than 30,000 miRBase precursor sequences from animal and plant species (see Methods section). We used BrumiR2reference as a double validation for all the predicted mature miRNAs generated by BrumiR for the animal and plant datasets (Figure 3C). On average, BrumiR2reference identified a valid precursor sequence having the characteristic hairpin structure for over 60% of the BrumiR candidates (Figure 3C).

In terms of speed, BrumiR core was the fastest tool. BrumiR was on average 19X and 38X times faster than miRDeep2 and miR-PREFeR, respectively (See Table S6).

Overall, we demonstrated that BrumiR is a competitive tool for discovering mature miRNAs without a reference genome. We showed that it was the most sensitive on most of the datasets tested. The performance of our method was not only faster, but also better or comparable to the state-of-the-art tools. Moreover, we also provide a

new mapper approach to be used when a reference genome is available, to further verify if a precursor sequence of the predicted mature miRNA is present in the genome. BrumiR therefore represents a reliable alternative for the discovery of mature miRNAs in model and non-model species with or without a reference genome.

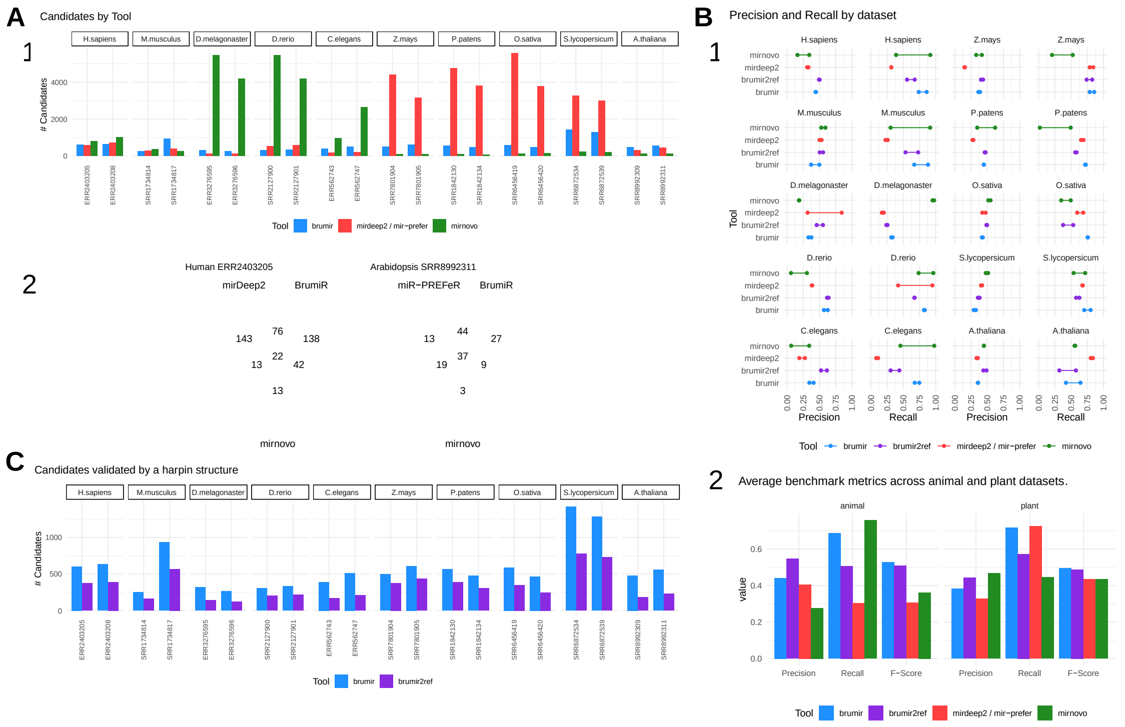

Figure 3. Real dataset benchmark of BrumiR and state-of-the-art tools. A) Number of predictions by tool for all the datasets and the overlap between them for 2 datasets (1 for animal and 1 for plant); **B)** Benchmarking metrics computed using miRBase annotated miRNAs, precision and recall for each dataset; and average metrics, including F-score. **C)** BrumiR candidates validated by Hairpin structure (BrumiR2Reference).

Using a supervised machine learning approach to refine the BrumiR-core prediction.

To further refine the prediction of BrumiR, especially in the plant datasets, we developed and implemented a supervised machine learning method based on a Random Forest model [44]. The Random Forest model classifies the BrumiR candidates into putative miRNA or random sequences. The Random Forest model is composed of 19 features, of which 16 are inferred directly from the 15-mer sequences of each BrumiR candidate and 3 are derived from the nucleotide composition observed on reference mature miRNA sequences. In order to use a confident input and reduce as much as possible the number of false predictions, we employed the manually curated database mirGeneDB [40] for training with animal species, while for plant species we kept miRBase due to the low number of miRNA plant entries present on mirGeneDB. The 16 derived features are GC content (gc), GC skew content (gcs), CpG content (cpg), sequence complexity by Wootton & Federhen values (cwf), sequence Shannon entropy (ce), sequence complexity of Markov model values (cm1,cm2,cm3), sequence complexity by Trifonov values (ct3,ct4,ct5,ct6) and sequence complexity linguistic values (cl3,cl4,cl5,cl6) [45]. The nucleotide compositions are the 6-mer, 7-mer, and 8-mer observed frequency of mature miRNA sequences from the reference miRNA databases (MirGeneDB or miRBase). The features were computed on a 15-mer basis to classify any length of miRNA candidates (18-22 base pairs). A total of 35570 15-mers were derived from

the MirGeneDB, and all the 19 features were computed for each. The top-5 most informative features for discriminating miRNA from random sequences were 8-mers, 7-mers, 6-mers, CpG content, GC content, and the complexity of Markov models (Figure 4A). The benchmark results show that the Random Forest classifier achieves an accuracy of 90%, a precision of 87%, and a recall of 94% for discriminating animal miRNA 15-mers from random ones (Figure 4B). The miRBase model achieves an accuracy of 90%, a precision of 87%, and a recall of 93% for discriminating plant miRNA 15-mers from random ones (Figure 4B). We used the Random Forest classifier to further refine the BrumiR prediction on animal and plant real datasets. Similar to BrumiR2reference, BrumiR-RF reduced the number of BrumiR-core candidates (Figure 4C) but without the need of a reference genome. We observe that most of the discarted candidates were likely false-positives (considering the reference miRNA database is the ground truth), which results in an improved precision without affecting the recall (Figure 4D). In summary, the BrumiR-RF classifier allowed us to increase the precision of BrumiR without affecting its overall recall, and without the need of a high quality reference genome. The BrumiR-toolkit now provides tools for handling all kinds of miRNA-related information for an enhanced miRNA prediction discovery.

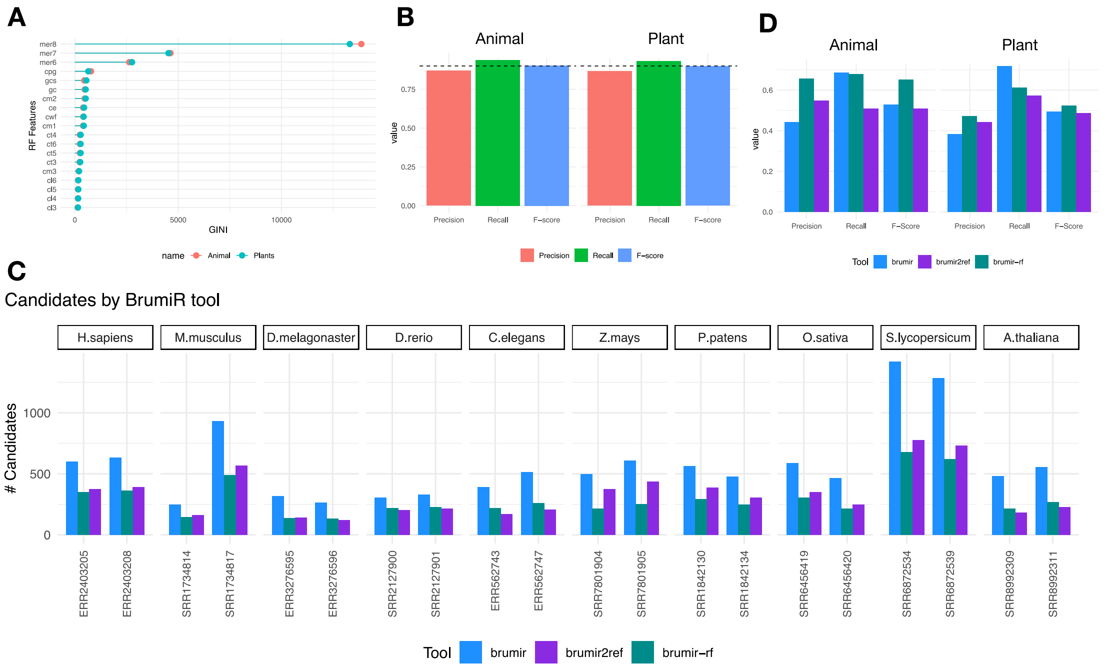

Figure 4. **Improving the precision of BrumiR by using a Random Forest classifier**. A) Most informative features of the Random Forest classifier for animal and plant species. B) Benchmark metrics of the Random Forest Model to discriminate 15-mer miRNA mature sequences from random 15-mer sequences. C) Number of candidates by BrumiR tools (BrumiR-core, BrumiR2reference, BrumiR-RF). D) Average metrics across animal and plant real datasets for all BrumiR tools.

Discovering novel miRNAs from sRNA-seq data of *A. thaliana* roots using BrumiR.

A. thaliana is one of the best characterized model organisms, and the first plant species in which miRNAs were cloned and sequenced [46]. To date, 436 mature miRNA sequences are included in the miRBase database. Most of these miRNAs have been identified by studies addressing the sRNAome of different plant organs [47], cell types [48], or responses to biotic or abiotic stress using sRNA-seq [49], [50] (Hsieh et al., 2009).

We sequenced sRNA-seq libraries from the roots of *A. thaliana* after different time points during vegetative development (see Methods section) (Figure S12) to demonstrate the potential of BrumiR to discover novel mature miRNAs in a known biological context. BrumiR was run independently for each condition and replicate. The day 5 samples were excluded because of the low number of reads when compared to the other samples (Supplementary Table S8). BrumiR predicted, on average, 1,160 mature miRNAs per sample, which were further refined to 719 using the BrumiR2ref tool. To take advantage of our experimental design, we considered as a putative miRNA the ones present in the three replicates (core predictions) [51] (Figure 4A). Novel miRNAs were identified using the following steps: First, predictions were classified as known miRNAs by comparing with miRBase (141 known miRNAs out of a total of 159 miRNAs already described for *A. thaliana* in miRBase). These known miRNAs were put aside to explore the sensitivity of BrumiR in detecting novel putative miRNAs. We then clustered the remaining putative miRNAs into three stages: early, late, and constitutive (Figure 4B). The days 9, 13 and 17 represent an early stage of the plant development; days 17, 21 and 25 represent a late stage of the plant development [52], and the putative miRNAs expressed in all conditions represent the constitutive category (Supplementary Table S8). A total of 21 putative novel miRNAs were identified, and a manual curation was carried out revising all the criteria to validate and annotate miRNAs in plants [51]. We discovered two novel miRNAs candidates that fulfill all the recommended

criteria to annotate miRNAs in plants (Figure S10, Table S8). According to the revised criteria, confirmation by blot of the expression of the miRNA or miRNA* is disallowed, and it is suggested that validation of miRNA expression should be based on sRNA-seq reads only. In this way, these two curated novel miRNA candidates are supported directly from the sRNA-seq libraries and are expressed in all replicates in all conditions [51].

One of the curated novel miRNAs candidates (miR-8) is located in Chromosome 5 (Figure 4C), this miRNA locus has not been previously discovered because its mature sequence maps to multiple chromosomes, and is therefore discarded by genome-based tools [26].

In an exploratory analysis to shed light on the potential targets of these novel miRNAs, we conducted an *in silico* target transcript prediction using the 1 algorithm [53] (Supplementary Table S10). EXO84b (AT5G49830) was found to be one of the top genes regulated by this novel miRNA miR-8 (Supplementary Table S9). In A. thaliana, it has been demonstrated the importance of EXO84b in the development of trackeary elements or vassel xylem system which is essencial for water and nutrient transport of vascular plants [54]. EXO84b is expressed over all days but significantly abundantly expressed in the last days, and its differential accumulation between root zones is related to emerging patterns of lateral roots and hair formation from trichomes [55].

We have also explored the known miRNAs identified by BrumiR in where we have found in almost all the samples, with a higly expression, the plant miRNAs that would be playing a key role in root specification and development [56].

It is plausible to say that these novel and known miRNAs may be involved in the fine-tuning of lateral root growth in the early stages of development.

These results highlight the value of the BrumiR toolkit for discovering novel and known miRNA candidates with functional impact on the organisms studied, even in the case where high quality genomes are available.

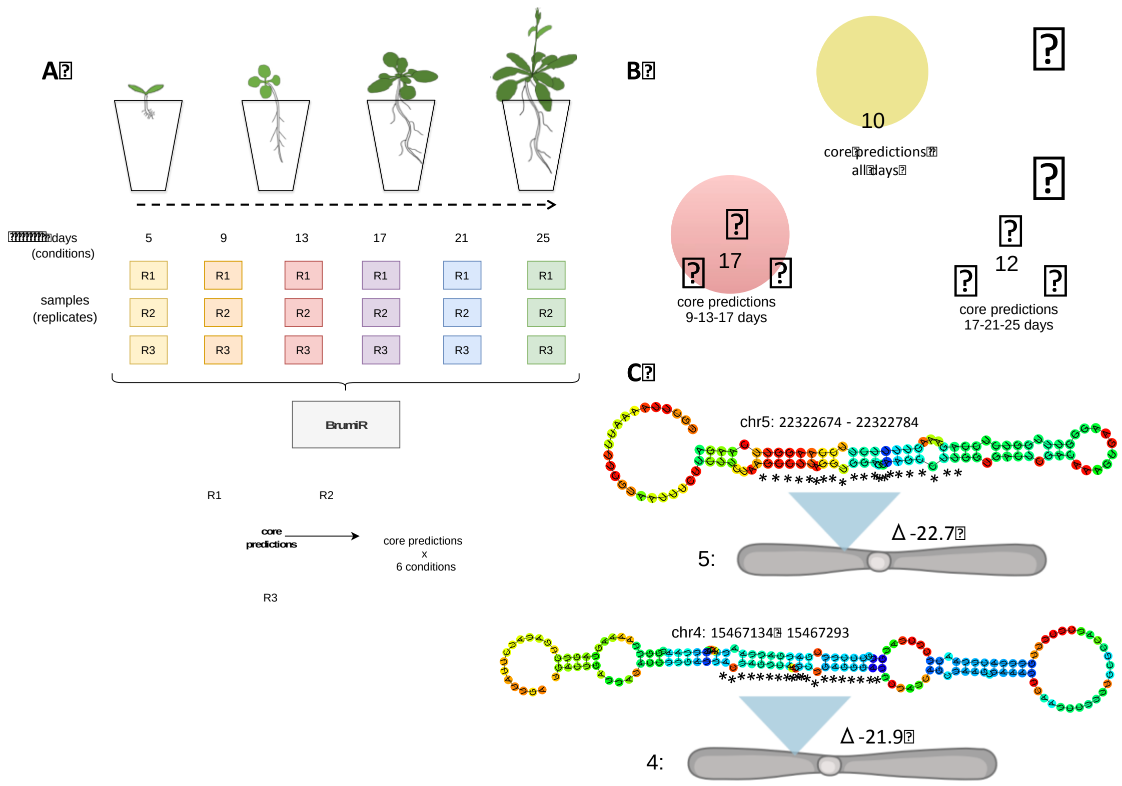

Figure 5. **Applying BrumiR on sRNA-seq from *Arabidopsis* root libraries. A)** Experimental design implemented; roots from *Arabidopsis* on a time-scale per day as conditions were sequenced in three technical replicates. BrumiR was used to analyze all sRNA-seq libraries, and conserved predictions by the three replicates were considered as a core by condition. **B)** Different combinations of root growth per day were analyzed together to identify novel putative miRNAs conserved in all conditions. **C)** We discovered 2 candidates as novel miRNAs that fulfill the current criteria to annotate miRNAs in plants. Moreover, they were supported directly from the sRNA-seq libraries and are conserved in all replicates in all conditions.

DISCUSSION

In this paper, we introduced and benchmarked the BrumiR toolkit, which was designed for enabling the identification of mature miRNAs in model and non-model species with or without a reference genome, encompassing the plant and animal kingdoms. The BrumiR toolkit implements the following algorithms: 1) a new discovery miRNA tool (BrumiR-core), 2) a specific genome mapper (BrumiR2ref), 3) an sRNA-seq read simulator (miRsim), and 4) a mature miRNA sequence classifier (BrumiR-RF). We demonstrated that BrumiR is capable of identifying mature miRNAs based only on the sequence information and generates results that are better or comparable to the state-of-the-art tools on simulated and real datasets. We further tested the usefulness of the BrumiR toolkit for discovering novel miRNAs potentially involved in the regulation of the root development of the extensively annotated *A. thaliana* genome.

Unlike the state-of-the-art tools, BrumiR starts by encoding the sRNA-seq reads using a de Bruijn graph. This avoids the read mapping stage and the dependency on

previous miRNA annotations. It also enables the identification of sequencing artifacts. A critical step of genome-based miRNA discovery tools is to identify the precursor sequence when a reference genome is available. BrumiR introduces a new mapping approach, BrumiR2reference, which scans every possible hairpin precursor in the genome, when such is available, for all the BrumiR predictions. As the hairpin structure is determined using the predicted mature miRNA instead of the reads, this alignment can support mismatches and indels and handles the case of multi-mapped candidates (due to repetitive regions of the genome). Such features distinguish BrumiR from the current genome-based methods.

Discovering miRNAs in non-model species is one of the limitations of the current methods. One exception is mirnovo, which similarly to BrumiR can predict miRNAs using only the sRNA-seq data, and a specific training set for animal and plant species. We thus compared its performance to the one of BrumiR. Our results show that mirnovo is very conservative, generating few predictions in comparison to BrumiR. This could be due to the low number of entries of plant miRNAs in miRBase because the mirnovo approach is based on miRNA families present in this database. However, miR-PREFeR generates a larger number of candidates in plant species. The higher number of predictions of miR-PREFeR results in lower precision in most of the evaluated datasets, on which BrumiR obtained the highest F-score in 6 out of 10 cases. In animal species, BrumiR has a lower precision compared to miRDeep2 in some of the datasets, but considering the F-score, BrumiR obtains the highest rate in

all the datasets. We examined possible piRNA sequences present in the sample of *Mus musculus* SRR1734817 to see if this high number of candidates was due to wrong predictions but no relationship was found (Supplementary Table S13).

When we use the complementary tools of BrumiR, BrumiR2reference and BrumiR-RF, BrumiR exceeds its performance reaching the highest metric in 16 of the 20 datasets, improving the precision and reducing the number of candidates without sacrificing the recall rates.

In an attempt to increase the accuracy of BrumiR, we developed the BrumiR2reference and BrumiR-RF tools, thus reducing the number of false-positive miRNAs without sacrificing recall. We implemented a supervised Random Forest classifier trained on the high confidence mature sequences available in the manually curated database mirGeneDB. The latter led to an important improvement in the accuracy of BrumiR even in the case when a reference genome is not available. It is important to observe that the miRNA annotations remain incomplete and although miRBase is the main repository for miRNAs, it cannot be considered the gold standard for most species (many of the entries have not been correctly validated, for example) [18]. For this reason, we used mirGeneDB but the predictions of BrumiR are not based on miRBase nor on mirGeneDB in any step of the algorithm. These tools can be used in a posterior analysis to verify the miRNAs inferred in case of not having any reference genome as a post-prediction step in a complementary way.

In terms of computational resources and usability, BrumiR is the fastest method and provides a stand-alone package for running locally all the analyses. It further generates an output that is compatible with the bandage software [37], which can be employed to visualize and explore the results of BrumiR in a user-friendly way.

Moreover, BrumiR reports other sequences expressed in the sRNA-seq data among which are putative isomiRs and longer non-coding RNAs, thereby providing additional biological insight.

Finally, we tested the effectiveness of BrumiR on sequenced sRNA-seq libraries from the roots of *A. thaliana*, and were able to discover 2 novel putative miRNAs based on the very conservative criteria proposed in [51], showing the potential of it being used alone or in combination with other methods.

In summary, we present a new and versatile method that implements novel algorithmic ideas for the study of miRNAs that complements and extends the currently existing approaches.

MATERIALS AND METHODS

Building a de Bruijn graph for sRNA-seq data.

BrumiR starts by building a compact de Bruijn graph from the sRNA-seq reads given as input. De Bruijn graphs are a widely used approach in the genome assembly problem [34]. BrumiR uses this graph to organize, detect, and exploit the sequence information of sRNA-seq experiments. BrumiR takes as input sequencing files in

FASTA or FASTQ formats. The sequencing data can be cleaned, using a fastq pre-processor [39] (*i.e.* fastp), to remove adapter sequences and trim low quality bases. BrumiR employs the BCALM [35] tool to build a de Bruijn graph from the sRNA-seq reads. BCALM uses a node-centric bi-directed de Bruijn graph where the nodes are *k*-mers, that is words of length *k*, and an arc between two nodes if the *k-1* suffix of one node is equal to the *k-1* prefix of the subsequent node, representing an exact overlap of *k-1* bases [35]. A critical parameter of any de Bruijn graph approach is the *k*-mer size [57]. We observed that the length of all mature miRNA sequences stored in the miRBase database (v21) [22] fluctuates between 18 to 24nt (Supplementary Figure S1). To determine the optimal *k*-mer size for BrumiR, we compared the performance of BrumiR using different *k*-mer sizes (14-16-18-20-22). The benchmark shows that the optimal *k*-mer size for BrumiR is 14 (Supplementary Table S1, Supplementary Figure S2), because it allows for a better handling of the sequencing errors and enables a more sensitive clustering of identical miRNA candidates, even when comparing with 18-mers which is the *k*-mer size expected for a mature miRNA sequence (Supplementary Figure S3). We thus empirically set the *k*-mer size equal to 14. BCALM compacts the nodes of the de Bruijn graph into maximal unipaths by gluing all the nodes of the graph with an in-degree and an out-degree equal to one, thus generating the so-called *unipath graph* [35]. The unipath graph is the starting point of BrumiR (Figure1A). Notice that the unipath graph generated by BCALM does not represent what is expected for a set of mature

miRNAs (one connected component for each miRNA) and therefore further graph operations are needed. BrumiR uses a minimum *k*-mer frequency (KM value) of 5 and all *k*-mers with lower frequency are ignored, without losing most of the information contained in the sequencing reads (Supplementary Figure S4). Additionally, we ran BrumiR-core using different depth coverages in order to define the optimal coverage resolution for the resulting de Bruijn graph (Supplementary TableS8). The comparison shows a convergence in the number of candidates at a depth coverage of 50, and because of this, we set this parameter at this value (Supplementary Figure S5).

Removing sequencing errors from the unipath sRNA-seq graph.

BrumiR deletes from the unipath graph all the nodes that have only one connection (degree equal to 1), known as dead-end paths or tips [58]. Usually, these nodes have a low abundance value associated to them (KM less than or equal to 5, the default parameter). Moreover, BrumiR deletes isolated nodes (degree equal to 0) having a low abundance; isolated nodes highly expressed are however conserved for further analysis. All these nodes are likely artifacts generated from sequencing errors because they are not deeply expressed in the sRNAs-seq reads [59]. BrumiR iterates this step 3 times in order to prune and clean the unipath graph (polishing). This operation, called 'tip removal', edits the original unipath graph, and therefore a new unipath graph with a new structure is generated (Figure 1B).

An expressed mature miRNA has uniform coverage.

The unipath graph of a set of miRNAs from an sRNA-seq experiment has non-uniform coverage as different miRNAs and other elements may be connected in a single big component (Figure 1.1). BrumiR evaluates each connection of the unipath graph to identify those that link two nodes with a large expression difference. According to the miRNA biogenesis, after a stable miRNA precursor is cleaved by Dicer, among its three products, the miRNA mature sequence is the most abundant and when it is sequenced, it has a uniform expression along its sequence [29]. Thus due to miRNA biogenesis, it is possible to capture the complete miRNA mature sequence having a homogeneous expression [30] directly from the sRNA-seq experiments. BrumiR expects a similar KM value for *k*-mers originating from the same mature miRNA gene. Accordingly, if we observe two connected nodes that show a big difference in their abundance values, this connection is deleted and we keep the nodes unconnected. In particular, two unipaths $U=\{a,b\}$ connected in the graph have a KM value associated to them that represents their coverage from the reads information. BrumiR scans all the neighbor connections and if the difference between their KMs is larger than three-fold, the connection is deleted ($Ui_{km}/Uj_{km} > 3$). In this way, BrumiR defines a relative threshold that will depend on each unipath neighborhood in the graph. Finally, BrumiR repeats the tips removal step to eliminate new low frequency isolated nodes (Figure 1C).

miRNAs and other sequences are captured in single connected components.

After the previous steps of BrumiR, a new unipath graph emerges, with a new structure. It is thus necessary to identify and classify the new connected elements within the graph (Figure 1). A connected component (CC) of a graph is a maximal strongly connected subgraph [60]. BrumiR computes the CCs of the unipath graph, and then each CC is processed independently to identify miRNA candidates as well as to discard other sequences present in the unipath graph.

BrumiR classifies low abundance non-linear topologies as sequencing artefacts.

BrumiR detects topologies that are potentially related to sequencing errors and thus unlikely to be miRNA candidates. The shapes of these topologies were identified by visual inspection of several unipath graphs and are described in detail in Figure S6. Usually they have low KM and are composed of lowly expressed branching nodes with 3, 4 or 5 connections to the principal structures in the graph (Figure S6). Moreover, we observed that the sequences contained in these topologies were usually redundant and contained in other linear and more expressed CCs. In this

way, we are not discarding relevant sequence information. BrumiR removes about 10% of the CCs in this step.

Re-assembling unipaths within each CC.

BrumiR re-assembles all unipaths present in the linear CCs by bundling the nodes with in and out degree equal to 1 into a new unipath. BrumiR classifies them into different types based on their length. The latter is the length of the sequence represented by the new unipath. All CCs having a length between 18 and 24 are stored as potential miRNA sequences. The CCs corresponding to an isolated node that have high KM (KM>50) are included in the latter group. CCs with lengths over 24 are classified as longer sequences or other types of genomic sequences captured along with the miRNAs. The longer sequences are put aside for later analysis. Moreover, BrumiR identifies circular CCs and branching CCs. The former are circular unipaths and the latter CCs with a high number of branching nodes. Branching CCs are not considered in the subsequent steps because they are likely sequencing errors (low abundance) or contamination present in the sRNA-seq data (Figure S7).

Re-clustering potential miRNAs.

After grouping unipaths by CCs, BrumiR builds an overlap graph to rescue the missing connections between potential miRNA candidates sharing an overlap with another candidate. First, BrumiR adds all the candidates as nodes of the overlap graph, then an all-vs-all k-mer comparison is performed using exact overlaps of length k=15. Candidates sharing an exact overlap are connected in the overlap graph. Then, the connected components are computed to identify clusters of miRNA candidates, and the most expressed candidate within each component is selected as the representative candidate of the cluster. The representative candidates are compared all-vs-all in a second overlap step that allows a maximum edit distance of 2, which is implemented using the edlib library [61]. BrumiR then builds a second overlap graph, computes again the connected components, and selects the most expressed candidate as the representative of each cluster. The other members of each connected component are classified as putative isomiRs and saved in a file for later analysis.

Identifying other expressed RNA sequences.

In sRNA-seq experiments, different types of RNAs are expressed, some of which, such as small non-coding RNA elements, may have similar length with miRNAs [62]. The RFAM database [38] is a collection of curated RNA families including three

functional classes of RNAs (non-coding, cis-regulatory elements, and self-splicing RNAs), which are classified into families according to their secondary structure and sequence information (Covariance Models) [36]. We downloaded 3,017 RNA families present in RFAM (v14.1) and excluded 529 miRNA families. The sequences of 2,488 RFAM families were concatenated (a total of 2,736,549 sequences) and used to build a 16-mer database with the KMC3 *k*-mer counter tool [63] ("-fm -n100 -k16 -ci5"). All distinct 16-mers with a frequency lower than 5 were excluded, leading to a total of 6,204,556 distinct 16-mers related to RNA elements. Additionally, we downloaded all the mature miRNA sequences from miRBase (v22.1) [22] and built a 16-mer database with KMC3 ("-fm -n100 -k16 -ci1 mature.fa.gz"). RFAM 16-mers matching 16-mers from the 16-mer mature miRBase database were excluded from RFAM, leading to a 16-mer RFAM database with a total of 6,204,487 distinct 16-mers. Finally, the BrumiR candidates (18-24 length) were matched to the 16-mer RFAM database, and matching candidates were excluded and reported as sequences potentially associated to other RNA elements. The BrumiR candidates passing the aforementioned filter are reported as the final list of miRNA candidates.

Identifying precursor sequences for BrumiR candidates (BrumiR2Reference).

Unlike current state-of-the-art tools that perform miRNA discovery by mapping the sRNA-seq reads to a reference genome, BrumiR generates candidates by operating directly on the sRNA-seq reads. The reduced list of potential BrumiR miRNA candidates permits the computation of a more exhaustive alignment than when mapping directly the sRNA-seq reads to the reference genome. BrumiR aligns each candidate to the reference genome using an exact alignment method that computes the edit distance [14] between two strings and thus support mismatches, insertions and deletions. The BrumiR2reference tool divides the reference genome in non-overlapping windows of 200bp (adjustable parameter), then the window is indexed using 12-mers and each miRNA candidate is matched in both strands (split at 12-mers). When a 12-mer match is found, an exhaustive alignment is computed between the window and the matching miRNA candidate. The alignment is performed using a fast implementation of Myers' bit-vector algorithm [61].

A miRNA candidate is stored as a hit if the alignment in the current genomic window has an edit distance less than or equal to 2. After scanning all the genomic windows, the vector of hits is sorted by miRNA-candidate; edit distance (0-2), and alignment sequence coverage. For a single miRNA-candidate, a maximum of 100 genomic locations (best hits) are selected. BrumiR2reference then builds a potential precursor

sequence for each selected hit using a strategy similar to the ones employed by miRDeep2 [30] and Mirinho [64]. BrumiR excises the potential precursor hairpin sequence from the flanking genomic coordinates of the reported miRNA candidate hits (mature sequence) in both strands. Potential precursor hairpin sequences of length 110 bp are built for animal species from both strands, while for plant species hairpin sequences of lengths 110, 150, 200, 250 and 300 bp are built from both strands [14]. Secondary structure prediction for all the potential precursor sequences is performed using RNAfold (v2.4.9) [65]. Secondary structures with a minimum free energy in the range of 15-80 kcal/mol are checked for a hairpin loop characteristic of miRNAs [43] (Figure S8). Structures with a hairpin loop composed of a single segment without pseudo-knot, multi-loops, external loops and with less than 5 bulges, 3 dangling ends, and 10 internal loops are classified as characteristic secondary structures of miRNA precursor sequences. The aforementioned filters were derived from analyzing the secondary structure of 38,589 precursor sequences stored in miRBase (v22.1) [22] using a modified version of the bpRNA program [66] (Figure S9).

Benchmarking BrumiR against transcriptome de Bruijn graph assemblers.

In order to determine the value of BrumiR for extracting miRNA candidates directly from a de Bruijn graph, we compared the BrumiR approach against two de Bruijn

graph transcriptome de novo assemblers, namely Trinity [41] and Velvet [42]. The benchmark was performed using 4 real datasets; including human and *Arabidopsis*. The seed length and minimum contig length for the transcriptome assemblers were fixed at 14-mer for all tools. Then, the contigs longer than 24 nt were filtered out for Trinity and Velvet. For BrumiR, we eliminated the last step using the RFAM database [36] information to filter out other kinds of sRNA sequences, and we used all the predictions to compare to the transcriptome de novo assemblers. Finally, the BrumiR candidates and contigs generated by Trinity and Velvet were mapped against the miRBase database (Blast search).

Benchmarking BrumiR using simulated sRNA-seq reads.

We simulated synthetic reads from animal and plant species, and compared the results of BrumiR to those obtained with the miRDeep2 [30] and miR-PREFeR [31] tools. The sRNA-seq reads were simulated using miRsim (https://github.com/camoragaq/miRsim), a tool that we developed specifically for simulating sRNA-seq reads from a list of known miRNA mature sequences. miRsim is based on *wgsim* (https://github.com/lh3/wgsim), which is a widely used tool for simulating short Illumina genomic reads. miRsim includes functionalities specific of sRNA-seq reads such as variable depth/coverage and shorter read lengths. miRNA mature sequences were obtained from miRBase [22] for animal (High Confidence) and plant species. Additionally, to simulate the typical fragments contained in real

sRNA-seq data, we included sequences from the RFAM database (v14.1) [36] and random genomic sequences from the genomes for each of the species included in the benchmark (10% of the sequences for RFAM and genomic sequences, respectively). The animal species that we considered were: *Homo sapiens, Mus musculus*, *Drosophila melanogaster, Danio rerio*, and *Caenorhabditis elegans*, while the following plant species were included: *A. thaliana*, *Oryza sativa, Physcomitrella patens, Zea mays,* and *Solanum lycopersicum*. Supplementary Table S2 provides further details (*i.e.* number of reads, number of mature miRNAs *etc.*) for each simulated dataset. MiRDeep2 was run on the animal datasets with the default parameters and using the score suggested by the developers, providing the respective reference genome. Similarly, miR-PREFeR was run with the default parameters on the plant datasets. BrumiR was run with the default parameters on both the animal and plant datasets. The miRNA annotations were not included for the genome-based tools in order to make a fairer comparison with BrumiR which does not use this information. The list of simulated miRNAs was considered as the ground truth, and precision, recall and F-Score quality metrics were computed to assess the performance of each discovery tool. The benchmark metrics were defined as follows:

$$Recall = \frac{TP}{TP + FN}$$

$$Precision = \frac{TP}{TP + FP}$$

$$F\text{-}score = 2 * \frac{(Recall * Precision)}{(Recall + Precision)}$$

where:

TP= true positive elements predicted as miRNAs present in the miRBase input list.

FP= false positive elements predicted as miRNAs but not present in the miRBase input list.

FN= false negative elements not predicted as miRNAs, but that were present in the miRBase input list.

Benchmarking BrumiR using real sRNA-seq reads.

We downloaded publicly available sRNA-seq data for the plant and animal species listed in the synthetic benchmark, and two datasets for each species were included (Supplementary Table S4). We wanted to benchmark BrumiR in a simple but exhaustive way by selecting the top performer for genome-based and genome-free methods. We benchmarked some of the most used prediction tools in a reduced version of the real dataset, and the results were conclusive to select the best methods (Supplementary Table S5, Supplementary Figure S10). We included mirnovo [23], a tool that can discover miRNAs without a reference genome. The predictions of BrumiR were benchmarked along with MiRDeep2 (v2.0.1.2) [30] and mirnovo for the animal datasets. Similarly, miR-PREFeR [31] replaced MiRDeep2 for the plant datasets (Supplementary Table S4).

The stand-alone packages of BrumiR, miRDeep2 and miR-PREFeR were used to discover miRNAs in all datasets. The software mirnovo was run using its web version because the stand-alone package was not available and the developer recommends the use of the web version instead. The miRNA discovery was performed for each sample independently using default parameters for MiRDeep2, miR-PREFeR and mirnovo. In particular, we used the scripts provided by miRDeep2 and miR-PREFeR to map the reads to the reference genome, and the predictions for these tools were performed on the resulting alignment files. The mirnovo predictions were done using the animal and plant universal panel respectively, as recommended when the reference genome is not available. BrumiR was run using the command line and parameters provided in the Supplementary Section 1. Moreover, the predictions of BrumiR were refined using the BrumiR2reference tool on the available reference genome of the selected species (Supplementary Table S4). Benchmark metrics (precision, recall, and F-Score) were computed as before but considering all the annotated mature sequences present in mirGeneDB for animal and miRBase (v22.1) for plant species, as the ground-truth.

A Random Forest model to refine the predictions of BrumiR-core.

The Random Forest model is composed of 19 features, of which 16 are inferred directly from 15-mer sequences of each BrumiR candidate and three derived from

the nucleotide composition observed on reference mature miRNA sequences (miRGeneDB and miRbase) [22], [40]. The nucleotide composition was analyzed using the 6-mer, 7-mer, and 8-mer observed frequency of the mature miRNA sequences of reference miRNA databases (MirGeneDB and miRbase). The features are computed on a 15-mer basis to classify any length of miRNA candidates (18-22 base pairs). A total of 35570 15-mers were derived from the MiRGeneDB, and all the 19 features were computed for each. A matching amount of 15-mer random sequences was generated, and all the 19 features were computed for each. The whole training and evaluation dataset comprised 71.140 15-mers of the two classes (random and mature miRNA sequences). The training and evaluation of the random forest were performed using 75% and 25%, respectively. The performance of this classifier on all the real datasets is reported in Supplementary Table S9. Finally, a Rnotebook including all the steps required to build the Random Forest model is available at the BrumiR GitHub repository here: https://github.com/camoragaq/BrumiR/tree/master/brumir-rf

miRNA discovery from *Arabidopsis* root samples.

A. thaliana Col-0 seedlings were grown hydroponically on Phytatrays on 0.5X Murashige and Skoog medium (Phytotechnology Laboratories, cat. M519) under long-day conditions (16h light and 8h dark) at 22 °C. Total RNA was isolated from plant roots after 5, 9, 13, 17, 21, and 25 days post-germination using the mirVana

miRNA Isolation Kit (Thermo Fisher Scientific, cat. AM1560). RNA concentration was determined using the Qubit RNA BR Assay Kit (Thermo Fisher Scientific, cat. Q10210), and integrity was verified by capillary electrophoresis on a Fragment Analyzer™ (Advanced Analytical Technologies, Inc.). The indexed sRNA libraries were built employing the TruSeq small RNA Sample Preparation Kit (Illumina, Inc.) following the manufacturer's instructions. Briefly, 3' and 5' adaptors were sequentially ligated to 1 μg of total RNA prior to reverse transcription and library amplification by PCR. Size selection of the sRNA libraries was performed on 6% Novex TBE PAGE Gels (Thermo Fisher Scientific, cat. EC6265BOX) and purified by ethanol precipitation. Both the library size assessment and library quantification were carried out in a Fragment Analyzer™. Finally, the libraries were pooled and sequenced on an Illumina NextSeq 500 platform (Supplemetary FigureS12).

All samples were analyzed with BrumiR separately with default parameters to identify the candidate miRNAs. We further validated the candidates having a putative precursor with a hairpin structure analysis using the BrumiR2ref tool with the reference genome for *A. thaliana* (GCF_000001735.4_TAIR10.1_genomic.fna). All validated candidate miRNAs were compared to known miRNAs described for *A. thaliana* (437) present in miRBase (v21) (Supplemetary Table S9). We used the current criteria to validate and annotate miRNAs in plants which are based on experimental evidence coming directly from the sequencing libraries, as shown in Axtell 2018 [51]. We conserved the candidates predicted in all the replicates (as is

described in Figure 4), and the putative novel miRNAs were manually curated. Specifically (Supplementary Table S11), we checked the criteria related to precursor length, hairpin structure and miRNA length in at least two sRNA-seq libraries (biological replicates) (Figure S11) [18]. Then a target analysis was performed using the Araport 11 cDNA library with the plant- specific psRNATarget algorithm (based on a best expectation score) (Supplementary Table S12) [53].

AVAILABILITY OF SUPPORTING SOURCE CODE AND REQUIREMENTS

Project name: BrumiR

Project home page: https://github.com/camoragaq/BrumiR

Operating system(s): Unix, Linux and Mac OSX

Programming language: C++, PERL and R

Other requirements: Compilation was tested with g++ using -std=c++11 for Linux and Mac OSX system

RRID: SCR_022727

License: MIT

Any restrictions to use by non-academics: none

AVAILABILITY OF SUPPORTING DATA

Snapshots of our code and other data further supporting this work, are openly available in the *GigaScience* repository, GigaDB [67].

ABBREVIATIONS

NGS: Next Generation Sequencing; CC: Connected Component; GFA: Graphical fragment assembly; CPU: central processing unit; GC: guanine cytosine; CPG: cytosine and guanine separated by only one phosphate group; CWF: sequence complexity by Wootton & Federhen values; CE: sequence Shannon entropy; KM:

kmer mean abundance; PCR: polymerase chain reaction; SRA: Sequence Read Archive; ENA: European Nucleotide Archive.

COMPETING INTEREST

The authors declare that they have no competing interests.

FUNDING

This work was supported by CONICYT BECAS CHILE DOCTORADO 2016/FOLIO 72170320 granted to CM, by a post-doctorate fellowship from the Agence National de Recherche (ANR-GREEN 17_CE20_0031_01) granted to MGF, as well as by Fondo Nacional de Desarrollo Científico y Tecnológico (FONDECYT)-ANID grant 1170926 and 1211130, ANID PCI-Redes Internacionales entre Centros de Investigación grant REDES180097 and ANID—Millennium Science Initiative Program—ICN17_022, ANID/ACT210007 granted to EAV.

AUTHOR CONTRIBUTIONS

CM designed, developed, implemented and benchmarked BrumiR. MFS guided the development of BrumiR. ES conducted the *A. thaliana* experiments. EAV designed and supervised the *A. thaliana* experiments. CM wrote the initial version of the manuscript with inputs from all other authors. MFS and MGF helped to improve the

manuscript. EAV, MFS, MGF and RAG provided crucial biological feedback. All authors provided helpful discussions for the work and reviewed the manuscript.

ACKNOWLEDGEMENTS

This research was performed using the computing facilities of the LBBE/PRABI, the France Génomique e-infrastructure (ANR-10-INBS-09-08) and the supercomputing infrastructure of the NLHPC (ECM-02) a. Special acknowledgments to Dr. Alex Di Genova for all his advice and fruitful discussions.

References

[1] D. P. Bartel, "MicroRNAs: Genomics, Biogenesis, Mechanism, and Function," *Cell*, vol. 116, no. 2, pp. 281–297, Jan. 2004, doi: 10.1016/S0092-8674(04)00045-5.

[2] D. P. Bartel, "MicroRNAs: target recognition and regulatory functions," *Cell*, vol. 136, no. 2, pp. 215–233, Jan. 2009, doi: 10.1016/j.cell.2009.01.002.

[3] J. Greene *et al.*, "Circular RNAs: Biogenesis, Function and Role in Human Diseases," *Front. Mol. Biosci.*, vol. 4, p. 38, 2017, doi: 10.3389/fmolb.2017.00038.

[4] Y. Peng and C. M. Croce, "The role of MicroRNAs in human cancer," *Signal Transduct. Target. Ther.*, vol. 1, p. 15004, Jan. 2016, doi: 10.1038/sigtrans.2015.4.

[5] R. Lin *et al.*, "Comprehensive analysis of microRNA-Seq and target mRNAs of rice sheath blight pathogen provides new insights into pathogenic regulatory mechanisms," *DNA Res.*, vol. 23, no. 5, pp. 415–425, Oct. 2016, doi: 10.1093/dnares/dsw024.

[6] J. Wang, J. Chen, and S. Sen, "MicroRNA as Biomarkers and Diagnostics," *J. Cell. Physiol.*, vol. 231, no. 1, pp. 25–30, 2016, doi: 10.1002/jcp.25056.

[7] M. Lagos-Quintana, R. Rauhut, W. Lendeckel, and T. Tuschl, "Identification of novel genes coding for small expressed RNAs," *Science*, vol. 294, no. 5543, pp. 853–858, Oct. 2001, doi: 10.1126/science.1064921.

[8] N. C. Lau, L. P. Lim, E. G. Weinstein, and D. P. Bartel, "An Abundant Class of Tiny RNAs with Probable Regulatory Roles in Caenorhabditis elegans," *Science*, vol. 294, no. 5543, pp. 858–862, Oct. 2001, doi: 10.1126/science.1065062.

[9] X. Cai, C. H. Hagedorn, and B. R. Cullen, "Human microRNAs are processed from capped, polyadenylated transcripts that can also function as mRNAs," *RNA N. Y. N*, vol. 10, no. 12, pp. 1957–1966, Dec. 2004, doi: 10.1261/rna.7135204.

[10] Y. Lee, K. Jeon, J.-T. Lee, S. Kim, and V. N. Kim, "MicroRNA maturation: stepwise processing and subcellular localization," *EMBO J.*, vol. 21, no. 17, pp. 4663–4670, Sep. 2002, doi: 10.1093/emboj/cdf476.

[11] Y. Lee *et al.*, "MicroRNA genes are transcribed by RNA polymerase II," *EMBO J.*, vol. 23, no. 20, pp. 4051–4060, Oct. 2004, doi: 10.1038/sj.emboj.7600385.

[12] G. M. Borchert, W. Lanier, and B. L. Davidson, "RNA polymerase III transcribes human microRNAs," *Nat. Struct. Mol. Biol.*, vol. 13, no. 12, pp. 1097–1101, Dec. 2006, doi: 10.1038/nsmb1167.

[13] Y. Lee *et al.*, "The nuclear RNase III Drosha initiates microRNA processing," *Nature*, vol. 425, no. 6956, pp. 415–419, Sep. 2003, doi: 10.1038/nature01957.

[14] B. C. Meyers *et al.*, "Criteria for Annotation of Plant MicroRNAs," *Plant Cell*, vol. 20, no. 12, pp. 3186–3190, Dec. 2008, doi: 10.1105/tpc.108.064311.

[15] A. Khvorova, A. Reynolds, and S. D. Jayasena, "Functional siRNAs and miRNAs exhibit strand bias," *Cell*, vol. 115, no. 2, pp. 209–216, Oct. 2003, doi: 10.1016/s0092-8674(03)00801-8.

[16] D. S. Schwarz, G. Hutvágner, T. Du, Z. Xu, N. Aronin, and P. D. Zamore, "Asymmetry in the assembly of the RNAi enzyme complex," *Cell*, vol. 115, no. 2, pp. 199–208, Oct. 2003, doi: 10.1016/s0092-8674(03)00759-1.

[17] D. P. Bartel, "Metazoan MicroRNAs," *Cell*, vol. 173, no. 1, pp. 20–51, Mar. 2018, doi: 10.1016/j.cell.2018.03.006.

[18] M. Bortolomeazzi, E. Gaffo, and S. Bortoluzzi, "A survey of software tools for microRNA discovery and characterization using RNA-seq," *Brief. Bioinform.*, vol. 20, no. 3, pp. 918–930, 21 2019, doi: 10.1093/bib/bbx148.

[19] N. Pinzón *et al.*, “microRNA target prediction programs predict many false positives,” *Genome Res.*, vol. 27, no. 2, pp. 234–245, Feb. 2017, doi: 10.1101/gr.205146.116.
[20] R. D. Morin *et al.*, “Application of massively parallel sequencing to microRNA profiling and discovery in human embryonic stem cells,” *Genome Res.*, vol. 18, no. 4, pp. 610–621, Apr. 2008, doi: 10.1101/gr.7179508.
[21] L. Chen, L. Heikkinen, C. Wang, Y. Yang, H. Sun, and G. Wong, “Trends in the development of miRNA bioinformatics tools,” *Brief. Bioinform.*, vol. 20, no. 5, pp. 1836–1852, 27 2019, doi: 10.1093/bib/bby054.
[22] A. Kozomara and S. Griffiths-Jones, “miRBase: annotating high confidence microRNAs using deep sequencing data,” *Nucleic Acids Res.*, vol. 42, no. Database issue, pp. D68–D73, Jan. 2014, doi: 10.1093/nar/gkt1181.
[23] D. M. Vitsios *et al.*, “Mirnovo: genome-free prediction of microRNAs from small RNA sequencing data and single-cells using decision forests,” *Nucleic Acids Res.*, vol. 45, no. 21, pp. e177–e177, Dec. 2017, doi: 10.1093/nar/gkx836.
[24] B. Langmead, C. Trapnell, M. Pop, and S. L. Salzberg, “Ultrafast and memory-efficient alignment of short DNA sequences to the human genome,” *Genome Biol.*, vol. 10, no. 3, p. R25, Mar. 2009, doi: 10.1186/gb-2009-10-3-r25.
[25] H. Li and R. Durbin, “Fast and accurate short read alignment with Burrows–Wheeler transform,” *Bioinformatics*, vol. 25, no. 14, pp. 1754–1760, Jul. 2009, doi: 10.1093/bioinformatics/btp324.
[26] M. Ziemann, A. Kaspi, and A. El-Osta, “Evaluation of microRNA alignment techniques,” *RNA*, vol. 22, no. 8, pp. 1120–1138, Aug. 2016, doi: 10.1261/rna.055509.115.
[27] Y. Li, Z. Zhang, F. Liu, W. Vongsangnak, Q. Jing, and B. Shen, “Performance comparison and evaluation of software tools for microRNA deep-sequencing data analysis,” *Nucleic Acids Res.*, vol. 40, no. 10, pp. 4298–4305, May 2012, doi: 10.1093/nar/gks043.
[28] “A reference standard for genome biology,” *Nat. Biotechnol.*, vol. 36, no. 12, p. 1121, Dec. 2018, doi: 10.1038/nbt.4318.
[29] M. R. Friedländer *et al.*, “Discovering microRNAs from deep sequencing data using miRDeep,” *Nat. Biotechnol.*, vol. 26, no. 4, pp. 407–415, Apr. 2008, doi: 10.1038/nbt1394.
[30] M. R. Friedländer, S. D. Mackowiak, N. Li, W. Chen, and N. Rajewsky, “miRDeep2 accurately identifies known and hundreds of novel microRNA genes in seven animal clades,” *Nucleic Acids Res.*, vol. 40, no. 1, pp. 37–52, Jan. 2012, doi: 10.1093/nar/gkr688.
[31] J. Lei and Y. Sun, “miR-PREFeR: an accurate, fast and easy-to-use plant miRNA prediction tool using small RNA-Seq data,” *Bioinforma. Oxf. Engl.*, vol. 30, no. 19, pp. 2837–2839, Oct. 2014, doi: 10.1093/bioinformatics/btu380.
[32] A. Jha and R. Shankar, “miReader: Discovering Novel miRNAs in Species without Sequenced Genome,” *PLOS ONE*, vol. 8, no. 6, p. e66857, Jun. 2013, doi: 10.1371/journal.pone.0066857.
[33] D. Mapleson, S. Moxon, T. Dalmay, and V. Moulton, “MirPlex: a tool for identifying miRNAs in high-throughput sRNA datasets without a genome,” *J. Exp. Zoolog. B Mol. Dev. Evol.*, vol. 320, no. 1, pp. 47–56, Jan. 2013, doi: 10.1002/jez.b.22483.

[34] P. E. C. Compeau, P. A. Pevzner, and G. Tesler, “Why are de Bruijn graphs useful for genome assembly?,” *Nat. Biotechnol.*, vol. 29, no. 11, pp. 987–991, Nov. 2011, doi: 10.1038/nbt.2023.

[35] R. Chikhi, A. Limasset, and P. Medvedev, “Compacting de Bruijn graphs from sequencing data quickly and in low memory,” *Bioinformatics*, vol. 32, no. 12, pp. i201–i208, Jun. 2016, doi: 10.1093/bioinformatics/btw279.

[36] I. Kalvari *et al.*, “Rfam 13.0: shifting to a genome-centric resource for non-coding RNA families,” *Nucleic Acids Res.*, vol. 46, no. D1, pp. D335–D342, Jan. 2018, doi: 10.1093/nar/gkx1038.

[37] R. R. Wick, M. B. Schultz, J. Zobel, and K. E. Holt, “Bandage: interactive visualization of de novo genome assemblies,” *Bioinformatics*, vol. 31, no. 20, pp. 3350–3352, Oct. 2015, doi: 10.1093/bioinformatics/btv383.

[38] I. Kalvari *et al.*, “Non-Coding RNA Analysis Using the Rfam Database,” *Curr. Protoc. Bioinforma.*, vol. 62, no. 1, p. e51, 2018, doi: 10.1002/cpbi.51.

[39] S. Chen, Y. Zhou, Y. Chen, and J. Gu, “fastp: an ultra-fast all-in-one FASTQ preprocessor,” *Bioinformatics*, vol. 34, no. 17, pp. i884–i890, Sep. 2018, doi: 10.1093/bioinformatics/bty560.

[40] B. Fromm *et al.*, “MirGeneDB 2.0: the metazoan microRNA complement,” *Nucleic Acids Res.*, vol. 48, no. D1, pp. D132–D141, Jan. 2020, doi: 10.1093/nar/gkz885.

[41] M. G. Grabherr *et al.*, “Trinity: reconstructing a full-length transcriptome without a genome from RNA-Seq data,” *Nat. Biotechnol.*, vol. 29, no. 7, pp. 644–652, May 2011, doi: 10.1038/nbt.1883.

[42] D. R. Zerbino and E. Birney, “Velvet: Algorithms for de novo short read assembly using de Bruijn graphs,” *Genome Res.*, vol. 18, no. 5, pp. 821–829, May 2008, doi: 10.1101/gr.074492.107.

[43] C. Roden *et al.*, “Novel determinants of mammalian primary microRNA processing revealed by systematic evaluation of hairpin-containing transcripts and human genetic variation,” *Genome Res.*, vol. 27, no. 3, pp. 374–384, Mar. 2017, doi: 10.1101/gr.208900.116.

[44] M. Pal, “Random forest classifier for remote sensing classification,” *Int. J. Remote Sens.*, vol. 26, no. 1, pp. 217–222, Jan. 2005, doi: 10.1080/01431160412331269698.

[45] P. Romero, Z. Obradovic, X. Li, E. C. Garner, C. J. Brown, and A. K. Dunker, “Sequence complexity of disordered protein,” *Proteins Struct. Funct. Bioinforma.*, vol. 42, no. 1, pp. 38–48, 2001, doi: 10.1002/1097-0134(20010101)42:1<38::AID-PROT50>3.0.CO;2-3.

[46] B. J. Reinhart, E. G. Weinstein, M. W. Rhoades, B. Bartel, and D. P. Bartel, “MicroRNAs in plants,” *Genes Dev.*, vol. 16, no. 13, pp. 1616–1626, Jul. 2002, doi: 10.1101/gad.1004402.

[47] N. Fahlgren *et al.*, “High-Throughput Sequencing of Arabidopsis microRNAs: Evidence for Frequent Birth and Death of MIRNA Genes,” *PLOS ONE*, vol. 2, no. 2, p. e219, Feb. 2007, doi: 10.1371/journal.pone.0000219.

[48] N. W. Breakfield *et al.*, “High-resolution experimental and computational profiling of tissue-specific known and novel miRNAs in Arabidopsis,” *Genome Res.*, Sep. 2011, doi: 10.1101/gr.123547.111.

[49] L.-C. Hsieh *et al.*, "Uncovering small RNA-mediated responses to phosphate deficiency in Arabidopsis by deep sequencing," *Plant Physiol.*, vol. 151, no. 4, pp. 2120–2132, Dec. 2009, doi: 10.1104/pp.109.147280.

[50] D. Moldovan, A. Spriggs, J. Yang, B. J. Pogson, E. S. Dennis, and I. W. Wilson, "Hypoxia-responsive microRNAs and trans-acting small interfering RNAs in Arabidopsis," *J. Exp. Bot.*, vol. 61, no. 1, pp. 165–177, Jan. 2010, doi: 10.1093/jxb/erp296.

[51] M. J. Axtell and B. C. Meyers, "Revisiting Criteria for Plant MicroRNA Annotation in the Era of Big Data," *Plant Cell*, vol. 30, no. 2, pp. 272–284, Feb. 2018, doi: 10.1105/tpc.17.00851.

[52] S. B. Satbhai, D. Ristova, and W. Busch, "Underground tuning: quantitative regulation of root growth," *J. Exp. Bot.*, vol. 66, no. 4, pp. 1099–1112, Feb. 2015, doi: 10.1093/jxb/eru529.

[53] X. Dai, Z. Zhuang, and P. X. Zhao, "psRNATarget: a plant small RNA target analysis server (2017 release)," *Nucleic Acids Res.*, vol. 46, no. W1, pp. W49–W54, Jul. 2018, doi: 10.1093/nar/gky316.

[54] N. Vukašinović *et al.*, "Microtubule-dependent targeting of the exocyst complex is necessary for xylem development in Arabidopsis," *New Phytol.*, vol. 213, no. 3, pp. 1052–1067, Feb. 2017, doi: 10.1111/nph.14267.

[55] P. Dvořák *et al.*, "FSD1: developmentally-regulated plastidial, nuclear and cytoplasmic enzyme with anti-oxidative and osmoprotective role," *Plant Cell Environ.*, Apr. 2020, doi: 10.1111/pce.13773.

[56] J.-M. Couzigou and J.-P. Combier, "Plant microRNAs: key regulators of root architecture and biotic interactions," *New Phytol.*, vol. 212, no. 1, pp. 22–35, 2016, doi: 10.1111/nph.14058.

[57] D. A. Durai and M. H. Schulz, "Informed kmer selection for de novo transcriptome assembly," *Bioinformatics*, vol. 32, no. 11, pp. 1670–1677, Jun. 2016, doi: 10.1093/bioinformatics/btw217.

[58] R. Chikhi and G. Rizk, "Space-efficient and exact de Bruijn graph representation based on a Bloom filter," *Algorithms Mol. Biol.*, vol. 8, no. 1, p. 22, Sep. 2013, doi: 10.1186/1748-7188-8-22.

[59] S. Deorowicz, A. Debudaj-Grabysz, and S. Grabowski, "Disk-based k-mer counting on a PC," *BMC Bioinformatics*, vol. 14, no. 1, p. 160, May 2013, doi: 10.1186/1471-2105-14-160.

[60] H. R. Lewis and C. H. Papadimitriou, "Symmetric space-bounded computation," *Theor. Comput. Sci.*, vol. 19, no. 2, pp. 161–187, Aug. 1982, doi: 10.1016/0304-3975(82)90058-5.

[61] M. Šošić and M. Šikić, "Edlib: a C/C ++ library for fast, exact sequence alignment using edit distance," *Bioinformatics*, vol. 33, no. 9, pp. 1394–1395, May 2017, doi: 10.1093/bioinformatics/btw753.

[62] M. Lambert, A. Benmoussa, and P. Provost, "Small Non-Coding RNAs Derived from Eukaryotic Ribosomal RNA," *Non-Coding RNA*, vol. 5, no. 1, p. 16, Mar. 2019, doi: 10.3390/ncrna5010016.

[63] M. Kokot, M. Dlugosz, and S. Deorowicz, "KMC 3: counting and manipulating k-mer statistics," *Bioinforma. Oxf. Engl.*, vol. 33, no. 17, pp. 2759–2761, Sep. 2017, doi: 10.1093/bioinformatics/btx304.

[64] S. Higashi, C. Fournier, C. Gautier, C. Gaspin, and M.-F. Sagot, "Mirinho: An efficient and general plant and animal pre-miRNA predictor for genomic and deep

sequencing data," *BMC Bioinformatics*, vol. 16, no. 1, p. 179, May 2015, doi: 10.1186/s12859-015-0594-0.

[65] R. Lorenz *et al.*, "ViennaRNA Package 2.0," *Algorithms Mol. Biol.*, vol. 6, no. 1, p. 26, Nov. 2011, doi: 10.1186/1748-7188-6-26.

[66] P. Danaee, M. Rouches, M. Wiley, D. Deng, L. Huang, and D. Hendrix, "bpRNA: large-scale automated annotation and analysis of RNA secondary structure," *Nucleic Acids Res.*, vol. 46, no. 11, pp. 5381–5394, Jun. 2018, doi: 10.1093/nar/gky285.

[67] Moraga C; Sanchez E; Ferrarini MG; Gutierrez RA; Vidal EA; Sagot M: Supporting data for "BrumiR: A toolkit for de novo discovery of microRNAs from sRNA-seq data." GigaScience Database. 2022. http://doi.org/10.5524/102250.

Click here to access/download
Supplementary Material
brumir-sup-rev.pdf

GIGA-D-20-00262R1
BrumiR: A toolkit for de novo discovery of microRNAs from sRNA-seq data.
Carol Moraga; Evelyn Sanchez; Mariana Galvao Ferrarini; Rodrigo A Gutierrez; Elena A Vidal; Marie-France Sagot GigaScience

Dear Dr. Moraga,

Your manuscript "BrumiR: A toolkit for de novo discovery of microRNAs from sRNA-seq data." (GIGA-D-20-00262R1) has been assessed by our reviewers. Although it is of interest, we are unable to consider it for publication in its current form. The reviewers and Editorial Board member have raised a number of points which we believe would improve the manuscript and may allow a revised version to be published in GigaScience.

Reviewer #1 is happy with the revisions made. However, Reviewer #2 was still not happy with the revisions and decided not to submit a formal review; however, they have provided some detailed and useful feedback. We then had to seek advice from an Editorial board member who has considered the feedback from Reviewer #2 and they have s suggested the following major revisions, which we feel is fair, and must be made in order for us to consider this manuscript for GigaScience.

Overall, there is a concern with regards to the robustness of the tool, and this must be proven.

19th July 2022

Dear Dr. Nogoy,

Thank you very much for considering our manuscript for publication in GigaScience. We would like to thank the referees, the Editorial Board member, and you for the careful assessment of our manuscript. We have attempted to address all points raised by the referees putting special emphasis on the precision of our method and hope that the responses are satisfactory. With these revisions, we believe that our manuscript has been substantially improved and hope that it is now suitable for publication in GiGaScience.

Please find below our point-by-point replies to the reviewers' comments. All changes in the main manuscript and the supplement have been marked in blue font. We have reformatted the manuscript according to the guidelines of GiGaScience.

Yours sincerely,

Carol Moraga

1) Both reviewer 1 and myself noted the low precision of BrumiR on many datasets. The authors have improved precision by using a seed size of 14, but the precision is still down to around 0.35 for the C. elegans datasets using BrumiR and down to ~0.5 when using BrumiR2Reference (Figure 3B). Further, BrumiR still reports ~1500 miRNA candidates for one mouse dataset (Figure 3A), most of which are likely to be false positives given that mouse miRNA annotation is by now fairly saturated. The authors provide compelling evidence that these novel candidates are not piRNAs, but this does not fully clarify the matter, since it is still not clear what they are. The concern raised by reviewer 1 and myself thus remains - what is the use for software that generates so many false positives? This should also be seen in the light that miRBase already contains many false positives that can confound e.g. evolutionary analyses. This topic is discussed here:
https://academic.oup.com/nar/article/48/D1/D132/5584683

Our Editorial Board member suggests you must clarify the question "Whether the de novo miRNAs really exist?"

We agree that the BrumiR precision might seem low in some datasets and that the results presented in the mouse dataset seem problematic. To improve the BrumiR precision we have explored new methods and algorithms. After thoroughly examining the BrumiR code, we attempted to resolve the precision issue as a classification problem and implemented/designed a supervised machine learning method (random forest) to classify BrumiR candidates using an additional and new BrumiR tool (brumirRF.pl). The random forest model is composed of 19 features, of which 16 are inferred directly from 15-mer sequences of each BrumiR candidate and three derived from nucleotide composition observed on reference mature miRNA sequences (miRGeneDB and miRbase). The 16 derived features are GC content(gc), GC skew content (gcs), CpG content (cpg), sequence complexity by Wootton & Federhen (cwf), sequence Shannon entropy (ce), sequence complexity of Markov model values (cm1,cm2,cm3), sequence complexity by Trifonov values (ct3,ct4,ct5,ct6) and sequence complexity linguistic values (cl3,cl4,cl5,cl6). The nucleotide composition are 6-mer, 7-mer, and 8-mer observed frequency of mature miRNA sequences on reference miRNA databases (MirGeneDB and miRBase). The features are computed on a 15-mer basis to classify any length of miRNA candidates (18-22 base pairs). We built one model for animals using

the curated entries from MirGeneDB as suggested by Editorial board member. A total of 35570 15-mer were derived from the MiRGeneDB, and all the 19 features were computed for each. A matching amount of 15-mer random sequences were generated, and all the 19 features were computed for each. The whole training and evaluation dataset comprised 71140 15-mers of the two classes (random and mature miRNAs sequences). The training and evaluation of the random forest were performed using 75% and 25%, respectively. The benchmark results show that the random forest classifier achieves an accuracy of 90%, a precision of 87%, and a recall of 94% for discriminating miRNAs 15-mers from random 15-mers. The most top-5 informative features were 8-mer, 7-mer, 6-mer, CpG content, GC content, and the complexity of markov models. Another model using miRBase entries and the same features achieves an accuracy of 90%, a precision of 87%, and a recall of 93% for discriminating plant miRNAs 15-mers from random 15-mer sequences. The building and evaluation of both random forest models are available on the BrumiR GitHub repository (here: https://github.com/camoragaq/BrumiR/tree/master/brumir-rf). Our major aim in implementing this classifier was to keep the BrumiR recall and increase the precision, therefore, reducing the false positive rate. We evaluated the performance of this classifier on the mouse dataset, prior to incrementing the minimal candidate coverage to 50X, which is now the default for reporting a miRNA candidate (before was 20X). The number of candidates for this mouse sample is now 934 and after applying the random forest it decreases to 490 candidates, which results in a recall of 87%, precision of 60%, and F-Score of 70%. The previous number represents an increase of 3,5 fold in precision (previously was 17%). We do want to remark that the only information about know miRNA sequences is the composition of 6-mer, 7-mer, and 8-mer. Therefore, BrumiR uses little information of know miRNAs sequences. In the current random forest implementation, we do classify BrumiR candidates as potential miRNAs sequences when candidates have a random forest probability greater than 0.8. Similar results were observed on the other evaluated datasets (Figure 4), therefore the random forest classifier allowed us to increase the BrumiR precision without affecting the BrumiR recall. The new BrumiR tool is described in the main text in the section "**Using a supervised Machine Learning approach to refine the BrumiR-core predictions**".

2) Our Editorial board member also requests pairwise performance data comparison of BrumiR and a manually curated and open-source miRNA gene database, MirGeneDB, which is based on consistent annotation and nomenclature criteria. The database contains high-quality annotations of 10 899 bonafide and consistently named miRNAs constituting 1275 miRNA families from 45 species, representing every major metazoan group, including many well-established and emerging invertebrates and vertebrate model organisms.

We have followed the reviewer recommendations and we computed the performance of BrumiR and those of other methods using the MirGeneDB database on 5 animal datasets (with two replicates) which are the ones with entries in the MirGeneDB. The new benchmark shows that BrumiR achieves the highest F-Score (9/10 datasets) with an average F-Score of 0.53 while their competitors achieve 0.3 and 0.36 for mirDeep2 and mirnovo, respectively. Regarding the precision, we have been aware of the low precision of all the methods, over the revisions we have improved the BrumiR precision (first report) and now we improved substantially by the use of a random forest classifier (see the answer to question 1) from X to Y. Final values of benchmark metrics are available on supplementary table X and discussions about BrumiR precision are provided on the new manuscript section "**Using a supervised Machine Learning approach to refine the BrumiR-core predictions**". We do thank the reviewer for pointing out the MirGeneDB database which was key for inspiring us to further increase the precision of our method by means of a machine learning classifier.

3) In addition, a fair comparison to other de novo tools would be nice to further support the work, and a small-scale validation would be a big plus, but may not be necessary if the miRNA species from MirGeneDB could be predicted accurately.

We evaluated all methods' performance using the MirGeneDB and miRBase databases. We observe that BrumiR outperforms current approaches and is the only tool that generates reliable results in the absence of a reference genome. We want to remark that this version is the first one of our method and that further improvements are still possible (regarding precision and recall). Regarding experiments, we have included extensive Arabidopsis data showing the utility of our method by discovering high-quality novel miRNAs even in the reference plant model Arabidopsis thaliana. Unfortunately, further experimental validation of the BrumiR candidates has been out of our reach, and we do plan to perform it shortly after securing funding for further development of BrumiR. Still, as one of the reviewers pointed out, this is the first tool that implements a Bruijn Graph approach, and additional improvement from the community or us might be possible after BrumiR publication.

Please also take a moment to check our website at https://www.editorialmanager.com/giga/ for any additional comments that were saved as attachments.

In addition, please register any new software application in the bio.tools and SciCrunch.org databases to receive RRID (Research Resource Identification Initiative ID) and biotoolsID identifiers, and include these in your manuscript. This will facilitate tracking, reproducibility and re-use of your tool.

If you are able to fully address these points, we would encourage you to submit a revised manuscript to GigaScience. Once you have made the necessary corrections, please submit online at:

https://www.editorialmanager.com/giga/

If you have forgotten your username or password please use the "Send Login Details" link to get your login information. For security reasons, your password will be reset.

Please include a point-by-point within the 'Response to Reviewers' box in the submission system. Please ensure you describe additional experiments that were carried out and include a detailed rebuttal of any criticisms or requested revisions that you disagreed with. Please also ensure that your revised manuscript conforms to the journal style, which can be found in the Instructions for Authors on the journal homepage. If the data and code has been modified in the revision process please be sure to update the public versions of this too.

The due date for submitting the revised version of your article is 09 Mar 2022. I look forward to receiving your revised manuscript soon.
Best wishes,

Nicole Nogoy, Ph.D GigaScience

Reviewer reports:

Reviewer #1: The authors have significantly improved their manuscript, especially by fixing all my previous questions regarding the performance of BrumiR. Although my question about wet-lab validation has not been fully settled, I agree with the authors that it can be considered as out of the scope of this study.
The revised BrumiR outperformed some of the commonly used tools in the miRNA prediction field. The overall performance, in terms of F1-score, is on the top among the comparisons, despite the potential to further improve its precision.
In summary, I believe BrumiR is a solid tool for miRNA prediction, and its idea of using de Bruijn graph and reference-free approaches could inspire further application development in the genetic field. This manuscript is ready to be accepted by Giga Science.

Again, we would like to thank all referees, the Editorial Board member, and you for the crucial suggestions that inspired us to extend the range of applications for BrumiR (BrumiR random forest), which led to improved new benchmark results that make the BrumiR predictions more robust.

Sincerely,
Carol Moraga on behalf of all authors
