# Supplementary material for: BrumiR: A toolkit for de novo discovery of microRNAs from sRNA-seq data
Source: Gigascience. 2022 Oct 25;11:giac093. doi: 10.1093/gigascience/giac093 (PMC9596168; doi:10.1093/gigascience/giac093)
Supplement: giac093_Supplemental_File [file giac093_supplemental_file.pdf]

Supplementary Material: ”BrumiR: A toolkit for *de novo* discovery of microRNAs from sRNA-seq data.”

Carol Moraga[1,2], Evelyn Sanchez[3,4], Mariana Galvão Ferrarini[5], Rodrigo A. Gutierrez[4,6,7], Elena A. Vidal[3,4,8] and Marie-France Sagot[1,2]

[1]*Inria Grenoble Rhoñe-Alpes, 655, Avenue de l'Europe, 38334 Montbonnot, France.*

[2]*Université de Lyon, Université Lyon 1, CNRS, Laboratoire de Biométrie et Biologie Evolutive UMR 5558, F-69622 Villeurbanne, France.*

[3]*Centro de Genómica y Bioinformática, Facultad de Ciencias, Universidad Mayor, Chile.*

[4]*Millennium Institute for Integrative Biology iBio, Chile.*

[5]*Université de Lyon, INSA-Lyon, INRA, BF2i, UMR0203, F-69621 Villeurbanne, France.*

[6]*Departamento de Genética Molecular y Microbiología, Facultad de Ciencias Biológicas, Pontificia Universidad Católica de Chile.*

[7]*FONDAP Center for Genome Regulation.*

[8]*Escuela de Biotecnología, Facultad de Ciencias, Universidad Mayor.*

Correspondence should be addressed to Carol Moraga (email: camoragaq@gmail.com) & Marie France Sagot (email: marie-france.sagot@inria.fr)

List of Figures

Figure S1 Length distribution of mature sequences in miRBase. 17
Figure S2 Determination of the optimal BrumiR k-mer size 18
Figure S3 Benchmark metric comparison across the whole dataset between 14-mers and 18-mers 19
Figure S4 Kmer spectrum of sRNA-seq data. 20
Figure S5 BrumiR depth coverage distribution. 21
Figure S6 BrumiR classifies low abundance non-linear topologies as sequencing errors. 21
Figure S7 Re-assembling unipaths within each CC. 22
Figure S8 Workflow of the BrumiR2Reference tool. 23
Figure S9 Structure properties of miRBase precursor sequences. 24
Figure S10 F-score distribution of reduced benchmark including 5 miRNA discovery tools. 24
Figure S11 Novel miRNAs candidates discovered by BrumiR using *Arabidopsis thaliana* root samples. 25
Figure S12 Visualization with Bandage. 26
Figure S13 Experimental procedure of the *Arabidopsis thaliana* roots sampling and the sRNA-seq libraries construction. 27

List of Tables

Table S1 5
Table S2 6
Table S3 7
Table S4 8
Table S5 9
Table S6 10
Table S7 11
Table S8 12
Table S9 13
Table S10 14
Table S11 14
Table S12 15
Table S13 16

BrumiR commands

BrumiR commands for real and simulated benchmark.

```
#trimming raw sequences using fastp
fastp --adapter_fasta ../adapters.fa -i <prefix>.fastq.gz -o <prefix>.trim.fastq.gz

#running miRDeep2
mapper.pl <prefix>.fa -c -p genome-index -m -q -s <prefix>.reads_collapsed.fa -t <prefix>.reads_collapsed_vs_genome.arf -v -o 2
miRDeep2.pl <prefix>.reads_collapsed.fa genome.fna <prefix>.reads_collapsed_vs_genome.arf none none none 2><prefix>.report.log

#running miR-PREFeR
python process-reads-fasta.py samplelist.txt <prefix>.fa <prefix>.fa
python bowtie-align-reads.py -p 2 -k 20 -f -r genome.fna <prefix>.fa.processed
python miR_PREFeR.py -L -k pipeline config.file

#running BrumiR
perl brumir.pl -a <prefix>.trim.fastq.gz -p <prefix> -T 10 -R 2 -d 50 > <prefix>.log

#running BrumiR2reference
#for animal species
perl brumir2reference.pl -a <prefix>.candidate_miRNA.fasta -b genome.fna -t 4 -p <prefix>

#for plant species
perl brumir2reference.pl -a <prefix>.candidate_miRNA.fasta -b genome.fna -t 4 -p <prefix> -x 1
```

Table S1: Different k-mer seed length to evaluate the performance of BrumiR.

	kmer size	Tool	candidates	tp	fp	match	fn	accuracy	recall	F-score
		brumir	1118	733	385	12435	2938	0,6556	0,8089	0,7242
	k14	mirdeep2	579	457	122	5931	9442	0,7893	0,3858	0,5183
		mirnovo	789	341	448	4363	11010	0,4322	0,2838	0,3426
		brumir	1250	810	440	10974	3080	0,6480	0,7808	0,7082
	k16	mirdeep2	579	457	122	5931	8123	0,7893	0,4220	0,5500
		mirnovo	789	341	448	4363	9691	0,4322	0,3104	0,3613
		brumir	1181	686	495	10041	3121	0,5809	0,7629	0,6595
ERR2403205 Human	k18	mirdeep2	579	457	122	5931	7231	0,7893	0,4506	0,5737
		mirnovo	789	341	448	4363	8799	0,4322	0,3315	0,3752
		brumir	941	529	412	9435	3337	0,5622	0,7387	0,6385
	k20	mirdeep2	579	457	122	5931	6841	0,7893	0,4644	0,5847
		mirnovo	789	341	448	4363	8409	0,4322	0,3416	0,3816
		brumir	719	400	319	8890	3592	0,5563	0,7122	0,6247
	k22	mirdeep2	579	457	122	5931	6551	0,7893	0,4752	0,5932
		mirnovo	789	341	448	4363	8119	0,4322	0,3495	0,3865
		brumir	1291	588	703	7949	5098	0,4555	0,6093	0,5213
	k14	mirprefer	311	110	201	3296	9751	0,3537	0,2526	0,2947
		mirnovo	120	54	66	2183	10864	0,4500	0,1673	0,2439
		brumir	1382	593	789	4384	5206	0,4291	0,4571	0,4427
	k16	mirprefer	311	110	201	3296	6294	0,3537	0,3437	0,3486
		mirnovo	120	54	66	2183	7407	0,4500	0,2276	0,3023
		brumir	1238	437	801	3386	5175	0,3530	0,3955	0,3730
SRR8992309 Arabidopsis	k18	mirprefer	311	110	201	3296	5265	0,3537	0,3850	0,3687
		mirnovo	120	54	66	2183	6378	0,4500	0,2550	0,3255
		brumir	993	218	775	2373	5755	0,2195	0,2920	0,2506
	k20	mirprefer	311	110	201	3296	4832	0,3537	0,4055	0,3778
		mirnovo	120	54	66	2183	5945	0,4500	0,2686	0,3364
		brumir	644	122	522	1467	6370	0,1894	0,1872	0,1883
	k22	mirprefer	311	110	201	3296	4541	0,3537	0,4206	0,3842
		mirnovo	120	54	66	2183	5654	0,4500	0,2786	0,3441

Table S2: Simulated sRNA-seq used to evaluate the performance of BrumiR.

							BrumiR		miRDeep2/miR-PREFeR		BrumiR			miRDeep2/miR-PREFeR		
		miRBase input	processed reads	map to the reference genome	%mapped (reference genome approach)	unipaths (BrumiR approach)	candidates	match in miRBase	candidates	match miRBase	precision	recall	F-score	precision	recall	F-score
Homo sapiens	f1	897	27,21	23,92	87,90	114712	1756	869	1055	586	0,495	0,969	0,655	0,555	0,653	0,600
	f2		27,21	23,37	85,89	114960	1685	870	1172	589	0,516	0,969	0,674	0,503	0,653	0,568
Mus musculus	f1	816	25,28	22,63	89,52	98642	1582	787	683	507	0,497	0,964	0,656	0,742	0,621	0,676
	f2		25,28	22,11	87,47	99287	1605	787	739	510	0,490	0,964	0,650	0,690	0,625	0,656
Drosophila melagonaster	f1	284	8,84	8,04	90,98	35281	589	277	134	122	0,470	0,975	0,635	0,910	0,430	0,584
	f2		8,84	7,81	88,32	35542	582	277	134	126	0,476	0,975	0,640	0,875	0,444	0,589
Danio rerio	f1	274	8,27	6,93	83,77	34370	475	268	332	167	0,564	0,978	0,716	0,503	0,609	0,551
	f2		8,27	6,72	81,30	34102	485	268	332	166	0,553	0,978	0,706	0,500	0,606	0,548
Caenorhabditis elegans	f1	223	6,49	5,99	92,32	24806	449	220	208	124	0,490	0,987	0,655	0,596	0,556	0,575
	f2		6,49	5,81	89,50	24818	437	218	214	124	0,499	0,978	0,661	0,579	0,556	0,568
Zea mays	f1	325	10,03	7,32	73,04	36385	290	285	633	236	0,983	0,877	0,927	0,231	0,449	0,305
	f2		10,03	7,12	70,96	36324	298	215	665	174	0,990	0,908	0,947	0,260	0,532	0,349
Physcomitrella	f1	298	9,22	7,40	80,32	32789	394	292	383	240	0,741	0,980	0,844	0,627	0,805	0,705
	f2		9,22	7,16	77,67	32944	403	292	415	240	0,725	0,980	0,833	0,578	0,805	0,673
Oryza sativa	f1	775	23,65	16,74	70,77	91445	1157	759	1423	503	0,656	0,979	0,786	0,353	0,649	0,458
	f2		23,65	16,24	68,66	92322	1211	747	1447	492	0,617	0,964	0,752	0,340	0,635	0,443
Solanum lycopersicum	f1	147	4,11	3,14	76,34	15753	242	142	1076	113	0,587	0,966	0,730	0,105	0,769	0,185
	f2		4,11	3,07	74,78	15791	239	142	1040	108	0,594	0,966	0,736	0,104	0,735	0,182
Arabidopsis thaliana	f1	437	13,16	10,70	81,27	49016	705	427	302	278	0,606	0,977	0,748	0,921	0,636	0,752
	f2		13,16	10,38	78,87	49033	714	423	319	260	0,592	0,968	0,735	0,815	0,595	0,688

Table S3: Total elapsed time per tool (seconds) on synthetic datasets. The total elapsed time reported include only the core step of each algorithm.

	species	dataset	BrumiR	miRDeep2/miR-PREFeR
animal	Homo sapiens	f1	263	6394
		f2	368	9661
	Mus musculus	f1	235	5101
		f2	336	8169
	Drosophila melagonaster	f1	87	922
		f2	104	1182
	Danio rerio	f1	86	1679
		f2	95	2067
	Caenorhabditis elegans	f1	79	917
		f2	90	1044
plant	Zea mays	f1	81	177
		f2	87	173
	Physcomitrella	f1	74	196
		f2	95	222
	Oryza sativa	f1	192	1661
		f2	243	1342
	Solanum lycopersicum	f1	46	1204
		f2	53	1193
	Arabidopsis thaliana	f1	125	363
		f2	160	362

Table S4: Real sRNA-seq data used to evaluate the performance of BrumiR.

		Homo sapiens		Mus musculus		Drosophila melagonaster		Danio rerio		Caenorhabditis elegans		Zea mays		Physcomitrella		Oryza sativa		Solanum lycopersicum		Arabidopsis thaliana	
		ERR2403205	ERR2403208	SRR1734814	SRR1734817	ERR3276595	ERR3276596	SRR2127900	SRR2127901	ERR562743	ERR562747	SRR7801904	SRR7801905	SRR1842130	SRR1842134	SRR6456419	SRR6456420	SRR6872534	SRR6872539	SRR8992309	SRR8992311
	reads	23787456	23195170	3492694	39444355	15420047	11770115	5964199	5813773	7109534	18014441	14963874	15958874	8258652	7381000	14099053	13193443	21093873	18211752	36783111	32827606
	processed reads	21837154	20248581	3483185	39349213	15300462	11690069	5766858	5526102	6666247	10723675	11698286	11770953	7965525	7144623	13263168	11582302	19827894	17343516	36070219	32512424
	map to the reference genome	15397405	15468470	1686235	18956030	7930708	6611362	4025133	3580407	5793813	8859905	2732893	3447092	4093364	3194414	8949699	6560968	11143699	10163965	33816174	29856834
	%mapped (refrence genome approach)	70,51	76,39	48,41	48,17	51,83	56,56	69,80	64,79	86,91	82,62	23,36	29,29	51,39	44,71	67,48	56,65	56,20	58,60	93,75	91,83
	unipaths (BrumiR approach)	122147	133437	92975	1145535	119292	82120	65788	73113	109844	67813	399293	398490	88192	79899	590548	342118	1376168	1304218	213899	226501
	candidates	602	632	249	934	318	266	304	330	391	514	497	608	565	478	588	466	1418	1284	480	557
BrumiR	candidates with match (true positives)	265	282	124	347	105	100	191	188	160	177	181	237	252	215	246	201	413	418	170	200
	match in MirGeneDB/ miRBase	6352	6391	5831	8304	1929	1912	8060	7998	2284	2211	3437	3328	2138	2247	3543	4424	4343	3720	1659	2711
	candidates	374	391	160	567	140	121	201	214	169	208	376	435	386	306	350	247	778	731	182	229
BrumiR2ref	candidates with match (true positives)	188	193	89	286	64	67	129	132	104	109	152	190	178	145	171	123	277	280	80	112
	match in MirGeneDB/ miRBase	4987	4833	4660	6880	1464	1459	6545	6537	1029	1299	3314	3126	1748	1742	2486	2207	3410	3059	1256	2416
	candidates	579	711	278	383	120	115	537	579	171	196	4401	3158	4769	3798	5569	3774	3257	3001	311	441
miRDeep2/miR-PREFeR	candidates with match (true positives)	191	220	148	192	38	97	209	221	47	37	649	493	1301	1088	2361	1792	1317	1263	110	148
	match in miRBase	2329	2728	1962	2448	5104	1131	9207	4096	376	242	3377	3328	2064	2025	3207	3482	3650	3603	3296	3376
	candidates	789	1017	358	259	5476	4181	5476	4181	955	2642	91	82	90	78	127	147	230	210	120	134
mirnovo	candidates with match (true positives)	127	348	212	137	1000	791	1679	252	326	168	38	27	56	27	70	76	110	108	54	60
	match in miRBase	2866	7944	7975	2868	4476	3390	3797	9397	3302	1348	2109	875	1475	56	2308	2034	2916	3795	2183	2373
	precision	0,4385	0,4462	0,4980	0,3715	0,3302	0,3759	0,6283	0,5697	0,4092	0,3444	0,3642	0,3898	0,4460	0,4498	0,4184	0,4313	0,2913	0,3255	0,3542	0,3591
BrumiR	recall	0,8601	0,7370	0,6685	0,8805	0,3139	0,3346	0,8305	0,8191	0,6757	0,7465	0,8567	0,7873	0,7160	0,7267	0,7596	0,7547	0,8007	0,7027	0,4209	0,6450
	F-score	0,5809	0,5559	0,5708	0,5226	0,3218	0,3540	0,7154	0,6720	0,5097	0,4713	0,5111	0,5214	0,5496	0,5557	0,5396	0,5489	0,4271	0,4449	0,3846	0,4613
	precision	0,5027	0,4936	0,5563	0,5044	0,4571	0,5537	0,6418	0,6168	0,6154	0,5240	0,4043	0,4368	0,4611	0,4739	0,4886	0,4980	0,3560	0,3830	0,4396	0,4891
BrumiR2ref	recall	0,6753	0,5573	0,5342	0,7295	0,2382	0,2553	0,6744	0,6695	0,3044	0,4386	0,8260	0,7395	0,5854	0,5634	0,5330	0,3765	0,6287	0,5778	0,3186	0,5748
	F-score	0,5763	0,5235	0,5450	0,5964	0,3132	0,3495	0,6577	0,6421	0,4074	0,4775	0,5428	0,5492	0,5159	0,5148	0,5098	0,4288	0,4546	0,4607	0,3694	0,5285
	precision	0,3299	0,3094	0,5324	0,5013	0,3167	0,8435	0,3892	0,3817	0,2749	0,1888	0,1475	0,1561	0,2728	0,2865	0,4240	0,4748	0,4044	0,4209	0,3537	0,3356
miRDeep2/miR-PREFeR	recall	0,3154	0,3146	0,2249	0,2596	0,1695	0,1979	0,9487	0,4195	0,1112	0,0817	0,8417	0,7873	0,6912	0,6549	0,6876	0,5940	0,6729	0,6806	0,8361	0,8032
	F-score	0,3225	0,3120	0,3162	0,3420	0,2208	0,3206	0,5520	0,3997	0,1584	0,1140	0,2510	0,2606	0,3912	0,3986	0,5245	0,5278	0,5052	0,5201	0,4971	0,4734
	precision	0,1610	0,3422	0,5922	0,5290	0,1826	0,1892	0,3066	0,0603	0,3414	0,0636	0,4176	0,3293	0,6222	0,3462	0,5512	0,5170	0,4783	0,5143	0,4500	0,4478
mirnovo	recall	0,3881	0,9161	0,9142	0,3041	0,9507	0,9753	0,7358	0,9624	0,9769	0,4551	0,5257	0,2070	0,4940	0,0181	0,4949	0,3470	0,5376	0,7168	0,5538	0,5646
	F-score	0,2275	0,4982	0,7188	0,3862	0,3064	0,3169	0,4329	0,1134	0,5059	0,1116	0,4654	0,2542	0,5507	0,0344	0,5215	0,4153	0,5062	0,5989	0,4965	0,4994

Table S5: Reduced benchmark 5 miRNA discovery tools.

			candidates	miRBase match (TP)	putative novel (FP)	match	false negative (FN)	accuracy	recall	F-score
miRanalyzer	Homo sapiens	ERR2403205	242	202	40	202	13028	0,835	0,015	0,030
		ERR2403208	256	219	37	219	14672	0,855	0,015	0,029
	Arabidopsis thaliana	SRR8992309	7	7	0	7	4510	1,000	0,002	0,003
		SRR8992311	4	4	0	4	5086	1,000	0,001	0,002
miRNAgFree	Homo sapiens	ERR2403205	230	193	37	5420	7810	0,839	0,410	0,551
		ERR2403208	276	223	53	5532	7698	0,808	0,418	0,551
	Arabidopsis thaliana	SRR8992309	18	7	11	141	4376	0,389	0,031	0,058
		SRR8992311	26	8	18	132	4958	0,308	0,026	0,048
miRDeep2/ miR-PREfeR	Homo sapiens	ERR2403205	579	457	122	5931	7299	0,789	0,448	0,572
		ERR2403208	711	536	175	6607	8284	0,754	0,444	0,559
	Arabidopsis thaliana	SRR8992309	311	110	201	3296	1221	0,354	0,730	0,476
		SRR8992311	441	148	293	3376	1714	0,336	0,663	0,446
mirnovo	Homo sapiens	ERR2403205	789	341	448	4378	8852	0,432	0,331	0,375
		ERR2403208	1017	704	313	11655	3236	0,692	0,783	0,735
	Arabidopsis thaliana	SRR8992309	120	54	66	2183	2334	0,450	0,483	0,466
		SRR8992311	134	60	74	2373	2717	0,448	0,466	0,457
BrumiR	Homo sapiens	ERR2403205	1244	761	483	10281	2949	0,612	0,777	0,685
		ERR2403208	1365	821	544	10517	4374	0,601	0,706	0,650
	Arabidopsis thaliana	SRR8992309	1285	435	850	3855	662	0,339	0,853	0,485
		SRR8992311	1531	515	1016	4591	499	0,336	0,902	0,490

Table S6: Total elapsed time per tool (seconds) on real datasets. The total elapsed time reported include only the core step of each algorithm.

	species	dataset	BrumiR	miRDeep2/miR-PREFeR
animal	Homo sapiens	ERR2403205	138	6319
		ERR2403208	151	5421
	Mus musculus	SRR1734814	92	8342
		SRR1734817	2828	11103
	Drosophila melagonaster	ERR3276595	141	5382
		ERR3276596	96	8270
	Danio rerio	SRR2127900	84	4921
		SRR2127901	94	5554
	Caenorhabditis elegans	ERR562743	138	10159
		ERR562747	122	8172
plant	Zea mays	SRR7801904	560	52875
		SRR7801905	611	13002
	Physcomitrella	SRR1842130	91	25909
		SRR1842134	82	39644
	Oryza sativa	SRR6456419	798	34346
		SRR6456420	382	17916
	Solanum lycopersicum	SRR6872534	1975	31902
		SRR6872539	1749	37629
	Arabidopsis thaliana	SRR8992309	264	4045
		SRR8992311	284	1802

Table S7: RNA transcriptome assemblers comparison.

			velvet				Trinity-inchworn (k-mer=14)				BrumiR(k-mer=14)		
			contigs	filter by length <25	candidates with a match in miRBase	precision	contigs	filter by length <25	candidates with a match in miRBase	precision	candidates	candidates with a match in miRBase	precision
Homo sapiens	ERR2403205	k-mer 13	17288	4020	33	0,0082	98292	39722	16181	0,4074	1240	731	0,5895
		k-mer 15	9792	1135	30	0,0264							
	ERR2403208	k-mer 13	17080	4012	40	0,0100	113596	47103	18952	0,4024	1360	819	0,6022
		k-mer 15	9954	1031	33	0,0320							
Arabidopsis thaliana	SRR8992309	k-mer 13	14997	3156	19	0,0060	143970	52881	16897	0,3195	1284	583	0,4540
		k-mer 15	13539	1522	4	0,0026							
	SRR8992311	k-mer 13	14270	3030	23	0,0076	151355	54985	17659	0,3212	1528	683	0,4470
		k-mer 15	12534	1429	4	0,0028							

Table S8: Number of candidates per depth coverage.

Species	dataset	coverage	candidates
Homo sapiens	*ERR2403205*	d10	1247
		d20	966
		d50	602
		d80	672
		d100	603
		d120	552
		d200	445
	ERR2403208	d10	1372
		d20	1046
		d50	632
		d80	694
		d100	635
		d120	574
		d200	439
Arabidopsis thaliana	*SRR8992309*	d10	1272
		d20	899
		d50	480
		d80	562
		d100	481
		d120	422
		d200	288
	SRR8992311	d10	1534
		d20	1282
		d50	557
		d80	655
		d100	560
		d120	483
		d200	338

Table S9: Random Forest classifier benchmark to evaluate the performance BrumiR.

		Homo sapiens		Mus musculus		Drosophila melagonaster		Danio rerio		Caenorhabditis elegans		Zea mays		Physcomitrella		Oryza sativa		Solanum lycopersicum		Arabidopsis thaliana	
		ERR2403205	ERR2403208	SRR1734814	SRR1734817	ERR3276595	ERR3276596	SRR2127900	SRR2127901	ERR562743	ERR562747	SRR7801904	SRR7801905	SRR1842130	SRR1842134	SRR6456419	SRR6456420	SRR6872534	SRR6872539	SRR8992309	SRR8992311
	reads	23787456	23195170	3492694	39444355	15420047	11770115	5964199	5813773	7109534	18014441	14963874	15958874	8258652	7381000	14099053	13193443	21093873	18211752	36783111	32827606
	processed reads	21837154	20248581	3483185	39349213	15300462	11690069	5766858	5526102	6666247	10723675	11698286	11770953	7965525	7144623	13263168	11582302	19827894	17343516	36070219	32512424
	map to the reference genome	15397405	15468470	1686235	18956030	7930708	6611362	4025133	3580407	5793813	8859905	2732893	3447092	4093364	3194414	8949699	6560968	11143699	10163965	33816174	29856834
	%mapped (refrence genome approach)	70,51	76,39	48,41	48,17	51,83	56,56	69,80	64,79	86,91	82,62	23,36	29,29	51,39	44,71	67,48	56,65	56,20	58,60	93,75	91,83
	unipaths (BrumiR approach)	122147	133437	92975	1145535	119292	82120	65788	73113	109844	67813	399293	398490	88192	79899	590548	342118	1376168	1304218	213899	226501
	candidates	602	632	249	934	318	266	304	330	391	514	497	608	565	478	588	466	1418	1284	480	557
BrumiR	candidates with match (true positives)	265	282	124	347	105	100	191	188	160	177	181	237	252	215	246	201	413	418	170	200
	match in MirGeneDB/ miRBase	6352	6391	5831	8304	1929	1912	8060	7998	2284	2211	3437	3328	2138	2247	3543	4424	4343	3720	1659	2711
	candidates	374	391	160	567	140	121	201	214	169	208	376	435	386	306	350	247	778	731	182	229
BrumiR2ref	candidates with match (true positives)	188	193	89	286	64	67	129	132	104	109	152	190	178	145	171	123	277	280	80	112
	match in MirGeneDB/ miRBase	4987	4833	4660	6880	1464	1459	6545	6537	1029	1299	3314	3126	1748	1742	2486	2207	3410	3059	1256	2416
	candidates	352	364	145	490	138	132	218	226	220	260	216	252	293	247	304	215	679	619	214	269
BrumiR-RF	candidates with match (true positives)	232	243	114	289	80	86	173	165	128	137	100	111	161	135	161	118	254	246	93	117
	match in MirGeneDB/ miRBase	6233	6336	5805	8230	1904	1893	8015	7953	2257	2148	3281	3096	1938	1771	2898	2748	3682	3186	1471	2492
	candidates	579	711	278	383	120	115	537	579	171	196	4401	3158	4769	3798	5569	3774	3257	3001	311	441
miRDeep2/miR-PREFeR	candidates with match (true positives)	191	220	148	192	38	97	209	221	47	37	649	493	1301	1088	2361	1792	1317	1263	110	148
	match in miRBase	2329	2728	1962	2448	5104	1131	9207	4096	376	242	3377	3328	2064	2025	3207	3482	3650	3603	3296	3376
	candidates	789	1017	358	259	5476	4181	5476	4181	955	2642	91	82	90	78	127	147	230	210	120	134
mirnovo	candidates with match (true positives)	127	348	212	137	1000	791	1679	252	326	168	38	27	56	27	70	76	110	108	54	60
	match in miRBase	2866	7944	7975	2868	4476	3390	3797	9397	3302	1348	2109	875	1475	56	2308	2034	2916	3795	2183	2373
	precision	0,4385	0,4462	0,4980	0,3715	0,3302	0,3759	0,6283	0,5697	0,4092	0,3444	0,3642	0,3898	0,4460	0,4498	0,4184	0,4313	0,2913	0,3255	0,3542	0,3591
BrumiR	recall	0,8601	0,7370	0,6685	0,8805	0,3139	0,3346	0,8305	0,8191	0,6757	0,7465	0,8567	0,7873	0,7160	0,7267	0,7596	0,7547	0,8007	0,7027	0,4209	0,6450
	F-score	0,5809	0,5559	0,5708	0,5226	0,3218	0,3540	0,7154	0,6720	0,5097	0,4713	0,5111	0,5214	0,5496	0,5557	0,5396	0,5489	0,4271	0,4449	0,3846	0,4613
	precision	0,5027	0,4936	0,5563	0,5044	0,4571	0,5537	0,6418	0,6168	0,6154	0,5240	0,4043	0,4368	0,4611	0,4739	0,4886	0,4980	0,3560	0,3830	0,4396	0,4891
BrumiR2ref	recall	0,6753	0,5573	0,5342	0,7295	0,2382	0,2553	0,6744	0,6695	0,3044	0,4386	0,8260	0,7395	0,5854	0,5634	0,5330	0,3765	0,6287	0,5778	0,3186	0,5748
	F-score	0,5763	0,5235	0,5450	0,5964	0,3132	0,3495	0,6577	0,6421	0,4074	0,4775	0,5428	0,5492	0,5159	0,5148	0,5098	0,4288	0,4546	0,4607	0,3694	0,5285
	precision	0,6591	0,6676	0,7862	0,5898	0,5797	0,6515	0,7936	0,7301	0,5818	0,5269	0,4630	0,4405	0,5495	0,5466	0,5296	0,5488	0,3741	0,3974	0,4346	0,4349
BrumiR-RF	recall	0,8440	0,7306	0,6655	0,8727	0,3098	0,3312	0,8259	0,8145	0,6678	0,7252	0,8178	0,7324	0,6490	0,5728	0,6214	0,4688	0,6788	0,6018	0,3732	0,5929
	F-score	0,7402	0,6977	0,7208	0,7039	0,4038	0,4392	0,8094	0,7700	0,6218	0,6104	0,5912	0,5501	0,5951	0,5594	0,5718	0,5057	0,4824	0,4787	0,4015	0,5018
	precision	0,3299	0,3094	0,5324	0,5013	0,3167	0,8435	0,3892	0,3817	0,2749	0,1888	0,1475	0,1561	0,2728	0,2865	0,4240	0,4748	0,4044	0,4209	0,3537	0,3356
miRDeep2/miR-PREFeR	recall	0,3154	0,3146	0,2249	0,2596	0,1695	0,1979	0,9487	0,4195	0,1112	0,0817	0,8417	0,7873	0,6912	0,6549	0,6876	0,5940	0,6729	0,6806	0,8361	0,8032
	F-score	0,3225	0,3120	0,3162	0,3420	0,2208	0,3206	0,5520	0,3997	0,1584	0,1140	0,2510	0,2606	0,3912	0,3986	0,5245	0,5278	0,5052	0,5201	0,4971	0,4734
	precision	0,1610	0,3422	0,5922	0,5290	0,1826	0,1892	0,3066	0,0603	0,3414	0,0636	0,4176	0,3293	0,6222	0,3462	0,5512	0,5170	0,4783	0,5143	0,4500	0,4478
mirnovo	recall	0,3881	0,9161	0,9142	0,3041	0,9507	0,9753	0,7358	0,9624	0,9769	0,4551	0,5257	0,2070	0,4940	0,0181	0,4949	0,3470	0,5376	0,7168	0,5538	0,5646
	F-score	0,2275	0,4982	0,7188	0,3862	0,3064	0,3169	0,4329	0,1134	0,5059	0,1116	0,4654	0,2542	0,5507	0,0344	0,5215	0,4153	0,5062	0,5989	0,4965	0,4994

Table S10: miRNA discovery from the root samples of *Arabidopsis thaliana* using BrumiR.

		Raw reads (M)	Processed reads (M)	Unipaths	Candidates	Hairpin validated	Core predictions	Known miRNAs	Putative novel miRNAs
	1	22.19	2.10	79,719	142	94			
day 5	2	25.31	1.35	34,179	64	50	37	17	20
	3	25.77	2.97	88,129	140	101			
	4	24.65	15.60	513,818	1,168	701			
day 9	5	21.70	14.58	296,520	947	546	151	45	106
	6	24.66	14.46	620,215	1,597	1,069			
	7	29.30	19.63	489,036	1,316	734			
day 13	8	25.73	14.49	584,786	1,315	833	182	46	136
	9	27.94	20.22	959,711	1,763	1,186			
	10	21.77	13.44	303,980	971	551			
day 17	11	19.78	10.33	166,774	641	363	140	49	91
	12	16.49	9.55	500,078	1,275	834			
	13	24.42	17.80	779,801	1,877	1,116			
day 21	14	16.81	6.90	195,541	501	355	100	33	67
	15	22.54	5.54	144,097	462	295			
	16	24.19	16.31	779,977	2,013	1,277			
day 25	17	35.56	27.06	1,250,149	2,637	1,543	457	62	392
	18	26.05	18.21	931,565	2,109	1,307			

Table S11: Novel miRNAs in the root samples of *Arabidopsis thaliana* predicted by BrumiR.

miRID	chr:pos	mature sequence	precursor sequence
miR-8-core	chr5:22322674-22322784	AGGGTTTGGTCTCCAGAAAGT	TGCTTAAAATTTTCGTAATTTCTTCTTCTAAGCCTT<br>AGGTGGAGCAAGCCTTGGTGACTCGACAAAGTGAAGGGTT<br>TGGTCTCCAGAAAGTTTTCTTCCAAGGTTCAAGA
miR-15 9-13-17 days	chr4:15467134-15467293	ACTAGTCAATTCTGAGGGAT	TGACTGATTATACCTTGATTACTAGTCAATTCTGAGGGATTCTAT<br>CAGCTAAGCTAAATTCAATCCTTTTGCGCCATCTCTTTGTTTACTTAACTCT<br>CTATGTGCCCTTCGATGATTAATAATATATTAATGGTTAAAAGTAGTCTGATATCTTATTGA

Table S12: Novel microRNAs and their putative interactions obtained using psRNATarget. miRNA Acc.: microRNA identification; Target Acc.: mRNA target identification, linked to the Arabidopsis thaliana mRNA library with the Araport V11 genome annotation. Expectation: mismatches penalty between mature small RNA and the target sequence, the lower the value the better the prediction (with 5.0 as a maximum threshold). Inhibition: refers to the possible mechanisms used by the sRNA to regulate its mRNA target, described in plants. Target Desc: refers to the gene description for the mRNA target, found in the Araport V11 annotation. Multiplicity: indicates how many times a sRNA has a target sequence in a unique mRNA.

miRNA_Acc.	Target_Acc.	Expectation	Inhibition	Target_Desc.	Multiplicity
miR-15	AT1G64210.1	2.5	Cleavage	— Leucine-rich repeat protein kinase family protein — Chr1:23830688-23832863 FORWARD LENGTH=2109 — 201606	1
miR-8	AT5G49830.3	2.5	Cleavage	— exocyst complex component 84B — Chr5:20250486-20255039 REVERSE LENGTH=2942 — 201606	1
miR-8	AT3G60570.2	2.5	Cleavage	— expansin B5 — Chr3:22391475-22392640 FORWARD LENGTH=844 — 201606	1
miR-8	AT4G15020.2	2.5	Cleavage	— hAT transposon superfamily — Chr4:8575342-8578729 FORWARD LENGTH=2920 — 201606	1
miR-8	AT4G15020.1	2.5	Cleavage	— hAT transposon superfamily — Chr4:8575409-8578688 FORWARD LENGTH=3020 — 201606	1
miR-8	AT5G35210.1	2.5	Cleavage	— PHD type transcription factor with transmembrane domain protein (PTM) — Chr5:13474196-13482591 REVERSE LENGTH=5856 — 201606	2
miR-15	AT5G01290.1	3.0	Cleavage	— mRNA capping enzyme family protein — Chr5:117297-121569 FORWARD LENGTH=2374 — 201606	1
miR-15	AT5G60040.2	3.0	Cleavage	— nuclear RNA polymerase C1 — Chr5:24173259-24183298 FORWARD LENGTH=4428 — 201606	1
miR-15	AT5G43530.1	3.0	Cleavage	— Helicase protein with RING/U-box domain-containing protein — Chr5:17489271-17495024 FORWARD LENGTH=4084 — 201606	1
miR-8	AT2G47900.2	3.0	Cleavage	— tubby like protein 3 — Chr2:19610943-19613219 REVERSE LENGTH=1628 — 201606	1
miR-8	AT2G47900.1	3.0	Cleavage	— tubby like protein 3 — Chr2:19610943-19613325 REVERSE LENGTH=2033 — 201606	1
miR-8	AT3G50860.1	3.0	Cleavage	— Clathrin adaptor complex small chain family protein — Chr3:18901997-18904197 FORWARD LENGTH=1088 — 201606	1
miR-8	AT5G20010.1	3.5	Cleavage	— RAS-related nuclear protein-1 — Chr5:6760094-6762330 FORWARD LENGTH=1519 — 201606	1
miR-8	AT1G65840.1	3.5	Translation	— polyamine oxidase 4 — Chr1:24489758-24493077 FORWARD LENGTH=2258 — 201606	1
miR-8	AT2G13540.1	3.5	Cleavage	— ARM repeat superfamily protein — Chr2:5636884-5643066 FORWARD LENGTH=2969 — 201606	1
miR-8	AT5G06960.2	3.5	Cleavage	— OCS-element binding factor 5 — Chr5:2154746-2157719 FORWARD LENGTH=1677 — 201606	1
miR-8	AT3G50740.1	3.5	Cleavage	— UDP-glucosyl transferase 72E1 — Chr3:18855043-18857040 REVERSE LENGTH=1998 — 201606	1
miR-8	AT1G22275.3	3.5	Cleavage	— Myosin heavy chain-related protein — Chr1:7867133-7870615 FORWARD LENGTH=2132 — 201606	1
miR-8	AT1G22260.2	3.5	Cleavage	— Myosin heavy chain-related protein — Chr1:7860160-7864703 REVERSE LENGTH=2468 — 201606	1
miR-8	AT1G03650.1	3.5	Cleavage	— Acyl-CoA N-acyltransferases (NAT) superfamily protein — Chr1:910023-911292 REVERSE LENGTH=892 — 201606	1
miR-8	AT3G59240.1	3.5	Translation	— RNI-like superfamily protein — Chr3:21898682-21900364 REVERSE LENGTH=1515 — 201606	1
miR-8	AT3G46550.1	3.5	Cleavage	— Fasciclin-like arabinogalactan family protein — Chr3:17136346-17138259 REVERSE LENGTH=1914 — 201606	1
miR-8	AT5G52530.2	3.5	Cleavage	— dentin sialophosphoprotein-like protein — Chr5:21317130-21321267 FORWARD LENGTH=3311 — 201606	1
miR-8	AT1G07650.1	3.5	Cleavage	— Leucine-rich repeat transmembrane protein kinase — Chr1:2359111-2366736 REVERSE LENGTH=4064 — 201606	1
miR-8	AT4G15417.1	3.5	Cleavage	— RNAse II-like 1 — Chr4:8821690-8823076 FORWARD LENGTH=949 — 201606	2
miR-8	AT4G15417.2	3.5	Cleavage	— RNAse II-like 1 — Chr4:8821651-8823076 FORWARD LENGTH=988 — 201606	2
miR-8	AT5G60610.2	3.5	Translation	— F-box/RNI-like superfamily protein — Chr5:24363933-24365565 FORWARD LENGTH=1462 — 201606	1
miR-8	AT5G16890.1	3.5	Cleavage	— Exostosin family protein — Chr5:5551627-5554919 FORWARD LENGTH=1751 — 201606	1
miR-15	AT3G09100.1	4.0	Cleavage	— mRNA capping enzyme family protein — Chr3:2789548-2793532 REVERSE LENGTH=2214 — 201606	1
miR-15	AT4G33990.1	4.0	Cleavage	— Tetratricopeptide repeat (TPR)-like superfamily protein — Chr4:16289686-16292612 REVERSE LENGTH=2927 — 201606	1
miR-15	AT1G64750.3	4.0	Translation	— deletion of SUV3 suppressor 1(I) — Chr1:24052362-24053908 REVERSE LENGTH=879 — 201606	1
miR-15	AT2G46940.1	4.0	Cleavage	— fold protein — Chr2:19286434-19287590 REVERSE LENGTH=1068 — 201606	1
miR-15	AT4G11890.4	4.0	Cleavage	— Protein kinase superfamily protein — Chr4:7148386-7149942 FORWARD LENGTH=1209 — 201606	1
miR-15	AT1G34180.3	4.0	Cleavage	— NAC domain containing protein 16 — Chr1:12448578-12450575 FORWARD LENGTH=1520 — 201606	1
miR-15	AT4G11890.2	4.0	Cleavage	— Protein kinase superfamily protein — Chr4:7147865-7149921 FORWARD LENGTH=1612 — 201606	1
miR-15	AT1G22310.1	4.0	Translation	— methyl-CPG-binding domain 8 — Chr1:7881522-7883824 REVERSE LENGTH=1876 — 201606	1
miR-15	AT4G15230.4	4.0	Cleavage	— pleiotropic drug resistance 2 — Chr4:8679901-8687230 FORWARD LENGTH=4794 — 201606	1
miR-15	AT4G27630.7	4.0	Cleavage	— GPCR-type G protein 2 — Chr4:13792546-13797147 REVERSE LENGTH=1504 — 201606	1
miR-15	AT5G27120.1	4.0	Cleavage	— NOP56-like pre RNA processing ribonucleoprotein — Chr5:9540861-9544065 FORWARD LENGTH=2114 — 201606	1
miR-15	AT1G13630.1	4.0	Cleavage	— Tetratricopeptide repeat (TPR)-like superfamily protein — Chr1:4669784-4672826 REVERSE LENGTH=2421 — 201606	1
miR-15	AT3G22830.1	4.0	Translation	— heat shock transcription factor A6B — Chr3:8078789-8081059 FORWARD LENGTH=1577 — 201606	1
miR-15	AT1G70320.1	4.0	Cleavage	— ubiquitin-protein ligase 2 — Chr1:26488454-26501652 REVERSE LENGTH=11639 — 201606	1
miR-15	AT1G16910.1	4.0	Cleavage	— LIGHT-DEPENDENT SHORT HYPOCOTYLS-like protein (DUF640) — Chr1:5785003-5785959 FORWARD LENGTH=957 — 201606	1
miR-15	AT4G21590.2	4.0	Cleavage	— endonuclease 3 — Chr4:11480329-11482724 FORWARD LENGTH=1137 — 201606	1
miR-15	AT4G14590.1	4.0	Translation	— embryo defective 2739 — Chr4:8374391-8376353 FORWARD LENGTH=1918 — 201606	1
miR-15	AT4G33510.2	4.0	Cleavage	— 3-deoxy-d-arabino-heptulosonate 7-phosphate synthase — Chr4:16116447-16118825 FORWARD LENGTH=2085 — 201606	1
miR-15	AT4G33460.1	4.0	Translation	— ABC transporter family protein — Chr4:16098070-16100440 REVERSE LENGTH=1398 — 201606	1
miR-8	AT4G05450.2	4.0	Cleavage	— mitochondrial ferredoxin 1 — Chr4:2758997-2760912 FORWARD LENGTH=923 — 201606	1
miR-8	AT1G49660.1	4.0	Cleavage	— carboxyesterase 5 — Chr1:18378634-18379901 REVERSE LENGTH=1268 — 201606	1
miR-8	AT1G07780.13	4.0	Translation	— phosphoribosylanthranilate isomerase 1 — Chr1:2410108-2412677 REVERSE LENGTH=1652 — 201606	1
miR-8	AT1G17840.1	4.0	Cleavage	— white-brown complex-like protein — Chr1:6142402-6146521 FORWARD LENGTH=3207 — 201606	1
miR-8	AT4G28130.1	4.0	Translation	— diacylglycerol kinase 6 — Chr4:13971552-13974323 FORWARD LENGTH=1401 — 201606	1
miR-8	AT3G06710.1	4.0	Translation	— E3 ubiquitin ligase — Chr3:2118169-2120181 FORWARD LENGTH=1631 — 201606	1
miR-8	AT3G21160.1	4.0	Cleavage	— alpha-mannosidase 2 — Chr3:7413776-7418476 REVERSE LENGTH=2220 — 201606	1
miR-8	AT2G31610.1	4.0	Cleavage	— Ribosomal protein S3 family protein — Chr2:13450241-13451982 FORWARD LENGTH=1209 — 201606	1
miR-8	AT5G20020.1	4.0	Cleavage	— RAS-related GTP-binding nuclear protein 2 — Chr5:6762569-6764683 FORWARD LENGTH=1216 — 201606	1
miR-8	AT4G00940.2	4.0	Cleavage	— Dof-type zinc finger DNA-binding family protein — Chr4:402959-404670 REVERSE LENGTH=1458 — 201606	1
miR-8	AT1G32270.2	4.0	Cleavage	— syntaxin — Chr1:11642593-11645237 FORWARD LENGTH=1473 — 201606	1
miR-8	AT1G01880.4	4.0	Cleavage	— 5'-3' exonuclease family protein — Chr1:306558-308602 REVERSE LENGTH=1634 — 201606	1
miR-8	AT4G31550.2	4.0	Cleavage	— WRKY DNA-binding protein 11 — Chr4:15289788-15291934 REVERSE LENGTH=1728 — 201606	1
miR-8	AT1G01880.2	4.0	Cleavage	— 5'-3' exonuclease family protein — Chr1:306480-309109 REVERSE LENGTH=1993 — 201606	1
miR-8	AT2G23200.1	4.0	Cleavage	— Protein kinase superfamily protein — Chr2:9879275-9881924 FORWARD LENGTH=2650 — 201606	1
miR-8	AT3G07980.3	4.0	Cleavage	— mitogen-activated protein kinase kinase kinase 6 — Chr3:2543611-2550458 REVERSE LENGTH=4174 — 201606	1
miR-8	AT1G10020.1	4.0	Cleavage	— formin-like protein (DUF1005) — Chr1:3269539-3272110 REVERSE LENGTH=2164 — 201606	1
miR-8	AT5G56360.1	4.0	Cleavage	— calmodulin-binding protein — Chr5:22823189-22828062 REVERSE LENGTH=2453 — 201606	1
miR-8	AT5G50340.3	4.0	Cleavage	— DNA repair protein RadA-like protein — Chr5:20491417-20496589 REVERSE LENGTH=2533 — 201606	1
miR-8	AT2G39340.1	4.0	Cleavage	— SAC3/GANP/Nin1/mts3/eIF-3 p25 family — Chr2:16423541-16430108 FORWARD LENGTH=3740 — 201606	1
miR-8	AT1G47810.1	4.0	Translation	— F-box and associated interaction domains-containing protein — Chr1:17603504-17604559 FORWARD LENGTH=1056 — 201606	1
miR-8	AT5G17100.1	4.0	Cleavage	— Cystatin/monellin superfamily protein — Chr[illegible]5623021-5624800 REVERSE LENGTH=1108 — 201606	1
miR-8	AT1G02560.1	4.0	Cleavage	— nuclear encoded CLP protease 5 — Chr1:537740-540127 FORWARD LENGTH=1479 — 201606	1
miR-8	AT1G16720.1	4.0	Cleavage	— high chlorophyll fluorescence phenotype 173 — Chr1:5722891-5726478 FORWARD LENGTH=2297 — 201606	1
miR-8	AT3G07020.1	4.0	Cleavage	— UDP-Glycosyltransferase superfamily protein — Chr3:2217622-2221867 REVERSE LENGTH=2410 — 201606	1
miR-8	AT1G77590.2	4.0	Cleavage	— long chain acyl-CoA synthetase 9 — Chr1:29148256-29152364 REVERSE LENGTH=2669 — 201606	1
miR-8	AT1G08600.1	4.5	Cleavage	— P-loop containing nucleoside triphosphate hydrolases superfamily protein — Chr1:2723824-2733613 FORWARD LENGTH=4809 — 201606	1
miR-15	AT3G62630.1	5.0	Translation	— stress response NST1-like protein (DUF1645) — Chr3:23163747-23165387 REVERSE LENGTH=1449 — 201606	1
miR-15	AT5G26940.4	5.0	Cleavage	— Polynucleotidyl transferase%2C ribonuclease H-like superfamily protein — Chr5:9480308-9482818 FORWARD LENGTH=1590 — 201606	1
miR-15	AT4G09570.1	5.0	Cleavage	— calcium-dependent protein kinase 4 — Chr4:6049441-6052380 FORWARD LENGTH=1821 — 201606	1

Table S13: piRNAs-db total entries:54865 match entries:345 . . . .

	candidates	match piRNAs	entries piRNAs db
BrumiR	2954	117	4%
miRDeep2	383	12	3%
mirnovo	785	78	10%

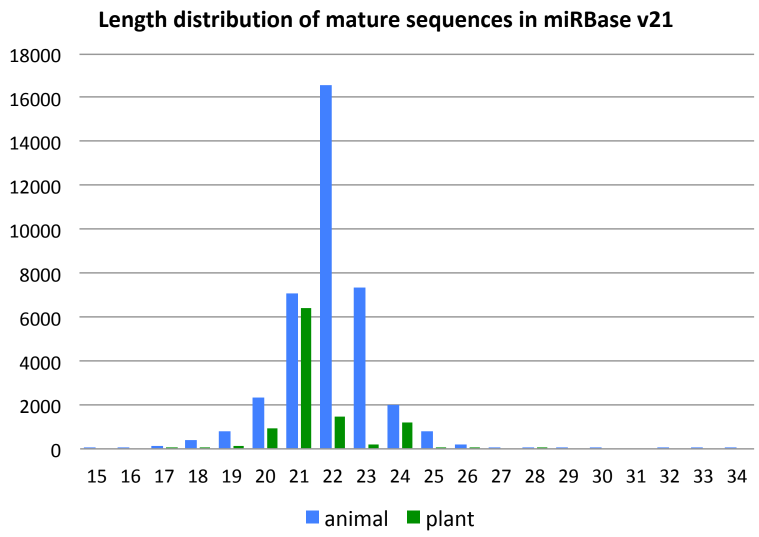

Figure S1: Length distribution of mature sequences in miRBase. We used miRBase v21 with a total of 35828 entries and we observed that the length is between 19-24 nt.

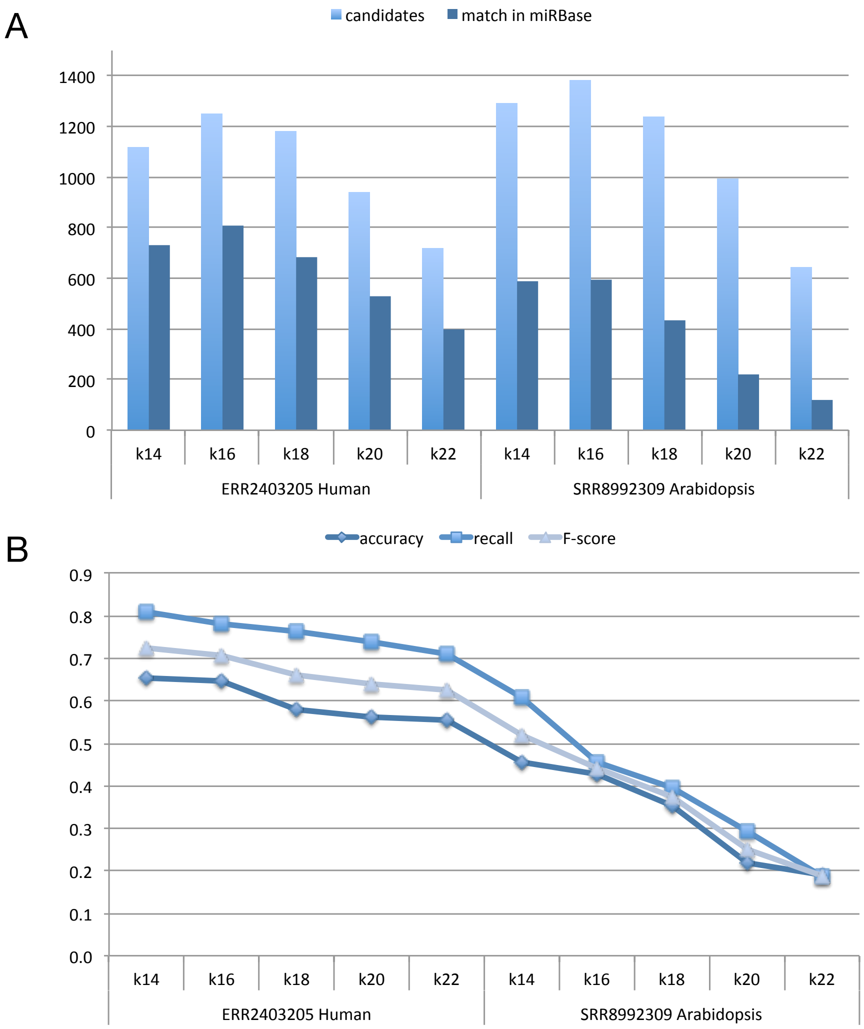

Figure S2: Determination of the optimal BrumiR k-mer size. A) Number of candidates predicted and matching mriBase entries as a fuction of the k-mer size for an animal and plant dataset. B) Benchmark metric computed for each k-mer size evaluated. The best performance is obtained at k-mer=14.

A

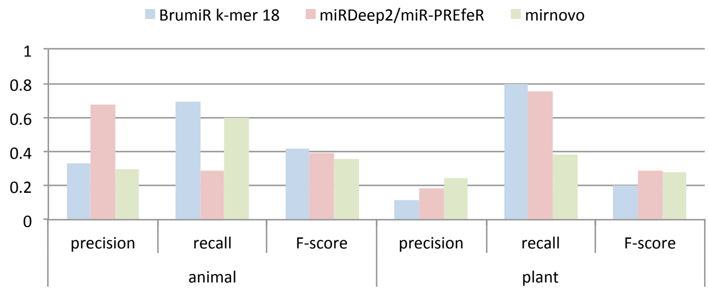

B

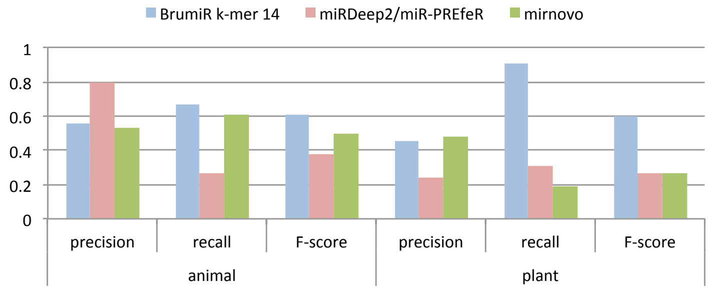

Figure S3: Benchmark metric comparison for animals and plants using diferent miRNA discovery tools and two BrumiR k-mer sizes (14-mers and 18-mers). The top and bottom plots shown the average of precision, recall and F-Score across the animal and plant datasets for BrumiR (k=18 and k=14), miRdeeep2/Mir-PREFER and mirnovo. The best performance for BrumiR is achieved when using a k-mer size of 14.

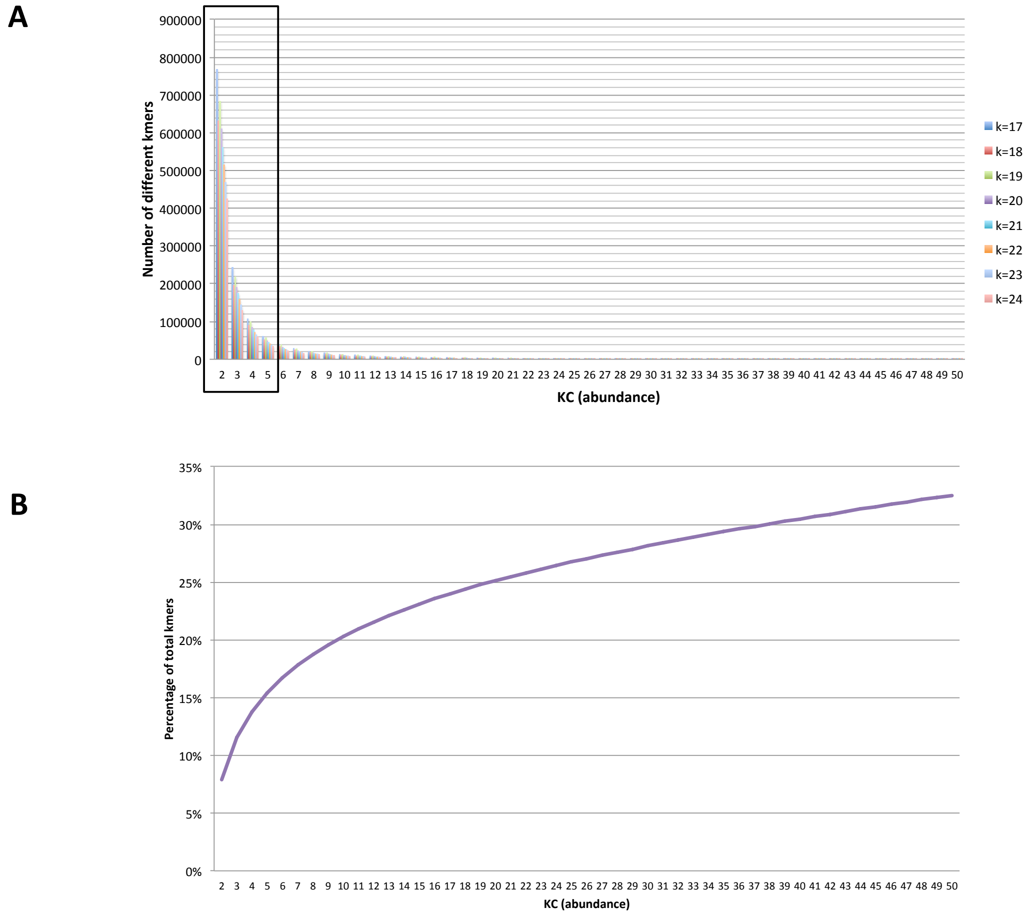

Figure S4: Kmer spectrum of sRNA-seq data. A) The histogram shows the number of distinct kmers (Y-axis) as a function of the read coverage (KC X-axis). In the lower coverage of the spectrum (black rectangle), we observe a high number of distinct kmers which are likely sequencing errors. The kmers that correspond to noise represent approximately less than 15% of the total number of kmers.

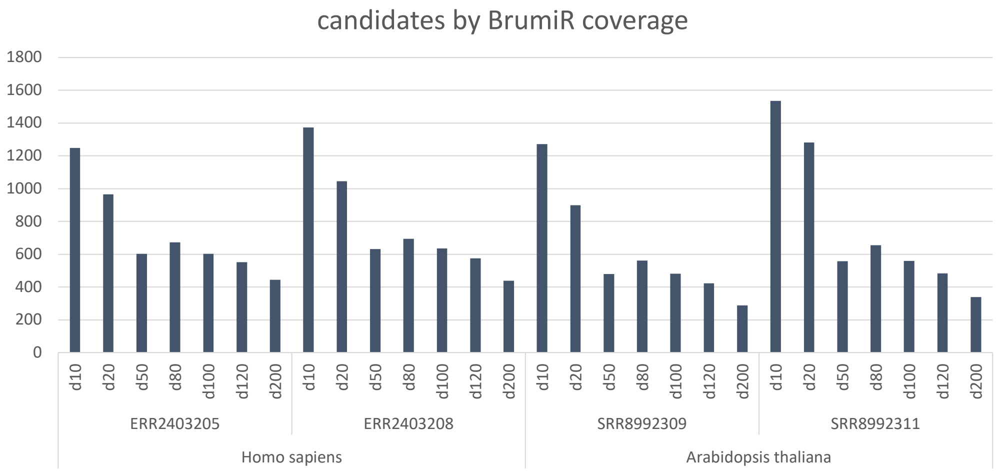

Figure S5: Comparing the performance of BrumiR using different depth coverage values.

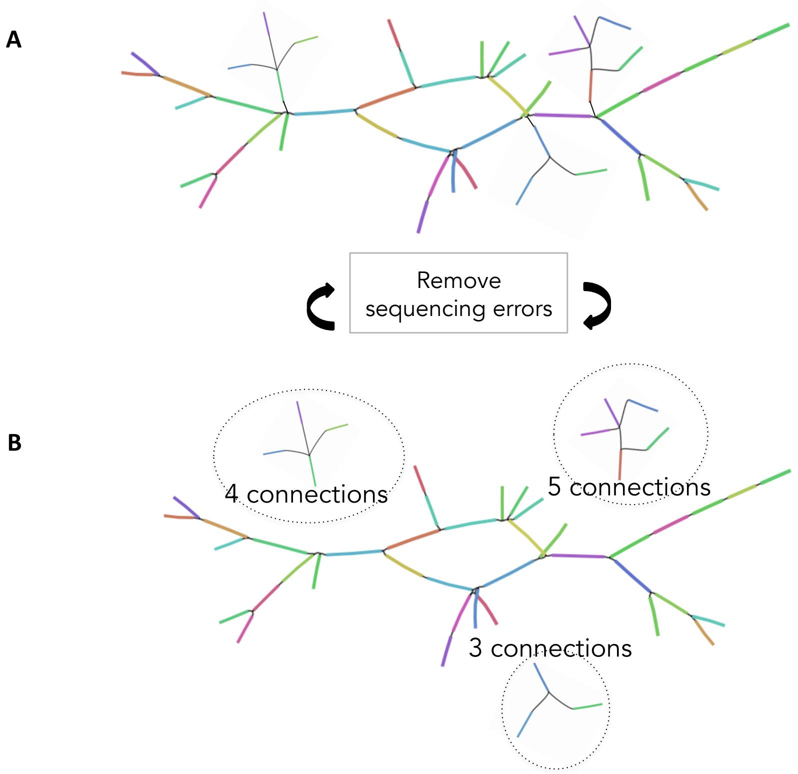

Figure S6: BrumiR classifies low abundance non-linear topologies as sequencing errors. A) BrumiR identifies these topologies connected to the principal structures in the graph, which appear after the first tip removal steps of BrumiR. B) These topologies have low abundance (KM value) and are composed of branching nodes with 3, 4, or 5 connections.

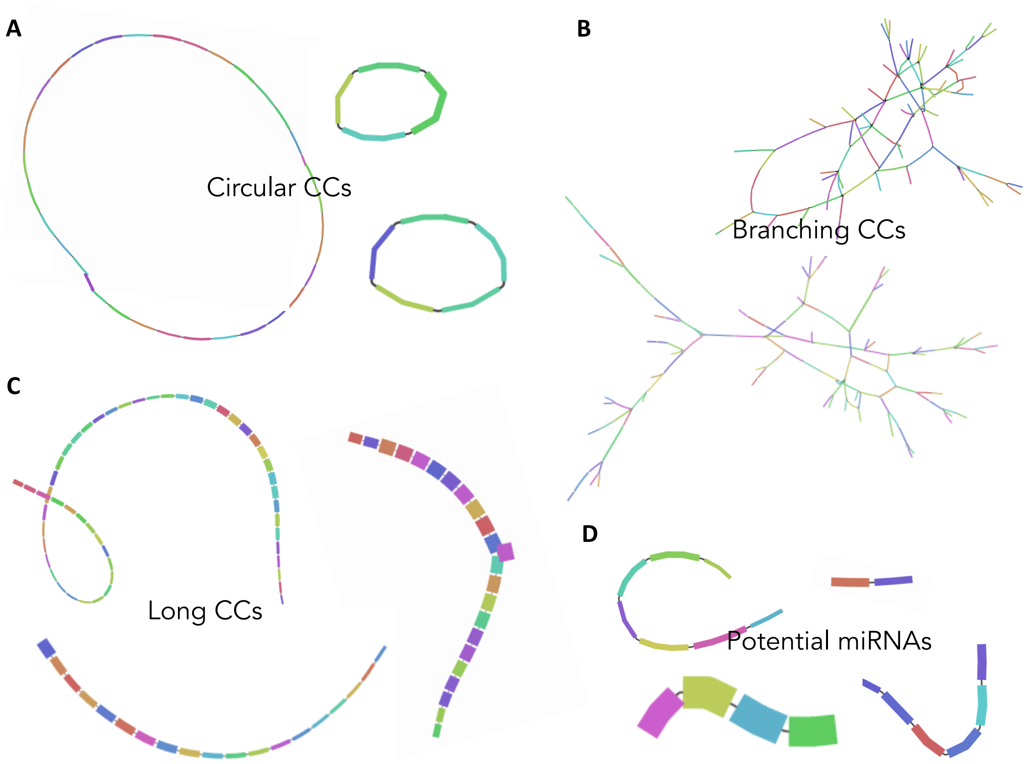

Figure S7: Re-assembling unipaths within each CC. BrumiR re-assembles all unipaths present in a linear CC by bundling the nodes with in and out degree equal to 1 into a new unipath. BrumiR rebuilds each unipath within a CC and classifies them into different types. A) Circular CCs: when all unipaths are have an in and out connection, we classify the CC as a circular sequence that is not a putative miRNA. B) Branching CCs: when we detect a CC with a high number of branching nodes, we do not consider it anymore for the moment, because we consider it related to sequencing errors (usually they have a low KM value). C) Long CCs: when we detect more than 10 unipaths, we can classify them as longer non coding sequences, but we still keep them for later analysis. D) Potential miRNAs: all assembled unipaths (CCs) having a length between 18 and 24 are stored as potential miRNA sequences.

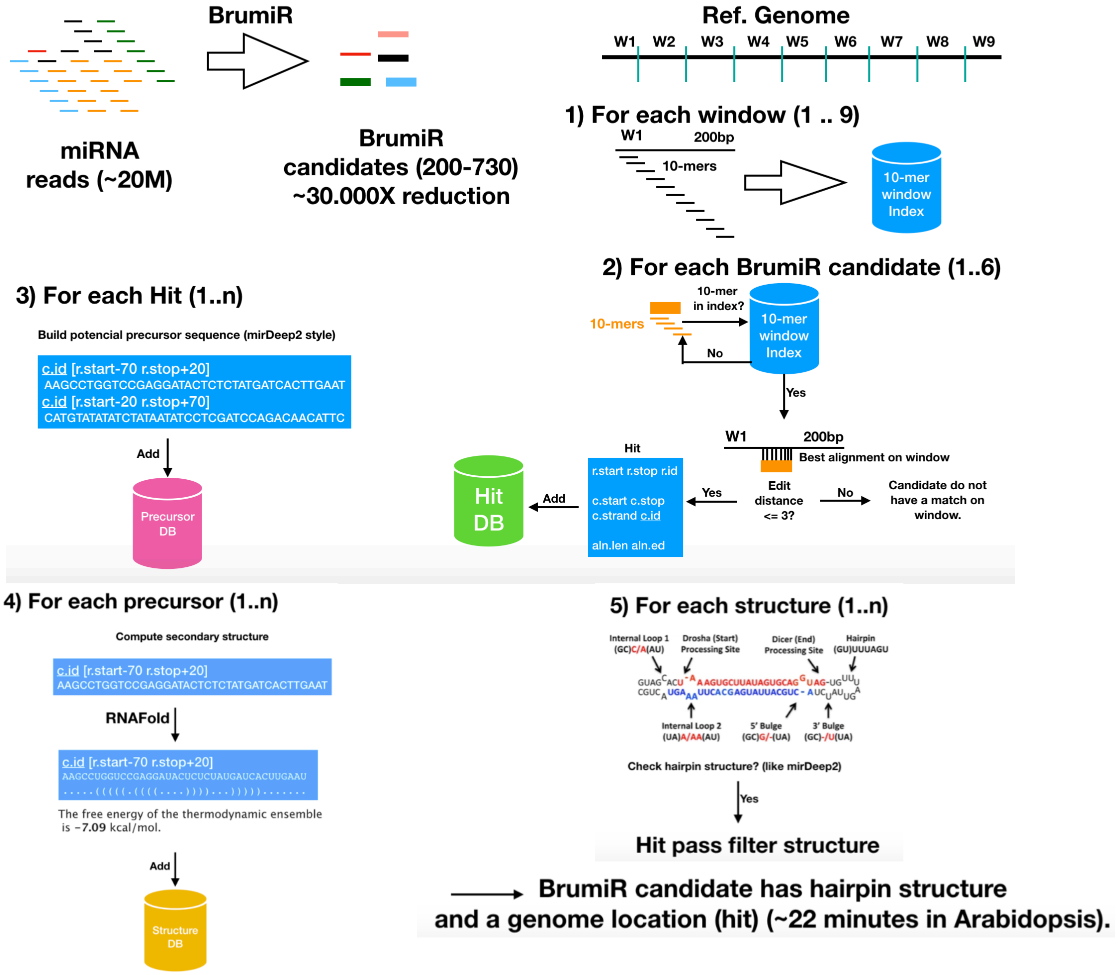

Figure S8: Workflow of the BrumiR2Reference tool. The main steps involved the mapping of the miRNA candidates to the genome using non-overlapping windows (1); each hit is further refined using an exhaustive alignment (2). For each hit, a precursor sequence is built (3), and its secondary structure is determined using RNAfold (4). Finally, structures fulfilling a set of criteria (5) are classified as precursor sequences.

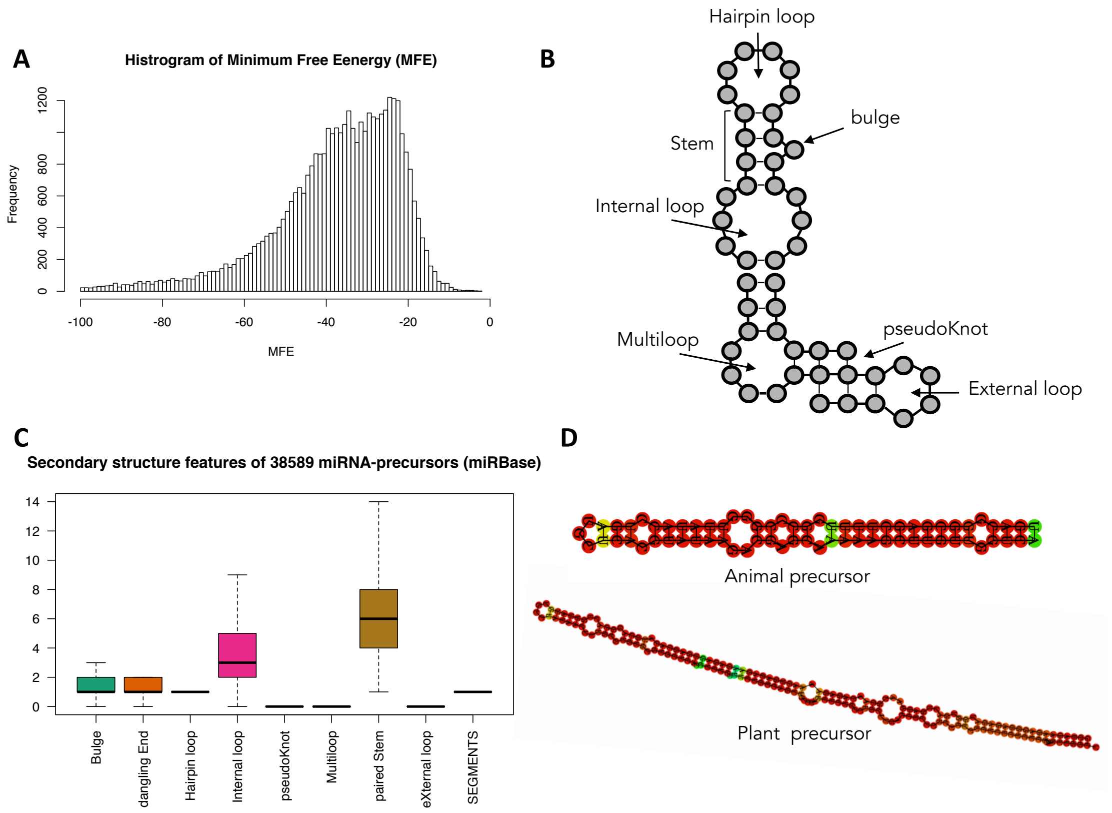

Figure S9: Structure properties of miRBase precursor sequences. A) Free-energy distribution of 38.589 precursor sequences folded with RNAfold. B) Different types of RNA secondary structure elements composing precursor miRNA sequences. C) Analysis of secondary structure elements performed on 38.589 precursor sequences in miRBase using the bpRNA package. D) Examples of precursor sequences for animal and plant species.

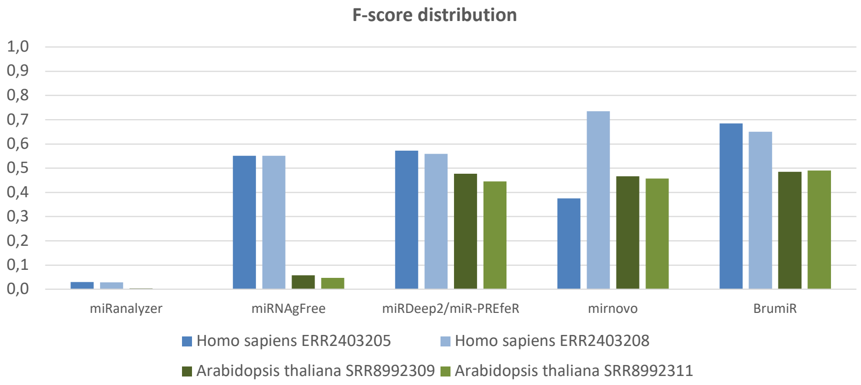

Figure S10: F-score distribution of reduced benchmark including 5 miRNA discovery tools.

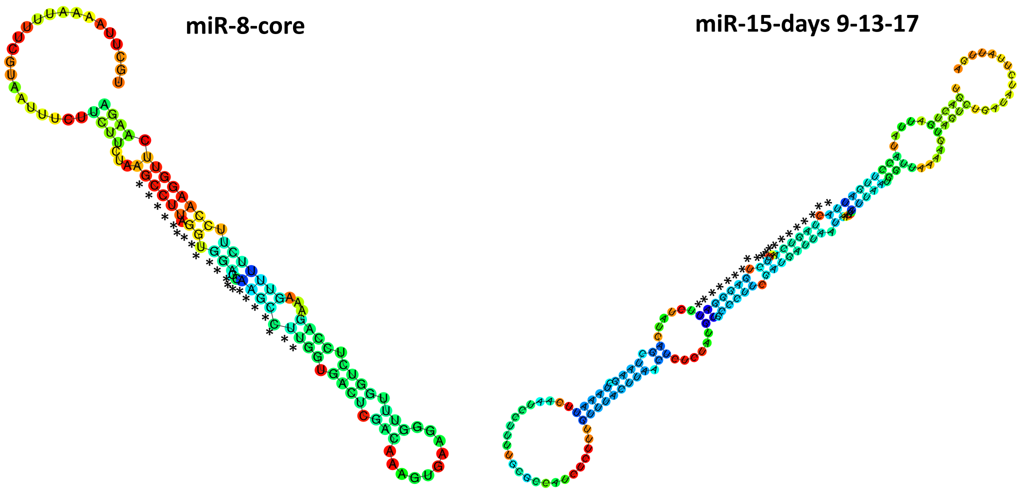

Plant criteria **miR-8-core**:
1.1 Single miRNA:miRNA* duplex
1.2 No secondary stems or large loops
1.3 Precursor size < 300 nt (111 nt)
2. Expression confirmed by sRNA-seq (expressed in all replicates)
3. Up to 5 mismatched positions (5 mismatched)
4. Average precision >90% confirmed by the reads (**98%**)
5. Replication: **Yes (confirmed by all conditions)**
6. Annotation by homology to miRNA families: **No**
7. miRNA length: 21 nt

Plant criteria **miR-15-days 9-13-17**:
1.1 Single miRNA:miRNA* duplex
1.2 No secondary stems or large loops
1.3 Precursor zise < 300 nt (159 nt)
2. Expression confirmed by sRNA-seq (expressed in all replicates)
3. Up to 5 mismatched positions (4 mismatched)
4. Average precision >90% confirmed by the reads (**95%**)
5. Replication: **Yes (confirmed by all conditions)**
6. Annotation by homology to miRNA families: **No**
7. miRNA length: 20 nt

Figure S11: Novel miRNAs candidates discovered by BrumiR using *Arabidopsis thaliana* root experiments that fulfill all the criteria to validate and annotate miRNAs in plants (Axtell Meyers, 2018).

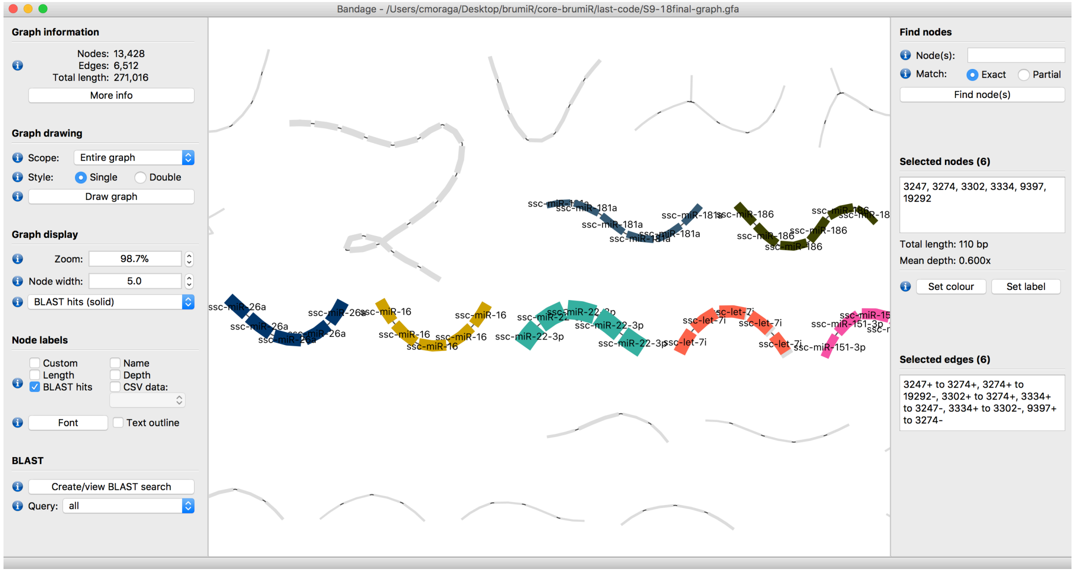

Figure S12: Visualization with Bandage. BrumiR provides an output compatible with the Bandage software, which can be employed to visualize and explore the results in a user-friendly way.

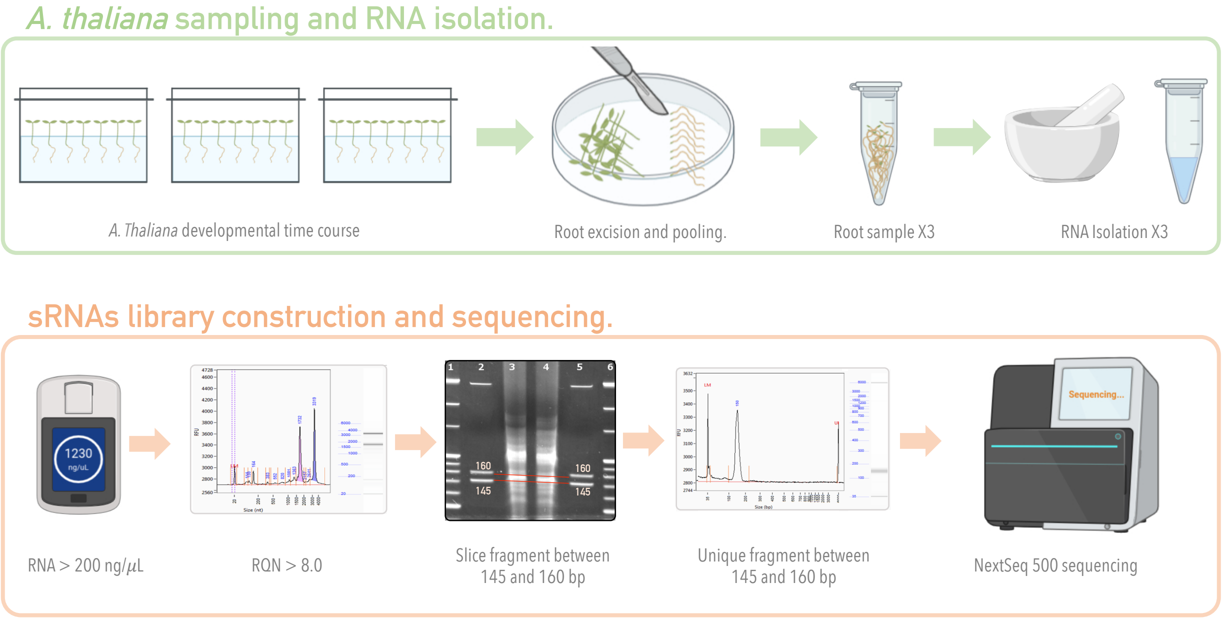

Figure S13: Experimental procedure of the *Arabidopsis thaliana* roots sampling and the sRNA-seq libraries construction. The seedlings for each sampling point are excised from their aerial shoots, the roots are pooled and stored in triplicates for RNA isolation. The RNAs are evaluated for concentration and integrity, above 200ng/uL, and an RQN score over 8.0 respectively, to begin a sRNA-Seq library construction. The 145-160 bp library was purified from polyacrylamide gels and validated as a unique fragment between 145-160 bp. Finally, the successful libraries were processed for next-generation sequencing (NGS) procedures. Representative AATI Fragment Analyzer electropherograms are shown for RNA integrity and sequencing library validation; LM, a lower marker at 20 nt and 35 bp; UM: upper marker at 4,000 nt and 6,000 bp. Polyacrylamide gel electrophoresis (PAGE) of reverse-transcribed cDNAs from small RNAs are shown. Red lines indicate fragments of interest (including sRNAs and miRNAs). Extraction procedures for fragments from PAGE are described in the library construction procedures by the manufacturer. * Figure partially created with BioRender.com.
