# Supplementary material for: BrumiR: A toolkit for de novo discovery of microRNAs from sRNA-seq data
Source: Gigascience. 2022 Oct 25;11:giac093. doi: 10.1093/gigascience/giac093 (PMC9596168; doi:10.1093/gigascience/giac093)
Supplement: giac093_Response_to_Reviewer_Comments_Original_Submission [file giac093_response_to_reviewer_comments_original_submission.pdf]

Author's Response To Reviewer Comments

Clo<u>s</u>e

5th November 2021
Dear Dr Nicole Nogoy,
Thank you very much for considering our manuscript for publication in GigaScience. We would like to thank the referees and you for the careful assessment of our manuscript. We have attempted to address all points raised by the referees and hope that the responses are satisfactory. With these revisions, we believe that our manuscript has been substantially improved and hope that it is now suitable for publication in GigaScience.
Please find below our point-by-point replies to the reviewers' comments. All changes in the main manuscript and the supplement have been marked in blue font, including all the Supplementary material. We refer to the changes we have made in the manuscript using consecutive line numbering.
Yours sincerely,
Carol Moraga

Reviewer #1: Summary:
The authors developed a de novo assembly method, BrumiR, for small RNA sequencing data based on de Bruijin graph algorithm. This tool displayed a relatively high sensitivity in finding miRNAs and helped the authors discover a novel miRNA in A. thaliana roots.
Major comments:
1. Have the authors compared the performance with different seed lengths? Even if the minimal miR length is 18nt in MiRBase 21, seed=18 might not necessarily lead to the best AUC or F score (This might also be related to Comment 4).

We followed the reviewer recommendations and we now compare the performance of BrumiR using different seed lengths of 14, 16, 18, 20, and 22; in both animal and plant datasets. The benchmark results are reported in Table S1. The benchmark shows that the optimal seed size for BrumiR is indeed 14 and not 18 as was previously set by default. The main reason for this improved performance is that shorter seed lengths permit a better handling of sequencing errors (shorter k-mers are less likely to be sequencing errors), and enable a more sensitive clustering of identical miRNAs. Overall, we observe that the benchmark metrics (Supplementary Table S1, Figure S2) improves in comparison to the observed for the previous seed length (k=18) by 1.67X , 0.97X, 1.46X for precision, recall, and F-score in animal species, and 4.09X, 1.13X, 3X for precision, recall, and F-score in plant species, respectively. In light of the results, we have now set the default seed length to k=14, and we thank the reviewer again for this key suggestion that allows us to further improve the performance of BrumiR. Finally, the code has been updated accordingly and a new release has been published in the Github repository.
The updated text is in page 8, lines 13; page 25, lines 18-22.

2. The authors need to benchmark BrumiR with more existing tools (e.g. those ML-based methods), and to include more genome-free methods (e.g. MiRNAgFree).

In our current benchmark, we included miRDeep2, miR-PREFeR and mirnovo, and a total of 10 animal and plant datasets. miRDeep2 is the most used tool in the field with more than 1530 citations and is considered the reference miRNA prediction tool for animal species. In addition, we included miR-PREFeR that, like miRDeep2, is one of the few tools designed specifically for plant species when a reference genome is available. Complementing these popular reference-based methods, we included mirnovo, which is a genome-free discovery tool based on machine learning. The mirnovo manuscript presents a benchmark comparison with miReader and miRplex which are de novo tools and mirnovo showed better performance than the aforementioned tools. This is one of our main reasons for considering mirnovo as the best tool for performing miRNA discovery without a reference genome. Therefore, we selected the tools that perform best for miRNA discovery, including reference and de novo based.

Nevertheless, we tried to follow the reviewer's suggestion and attempted to add more tools to our benchmark. However, we had difficulties in running the existing tools because most of them are not

under active development, do not provide update binaries (standalone versions), or their web-server does not work. For instance, miRplex does not provide recent binaries (the current one, v.01, is from 2012), leading to problems with some old libraries and we could not run it. In another example, the miReader software mirPlex is not in active development (updated for the last time in 2014). We were able to run the standalone version, but after letting it run for more than one month on a single sample, we decided to stop it.

Besides these issues, we succeeded in running the miRanalyzer (genome-reference based), and miRNAgFree (de novo based) tools. The benchmark results are provided in Supplementary Table S5 using a reduced version of the real dataset. The benchmark results show that BrumiR obtained the highest F-score rates, as do miRDeep2 and mirnovo (Supplementary FigureS13). We also observed the poor performance on plant species of most of the tools, which shows the potential of BrumiR, as well as being a tool that can be used for both animal and plant species.

Currently, there is no standard for benchmarking miRNA prediction methods, which means that each new method tries to make the comparison in its own way. For this reason, we tried to make an effort to create a benchmark standard and we developed miRsim to generate benchmarks based on a ground truth known a priori and using the same conditions for all the tools.

Overall, we wanted to benchmark BrumiR in a simple but exhaustive way selecting the top performers for genome-based and genome-free methods. Finally, several miRNA tools do not provide stand-alone versions and therefore prevent extensive benchmarking when only the server is available. Being able to generate a good benchmark remains a challenge in the field of miRNAs where there are no standards.

The updated text is in page 13, lines 17; page 14, lines 1-2; page 36, lines 11-16.

3. It is also interesting to know whether de novo method for mRNA assembly would be useful on the miRNA side. It would be great if the authors were able to compare the performance of BrumiR2reference (without filtering for RFAM) with Trinity in genome-guided mode, by tweaking its seed length to be the same as BrumiR.

We followed the reviewer’s recommendation and included two de Bruijn graph de novo transcriptome assemblers, namely Trinity (inchworm), and Velvet (Supplementary Table S7). The genome guide mode of Trinity first aligns the reads to the genome and then a de novo assembly is performed using the inchworm tool for each partition. The genome guide pipeline does not provide access to the seed length of inchworm (default k=25) whose default value exceeds the length of the miRNA sequences. Therefore, we ran it independently and without mapping the reads to the genome, because we thought that in this way the comparison between BrumiR and de novo transcriptome assemblers is more appropriate and allows us to focus on how the candidates are extracted from the de Bruijn graph. To perform the comparison, we used 4 real datasets; including human and Arabidopsis. The seed length and minimum contig length for the transcriptome assemblers were set to k=15 (edge-centric de Bruijn graph, overlap equals 14 nt), and l=18, for both inchworm and Velvet. Then, the contigs longer than 24 nt were filtered out for BrumiR (without filtering for RFAM) and the transcriptome de novo assemblers. Finally, the candidates were matched against miRBase (blast search) and the results of the comparison are presented in Supplementary Table S7. We can observe that the de novo transcriptome assemblers generated on average 40X and 4X more candidates than BrumiR, for Trinity and Velvet, respectively.

In general, the huge number of contigs generated by the transcriptome assemblers, even after filtering them by length, were poorly matched to miRBase entries (1,2% and 36%, indeed). On the other hand, BrumiR matched miRBase entries at a rate of 1 every 2 candidates (52% precision average). As expected, we can conclude that most of the contigs generated by a pure de Bruijn graph transcriptome assembler are poorly related to miRNA sequences. This was expected because they are developed for mRNAseq analysis and do not consider the complexities of the sRNA seq data like BrumiR.
In summary, this experiment showed that BrumiR and all the downstream steps it performs after the De Bruijn graph construction are essential for miRNA discovery.

The updated text is in page 15, lines 9-22; page 16, lines 1-3; page 33, lines 14-22; page 34, lines 1-5.

4. The tool's sensitivity is promising across animal and plant datasets. However, the average precision is quite low, an average precision of 0.3 means a false discovery rate of 0.7. This is not an accepted value for a tool designed to discover novel miRNA. Is there any parameter the author could tweak towards a better performance? For example, is the seed length of 18nt too short to start with? Are there any other

sequence features the authors should take into account to boost the performance? Or maybe some post-assembly filtering approaches might be sufficient and helpful.

We followed the reviewer's recommendations and we were able to improve the precision of BrumiR while maintaining a similar sensitivity. The major gain in the precision of BrumiR comes from a reduced seed length, which allows it to better deal with sequencing errors, and from an improved clustering of the reads coming from the same miRNA candidates, plus some code improvements. Overall, our precision for animals and plants increased by 1.67X and 4.09X on average respectively, which led to a combined F-score increased by 1.5X and 3X for animals and plants respectively, significantly reducing the rate of false-positives of our previous version. This can be further reduced when a reference genome is available and BrumiR2Reference is used. Additionally, we also provided a new script to annotate the candidates using the miRBase entries, providing a subset of high confidence BrumiR predictions. The novel candidates can be further refined using additional criteria (such as coverage, presence on several miRNA-seq experiments, etc.) that we describe in Figure 4 and Supplementary FigureS10, in the discovery of novel miRNAs in the Arabidopsis genome using BrumiR.
Moreover, we intend to improve our method by implementing a random forest classifier that will enable a better characterization of the novel candidates not present in miRbase. The random forest model learns the sequence features of the miRNA annotated in miRbase and can be used to assign a probability of being a miRNA or not for novel BrumiR candidates for species with or without a reference genome. This feature should be included in a future version of our method.
However, at this point we believe that in the absence of a reference genome, BrumiR is able to provide a list of potential miRNA candidates which is not usually possible with the other genome-based prediction tools that have a similar prediction power. We compared BrumiR against the best genome based miRNA discovery tool and we had comparable or better results, even without using a reference genome.

The updated text is in page 8, lines 13; page 12, lines 3-15 ; page 13, lines 1-3; page 14, lines 21-22 ; page 17, lines 5-6; page 23, lines 1-13; page 25, lines 18-22; page 26, lines 1-4.

5. Wet-lab validation (e.g. Luciferase assay) for the identified novel miRs will leverage the real-life usefulness of BrumiR. This is extremely important, as the tool showed a high false discovery rate.
We agree that functional validation of miRNAs predicted by BrumiR is an important point in order to highlight the usefulness of our tool; and we intend to continue our studies with Arabidopsis' novel miRNA candidates in the near future to provide additional evidence towards the in vivo functions of such candidates in a more specialized manuscript. Unfortunately, this is not within the scope of our study and more specifically the current global pandemic has not helped us finish such functional studies, because this part of the work has been done in Chile where our partner wet-lab was closed most of the part of the pandemic and currently is resuming is normal operation.

Even so, we would like to point out that current criteria to validate and annotate miRNAs in plants are based on experimental evidence coming directly from the sequencing libraries and that confirmation by blot of the expression of the miRNA or miRNA* is disallowed, as proposed by Axtell 2018*. What we think could be done in order to improve the sRNA-seq dataset instead would be to enrich the sequencing libraries with functional sRNAs coupled to proteins (as proposed in Grentzinger et al., 2014**). At this point, we believe that we currently fulfill several state-of-the-art criteria for miRNA annotation in plants. We have revised all the criteria exposed in Axtell, 2018* for novel miRNA candidates predicted by BrumiR on the Arabidopsis miRNA-seq data. We concentrated our effort on 22 novel miRNA candidates that were expressed in at least 3 replicates, and we found that 4 of them fulfilled at least 7 of 8 criteria, with 2 of them fulfilling all the criteria exposed in Axtell 2018. We have updated this part of the manuscript (Figure 4, Figure S10, and Table S8, S9, S10) accordingly to reflect these changes and we thank the reviewer for pointing out the need for more comprehensive criteria to reflect the accuracy of BrumiR when predicting new candidates.

(*) Axtell, M. J., & Meyers, B. C. (2018). Revisiting criteria for plant microRNA annotation in the era of big data. The Plant Cell, 30(2), 272-284.
(**) Grentzinger T, Armenise C, Pelisson A, Brun C, Mugat B, Chambeyron S. A user-friendly chromatographic method to purify small regulatory RNAs. Methods. 2014 May 1;67(1):91-101. doi: 10.1016/j.ymeth.2013.05.011. Epub 2013 May 28. PMID: 23727218.

The updated text is in page 19, lines 6-14; page 19, lines 21-23; page 20, lines 1-11; page 21, lines 1-10; page 38, lines 17-24.

Minor comments:
1. MiRNA maturation involves RNA editing. Can the authors comment on how this would be handled and captured by BrumiR. It seems that the authors allow mismatches when clustering the potential miRNAs via edlib library. It is interesting to know whether or not, or to what extent, edlib would help in including RNA edited candidates in the final result.

We use the edit distance library to allow a maximum of 2 nt differences in the cluster step of BrumiR. Then, for each cluster, we select the most expressed candidates but a reference is kept for all the cluster members. It is possible to use these clusters to identify miRNA candidates that may be RNA edited but the final output of BrumiR contains only one representative member of each cluster. We have included a flag in the output of BrumiR that reports the size of the cluster for each miRNA candidate. This allows us to explore if a candidate might be RNA edited or not. This is provided in a list as one of the BrumiR output files (*.edit_clusters.ED2.txt). Overall, BrumiR permits a mismatch maximum of two nucleotides when clustering candidates and therefore allows for the identification of candidates with potential RNA edition.

Reviewer #2:
The authors here present BrumiR, a de Bruijn-based method to discover miRNAs independently of a reference genome. Today most miRNA discovery and annotation is done by mapping sequenced RNAs to readily available reference genomes and analyzing the mapping profiles. However, there are some uses cases where the genome-free approach is needed (particularly for species that have no reference genome or where the genomes have missing parts); therefore BrumiR could potentially be useful for the community. However, the comparison to existing tools needs to be done in a more careful way.

Major comments:
1- RFAM filtering is not really part of the prediction step, this is rather a filtering step. Therefore, to make a fair comparison with mirnovo (the other genome-free tool), BrumiR should additionally be run without RFAM filtering, and mirnovo should additionally be run using the exact same RFAM filtering.

We used the RFAM database to filter out other kinds of RNAs present in the miRNA-seq experiment. Mirnovo calculates a consensus sequence for each cluster (candidate) and then an alignment is performed against the RFAM database to identify candidates matching rRNAs and tRNAs, and these matching candidates are filtered out by mirnovo. The latter step is performed by mirnovo by default and cannot be turned off (there is not a parameter allowing this). In BrumiR, we used the RFAM database to filter RNAs not associated with miRNAs. We built a k-mer database excluding all the RFAM families annotated as miRNAs. All the BrumiR candidates matching this k-mer database are excluded. This step is similar for both tools with the same goal (excluding other RNAs) but using different algorithms (alignment vs k-mer matches). We therefore considered that the comparison that we did is appropriate and fair for both tools. On average, 1% of the BrumiR candidates are filtered-out on this step.

The updated text is in page 9, lines 19-21.

2- it appears that 16-mers from miRBase miRNAs were specifically excluded from the RFAM catalog used for the filtering, which is reasonable. However, the miRNAs from the exact benchmarked species should not be included in the used miRBase 16-mer catalog, to avoid circular reasoning.

Yes, we agree with the reviewer that including miRNAs in this step may generate circular reasoning. This represents a final step of BrumiR where the aim is to remove sequences related to rRNAs and tRNAs, but we do not annotate or guide BrumiR towards previously annotated miRNA sequences present in the RFAM database. To achieve this, the 16-mer database was built from the RFAM database excluding all the families in all the taxa related to miRNA genes (529 families excluded), conserving the most abundant k-mers (more abundant than 5). Moreover, all 16-mers present in the miRBase database were also excluded. In summary, we excluded any reference to known miRNA sequences present in RFAM from the 16-mer database.

The updated text is in page 9, lines 19-21.

3- miRDeep2 software should ideally not be run with default options - this is particularly important since the miRDeep2 performance in this manuscript appears lower than what is reported in other studies (e.g. Friedlander et al. 2012). First, reference mature miRNAs from a related and well-annotated species should be included to support the prediction. Second, a score cut-off should be used that gives a decent signal-to-noise ratio according to the miRDeep2 output overview table (for instance 5:1). Third, all read pre-processing and genome mapping should be performed with the mapper.pl script which is part of the miRDeep2 package.

We agree with the reviewer that the ideal case is to use miRDeep2 according to the recommendations suggested by the developers (Friedländer et al, 2012). For this reason, we first used the script proposed by the developers to map the reads to the reference genome (mapper.pl). Then, we used the miRDeep2 pipeline in a “de novo” mode because we wanted to make a prediction without using previous knowledge, as we did with mirnovo and BrumiR. Finally, we selected all the predictions with a minimum miRDeep2 score of 5.1 (signal-to-noise ratio).
Also, the benchmark presented in the miRDeep2 manuscript was performed in a different way, because they used a pool of several samples, and the metrics were computed using all predictions, and for this reason, they are not comparable to our benchmark. As we mentioned above, there is no standard for benchmarking the miRNA prediction methods, which means that each new method tries to make the comparison in its own way and the metrics used could be quite different depending on the design of the benchmark.
miRDeep2 presented a good performance in most of the datasets, having the highest precision rate. However, we observed that it is a very conservative method because it does not predict many new candidates, even using the "de novo" mode.

The updated text is in page 35, lines 8-9, lines 11-13; page 13, lines 17; page 14, lines 1-2; page 15, lines 1-3;.

4- it appears that only miRNA-derived sequences were included in the simulated data. In fact, real small RNA-seq data typically contains fragments from other known types of RNA and also sequences from unannotated parts from the genome. Therefore, the authors should use simulated data that also includes samples from RFAM and randomly sampled sequences from the reference genome (for instance 10% of each). Overall, the use of simulated sequence data could be put a bit in the background in this study, since real small RNA-seq data is in fact readily available these days and typically has a structure that is not easy to simulate. Further, there is little reason not to use real data, since the miRNAs in miRBase tend to be reasonably well curated for most species and therefore can function well as a gold standard for benchmarking.

We followed the reviewer’s suggestion and we added to our simulated data sequences from the RFAM and the genomes for each of the species included in the benchmark. Now the simulated data include 10% of sequences from RFAM and 10% of random genomic sequences. We have repeated the synthetic benchmark and the new results are reported in the corresponding section. We agree that it is hard to mimic the structure of real miRNA-seq experiments, but the simulated data provide a good overview of the performance of the tools as well as a more controlled way to compare such performance since all the expressed miRNA sequences are known, which may not be the case for real data. Finally, we also included an extensive benchmark for the same species using real miRNA-seq data (Supplementary TableS4) and used the miRbase entries as the gold standard for computing the benchmark metrics as proposed by the reviewer.

The updated text is in page 11, lines 11-17; page 34, lines 19-22; page 35, lines 1-2.

5- precision of BrumiR is in some cases lower than 0.2, for instance for one mouse dataset. From this dataset ~3000 mouse miRNAs are reported - the majority of which are not in miRBase and can reasonably be presumed to be false positives. The authors should comment on why this particular dataset appears to produce so many false positives for BrumiR - could this have to do with the prevalence of piRNAs that the software cannot easily discern from miRNAs? Also, the authors should reflect on what kind of use cases could tolerate these thousands of false positives. Would this be for generating candidates for downstream high-throughput validation?

We took into account the recommendations of Reviewer #1 which led to a considerable improvement in our predictions and improved the benchmarking metrics in general across all the datasets. After we changed the k-mer size to 14, we improved over all the datasets the precision rates of BrumiR (Supplementary TableS1, TableS4). Moreover, to address the question of Reviewer #2 related to the

possibility of having piRNAs predicted as putative miRNAs in this dataset, we compared the predictions of BrumiR against the repository of piRNAs annotated for Mus musculus (https://www.pirnadb.org/). We observed that less than 4% of BrumiR candidates have a match with a piRNAs annotated in this database (Supplementary TableS9), a 3% and 10% of predictions of miRDeep2 and mirnovo, respectively, have a match with piRNAs. We can observe that none of the miRNA discovery tools would be capturing piRNAs instead of miRNAs, according
to the data presented here.

The updated text is in page 12, lines 3-15; page 14, lines 20-21; page 15, line 1-3; page 23, lines 10-13.

6- the authors should either benchmark BrumiR against the genome-free methods miReader and MirPlex, or explain why this comparison is not relevant.

We used mirnovo as a de novo predicted tool. As in the mirnovo paper, the authors compare mirnovo against these tools, miReader and MirPlex, and mirnovo has the best performance, we chose to compare BrumiR only against the best performance tools only in a genome-based and de novo approach, meaning against respectively miRDeep2 and mirnovo, as we explained above we compared BrumiR with 4 different miRNA discovery tools using a reduced version of the real dataset. The results provided in Supplementary TableS5 showed that the highest F-score rates are for BrumiR, miRDeep2, miR-PREFeR and mirnovo (Supplementary FigureS9). We decided to benchmark BrumiR in a simple but exhaustive way selecting the top performers for genome- based and genome-free methods.

The updated text is in page 13, lines 17; page 14, lines 1-2; page 36, lines 11-16.

Minor comments:
- the brief introduction to miRNA biology should be carefully edited by an expert in the field. Currently, very old reviews are being cited (e.g. Bartel 2004), and some of the other references appear to be a bit spurious (e.g. why focus on plant host-pathogen interactions out of the hundreds of established functions of miRNAs?). The excellent review of Dave Bartel from 2018 contains references to numerous milestone studies that the introduction could build on.
We included miRNA biology background and we verified our references. Thanks to the reviewers for having pointed this out.

The updated text is in page 3, lines 6-20.

- the authors write on page 2 that genome-based methods struggle with a high rate of false positive prediction, citing [9]. However, this is a mis-reference, since the reference [9] states that methods that rely on _only_ the genome and do not leverage on small RNA-seq data have high false positive rates.
We changed this sentence.

The updated text is in page 4, lines 20-21.

Reviewer #3:
The manuscript by Moraga et al. describes BrumiR, a software devoted to the de novo identification of miRNAs from deep sequencing experiments of the RNA fraction at low molecular weight.
In contrast with existing tools, BrumiR is based on de Bruijn graphs, generated directly from raw fastq reads. The performances on simulated and real sequencing data, in terms of precision, recall and F-Score, are very good. In addition, the tool is ultra-fast, enabling the analysis of huge amount of data.
I have tried to use BrumiR but I always got a GLIB error. I have tested the script on different Linux and Mac computers but I was not able to fix the GLIB error. It seems that a very recent version of the GLIB library is required. So, unfortunately, I didn't have the possibility to test the program and look at the outputs.
Major concerns:

1- I was not able to run the program and, thus, provide a correct revision. In my opinion, the github page should take into account this by providing the minimal software and hardware architecture to run BrumiR. Authors could also include a copy of the output files (by the way, there is a typo in the description of the second output file).

We do thank the reviewer for raising this point and we created a docker container that provides all the needed software to facilitate the execution of BrumiR. The docker image is available on dockerHub at (https://hub.docker.com/repository/docker/camoragaq/BrumiR) and instructions for downloading the image are provided on the Github repository of BrumiR. Additionally, we have created a demo dataset hosted at https://github.com/camoragaq/BrumiR_demo where we have included all the expected output files for the demo dataset.

The updated text is in page 40, lines 4-6.

2- Since the tool is able to identify novel miRNAs and look also at known ones, they could provide an output file including the read count per miRNA. In addition, since the tool is expected to be ultra-fast (not checked … see above), the differential gene expression analysis could also be implemented.

BrumiR outputs an abundance estimation based on the k-mer counts for each predicted miRNA candidate. Currently, BrumiR quantify each candidate using the total number of k-mers (KC), and the average number of k-mers per base (KM). Both values can be used as proxy to access the expression of each miRNA candidate. However, how has been previously shown for transcriptome mRNAseq data the accurate estimation of gene expression based on k-mers require the development of specific algorithms (Kallisto, Salmon) (Bray et al., 2016*) (Patro et al., 2017**). The adaptation of these algorithmics ideas to miRNAseq data is one of the main ideas that we are going to develop in a subsequent manuscript of the BrumiR toolkit.
(*) Bray, N., Pimentel, H., Melsted, P. et al. Near-optimal probabilistic RNA-seq quantification. Nat Biotechnol 34, 525–527 (2016). https://doi.org/10.1038/nbt.3519
(**) Patro, R., Duggal, G., Love, M. I., Irizarry, R. A., & Kingsford, C. (2017). Salmon provides fast and bias-aware quantification of transcript expression. Nature methods, 14(4), 417–419. https://doi.org/10.1038/nmeth.4197

3- I suggest also implementing a graphical output. A sort of summary in a decorated html page.

We followed the reviewer's suggestion and we now provide an R notebook to create an interactive and graphical summary of the output of BrumiR. We do plan to extend these capabilities in the future to further enhance the graphical report (i.e. by providing functions to quickly determine differentially expressed miRNAs). A full example of the R Notebook is provided with the demo dataset.

The updated text is in page 40, lines 5-6.

4- By using BrumiR, authors analyze miRNAs in Arabidopsis during the development, discovering three novel miRNAs. Although bioinformatics evidences indicate that they could be real miRNAs, an experimental validation is required. Indeed, these miRNAs have been detected by BrumiR only. I think that this validation could be easily done because authors directly performed sRNAseq data. In my opinion, this experiment could really improve the manuscript and assess the high performance of BrumiR.

We agree with the reviewer that experimental validation of the candidate miRNAs is an important step and a valuable suggestion for our work. Following the reviewer's suggestion, we have revised all the updated criteria on plant miRNA annotation based on Axtell, 2018* for each novel candidate miRNA predicted by BrumiR using the Arabidopsis thaliana datasets. These criteria are based directly on precursor and miRNA features that can be determined directly from the sRNA-Seq reads. Considering these criteria, we found that 2 of our miRNA candidates fulfill all of them (Figure 4, Figure S8, S9, S10). As such, these results highlight the usefulness of BrumiR for predicting new candidate miRNAs even in model species where the miRNA catalogs are highly complete. Although, this last results section of the BrumiR manuscript highlight the power of BrumiR to discover new miRNAs even in highly curated genomes, we believe that the biological insight and subsequent validations may be the focus of a more specialized manuscript rather than the current one where the focus is the new algorithmic ideas that we are presenting.

(*) Axtell, M. J., & Meyers, B. C. (2018). Revisiting criteria for plant microRNA annotation in the era of big data. The Plant Cell, 30(2), 272-284.

The updated text is in page 19, lines 6-14; page 19, lines 21-23; page 20, lines 1-11; page 21, lines 1-10; page 38, lines 17-24.

Again, we would like to thank all referees for their crucial suggestions that inspired us to further improve BrumiR, led to remarkable new benchmark results, and made the flow of the Results part more natural.
Sincerely,
Carol Moraga on behalf of all authors,

Close
