# Supplementary material for: BrumiR: A toolkit for de novo discovery of microRNAs from sRNA-seq data
Source: Gigascience. 2022 Oct 25;11:giac093. doi: 10.1093/gigascience/giac093 (PMC9596168; doi:10.1093/gigascience/giac093)
Supplement: giac093_Response_to_Reviewer_Comments_Revision_1 [file giac093_response_to_reviewer_comments_revision_1.pdf]

Author's Response To Reviewer Comments

Close

GIGA-D-20-00262R1
BrumiR: A toolkit for de novo discovery of microRNAs from sRNA-seq data.
Carol Moraga; Evelyn Sanchez; Mariana Galvao Ferrarini; Rodrigo A Gutierrez; Elena A Vidal; Marie-France Sagot GigaScience
Dear Dr. Moraga,
Your manuscript "BrumiR: A toolkit for de novo discovery of microRNAs from sRNA-seq data." (GIGA-D-20-00262R1) has been assessed by our reviewers. Although it is of interest, we are unable to consider it for publication in its current form. The reviewers and Editorial Board member have raised a number of points which we believe would improve the manuscript and may allow a revised version to be published in GigaScience.
Reviewer #1 is happy with the revisions made. However, Reviewer #2 was still not happy with the revisions and decided not to submit a formal review; however, they have provided some detailed and useful feedback. We then had to seek advice from an Editorial board member who has considered the feedback from Reviewer #2 and they have s suggested the following major revisions, which we feel is fair, and must be made in order for us to consider this manuscript for GigaScience.
Overall, there is a concern with regards to the robustness of the tool, and this must be proven.
19th July 2022
Dear Dr. Nogoy,
Thank you very much for considering our manuscript for publication in GigaScience. We would like to thank the referees, the Editorial Board member, and you for the careful assessment of our manuscript. We have attempted to address all points raised by the referees putting special emphasis on the precision of our method and hope that the responses are satisfactory. With these revisions, we believe that our manuscript has been substantially improved and hope that it is now suitable for publication in GiGaScience.
Please find below our point-by-point replies to the reviewers’ comments. All changes in the main manuscript and the supplement have been marked in blue font. We have reformatted the manuscript according to the guidelines of GiGaScience.
Yours sincerely,
Carol Moraga

1) Both reviewer 1 and myself noted the low precision of BrumiR on many datasets. The authors have improved precision by using a seed size of 14, but the precision is still down to around 0.35 for the C. elegans datasets using BrumiR and down to ~0.5 when using BrumiR2Reference (Figure 3B). Further, BrumiR still reports ~1500 miRNA candidates for one mouse dataset (Figure 3A), most of which are likely to be false positives given that mouse miRNA annotation is by now fairly saturated. The authors provide compelling evidence that these novel candidates are not piRNAs, but this does not fully clarify the matter, since it is still not clear what they are. The concern raised by reviewer 1 and myself thus remains - what is the use for software that generates so many false positives? This should also be seen in the light that miRBase already contains many false positives that can confound e.g. evolutionary analyses. This topic is discussed here:
https://academic.oup.com/nar/article/48/D1/D132/5584683
Our Editorial Board member suggests you must clarify the question "Whether the de novo miRNAs really exist?"
We agree that the BrumiR precision might seem low in some datasets and that the results presented in the mouse dataset seem problematic. To improve the BrumiR precision we have explored new methods and algorithms. After thoroughly examining the BrumiR code, we attempted to resolve the precision issue as a classification problem and implemented/designed a supervised machine learning method (random forest) to classify BrumiR candidates using an additional and new BrumiR tool (brumirRF.pl). The random forest model is composed of 19 features, of which 16 are inferred directly from 15-mer sequences of each BrumiR candidate and three derived from nucleotide composition observed on reference mature miRNA sequences (miRGeneDB and miRbase). The 16 derived features are GC content(gc), GC skew content (gcs), CpG content (cpg), sequence complexity by Wootton & Federhen (cwf), sequence Shannon entropy (ce), sequence complexity of Markov model values (cm1,cm2,cm3),

sequence complexity by Trifonov values (ct3,ct4,ct5,ct6) and sequence complexity linguistic values (cl3,cl4,cl5,cl6). The nucleotide composition are 6-mer, 7-mer, and 8-mer observed frequency of mature miRNA sequences on reference miRNA databases (MirGeneDB and miRBase). The features are computed on a 15-mer basis to classify any length of miRNA candidates (18-22 base pairs). We built one model for animals using the curated entries from MirGeneDB as suggested by Editorial board member. A total of 35570 15-mer were derived from the MiRGeneDB, and all the 19 features were computed for each. A matching amount of 15-mer random sequences were generated, and all the 19 features were computed for each. The whole training and evaluation dataset comprised 71140 15-mers of the two classes (random and mature miRNAs sequences). The training and evaluation of the random forest were performed using 75% and 25%, respectively. The benchmark results show that the random forest classifier achieves an accuracy of 90%, a precision of 87%, and a recall of 94% for discriminating miRNAs 15-mers from random 15-mers. The most top-5 informative features were 8-mer, 7-mer, 6-mer, CpG content, GC content, and the complexity of markov models. Another model using miRBase entries and the same features achieves an accuracy of 90%, a precision of 87%, and a recall of 93% for discriminating plant miRNAs 15-mers from random 15-mer sequences. The building and evaluation of both random forest models are available on the BrumiR GitHub repository (here: https://github.com/camoragaq/BrumiR/tree/master/brumir-rf). Our major aim in implementing this classifier was to keep the BrumiR recall and increase the precision, therefore, reducing the false positive rate. We evaluated the performance of this classifier on the mouse dataset, prior to incrementing the minimal candidate coverage to 50X, which is now the default for reporting a miRNA candidate (before was 20X). The number of candidates for this mouse sample is now 934 and after applying the random forest it decreases to 490 candidates, which results in a recall of 87%, precision of 60%, and F-Score of 70%. The previous number represents an increase of 3,5 fold in precision (previously was 17%). We do want to remark that the only information about know miRNA sequences is the composition of 6-mer, 7-mer, and 8-mer. Therefore, BrumiR uses little information of know miRNAs sequences. In the current random forest implementation, we do classify BrumiR candidates as potential miRNAs sequences when candidates have a random forest probability greater than 0.8. Similar results were observed on the other evaluated datasets (Figure 4), therefore the random forest classifier allowed us to increase the BrumiR precision without affecting the BrumiR recall. The new BrumiR tool is described in the main text in the section "Using a supervised Machine Learning approach to refine the BrumiR-core predictions".
2) Our Editorial board member also requests pairwise performance data comparison of BrumiR and a manually curated and open-source miRNA gene database, MirGeneDB, which is based on consistent annotation and nomenclature criteria. The database contains high-quality annotations of 10 899 bonafide and consistently named miRNAs constituting 1275 miRNA families from 45 species, representing every major metazoan group, including many well-established and emerging invertebrates and vertebrate model organisms.
We have followed the reviewer recommendations and we computed the performance of BrumiR and those of other methods using the MirGeneDB database on 5 animal datasets (with two replicates) which are the ones with entries in the MirGeneDB. The new benchmark shows that BrumiR achieves the highest F-Score (9/10 datasets) with an average F-Score of 0.53 while their competitors achieve 0.3 and 0.36 for mirDeep2 and mirnovo, respectively. Regarding the precision, we have been aware of the low precision of all the methods, over the revisions we have improved the BrumiR precision (first report) and now we improved substantially by the use of a random forest classifier (see the answer to question 1) from X to Y. Final values of benchmark metrics are available on supplementary table X and discussions about BrumiR precision are provided on the new manuscript section "Using a supervised Machine Learning approach to refine the BrumiR-core predictions". We do thank the reviewer for pointing out the MirGeneDB database which was key for inspiring us to further increase the precision of our method by means of a machine learning classifier.
3) In addition, a fair comparison to other de novo tools would be nice to further support the work, and a small-scale validation would be a big plus, but may not be necessary if the miRNA species from MirGeneDB could be predicted accurately.
We evaluated all methods' performance using the MirGeneDB and miRBase databases. We observe that BrumiR outperforms current approaches and is the only tool that generates reliable results in the absence of a reference genome. We want to remark that this version is the first one of our method and that further improvements are still possible (regarding precision and recall). Regarding experiments, we have included extensive Arabidopsis data showing the utility of our method by discovering high-quality novel miRNAs even in the reference plant model Arabidopsis thaliana. Unfortunately, further experimental validation of the BrumiR candidates has been out of our reach, and we do plan to perform it shortly after securing funding for further development of BrumiR. Still, as one of the reviewers pointed out, this is the first tool that implements a Bruijn Graph approach, and additional improvement from the community or us might be possible after BrumiR publication.

Please also take a moment to check our website at https://www.editorialmanager.com/giga/ for any additional comments that were saved as attachments.
In addition, please register any new software application in the bio.tools and SciCrunch.org databases to receive RRID (Research Resource Identification Initiative ID) and biotoolsID identifiers, and include these in your manuscript. This will facilitate tracking, reproducibility and re-use of your tool.
If you are able to fully address these points, we would encourage you to submit a revised manuscript to GigaScience. Once you have made the necessary corrections, please submit online at:
https://www.editorialmanager.com/giga/
If you have forgotten your username or password please use the "Send Login Details" link to get your login information. For security reasons, your password will be reset.
Please include a point-by-point within the 'Response to Reviewers' box in the submission system. Please ensure you describe additional experiments that were carried out and include a detailed rebuttal of any criticisms or requested revisions that you disagreed with. Please also ensure that your revised manuscript conforms to the journal style, which can be found in the Instructions for Authors on the journal homepage. If the data and code has been modified in the revision process please be sure to update the public versions of this too.
The due date for submitting the revised version of your article is 09 Mar 2022. I look forward to receiving your revised manuscript soon.
Best wishes,
Nicole Nogoy, Ph.D GigaScience
Reviewer reports:

Reviewer #1: The authors have significantly improved their manuscript, especially by fixing all my previous questions regarding the performance of BrumiR. Although my question about wet-lab validation has not been fully settled, I agree with the authors that it can be considered as out of the scope of this study.
The revised BrumiR outperformed some of the commonly used tools in the miRNA prediction field. The overall performance, in terms of F1-score, is on the top among the comparisons, despite the potential to further improve its precision.
In summary, I believe BrumiR is a solid tool for miRNA prediction, and its idea of using de Bruijn graph and reference-free approaches could inspire further application development in the genetic field. This manuscript is ready to be accepted by Giga Science.

Again, we would like to thank all referees, the Editorial Board member, and you for the crucial suggestions that inspired us to extend the range of applications for BrumiR (BrumiR random forest), which led to improved new benchmark results that make the BrumiR predictions more robust.
Sincerely,
Carol Moraga on behalf of all authors

Close
