# Supplementary material for: BrumiR: A toolkit for de novo discovery of microRNAs from sRNA-seq data
Source: Gigascience. 2022 Oct 25;11:giac093. doi: 10.1093/gigascience/giac093 (PMC9596168; doi:10.1093/gigascience/giac093)
Supplement: giac093_Response_to_Reviewer_Comments_Revision_2 [file giac093_response_to_reviewer_comments_revision_2.pdf]

Author's Response To Reviewer Comments

Close

Dear Nicole,

Following the last revisions, we have completed all the editorial requirements regarding the format and availability of datasets and code.
We provide the manuscript in the format required by GigaScience, and in an editable format for production.

Thanks and best regards,

Carol

Close
