# Supplementary material for: BrumiR: A toolkit for de novo discovery of microRNAs from sRNA-seq data
Source: Gigascience. 2022 Oct 25;11:giac093. doi: 10.1093/gigascience/giac093 (PMC9596168; doi:10.1093/gigascience/giac093)
Supplement: giac093_Reviewer_1_Report_Original_Submission — Dadi Gao -- 9/20/2020 Reviewed [file giac093_reviewer_1_report_original_submission.pdf]

Reviewer Report

Title: BrumiR: A toolkit for de novo discovery of microRNAs from sRNA-seq data.

Version: Original Submission **Date:** 9/20/2020

Reviewer name: Dadi Gao

Reviewer Comments to Author:

Summary:
The authors developed a de novo assembly method, BrumiR, for small RNA sequencing data based on de Bruijin graph algorithm. This tool displayed a relatively high sensitivity in finding miRNAs and helped the authors discover a novel miRNA in A. thaliana roots.
Major comments:
1. Have the authors compare the performance with different seed length? Even if the minimal miR length is 18nt in MiRBase 21, seed=18 might not necessarily lead to the best AUC or F score (This might also be related to Comment 4).
2. The authors need to benchmark BrumiR with more existing tools (e.g. those ML-based methods), and to include more genome-free methods (e.g. MiRNAgFree).
3. It is also interesting to know whether de novo method for mRNA assembly would be useful on the miRNA side. It would be great if the authors were able to compare the performance of BrumiR2reference (without filtering for RFAM) with Trinity in genome-guided mode, by tweaking its seed length to be the same as BrumiR.
4. The tool's sensitivity is promising across animal and plant datasets. However, the average precision is quite low, an average precision of 0.3 means a false discovery rate of 0.7. This is not an accepted value for a tool designed to discover novel miRNA. Is there any parameter the author could tweak towards a better performance? For example, is seed length of 18nt too short to start with? Is there any other sequences feature the authors should take into account to boost the performance? Or maybe some post-assembly filtering approaches might be sufficient and helpful.
5. Wet-lab validation (e.g. Luciferase assay) for the identified novel miRs will leverage the real-life usefulness of BrumiR. This is extremely important, as the tool showed a high false discovery rate.
Minor comments:
1. MiRNA maturation involves RNA editing. Can the authors comment on how this would be handled and captured by BrumiR. It seems that the authors allow mismatches when cluster the potential miRNAs via edlib library. It is interesting to know whether or not, or to what extent, edlib would help in including RNA edited candidates in the final result.

Level of Interest

Please indicate how interesting you found the manuscript: Choose an item.

Quality of Written English

Please indicate the quality of language in the manuscript: Choose an item.

Declaration of Competing Interests

Please complete a declaration of competing interests, considering the following questions:

- Have you in the past five years received reimbursements, fees, funding, or salary from an organisation that may in any way gain or lose financially from the publication of this manuscript, either now or in the future?
- Do you hold any stocks or shares in an organisation that may in any way gain or lose financially from the publication of this manuscript, either now or in the future?
- Do you hold or are you currently applying for any patents relating to the content of the manuscript?
- Have you received reimbursements, fees, funding, or salary from an organization that holds or has applied for patents relating to the content of the manuscript?
- Do you have any other financial competing interests?
- Do you have any non-financial competing interests in relation to this paper?

If you can answer no to all of the above, write 'I declare that I have no competing interests' below. If your reply is yes to any, please give details below.

I declare that I have no competing interests.

I agree to the open peer review policy of the journal. I understand that my name will be included on my report to the authors and, if the manuscript is accepted for publication, my named report including any attachments I upload will be posted on the website along with the authors' responses. I agree for my report to be made available under an Open Access Creative Commons CC-BY license (http://creativecommons.org/licenses/by/4.0/). I understand that any comments which I do not wish to be included in my named report can be included as confidential comments to the editors, which will not be published.

Choose an item.

To further support our reviewers, we have joined with Publons, where you can gain additional credit to further highlight your hard work (see: https://publons.com/journal/530/gigascience). On publication of this paper, your review will be automatically added to Publons, you can then choose whether or not to claim your Publons credit. I understand this statement.

Yes Choose an item.
