# Supplementary material for: BrumiR: A toolkit for de novo discovery of microRNAs from sRNA-seq data
Source: Gigascience. 2022 Oct 25;11:giac093. doi: 10.1093/gigascience/giac093 (PMC9596168; doi:10.1093/gigascience/giac093)
Supplement: giac093_Reviewer_2_Report_Original_Submission — Marc Friedlander -- 9/25/2020 Reviewed [file giac093_reviewer_2_report_original_submission.pdf]

Reviewer Report

Title: BrumiR: A toolkit for de novo discovery of microRNAs from sRNA-seq data.

Version: Original Submission **Date:** 9/25/2020

Reviewer name: Marc Friedlander

Reviewer Comments to Author:

The authors here present BrumiR, a de Bruijn-based method to discover miRNAs independently of a reference genome. Today most miRNA discovery and annotation is done by mapping sequenced RNAs to readily available reference genomes and analyzing the mapping profiles. However, there are some uses cases where the genome-free approach is needed (particularly for species that have no reference genome or where the genomes have missing parts); therefore BrumiR could potentially be useful for the community. However, the comparison to existing tools needs to be done in a more careful way.
Major comments:
- RFAM filtering is not really part of the prediction step, this is rather a filtering step. Therefore, to make a fair comparison with mirnovo (the other genome-free tool), BrumiR should additionally be run without RFAM filtering, and mirnovo should additionally be run using the exact same RFAM filtering.
- it appears that 16-mers from miRBase miRNAs were specifically excluded from the RFAM catalog used for the filtering, which is reasonable. However, the miRNAs from the exact benchmarked species should not be included in the used miRBase 16-mer catalog, to avoid circular reasoning.
- miRDeep2 software should ideally not be run with default options - this is particular important since the miRDeep2 performance in this manuscript appears lower than what is reported in other studies (e.g. Friedlander et al. 2012). First, reference mature miRNAs from a related and well-annotated species should be included to support the prediction. Second, a score cut-off should be used that gives a decent signal-to-noise ratio according to the miRDeep2 output overview table (for instance 5:1). Third, all read pre-processing and genome mapping should be performed with the mapper.pl script which is part of the miRDeep2 package.
- it appears that only miRNA-derived sequences were included in the simulated data. In fact, real small RNA-seq data typically contains fragments from other known types of RNA and also sequences from unannotated parts from the genome. Therefore, the authors should use simulated data that also includes samples from RFAM and randomly sampled sequences from the reference genome (for instance 10% of each). Overall, the use of simulated sequence data could be put a bit in the background in this study, since real small RNA-seq data is in fact readily available these days and typically has a structure that is not easy to simulate. Further, there is little reason not to use real data, since the miRNAs in miRBase tend to be reasonably well curated for most species and therefore can function well as a gold standard for benchmarking.
- precision of BrumiR is in some cases lower than 0.2, for instance for one mouse dataset. From this dataset ~3000 mouse miRNAs are reported - the majority of which are not in miRBase and can reasonably be presumed to be false positives. The authors should comment on why this particular

dataset appears to produce so many false positives for BrumiR - could this have to do with the prevalence of piRNAs that the software cannot easily discern from miRNAs? Also, the authors should reflect on in what kind of use cases could tolerate these thousands of false positives. Would this be for generating candidates for downstream high-throughput validation?
- the authors should either benchmark BrumiR against the genome-free methods miReader and MirPlex, or explain why this comparison is not relevant.
Minor comments:
- the brief introduction to miRNA biology should be carefully edited by an expert in the field. Currently, very old reviews are being cited (e.g. Bartel 2004), and some of the other references appear to be a bit spurious (e.g. why focus on plant host-pathogen interactions out of the hundreds of established functions of miRNAs?). The excellent review of Dave Bartel from 2018 contains references to numerous milestone studies that the introduction could build on.
- the authors write on page 2 that genome-based methods struggle with a high rate of false positive prediction, citing [9]. However, this is a mis-reference, since the reference [9] states that methods that rely on _only_ the genome and do not leverage on small RNA-seq data have high false positive rates.

Level of Interest

Please indicate how interesting you found the manuscript: Choose an item.

Quality of Written English

Please indicate the quality of language in the manuscript: Choose an item.

Declaration of Competing Interests

Please complete a declaration of competing interests, considering the following questions:

- Have you in the past five years received reimbursements, fees, funding, or salary from an organisation that may in any way gain or lose financially from the publication of this manuscript, either now or in the future?
- Do you hold any stocks or shares in an organisation that may in any way gain or lose financially from the publication of this manuscript, either now or in the future?
- Do you hold or are you currently applying for any patents relating to the content of the manuscript?
- Have you received reimbursements, fees, funding, or salary from an organization that holds or has applied for patents relating to the content of the manuscript?
- Do you have any other financial competing interests?
- Do you have any non-financial competing interests in relation to this paper?

If you can answer no to all of the above, write 'I declare that I have no competing interests' below. If your reply is yes to any, please give details below.

I declare that I have no competing interests.

I agree to the open peer review policy of the journal. I understand that my name will be included on my report to the authors and, if the manuscript is accepted for publication, my named report including any attachments I upload will be posted on the website along with the authors' responses. I agree for my report to be made available under an Open Access Creative Commons CC-BY license (http://creativecommons.org/licenses/by/4.0/). I understand that any comments which I do not wish to be included in my named report can be included as confidential comments to the editors, which will not be published.

Choose an item.

To further support our reviewers, we have joined with Publons, where you can gain additional credit to further highlight your hard work (see: https://publons.com/journal/530/gigascience). On publication of this paper, your review will be automatically added to Publons, you can then choose whether or not to claim your Publons credit. I understand this statement.

Yes Choose an item.
