# Supplementary material for: BrumiR: A toolkit for de novo discovery of microRNAs from sRNA-seq data
Source: Gigascience. 2022 Oct 25;11:giac093. doi: 10.1093/gigascience/giac093 (PMC9596168; doi:10.1093/gigascience/giac093)
Supplement: giac093_Reviewer_3_Report_Original_Submission — Ernesto Picardi -- 10/1/2020 Reviewed [file giac093_reviewer_3_report_original_submission.pdf]

Reviewer Report

Title: BrumiR: A toolkit for de novo discovery of microRNAs from sRNA-seq data.

Version: Original Submission **Date:** 10/1/2020

Reviewer name: Ernesto Picardi

Reviewer Comments to Author:

The manuscript by Moraga et al. describes BrumiR, a software devoted to the de novo identification of miRNAs from deep sequencing experiments of the RNA fraction at low molecular weight.
In contrast with existing tools, BrumiR is based on de Bruijn graphs, generated directly from raw fastq reads. The performances on simulated and real sequencing data, in terms of precision, recall and F-Score, are very good. In addition, the tool is ultra-fast, enabling the analysis of huge amount of data.
I have tried to use BrumiR but I always got a GLIB error. I have tested the script on different Linux and Mac computers but I was not able to fix the GLIB error. It seems that a very recent version of the GLIB library is required. So, unfortunately, I didn't have the possibility to test the program and look at the outputs.
Major concerns:
- I was not able to run the program and, thus, provide a correct revision. In my opinion, the github page should take into account this by providing the minimal software and hardware architecture to run BrumiR. Authors could also include a copy of the output files (by the way, there is a typo in the description of the second output file).
- Since the tools is able to identify novel miRNAs and look also at known ones, they could provide an output file including the read count per miRNA. In addition, since the tool is expected to be ultra-fast (not checked … see above), the differential gene expression analysis could also be implemented.
- I suggest also to implement a graphical output. A sort of summary in a decorated html page.
- By using BrumiR, authors analyze miRNAs in Arabidopsis during the development, discovering three novel miRNAs. Although bioinformatics evidences indicate that they could be real miRNAs, an experimental validation is required. Indeed, these miRNAs have been detected by BrumiR only. I think that this validation could be easily done because authors directly performed sRNAseq data. In my opinion, this experiment could really improve the manuscript and assess the high performance of BrumiR.

Level of Interest

Please indicate how interesting you found the manuscript: Choose an item.

Quality of Written English

Please indicate the quality of language in the manuscript: Choose an item.

Declaration of Competing Interests

Please complete a declaration of competing interests, considering the following questions:

- Have you in the past five years received reimbursements, fees, funding, or salary from an organisation that may in any way gain or lose financially from the publication of this manuscript, either now or in the future?
- Do you hold any stocks or shares in an organisation that may in any way gain or lose financially from the publication of this manuscript, either now or in the future?
- Do you hold or are you currently applying for any patents relating to the content of the manuscript?
- Have you received reimbursements, fees, funding, or salary from an organization that holds or has applied for patents relating to the content of the manuscript?
- Do you have any other financial competing interests?
- Do you have any non-financial competing interests in relation to this paper?

If you can answer no to all of the above, write 'I declare that I have no competing interests' below. If your reply is yes to any, please give details below.

I declare that I have no competing interests.

I agree to the open peer review policy of the journal. I understand that my name will be included on my report to the authors and, if the manuscript is accepted for publication, my named report including any attachments I upload will be posted on the website along with the authors' responses. I agree for my report to be made available under an Open Access Creative Commons CC-BY license (http://creativecommons.org/licenses/by/4.0/). I understand that any comments which I do not wish to be included in my named report can be included as confidential comments to the editors, which will not be published.

Choose an item.

To further support our reviewers, we have joined with Publons, where you can gain additional credit to further highlight your hard work (see: https://publons.com/journal/530/gigascience). On publication of this paper, your review will be automatically added to Publons, you can then choose whether or not to claim your Publons credit. I understand this statement.

Yes Choose an item.
